# Effectiveness of interventions to reduce homelessness: a systematic review and meta‐analysis

**DOI:** 10.4073/csr.2018.3

**Published:** 2018-02-28

**Authors:** Heather Menzies Munthe‐Kaas, Rigmor C Berg, Nora Blaasvær

## Abstract

This Campbell systematic review examines the effectiveness of interventions to reduce homelessness and increase residential stability for individuals who are homeless, or at risk of becoming homeless. Forty‐three studies were included in the review, 37 of which are from the USA.

Included interventions perform better than the usual services at reducing homelessness or improving housing stability in all comparisons. These interventions are:

- High intensity case management
- Housing First
- Critical time intervention
- Abstinence‐contingent housing
- Non‐abstinence‐contingent housing with high intensity case management
- Housing vouchers
- Residential treatment

These interventions seem to have similar beneficial effects, so it is unclear which of these is best with respect to reducing homelessness and increasing housing stability.

**Plain Language Summary:**

**Interventions to reduce homelessness and improve housing stability are effective**

There are large numbers of homeless people around the world. Interventions to address homelessness seem to be effective, though better quality evidence is required.

**What is this review about?**

There are large numbers of homeless people around the world. Recent estimates are over 500,000 people in the USA, 100,000 in Australia and 30,000 in Sweden. Efforts to combat homelessness have been made on national levels as well as at local government levels.

This review assesses the effectiveness of interventions combining housing and case management as a means to reduce homelessness and increase residential stability for individuals who are homeless, or at risk of becoming homeless.

**What is the aim of this review?**

This Campbell systematic review examines the effectiveness of interventions to reduce homelessness and increase residential stability for individuals who are homeless, or at risk of becoming homeless. Forty‐three studies were included in the review, 37 of which are from the USA.

**What studies are included?**

Included studies were randomized controlled trials of interventions for individuals who were already, or at‐risk of becoming, homeless, and which measured impact on homelessness or housing stability with follow‐up of at least one year.

A total of 43 studies were included. The majority of the studies (37) were conducted in the United States, with three from the United Kingdom and one each from Australia, Canada, and Denmark.

**What are the main findings of this review?**

Included interventions perform better than the usual services at reducing homelessness or improving housing stability in all comparisons. These interventions are:

- High intensity case management
- Housing First
- Critical time intervention
- Abstinence‐contingent housing
- Non‐abstinence‐contingent housing with high intensity case management
- Housing vouchers
- Residential treatment

These interventions seem to have similar beneficial effects, so it is unclear which of these is best with respect to reducing homelessness and increasing housing stability.

**What do the findings of this review mean?**

A range of housing programs and case management interventions appear to reduce homelessness and improve housing stability, compared to usual services.

However, there is uncertainty in this finding as most the studies have risk of bias due to poor reporting, lack of blinding, or poor randomization or allocation concealment of participants. In addition to the general need for better conducted and reported studies, there are specific gaps in the research with respect to: 1) disadvantaged youth; 2) abstinence‐contingent housing with case management or day treatment; 3) non‐abstinence contingent housing comparing group vs independent living; 4) Housing First compared to interventions other than usual services, and; 5) studies outside of the USA.

**How up‐to‐date is this review?**

The review authors searched for studies published up to January 2016. This Campbell systematic review was published in February 2018.

**Executive summary:**

**Background**

The United Nations Universal Declaration of Human Rights (Article 25) states that everyone has a right to housing. However, this right is far from being realized for many people worldwide. According to the United Nations High Commissioner for Refugees (UNHCR), there are approximately 100 million homeless people worldwide. The aim of this report is to contribute evidence to inform future decision making and practice for preventing and reducing homelessness.

**Objectives**

To identify, appraise and summarize the evidence on the effectiveness of housing programs and case management to improve housing stability and reduce homelessness among people who are homeless or at‐risk of becoming homeless.

**Search methods**

We conducted a systematic review in accordance with the Norwegian Knowledge Centre's handbook. We systematically searched for literature in relevant databases and conducted a grey literature search which was last updated in January 2016.

**Selection criteria**

Randomized controlled trials that included individuals who were already, or at‐risk of becoming, homeless were included if they examined the effectiveness of relevant interventions on homelessness or housing stability. There were no limitations regarding language, country or length of homelessness. Two reviewers screened 2,918 abstracts and titles for inclusion. They read potentially relevant references in full, and included relevant studies in the review.

**Data collection and analysis**

We pooled the results and conducted meta‐analyses when possible. Our certainty in the primary outcomes was assessed using the Grading of Recommendations Assessment, Development, and Evaluation for effectiveness approach (GRADE).

**Results**

We included 43 relevant studies (described in 78 publications) that examined the effectiveness of housing programs and/or case management services on homelessness and/or housing stability. The results are summarized below. Briefly, we found that the included interventions performed better than the usual services in all comparisons. However, certainty in the findings varied from very low to moderate. Most of the studies were assessed as having high risk of bias due to poor reporting, lack of blinding, or poor randomization and/or allocation concealment of participants.

**Case management**

Case management is a process where clients are assigned case managers who assess, plan and facilitate access to health and social services necessary for the client's recovery. The intensity of these services can vary. One specific model is Critical time intervention, which is based on the same principles, but offered in three three‐month periods that decrease in intensity.

High intensity case management compared to usual services has generally more positive effects: It probably reduces the number of individuals who are homeless after 12‐18 months by almost half (RR=0.59, 95%CI=0.41 to 0.87)(moderate certainty evidence); It may increase the number of people living in stable housing after 12‐18 months and reduce the number of days an individual spends homeless (low certainty evidence), however; it may have no effect on the number of individuals who experience some homelessness during a two year period (low certainty evidence). When compared to low intensity case management, it may have little or no effect on time spent in stable housing (low certainty evidence).

Critical time intervention compared to usual services may 1) have no effect on the number of people who experience homelessness, 2) lead to fewer days spent homeless, 3) lead to more days spent not homeless and, 4) reduce the amount of time it takes to move from shelter to independent housing (low certainty evidence).

**Abstinence‐contingent housing programs**

Abstinence‐contingent housing is housing provided with the expectation that residents will remain sober. The results showed that abstinence‐contingent housing may lead to fewer days spent homeless, compared with usual services (low certainty evidence).

**Non‐abstinence‐contingent housing programs**

Non‐abstinence‐contingent housing is housing provided with no expectations regarding sobriety of residents. Housing First is the name of one specific non‐abstinence‐contingent housing program. When compared to usual services Housing First probably reduces the number of days spent homeless (MD=‐62.5, 95%CI=‐86.86 to ‐38.14) and increases the number of days in stable housing (MD=110.1, 95%CI=93.05 to 127.15) (moderate certainty evidence). In addition, it may increase the number of people placed in permanent housing after 20 months (low certainty evidence).

Non‐abstinence‐contingent housing programs (not specified as Housing First) in combination with high intensity case management may reduce homelessness, compared to usual services (low certainty evidence). Group living arrangements may be better than individual apartments at reducing homelessness (low certainty evidence).

**Housing vouchers with case management**

Housing vouchers is a housing allowance given to certain groups of people who qualify. The results showed that it mayreduce homelessness and improve housing stability, compared with usual services or case management (low certainty evidence).

**Residential treatment with case management**

Residential treatment is a type of housing offered to clients who also need treatment for mental illness or substance abuse. We found that it mayreduce homelessness and improve housing stability, compared with usual services (low certainty evidence).

**Authors’ conclusions**

We found that a range of housing programs and case management interventions appear to reduce homelessness and improve housing stability, compared to usual services. The findings showed no indication of housing programs or case management resulting in poorer outcomes for homeless or at‐risk individuals than usual services.

Aside from a general need for better conducted and reported studies, there are specific gaps in the research. We identified research gaps concerning: 1)Disadvantaged youth; 2) Abstinence‐contingent housing with case management or day treatment; 3) Non‐abstinence contingent housing, specifically different living arrangements (group vs independent living); 4) Housing First compared to interventions other than usual services, and; 5) All interventions from contexts other than the USA.

## Background

### Description of homelessness

The United Nations Universal Declaration of Human Rights (Article 25) states that everyone has a right to housing. However, this right is far from being realized for many people worldwide. According to the United Nations High Commissioner for Refugees (UNHCR), there are approximately 100 million homeless people worldwide (1).

#### Defining homelessness

The term “homeless” is defined differently according to context, purpose and the geographical setting. There are three basic domains for understanding “home” and “homelessness”: 1) the physical domain (the absence of home); 2) the social domain (homelessness connected to discrimination and social exclusion), and 3) the legal domain (individuals have a right to tenancy, and people without homes still have rights and are deserving of dignity) (2, 3).

In the European Union, four categories of homelessness have been developed: roofless, houseless, insecure housing and inadequate housing (3). In the United States, the Department of Housing and Urban Development defines a person as homeless “if he or she lives in an emergency shelter, transitional housing program (including safe havens), or a place not meant for human habitation, such as a car, abandoned building, or on the streets”(4). For the purpose of this review, the following Norwegian definition of homeless should be considered:

“A person is homeless when s/he lacks a place to live, either rented or owned, and finds themselves in one of the three following situations: Has no place to stay for the night; Is referred to an emergency or temporary shelter/accommodation; Is a ward of the correctional and probation service and due to be released in two months at the latest; Is a resident of an institution and due to be discharged in two months at the latest; Lives with friends, acquaintances or family on a temporary basis” (5).

A glossary of terms related to homelessness, relevant interventions and study characteristics is included in Appendix 1.

#### Causes of homelessness

In discussing causes of homelessness, it is important to think of two different but related questions: ‘Why does homelessness exist?’ and ‘Who is most vulnerable to becoming homeless?’ (6). As Paul Koegel describes in Homelessness Handbook, the structural context of homelessness (why?) includes “a growing set of pressures that included a dearth of affordable housing, a disappearance of the housing on which the most unstable relied, and a diminished ability to support themselves either through entitlements or conventional or makeshift labour” while the people most affected (who?) “disproportionately include those people least able to compete for housing, especially those vulnerable individuals who had traditionally relied on a type of housing that was at extremely high risk of demolition and conversion…high numbers of people with mental illness and substance abuse…individuals with other sorts of personal vulnerabilities and problems” (6).

#### Homelessness around the world

Although homelessness has been defined and measured differently, some important descriptive statistics from different countries indicate the importance of the problem. Given the various ways of measuring homelessness, the following statistics are not meant to be compared among each other. A recent report stated that in the USA on a given night in January 2015, almost 565,000 people were experiencing homelessness (sleeping outside, in shelter or in transitional housing) (4). Although homelessness in the USA has decreased by 2% from 2014 to 2015, this figure is still very high (4). Homelessness is also a serious problem in Europe: 34,000 people were defined as homeless in Sweden in 2011 (7), and 14,780 households were defined as unintentionally homeless in the United Kingdom in 2016 (8). In Canada, it is estimated that approximately 1% of the population (35,000) are homeless on any given night (9) and more than 105,000 persons in Australia were counted as homeless on census night in 2011 (10). Little is known about the extent of homelessness in most developing countries due to little or no reliable data (11).

In this review we have included both individuals who are homeless (living on the streets, in shelter or temporary housing), and those who have been identified as at‐risk of becoming homeless (individuals with mental illness, chronic physical illness, substance abuse, recently released criminal offenders).

### Description of the intervention

A serious problem, affecting any effort to synthesize research on housing programs and case management for homelessness, is a lack of consistency in the use of program labels (12). Below is a short description of the groups of interventions included in this review.

#### Case management

Case management (CM) is a “collaborative process of assessment, planning, facilitation and advocacy for options and services to meet an individual's health and social needs through communication and available resources” (13). In an early review of case management, Morse (1998) summarized the research on why case management has been widely implemented with homeless individuals (14): people who are homeless have multiple serious problems and their service needs are often unmet (15, 16), and these services, and the necessary resources, are difficult to access (17). Furthermore, patients with a mental illness may refuse help and/or miss appointments and/or show aggressive or antisocial behaviour which leads to exclusion from care in many instances (16). Case managers are intended to help guide the individual through the system and facilitate their access to resources and services.

Morse (14)suggested that case management can be described in terms of seven process variables that impact on the intensity of care provided:

1. Duration of services (varying from brief or time limited to ongoing and open‐ended)
2. Intensity of services (involving frequency of client contact, and client‐staff ratios)
3. Focus of services (from narrow and targeted to comprehensive)
4. Resource responsibility (from system gatekeeper responsible for limiting service utilization to client advocate responsible for increasing access or utilization of services)
5. Availability (from scheduled office hours to 24‐hour availability)
6. Location of services (from all services delivered in office to all delivered in vivo)
7. Staffing ratios and composition (from individual caseloads to interdisciplinary teams with shared caseloads)

Case management interventions can be categorized into the following five models: broker case management (BCM), standard case management (SCM), intensive case management (ICM), assertive community treatment (ACT), and critical time intervention (CTI). See Table 3.1 in Appendix 3 for an adapted overview of case management models (14, 18).

In this review, we have organized case management according to intensity: high versus low. The following is a description of the interventions included under high intensity case management:

Assertive Community Treatment (ACT) is an example of intensive case management in which a high level of care is provided. The distinguishing features of ACT are described as follows:

“case management provided by a multidisciplinary team of professionals, including psychiatrists, social workers, nurses, occupational therapists, vocational specialists, etc.; 24‐hour, 7 days a week coverage; assertive outreach; and providing support to clients in the community where they live rather than office‐based practice” (19).

Intensive case management (ICM) is similar to ACT. However, the primary difference (McHugo et al., 2004; Meyer and Morrissey, 2007) is that while ACT involves a shared caseload approach, ICM case managers are responsible for their individual caseloads. Furthermore, each staff member of an ACT team provides direct services, while this is not the case when ICM is applied. Finally, ICM usually lacks a validated model including a manual for treatment fidelity. We will use the term *intensive case management* when referring to both categories (ICM and ACT). When it is necessary to separate the two alternatives, this is explicitly emphasized in the text.

Intensive case management (ICM and ACT) is intended to make sure that the client receives sufficient service, support and treatment when and where it is needed. In this way intensive case management (one case manager per 15 or fewer clients, available 24‐7, and the combined competence of a multidisciplinary team), may help homeless people to obtain accommodation, and once housed avoid eviction.

Low intensity case management refers to all other types of case management where 1) the case manager has responsibility for more than approximately 15 clients, is less available, and where meetings are scheduled less frequently than, for example, once per week, 2) the intervention is described as standard or broker case management, or 3) where intensity was not described.

#### Housing programs

Housing programs for homeless people typically provide accommodation and include goals such as long term residential stability, improved life‐skills and greater self‐determination (20, 21). These programs are complex and may include various forms of support and services, such as case management, work therapy, treatment of mental illness and substance abuse (22).

The objective, to find accommodation and avoid eviction, is assumed to be facilitated by combining case management with housing programs. The housing programs are more or less based on housing philosophies. The philosophy may determine the sequence of how specific program elements are introduced and removed. The intended endpoint is usually the same, i.e., independent living with as high degree of normality as possible, e.g., apartments owned or rented by the client, integrated among apartments for ordinary tenants, where housing is neither contingent on sobriety nor on treatment compliance, and with no on‐site staff (23).

##### Non‐abstinence‐contingent housing programs

According to one philosophy, stable and independent housing is needed for the client to become treatment ready(24). Housing should neither be contingent on sobriety nor on treatment compliance, but only on rules that apply for ordinary tenants(24). These housing programs aim to provide a safe and predictable living arrangementin order to make the clients treatment ready. The client's freedom to choose is crucial for treatment to be successful(25). Therefore, housing programs are neither contingent on treatment compliance nor on sobriety. In other words, housing is parallel to and not integrated with treatment, or with other services. This type of treatment is also sometimes referred to as *Parallel housing*, or *Housing First*.

“Housing First” is a specific model of non‐abstinence‐contingent housing developed by Pathways to Housing. The program is founded on the idea that housing is a basic right. The two core foundations of the program include psychiatric rehabilitation and consumer choice. Individuals are encouraged to define their own needs and goals. Housing is provided immediately by the program if the individual wishes, and there are no contingencies related to treatment or sobriety. The individual is also offered treatment, in the form of an adapted version of Assertive Community treatment (addition of a nurse practitioner to address physical health problems, and a housing specialist)(24).

##### Abstinence‐contingent housing programs

An alternative philosophy assumes that clients need a transitional period of sobriety and treatment compliance, before they can live independently in their own apartments. Without the transitional phase they will soon become evicted, and return to homelessness. In other words, this phase may be necessary for many clients to become housing ready. According to this philosophy housing is integrated with treatment. This approach has been referred to as treatment first, continuum of care, and or linear approach(22, 26).

##### Housing vouchers

Housing vouchers are financial support (usually) from the government where the individual can choose any free market rental property they wish, with no conditions based on tenancy other than financial contribution of 30% of their income(27).

#### Housing programs and case management

Housing programs and case management tend to appear in various combinations. Evaluations are typically based on comparison of one type of combination with another, or with “usual care” (often drop in centres, after care services, outpatient clinics, brokered case management, etc.). This means that housing programs are often not implemented and evaluated in similar forms. Any effort to analyse and synthesize evaluations of housings programs, case management and other included services, must therefore consider this complexity and lack of clarity. In addition to this complexity, the population of homeless people consists of subgroups that may respond differently to alternative interventions: mentally ill, substance abusers, veterans, women, etc., and each of these subgroups can be divided further.

In order to make the intervention complexity more comprehensible, two dimensions are outlined: (1) case management care intensity, and (2) contingency of tenancy in housing programs. On the one end of the case management scale there are teams with caseloads of maximum 15 clients per case manager, and full on‐site availability (24 hours, 7 days a week) for services and support. In the middle there is CM with caseloads with between 15 to 40 clients per case manager, and service and support only available duringoffice hours at the office. At the other end of the scale there are no case managers, and clients have to rely on drop‐in centres, outpatient clinics, after care services, charities, etc. With respect to contingency in housing programs, there appears to be a dichotomy where programs either require that individuals adhere to agreed‐upon treatment or sobriety obligations in order to remain in housing (abstinence‐contingent) or no conditionality is placed on tenancy, other than in some cases of financial contributions (non‐abstinence‐contingent).

### How the interventions may work

There are two objectives of the interventions: first to get accommodation, and then to avoid eviction. Housing programs provide accommodation to individuals. Case management (low or high intensity) is intended to compensate for the clients’ lack of resources and to help them either obtain accommodation, and/or after they have become housed, avoid eviction. It is a collaborative process, including assessment, planning, facilitation and advocacy for options and services.

### Why it is important to do this review

Efforts to combat homelessness have been made on national levels as well as at local government level, including specific treatments for particular types of clients. In addition, there have been many evaluations of housing and treatment programs for homeless individuals and/or persons at risk of homelessness. Several reviews and meta‐analyses have also been published (12, 18, 20, 28‐31). Yet, a large share of the reviews are out of date, or do not focus on homelessness and residential stability as primary outcomes, or are not systematic reviews of effectiveness.

Tabol and colleagues (2010) (12) aimed to determine how clearly the supported/supportive housing model is described and the extent to which it is implemented correctly (treatment fidelity). Another recent systematic review by de Vet and colleagues focussed on case management for homeless persons. They identified 21 randomized controlled trials or quasi‐experimental studies, but did not conduct a meta‐analysis, or GRADE the certainty of the evidence. A review by Chilvers and colleagues published in 2006 looked specifically at supported housing for adults with serious mental illness, but did not identify any relevant studies(32).

This review differs from previous attempts at reviewing the evidence in that we have only included randomized controlled trials that examine a broad range of interventions with follow‐up of at least one year. Furthermore, we have pooled the results where possible which has allowed us to look at the evidence across studies and not conclude based on small sample sizes from individual studies. Finally, we have applied GRADE to the outcomes, thus providing a more concrete indication of our certainty in the evidence.

## Objectives

The primary objective was to assess the effectiveness of various interventions combining housing and case management as a means to reduce homelessness and increase residential stability for individuals who are homeless, or at risk of becoming homeless. Interventions include:

- Abstinence‐contingent housing, non‐abstinence contingent housing, housing vouchers and residential treatment
- High intensity case management (intensive case management and assertive community treatment), and low (ordinary or brokered) case management
- Housing programs combined with case management programs.

## Methods

This systematic review of the effectiveness of interventions to reduce homelessness and increase residential stability for people who are homeless was conducted in accordance with the guidelines in the NOKC Handbook for Summarizing Evidence (33) and the Cochrane Handbook for Systematic Reviews of Interventions (22).

This review was carried out in two phases. The first phase began with a literature search in 2010. The project was taken over in 2014 by the current review team and two updates to the original search were conducted in addition to a search for grey literature. We reassessed studies included by the original review team for inclusion, and excluded those with a quasi‐experimental design (see further details below). Due to problems with archiving, there is no documentation of reasons for exclusion for some of the studies excluded in the first phase of the project.

A protocol was approved and published by the review team in the Campbell Library in 2010. The protocol was used as the basis for the development of a protocol by the current review team which was approved and published on the NOKC website in 2014(34). The updated searches (2014 and 2016) were based on the search specified in the Campbell approved protocol, and the inclusion criteria aresimilar, aside from study design. There are four main differences between the protocol published in Campbell Library and the protocol for the current review: Firstly, in this review protocol we only included RCTs. This decision was based on the number of RCTs identified, which seemed sufficient even after the original search. Secondly, we did not include data or analyses related to cost effectiveness as these outcomes were not prioritized by our commissioners. Thirdly, we did not exclude studies if they did not sufficiently report the results. The results from these studies were reported narratively. Finally, we applied the GRADE approach to all primary outcomes.

### Literature search

We systematically searched for literature in the following databases. Unless otherwise noted, the databases were searched in 2016, 2014, and 2010. Any databases that were not searched in 2016 and 2014 is due to lack of access. There were no limitations on the search with respect to date of publication (i.e. the databases were searched for their entirety since indexing began).

- PsycINFO
- ASSIA (2014, 2010)
- Campbell Library (2016)
- Cochrane Library (including CENTRAL)
- PsychInfo (2016, 2014)
- PubMed
- Social Services Abstracts
- Sociological Abstracts
- ERIC (2016, 2014)
- CINAHL
- ISI Web of Science (2016, 2014)

In addition, we conducted a search for grey literature through Google and Google Scholar and reference lists of identified and included studies using terms related to homelessness and housing. This search for grey literature was conducted in English, Norwegian, Swedish and Danish.

A research librarian planned and executed all the searches. The complete search strategy is published as an appendix to this report (Appendix 2). The search was last updated in January 2016.

### Inclusion criteria

**Table:** 

**Study design:**	Randomized controlled trials
**Population:**	People who are homeless or at risk of becoming homeless. A homeless person is defined as a person living in the streets without a shelter that could be classified as “living quarters”with no place of usual residence and who moves frequently between various types of accommodation (including dwellings, shelters, institutions for the homeless or other living quarters) which may include living in private dwellings but reporting “no usual/permanent address” on their census form. A person at risk of becoming homeless is someone who will be released from a prison, an institution (e.g. for psychiatric or rehabilitative care), or another accommodation within two months, and does not have any housing arranged for them in the near future (35). A person at risk can also be a person who lives temporarily with relatives or friends, or a person with short‐term subletting contracts who has applied to social services or another organization for assistance in solving their housing situation. There were no population restrictions regarding mental illness, addiction problems, age, gender, ethnicity, race, national contexts, etc. However, distinct subgroups were separated in our analyses when there was sufficient information in included studies.
**Intervention:**	Housing programs or case management or a combination of the two types of interventions. Qualified housing programs and forms of case management must meet the criteria defined by the Society for Prevention Research (36). To meet this standard, a detailed description of the program or policy must be available (p.4): “An adequate description of a program or policy includes a clear statement of the population for which it is intended; the theoretical basis or a logic model describing the expected causal mechanisms by which the intervention should work; and a detailed description of its content and organization, its duration, the amount of training required, intervention procedures, etc. The level of detail needs to be sufficient so that others would be able to replicate the programme or policy. With regard to policy interventions, the description must include information on relevant variations in policy definition and related mechanisms for implementation and enforcement.”
**Comparison:**	Any other intervention or treatment/services as usual.
**Outcome**:	Primary outcomes: homelessness and residential stability. The minimum follow up is 12 months after intake. Continuous data should describe the housing situation during specific periods, for instance, the past 30, 60, or 90 nights. This could be the mean number of nights, or the mean proportion of nights in a particular housing situation. Dichotomous data should involve the number of persons or the proportion of persons in different housing situations. Housing situations should be at least one of the following: homeless, unstable housing, or stable housing. Our goal is to use standardized definitions. Whether this is possible or not depends on the information given in included primary studies. For an outcome to be included in the meta‐analysis, necessary statistical information for calculating effect sizes or relative risks must be available. If such information is not available in identified documents or provided by authors when contacted, these outcomes and studies will be included in a narrative summary only. Secondary outcomes: (only included if primary outcomes are available) health‐related outcomes including presence/severity of mental illness or substance abuse, quality of life, marginalization, employment, criminal behaviour, school attendance.
**Language**:	No restrictions regarding language.

### Exclusion criteria

**Table:** 

**Study design:**	Other study designs, including quasi‐experimental studies with propensity score matching.
**Outcome**:	Outcomes only related to admission to hospital/psychiatric treatment, or cost‐related outcomes. However, studies were included if they also included primary outcomes.

We originally included quasi‐experimental designs for consideration when they met the other study criteria and used propensity score matching at baseline. However, given the number of randomized controlled trials identified in the updated literature search, we decided to limit inclusion to randomized controlled trials only. We thus excluded eleven studies from the final review. Given the inherent methodological limitations of quasi‐experimental designs in answering effectiveness questions, we do not believe that this decision influenced the final results of this review.

### Article selection

Two reviewers independently read and assessed references (titles and abstracts) for inclusion according to pre‐defined inclusion criteria (see above). When at least one review author considered the reference potentially relevant, the reference was ordered to be read in full‐text. Two reviewers independently read and assessed each article in full‐text for inclusion according to a pre‐defined inclusion form. Where differences in opinion emerged, the reviewers discussed until consensus was achieved. A third reviewer was brought in in instances where agreement was not possible, to assist in the decision.

### Critical appraisal

The included studies were assessed for methodological limitations using the Cochrane Risk of Bias (RoB) tool (37). Studies were assessed as having low, unclear or high risk of bias related to: (1) randomization sequencing, (2) allocation concealment, (3) blinding of personnel and participants, (4) blinding of assessors for subjective outcomes and (5) objective outcomes, (6) incomplete outcome data, (7) selective reporting and (8) any other potential risks of bias. One reviewer assessed each study and a second reviewer checked each assessment and made comments where there were disagreements. Results of the Risk of Bias assessments were discussed until consensus was reached.

### Data extraction

One reviewer systematically extracted data from the included studies using a pre‐designed data recording form. A second reviewer then checked the data extraction for all included studies. Any differences or comments were discussed until consensus was achieved.

The following core data were extracted from all included studies:

- Title, authors, and other publication details
- Study design and aim
- Setting (place and time of recruitment/data collection)
- Sample population characteristics (age, gender, ethnicity, mental health/substance use status, homelessness status, criminal activity)
- Intervention characteristics (degree and type of housing support and degree/type of service support and/or therapy offered)
- Methods of outcome measurement (clinical, self‐report, physical specimens for substance use outcomes)
- Primary outcomes related to number of days spent in stable housing or homeless
- Secondary outcomes related to housing (satisfaction with housing, type of housing, etc.), addiction status, mental or physical health, criminal activity, and/or quality of life.

Many of the studies were reported in more than one publication. One publication was identified as the main publication (usually the one with results related to the primary outcomes), and we only extracted data from publications in addition to the identified main publication when they added more information regarding the methods or results on relevant outcomes. We excluded studies if they reanalysed already included data using different techniques.

Given the complexity of the interventions being investigated, we attempted to categorize the included interventions along four dimensions: (1) was housing provided to the participants as part of the intervention; (2) to what degree was the tenants’ residence in the provided housing dependent on, for example, sobriety, treatment attendance, etc.; (3) if housing was provided, was it segregated from the larger community, or scattered around the city; and (4) if case management services were provided as part of the intervention, to what degree of intensity. We created categories of interventions based on the above dimensions:

1. Case management only
2. Abstinence‐contingent housing
3. Non‐abstinence‐contingent housing
4. Housing vouchers
5. Residential treatment with case management

Some of the interventions had multiple components (e.g. abstinence‐contingent housing with case management). These interventions were categorized according to the main component (the component that the primary authors emphasized). They were also placedin separate analyses. We then organized the studies according to which comparison intervention was used (any of the above interventions, or usual services).

For each comparison, we evaluated the characteristics of the population. In those cases where they were considered sufficiently similar (specifically with respect to individuals versus families, mental illness, substance abuse problems, literally homeless versus at risk of homelessness), and had comparable outcomes, the results from the studies were pooled in a meta‐analysis when possible. In those cases where the populations of studies with the same comparisons were considered too different to analyse together we have not pooled the results.

We extracted dichotomous and continuous data for all outcomes where available. We also extracted raw data and, when such data were available, adjusted outcome data (adjusted comparison (effect) estimates and their standard errors or confidence intervals). When information related to outcome measurement (e.g. sample sizes, exact numbers where graphs were only published in the article) were missing in the publication, we contacted the corresponding author(s) via e‐mail and requested the data.

### Data synthesis

Results for the primary outcomes (number of days spent in stable housing or homeless) are presented for each comparison along with a GRADE assessment. Results for secondary outcomes (for longest follow‐up time) for each comparison were not synthesized, but are presented in Appendix 4. For comparisons where more than two studies are included, we present the primary outcomes with the longest follow‐up time. Results for secondary outcomes are described in Appendix 4.

We summarized and presented data narratively in the text and table for each comparison. We also conducted a meta‐analysis with random effects model and presented the effect estimate, relative risk and the corresponding 95% confidence interval (CI) using risk ratio for dichotomous outcomes. For continuous outcomes we analysed the data using (standardized) mean difference ((S)MD) with the corresponding 95% CI. We used SMD when length of time was measured different between pooled studies (e.g. in days versus months, etc.). We conducted meta‐analyses using RevMan 5,using a random‐effects model and inverse‐variance approach(38). This method allowed us to weight each study according to the degree of variation in the confidence in the effect estimate.

In cases where the means, number of participants and test statistics for t‐test were reported, but not the standard deviations, and there was the opportunity to include results in a meta‐analysis, we calculated standard deviations, assuming same standard deviation for each of the two groups (intervention and control).

#### Heterogeneity

We assessed statistical heterogeneity using I^2^. Where I^2^was less than 25% we considered the results to have low heterogeneity. Where I^2^ was greater than 50% we considered the results to have high heterogeneity. Where this heterogeneity could be explained, we proceeded to pool results. However, if heterogeneity could not be explained, we did not pool the results and presented the results separately for each study.

#### Subgroup analysis

We did not plan or conduct moderator or subgroup analyses.

#### Dependent effect sizes

We did not include a comparison group more than once in an analysis. Where we were interested in an intervention and it was compared to two or more comparison interventions that were both considered to be within the realm of “usual services”, we combined the two comparison arms into one comparison group and compared the means of the combined control groups to the intervention for a given outcome (39).

In one study we have combined two intervention arms that both employed slightly differing versions of an intervention (assertive community treatment) into one intervention group and compared that to the usual services comparison condition (40).

#### Primary outcomes

Outcomes related to housing and homelessness were reported using multiple measurements/scales/methods in some studies. These included number of days spent in stable housing or homeless, length of time to move from shelter to permanent housing (measured in days), number or percentage of participants who reported being homeless during a given period, or at a certain measurement point, and the change in number/proportion of days spent in various living conditions between baseline and follow‐up points.

#### Secondary outcomes

We did not synthesize or report results for secondary outcomes. They are described in Appendix 4 as they are reported in the original primary publications.

### GRADING of the evidence

We assessed the certainty of the synthesized evidence for each primary outcome using GRADE (Grading of Recommendations Assessment, Development, and Evaluation). GRADE is a method for assessing the certainty of the evidence in systematic reviews, or the strength of recommendations in guidelines. Evidence from randomized controlled trials start as high certainty evidence but may be downgraded depending on five criteria in GRADE that are used to determine the certainty of the evidence: i) methodological study quality as assessed by review authors, ii) degree of inconsistency, iii) indirectness, iv) imprecision, and v) publication bias. Upgrading of results from observational studies is possible according to GRADE if there is a large effect estimate, or a dose‐response gradient, or if all possible confounders would only diminish the observed effect and that therefore the actual effect most likely is larger than what is suggested by the data. GRADE has four levels of certainty:

**High certainty:** Further research is very unlikely to change our confidence in the estimate of effect.

**Moderate certainty:** Further research is likely to have an important impact on our confidence in the estimate of effect and may change the estimate.

**Low certainty:** Further research is very likely to have an important impact on our confidence in the estimate of effect and is likely to change the estimate.

**Very low certainty:** We are very uncertain about the estimate.

Assessments are done for each outcome and are based on evidence coming from the individual primary studies contributing to the outcome. For more information on GRADE visit www.gradeworkinggroup.org, or see Balshem and colleagues 2011 (41).

For a detailed description of the Norwegian Knowledge Centre's procedures, see the Norwegian Knowledge Centre's Handbook(33).

## Results

**Figure 1:**
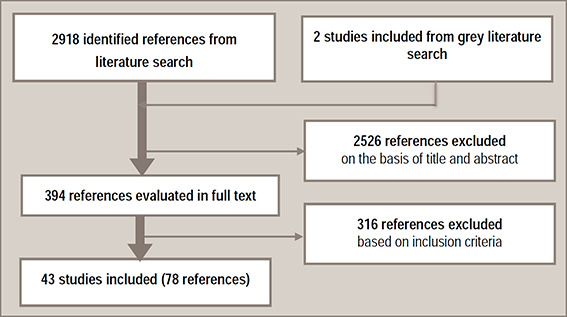
Flowchart of the literature selection process

The search was conducted in three stages. The original systematic search of databases in 2010 resulted in 1,764 unique references (Figure 1). We identified a further831 unique references from the update search in2014, and 323 more in the January 2016 update search. Altogether we identified 2,918potentially relevant references through database searches. In addition, a grey literature search identifiedan additional 2 relevant studies (and 11 references). We excluded 2,526references based on title and abstract. We read 394 references in full and excluded 316 based on the predefined inclusion and exclusion criteria. In total, we critically appraised 43 studies that were described in 78 publications. A list of excluded studies with reasons for exclusion is included in Appendix 5. Problems related to archiving from the first search in 2010 resulted in missing the references and the reasons for exclusion for 50 excluded studies.

### Description of the included studies

We identified 43 randomized controlled studies (RCTs) reported in 78 publications (24, 26, 27, 39, 40, 42‐81)that met our inclusion criteria, and two studies in progress (31, 82). See Appendix 9 for a description of the studies in progress.

Thirteen of the included studies were published in or after 2010, thirteen were published between 2000 and 2009, and seventeen studies were published before 2000.

The majority of the studies were conducted in the United States (n=37), and other included studies came from other high‐income countries, including United Kingdom (n=3), Australia (n=1), Canada (n=1), and Denmark (n=1). Eleven of the studies were conducted at multiple sites (cities/institutions).

The duration of the intervention was not reported in all of the included studies. It appears that in most of these cases the intervention was available/offered until the longest follow‐up. There were also some discrepancies between the number of participants randomized and the number of participants included in analyses in some cases. We have highlighted where we think this is a concern.

From these 43 RCTs we have summarized findings from 28 comparisons in five categories of interventions (see Table 1).

**Table 1:** Overview of comparisons of case management interventions

**Category**	**Intervention**	**Comparisons**
1. Case management	1. A. High intensity case management	1. A.1. Usual services
		1.A.2. Low intensity case management
		1.A.3. Other intervention (no case management or housing program)
	1.A. High intensity case management (with consumer case management)	1.A.4. High intensity case management (without consumer case management)
	1.B. Low intensity case management	1.B.1. Usual services
		1.B.2. Low intensity case management
		1.B.3. Other intervention (no case management or housing program)
	1.C. Critical time intervention	1.C.1. Usual services
Abstinence‐contingent housing programs	2.A. Abstinence‐contingent housing with case management	2.A.1. Usual services
		2.A.2. Case management
	2.B. Abstinence‐contingent housing with day treatment	2.B.1. Usual services
		2.B.2. Day treatment
		2.B.3. Non‐abstinence‐contingent housing with day treatment
		2.B.4. Abstinence‐contingent housing with community reinforcement approach
3. Non‐abstinence contingent housing programs	3.A. Housing First	3.A.1. Usual services
		3.A.2. Abstinence‐contingent housing
	3.B. Non‐abstinence‐contingent housing with high intensity case management	3.B.1. Usual services
	3.B. Non‐abstinence‐contingent group living arrangements with high intensity case management	3.B.2. Non‐abstinence‐contingent independent apartments with high intensity case management
	3.B. Non‐abstinence‐contingent housing with high intensity case management	3.B.3. Abstinence‐contingent housing with high intensity case management
	3.B. Non‐abstinence‐contingent housing with day treatment	3.B.4. Day treatment
4. Housing vouchers with case management	4. Housing vouchers with case management	4.1. Usual services
		4.2. Case management
5. Residential treatment	5. Residential treatment	5.1. Usual services

### Risk of bias in the included studies

The majority of the RCTs were assessed as having high risk of bias. In many instances this was due to inadequate reporting of methods in general (unclear risk of bias). In particular, most studies were at unclear or high risk of selection bias because they either did not report randomization or allocation concealment procedures or reported inadequate methods of randomization or allocation concealment. The vast majority of studies were assessed as having unclear or high risk of performance bias: Blinding of participants and personnel was either not described in many studies (unclear risk), or not possible and reported as such (high risk). In the majority of studies outcome assessors were not blinded (high risk), or blinding was not mentioned (unclear risk). The risk of bias was separated into blinding of outcome assessment for subjective and objective outcomes due to the poor reporting, or lack, of blinding. The intention behind this was to indier4’;cate that the blinding might have an impact on subjective outcomes, but not objective outcomes such as death or number of days housed when the data came from administrative records. Some studies also were assessed as being at high risk for attrition bias because they used inappropriate methods for dealing with missing data, or reporting bias because the results were not reported for all outcomes. It is not clear how much attrition has occurred in many of the primary studies, and in some cases the level of attrition differs between results within the same study but is not discussed by the primary authors. See Appendix 6 for a more detailed explanation of the risk of bias assessment for each study.

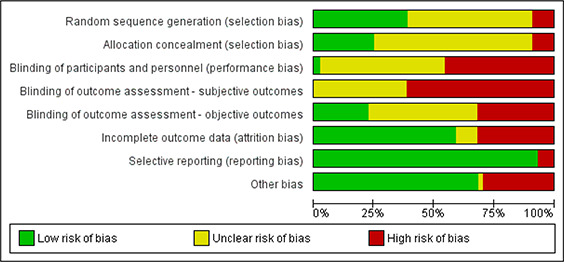

### Interventions and comparisons

We included and extracted data from 43RCTs(this information was presented in 78 publications). Some studies included multiple comparisons (multiple interventions), and some publications reported results from multiple studies (for example information related to two studies in one publication). Details on all of the included comparisons are described below. Details regarding data related to secondary outcomes is not reported in the main text of this report but can be found in Appendix 4.

The case management component in the included studies varied in terms of approach, intensity and case‐load for case managers. We have therefore categorized case management components as either low intensity (case management with no further details, brokered case management), high intensity (Assertive Community Treatment or Intensive Case Management), or Critical Time Intervention (intensive case management for a shorter defined period of time). In addition, some interventions included a housing component and a treatment component that could not be described as case management (e.g. day treatment or Community Reinforcement Approach). Interventions including these treatment components have been analysed separately from interventions that include low or high intensity case management components. Most of the interventions evaluated in the included comparisons were complex in that they were made up of multiple components, and there was a large degree of flexibility in terms of how the interventions were implemented (including varying levels of treatment fidelity). Furthermore, many of the studies reported that the interventions and control conditions changed and evolved during the course of the studies in terms of organization, and availability of resources and services. More details on the interventions evaluated in each study is reported under the relevant comparison.

The comparison groups varied considerably, and in many cases it is difficult to ascertain what kind of interventions participants in these groups received/were offered due to poor reporting. The comparison groups were described as usual services (care as usual), other types of housing programs or case management interventions, or other types of interventions. All of the comparison groups, however, received some type of active intervention. That is, even participants in the usual services groups had access to drop in centres, and to some degree case management and/or shelter.

### Population in the included studies

A total of approximately 10,570 participants were included in the identified studies. This is an approximate number due to poor reporting in many of the studies. The majority of the studies included adults who had a mental illness or substance dependence and were homeless or at‐risk of becoming homeless due to the previous mentioned illnesses. More detail on the populations in the included studies is available under each comparison.

### Description of outcomes reported in the included studies

All of the included studies reported at least one outcome related to homelessness or housing stability. This was reported in various ways including the number of days participants reported being housed/homeless, proportion of participants homeless or housed at follow‐up, time to exit from/return to shelter, and frequency of address change. Many of the included studies also included outcomes related to employment, mental or physical health, quality of life, social support and criminal activity. Details regarding outcomes are described under each comparison.

Secondary outcomes for each comparison are presented in Appendix 8.

### Category 1: Case management

#### Description of included studies

We identified 26 studies with four comparisons that evaluated the effect of case management on housing stability and/or homelessness (26, 39, 40, 44‐48, 50, 52‐54, 56, 59, 60, 64, 69‐72, 74, 76, 77, 79, 80, 83). The majority of the studies were conducted in the USA (N=22), with the remaining studies from either Australia (N=1), Denmark (N=1) or the United Kingdom (N=3). Data for the included studies were collected between the 1980s (earliest published study from 1990, but it is unclear when data was collected) and 2009, and thus represent varying populations and settings in terms of political and social climate in the various countries and states where the studies are conducted. The exact number of participants is not always clearly reported. We have reported the total number randomized and included in analyses where possible.

Within the category of case management, we identified four subcategories of interventions which were compared to usual services or other interventions. See Table 2 for an overview.

**Table 2:** Overview of case management comparisons

**Intervention**	**Comparisons**
1.A. High intensity case management	1.A.1. Usual services
	1.A.2. High intensity case management (without consumer case management)
	1.A.3. Low intensity case management
1.A. High intensity case management(with consumer case management)	1.A.4. Other intervention (no case management or housing program)
1.B. Low intensity case management	1.B.1. Usual services
	1.B.2. Low intensity case management
	1.B.3. Other intervention (no case management or housing program)
1.C. Critical time intervention	1.C.1. Usual services

Table 3 presents an overview of the populations, interventions, comparisons and outcomes in the included studies. The total number of participants indicates the number of participants randomized. The number of participants for each group does not always add up to the total number of participants because most studies reported the number included in analyses, but not always the number randomized. Participants in the included studies were adults (>18 years old) unless otherwise specified. We report the longest outcome assessment for each study (shorter follow‐up assessments were also done in some studies).

**Table 3:** Description of studies that evaluated effects of case management interventions (N=26)

**Study (ref); country**	**Population** **(N, description)**	**Intervention** **Follow‐up (FU) in months (mos), N**	**Comparison** **N**	**Primary outcome**
HIGH INTENSITY CASE MANAGEMENT (N=18)				
Bell 2015 (44), USA	N=1380, disabled Medicaid beneficiaries with mental health and/or substance abuse problems and comorbid physical conditions	Intensive care management FU: 24 mos N=690	Usual services (wait‐list) N=690	Mean number of homeless months per 1000 months Proportion of participants with any homeless months
Bond 1990 (45)(45), USA	N=88, serious mental illness, multiple hospitalizations	Assertive community treatment FU: 12 mos N=45	Drop‐in centre N=43	Housing stability Living arrangements
Grace 2014 (46), multisite, Australia	N=396 18‐35, (previously) homeless, receiving financial aid	Intensive case management FU: 18‐30 mos N=222	Usual services N=174	Number of moves Housing status Number of homelessness events
Clarke 2000 (48), USA	N=178, chronically mentally ill	Assertive community treatment FU: 24 mos N=114	Usual care N=49	Time to first instance of homelessness
Cox 1998 (50), USA	N=298 homeless, substance dependence	Intensive case management FU: 18 mos N=150	Usual care N=148	Nights in own residence, nights homeless
Drake 1998 (52), multisite, USA	N=224, 18‐60, mental illness, substance abuse disorder, no additional medical conditions	Integrated Assertive community treatment FU: 36 mos N=105	Standard case management N=98	Days in stable housing
Essock 2006 (53), multisite, USA	N=198 severe mental illness	Integrated Assertive community treatment FU: 36 mos N=99	Standard case management N=99	Days in stable housing
Garety 2006 (54), UK	N=144 mental illness	Assertive Community treatment FU: N=71	Usual services N=73	Not stably housed
Killaspy 2006 (59), multisite, UK	N=251 severe mental illness	Assertive community treatment FU: 18 mos N=127	Usual services N=124	Not homeless
Lehman 1997 (60), USA	N=126 severe mental illness	Assertive community treatment with housing opportunities FU: 12 mos N=77	Usual services N=75	Days in community housing Days homeless
Morse 1992 (39), USA	N=178 (103 analyzed), homeless adults with mental illness	Assertive community treatment FU: 12 mos N=52	Drop in centres N=62 or outpatient services N=64	Days not homeless Days homeless
Morse 1997 (40), USA	N=165 (85 analyzed), homeless, mental illness	Assertive community treatment with/out community workers FU: 18 mos N=35/28	Brokered case management N=22	Days in different housing settings Days not stably housed
Morse 2006 (69), USA	N=196 (149 analyzed), homeless, mental illness, substance dependence	Assertive community treatment with/out integrated treatment FU: 24 mos N=46/54	Usual services N=49	Days in stable housing
Nordentoft 2010 (70), multisite, Denmark	N=275 mental illness	Assertive community treatment FU: 5 years N=275	Usual services N=272	Days homeless
Rosenheck 2003 (71), multisite, USA	N=278 homeless veterans, mental illness and/or substance abuse	Intensive case management only FU: 36 mos N=90	Usual services N=188	Stably housed Homeless
Solomon 1995 (76), USA	N=96 (90 analyzed) major mental illness	Consumer case management FU: 24 mos N=48	Non‐consumer case management N=48	Homelessness
Toro 1997 (80), USA	N=202 homeless families	Intensive case management, employment training and housing FU: 18 mos N=101	Usual services N=101	Days homeless
Nyamathi 2015 (83), USA	N=600 men recently released from prison/jail	Intensive case management with peer coaching FU: 12 mos N=166	Usual services N=186 Peer coaching N=177	Homelessness
LOW INTENSITY CASE MANAGEMENT (N=5)				
Chapleau 2012 (47), USA	N=57 at risk or homeless, severe mental illness	Case management with Occupational therapist FU: 12 mos N=29	Case management N=28	Housing status
Marshall 1995 (64), USA	N=80 mental illness	Case management FU: 14 mos N=40	Usual services N=40	Days in better/worse accomodation
Slesnick 2015 (74), USA	N=270 homeless youth, substance abuse problems	Case management Duration: 12 mos N=91	Community reinforcement approach N=93 Motivation Enhancement therapy N=86	Homelessness
Sorensen 2003 (77), USA	N=190 substance abusers, HIV/AIDS	Case management Duration: 12 mos FU: 18 mos N=92	Brief contact N=98	Homelessness
Sosin 1995 (26), USA	N=191 analyzed, homeless, substance dependence	Abstinence‐contingent housing with case management Duration: average 6 mos FU: 12 mos N=70	Usual care N=121	Number of days housed of previous 60 days
**CRITICAL TIME INTERVENTION (N=3)**				
Herman 2011 (56), USA	N=150 recently discharged, psychotic disorder	Critical Time Intervention with post‐discharge housing FU: 18 mos N=77	Usual services with post‐discharge housing N=73	Days homeless Homeless at baseline
Samuels 2016 (72), USA	N=223 (210 analyzed) homeless mothers, mental illness	Critical time intervention with scattered site housing Duration: 9 mos FU: 15 mos N=97	Usual services N=113	Number of days to move out of shelter Proportion of days homeless
Susser 1997 (79), USA	N=96, homeless adult men, severe mental illness	Critical Time Intervention with supportive housing Duration: 18 mos N=48	Usual services N=48	Days homeless

#### Description of the intervention

The case management intervention in the included studies varied considerably in terms of intensity, organization and length. The interventions are described in more detail under the relevant comparison and in Appendix 7.

#### Category 1.A: High intensity case management

We identified 18 studies that evaluated the effect of high intensity case management on housing stability and/or homelessness (39, 40, 44‐46, 48, 50, 52‐54, 59, 60, 69‐71, 76, 80, 83). High intensity case management included interventions which were described as using either Assertive Community Treatment (ACT; N=12) or intensive case management (ICM; N=6). The included interventions varied in terms of ratio of clients per case manager, frequency of contact, length of treatment and follow‐up, location of appointments, degree of service provision versus referral, and team versus individual approach to case management.

The interventions in the majority of the included studies (N=13) are compared to usual services (44‐46, 48, 50, 54, 59, 60, 69‐71, 80, 83). One study compared the intervention to another type of high intensity case management (76) and two studies compared it to low intensity case management (53, 69). In two of the included studies, multiple intervention arms or comparison arms were relevant for this category of interventions (39, 40). In one study we have combined two intervention arms that both employed slightly differing versions of assertive community treatment into one intervention group compared to usual services (40). In the other study (39), we combined two comparison arms that both offered usual services to participants into one comparison group compared to the intervention.

Services provided as part of “usual services” varied greatly between and within the studies. We have chosen to include all studies that compared high intensity case management to “usual services” in one comparison. The term “usual services” covers a wide variety of services, but generally refers to the variety of services available to any person meeting the eligibility criteria of the study and not an alternative intervention which participants who are not randomized to the intervention group receive. Usual services in the included studies included drop‐in centres, provision of a list of services and information (69), case management style services (59)and limited peer coaching(83). Control conditions were too poorly described in most studies to accurately document what participants had access to.

##### 1.A.1. High intensity case management compared to usual services

We identified 18 studies (39, 40, 44‐46, 48, 50, 52‐54, 59, 60, 69‐71, 76, 80, 83) which evaluated the effect of high intensity case management compared to usual services on housing stability and homelessness in the USA (N=15), United Kingdom (N=2) and Denmark (N=1). The included studies were conducted over a long span of time; however, the majority of studies were conducted or began before the end of 2000 (N=12).

Fifteen of the included studies focused on adults with mental illness and/or substance abuse issues (39, 40, 44, 45, 48, 50, 52‐54, 59, 60, 69‐71, 76). One study focused on disadvantaged youth (46), one study included adults with families (80), and one study targeted recently released criminal offenders (83). While the studies differed slightly in the populations targeted, all of the studies included participants with mental illness and/or substance abuse even when that was not the main identifying characteristic of the target population. Information regarding mental illness and substance abuse was not reported for the study on disadvantaged youth; however, there was little reason to assume that this group would react differently to the intervention. More importantly, given the outcomes analysed here, housing stability and homelessness, one can assume that this is a universally sought after outcome, and the characteristics of the population might not be considered to be important. Below is a description of the results.

###### Primary outcome: Housing stability

Six of the included studies examined housing stability for adults with mental illness and/or substance dependence issues (45, 46, 50, 54, 59, 60, 69).

**Figure 2:**
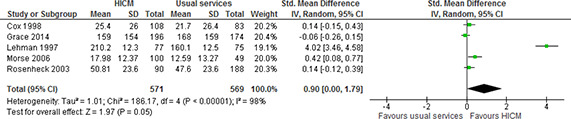
Number of days in stable housing, 12‐24 months follow‐up, high intensity case management vs usual services

We carried out a meta‐analysis for number of days in stable housing, pooling available data from four included studies (46, 50, 60, 69, 71) to examine the effect of high intensity case management compared to usual services on number of days in stable housing. As evident from the forest plot (Figure 2), the pooled analysis indicates that the high intensity case management leads to an increase in the number of days spent in stable housing compared to usual services (SMD=0.90, 95%CI=0.00 to 1.79). Although considerable heterogeneity is indicated by I^2^ and Chi^2^(I^2^=98%, chi^2^=186.17), this is expected due to the complexity of the included interventions, the geographical range of included studies (multiple cities across USA, and Australia) and the wide range of when the interventions were implemented.

**Figure 3:**
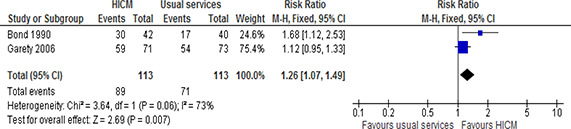
Number of participants in stable housing, 12‐18 months follow‐up, high intensity case management vs usual services

We carried out a meta‐analysis to estimate the number of participants in stable housing at 12‐18 months after the start of the intervention, pooling available data from two included studies (45, 54). As evident from the forest plot (Figure 3), the pooled analysis indicates that high intensity case management leads to a greater number of individuals living in stable housing compared to usual services (RR=1.26, 95%CI= 1.07 to1.49). While the heterogeneity was assessed as being high (I^2^=73%, chi^2^=3.64), this can be accounted for by differences in when the interventions were implemented (approximately 15 years between publications) and assessed and geographical differences (UK and USA). Together these differences may have implications for political or social contexts which may, in turn, have impacted, for example, the type of usual services being provided.

It is uncertain whether high intensity case management improves either the length of time individuals spend in their longest recorded residence, the number of clients who do not move (45), or the number of moves during the last half of a one or two year period (45).

One study reported that there was no difference between the intervention and control groups in the number of moves reported during the previous 12 months as measured at 24 months MD=0.30 (‐0.04, 0.64)(46).

###### Primary outcome: Homelessness

Thirteen of the included studies examined homelessness (39, 44‐46, 48, 50, 54, 59, 60, 70, 71, 80, 83). Seven studies reported outcomes related to length of time homeless, either in terms of number of months (44) or number of days (39, 46, 50, 60, 71, 80).

We carried out a meta‐analysis for the number of days spent homeless, pooling available (adjusted) data from six included studies (39, 46, 50, 60, 71, 80). One of the studies adjusted the results for demographic characteristics, specifically ethnicity (60). This study (60)also reported both number of days homeless in shelter and number of days homeless on streets. It was not possible to combine the data from these two outcomes (means and the standard error of the mean (SEM) were reported, but not the number of participants who reported experiencing these living arrangements), so we have chosen to include the number of days homeless in shelter in this meta‐analysis. The pooled estimate indicates that high intensity case management leads to fewer days spent homeless compared to usual services. Although there is considerable heterogeneity (I^2^=58%, chi^2^=11.77), this may be explained by a wide range of geographical settings (USA and Australia), and large differences in when the interventions were implemented and assessed (from 1990s to 2006). Together these differences may have implications for political or social contexts which may, in turn, have impacted, for example, the type of usual services being provided.

**Figure 4:**
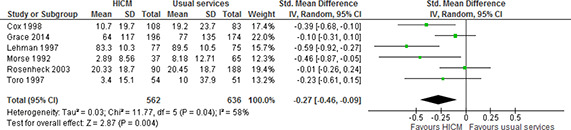
Number of days homeless, 12‐24 months, high intensity case management vs usual services

In one study (44), high intensity case management seemed to lead to fewer months homeless (mean number of months per 100 months homeless). However, the 95% confidence interval indicates that high intensity case management might make little or no difference the amount of time spent homeless (results as reported in original publication: n=‐1.5 [95%CI ‐4.3 to 1.3], p=0.29).

One study reported that participants in the high intensity case management group reported spending almost half as many days living on the street than participants in the usual services group (MD=0‐14.10 (‐15.77, ‐12.43))(60)

Three studies reported whether participants experienced homelessness during the study period (44, 48, 83). We conducted a meta‐analysis for the number of participants who experienced at least one episode of homelessness within one to two years, pooling data from two studies (48, 83). The third study was not included in the analysis due to incomplete reporting of results (baseline and follow‐up percentage of participants was not reported, only the pre‐post difference in percentage of participants who experienced homelessness during a two year period was reported along with the difference in difference (44).

**Figure 5:**
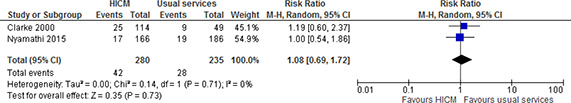
Number of participants who experienced at least one episode of homelessness, 12‐24 months, high intensity case management vs usual services

The pooled analysis, shown in Figure 5, indicates that high intensity case management may lead to little or no difference in whether individuals experience homelessness during a one to two year period compared to usual services. Results, as reported in the original publication, from the third study support this (Bell 2015 (44): OR=0.83, 95%CI=0.60 to 1.17).

**Figure 6:**
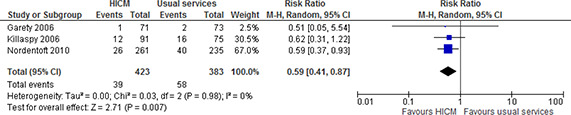
Number of participants who were homeless at last follow‐up point, 18 months, high intensity case management vs usual services

Three studies examined the number of participants who reported being homeless at the last follow‐up point (12 to 18 months after baseline) (54, 59, 70). We conducted a meta‐analysis for the number of participants who were homeless 12 to 18 months after the beginning of the study, pooling available data from three studies (54, 59, 70). One study reported the percentage of participants per group, but not the total number per group (amount of data on participants varied according to outcome), so we calculated the total number of participants per group using the information provided (70). As evident from the forest plot (Figure 6), the pooled analysis indicates that high intensity case management probably leads to fewer individuals who report being homeless at the 12 to 18 month follow‐up interview compared to usual services (RR=0.59, 95%CI=0.41 to 0.87).

The results and quality assessments for high intensity case management compared to usual services on housing stability and homelessness for adults with mental illness and/or substance abuse problems are summarized in Table 4. The complete GRADE evidence profile is shown in Appendix 8, Table 8.1.1.

**Table 4:** Summary of findings table for the effects of high intensity case management compared to usual services (Bell 2012, Bond 199, Cox 1998, Grace 2014, Garety 2006, Killaspy 2006, Nordentoft 2010, Nyamathi 2015, Toro 1997)

**Patient or population**: adults who are homeless or at‐risk of becoming homeless **Setting**: USA, **Intervention**: high intensity case management **Comparison**: usual services					
Outcomes	Anticipated absolute effects^*^ (95% CI)		Relative effect (95% CI)	№ of participants (studies)	Quality of the evidence (GRADE)
	Risk with usual services	**Risk with high intensity case management**			
Number of participants homeless at follow‐up assessed with: self‐report follow up: range 12 months to 18 months	151 per 1 000	**89 per 1 000** (62 to 132)	**RR 0.59** (0.41 to 0.87)	806 (3 RCTs)^12^	⨁⨁⨁◯ MODERATE ^5^
Number of participants living in stable community housing at follow‐up assessed with: self‐report follow up: range 12 months to 18 months	628 per 1 000	**792 per 1 000** (672 to 936)	**RR 1.26** (1.07 to 1.49)	226 (2 RCTs)	⨁⨁◯◯ LOW ^3^ ^,^ ^4^
Number of participants who experienced some homelessness assessed with: not reported follow up: 24 months	119 per 1,000	**129 per 1,000** (82 to 205)	**RR 1.08** (0.69 to 1.72)	1635 (3 RCTs)^7^	⨁⨁◯◯ LOW ^8^ ^,^ ^9^
Number of days homeless assessed with: self‐report follow up: range 12 months to 24 months	‐	SMD **0.27 SD fewer** (0.46 fewer to 0.09 fewer)	‐	1198 (6 RCTs)	⨁⨁◯◯ LOW ^6^
Mean number of days in stable housing assessed with: self‐report follow up: range 12 months to 24 months	‐	SMD **0.09 SD more** (0 to 1.79 more)	‐	1140 (5 RCTs)	⨁◯◯◯ VERY LOW ^1^ ^,^ ^2^
Number of days in longest residence during previous 6 months assessed with: not reported follow up: 12 months	The mean number of days in longest residence during previous 6 months was 160.9 days	The mean number of days in longest residence during previous 6 months in the intervention group was 16,3 days fewer (CI not reported)	‐	58 (1 RCT)	⨁◯◯◯ VERY LOW ^10^ ^,^ ^11^
Number of clients who did not move during previous 6 months assessed with: not reported follow up: 12 months	21 (62%) of HICM participants and 17 (77%) of usual services participants did not moved during this period (x2(1)=1.47, ns).			58 (1 RCT)	⨁◯◯◯ VERY LOW ^10^ ^,^ ^11^
Mean number of moves during previous 6 months assessed with: not reported follow up: 12 months	Participants in the HICM Group reported M=0.56 moves compared to M=0.29 for the usual services Group (t(53)=‐1.39, ns).			58 (1 RCT)	⨁◯◯◯ VERY LOW ^10^ ^,^ ^11^
**The risk in the intervention group** (and its 95% confidence interval) is based on the assumed risk in the comparison group and the **relative effect** of the intervention (and its 95% CI).**CI:** Confidence interval;**SMD:** Standardised mean difference;**RR:** Risk ratio;**MD:** Mean difference					

1. **Risk of performance bias in all studies**. Risk of attrition bias in three studies, risk of detection bias in two studies and risk of selection bias in one study. Inadequate reporting of randomization and/or allocation concealment methods in two studies and blinding of outcome assessors in one study.

2. Considerable heterogeneity (I2=98%, chi2=186.17).

3. Risk of performance bias.

4. Fewer than 300 participants.

5. Risk of performance bias in all studies. Risk of attrition bias in one study.

6. Risk of performance bias in four studies, risk of detection bias in two studies, risk of attrition bias in two studies and other risks of bias in two studies. Unclear reporting of selection bias in four studies and detection bias in two studies.

7. Two studies includd in the pooled analysis (N=515). One study not included in the analysis, but shows a similar result: Bell 2012 (intervention N=567, control N=563) OR=0.83, 95%CI=0.60, 1.17.

8. Inadequate reporting of randomization, allocation concealment and blinding methods in two studies.

9. Total number of events is less than 300.

10. Risk of detection bias and attrition bias. Inadequate reporting of blinding methods for participants and personnel.

11. Fewer than 400 participants.

12. Two studies included in the pooled analysis (Garety 2006 (54), Killaspy 2006). Nordentoft 2010 (N=496)showed that the intervention led to fewer homeless participants at 12 month follow‐up than the control group (OR=0.53, 95%CI=0.3, 0.9).

13. Risk of performance bias in four studies, risk of detection bias in two studies, risk of attrition bias in two studies and other risks of bias in two studies. Unclear reporting of selection bias in four studies and detection bias in two studies.

###### What does the evidence say?

High intensity case management compared to usual services:

- Probably reduces the number of individuals who are homeless after 12‐18 months (moderate certainty evidence).
- May increase the number of the number of people living in stable housing after 12‐18 months (low certainty evidence).
- May lead to little or no difference in the number of individuals who experience some homelessness during a two year period (low certainty evidence).
- May reduce the number of days an individual spends homeless (low certainty evidence).
- It is uncertain whether high intensity case management leads to a difference in the number of days an individual spends in stable housing, the number of days an individual spends in their longest residence, and the number of individuals who do or do not move (very low certainty evidence).

##### 1.A.2. High intensity case management compared to low intensity case management

We identified three studies (40, 52, 53) that examined the effects of integrated high intensity case management compared to standard case management (lower intensity) on housing stability and homelessness. The integrated treatment was based on the assertive community treatment model of case management in all three studies. Integrated treatment differs from standard case management models in that it integrates treatment for substance abuse and mental health issues into one service.

In one study (40), participants were randomized to either assertive community treatment, assertive community treatment with a community worker or brokered case management. The primary authors’ most central hypothesis was that assertive community treatment was better for clients with serious mental health issues than brokered case management. This focus fits with the aim of our review and we therefore attempted to combine results from the two assertive community treatment groups to compare them to the brokered case management group (usual services). For the purpose of this review we are interested only in the assertive community treatment condition and have thus combined the two interventions which employed the assertive community treatment model of case management. In this study the assertive community treatment model was expanded and modified: staff were instructed to visit shelters and were trained in engaging with homeless persons.

In two studies (52, 53),the high intensity case management interventions were based on the assertive community treatment model and were provided by two sites (health centres).

###### Primary outcome: Stable housing

Three studies (40, 52, 53)examined the effect of assertive community treatment compared to standard clinical case management on the number of days participants reported living in stable housing. In the first study (40),the total number of participants was not reported, and despite contacting the study authors, the information was not available. We therefore only report the results as they are reported in the study: High intensity case management led to more days spent in stable housing compared to low intensity case management (F=3.54, df=2, 129, p<0.032). The assertive community treatment group reported more days in stable housing than participants in the other two groups: at the 18 month follow‐up participants in the assertive community treatment group reported a mean of 23.70 days (SD=11.42) in stable housing during the previous month compared to 18.98 (SD=13.89) for the assertive community treatment with community workers group, and 16.02 days (SD=14.77) for the broker case management group. The authors conclude that “[t]he results provide substantial, although not complete, support for the study’ s most central prediction: assertive community treatment is a more effective intervention for people with serious mental illness who are at risk of homelessness than is broker case management” (40), p. 502).

**Figure 7:**
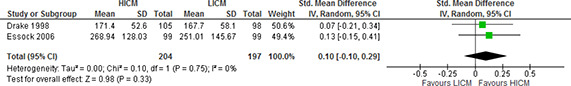
Mean number of days spent in stable housing, 36 months (high intensity case management vs low intensity case management)

We carried out a meta‐analysis for stable housing, pooling available data fromtwo studies (52, 53). The pooled analysis indicates that high intensity case management may make little or no difference to the amount of time spent in stable housing compared to low intensity case management (SMD=0.10 [95%CI ‐0.10 to 0.29], I^2^=0%) (Figure 7).

The results and quality assessments for high intensity case management compared to low intensity case management are summarized in Table 5. The complete GRADE evidence profile is shown in Appendix 8, Table 8.1.2.

**Table 5:** Summary of findings table for the effects of high intensity case management vs low intensity case management (Drake 1998, Essock 2006, Morse 1997)

**Patient or population:** individuals with mental illness and substance abuse problems **Setting:** USA **Intervention:** high intensity case management **Comparison:** low intensity case management					
Outcomes	Anticipated absolute effects^*^ (95% CI)		Relative effect (95% CI)	№ of participants (studies)	Quality of the evidence (GRADE)
	Risk with low intensity (GRADE) case management	**Risk with high intensity case management**			
Mean number of days spent in stable housing assessed with: self‐report follow up: 36 months		SMD **0.1 SD higher** (0.1 lower to 0.29 higher)^2^		458 (3 RCTs)^3^	⨁⨁◯◯
**The risk in the intervention group** (and its 95% confidence interval) is based on the assumed risk in the comparison group and the **relative effect** of the intervention (and its 95% CI).**CI:** Confidence interval;**SMD:** Standardised mean difference					

1. Risk of detection bias in one study. Inadequate reporting of methods in both studies.

2. The third study that could not be included in the pooled analysis (Morse 1997). The third study reported that HICM led to more days in stable housing (F=3.54, df=2, 129, p<0.032).

3. While only two studies are included in the analysis reported here (total population of 401 participants), the outcome is examined in three studies (total population of 458 participants).

###### What does the evidence say?

High intensity case management compared with low intensity case management for individuals with mental illness and substance abuse problems:

- Maylead to little or no difference in the number of days people spend in stable housing (low certainty evidence).

##### 1.A.3. High intensity case management compared to other intervention (no case management or housing program)

The study (83)that examined the effect of high intensity case management compared to another intervention that did not include case management or housing on housing stability and homelessness included three trial arms. The first comparison (high intensity case management compared to usual services) is included above. The high intensity case management intervention is described above, and the comparison condition consisted of peer coaching with brief nurse counselling which was identical to the peer coaching component of the intervention program, but lacked the case management component.

###### Primary outcome: Homelessness

Results from the included study (83)showed that approximately 10% of intervention group participants compared to 11% of comparison group participants reported living on the streets or in shelter during the study period (12 months) (intervention: 17/166; comparison: 20/177), and 50% of the intervention group compared to 41% of the control group reported living in someone else's house. Approximately 40% (66/166) of participants in the intervention group reported living in institutions compared to 47% (83/177) of participants in the comparison group (RR=0.91, 95%CI=0.49 to 1.67).

The results and quality assessments for high intensity case management compared to another intervention with no housing or case management component for recently released criminal offenders are summarized in Table 6. The complete GRADE evidence profile is shown in Appendix 8, Table 8.1.3.

**Table 6:** Summary of findings table for effects of high intensity case management vs other intervention (Nyamathi 2015)

**Patient or population**: recently released criminal offenders **Setting**: USA **Intervention**: high intensity case management **Comparison**: other intervention (no case management or housing component)					
Outcomes	Anticipated absolute effects^*^ (95% CI)		Relative effect (95% CI)	№ of participants (studies)	Quality of the evidence (GRADE)
	**Risk with other intervention (no case management or housing component)**	**Risk with high** **intensity case management**			
Number of participants who experience homelessness during study period assessed with: self‐report follow up: 12 months	113 per 1 000	**103 per 1 000** (55 to 189)	**RR 0.91** (0.49 to 1.67)	343 (1 RCT)	⨁◯◯◯ VERY LOW ^1^ ^,^ ^2^
*The risk in the intervention group (and its 95% confidence interval) is based on the assumed risk in the comparison group and the **relative effect** of the intervention (and its 95% CI).**CI:** Confidence interval;**RR:** Risk ratio					

1. Inadequate reporting of methods.

2. One small study. Wide confidence interval.

###### What does the evidence say?

It is uncertain whether high intensity case management reduces homelessness for recently released criminal offenders compared to another intervention (very low certainty).

##### 1.A.4. High intensity case management (with consumer case managers) compared to high intensity case management (with non‐consumer case managers)

In the study (76) that compared assertive community treatment with consumer case management to assertive community treatment with case management, the assertive community treatment model was similar in both interventions with slight differences in frequency of meetings between the teams. The main difference was that the consumer team had between none and 11 previous psychiatric hospitalizations and the non‐consumer team had no hospitalizations. There was no difference in the number of 15‐minute time units of services the first year of the program between the two teams, however consumer case managers provided more services in person to their clients and less office‐based services. Participants were recruited between 1990 and 1991.

###### Primary outcomes: Housing stability and homelessness

The results(76) show that 44 of a total of 90 participants lived in the same housing situation during the two year study period. Six participants (not specified from which group) reported being homeless at some point during the study. This study did not report any difference between the groups. There was no more data available and thus no outcomes for which we could assess certainty of the evidence (see GRADE Evidence profile in Appendix 8, Table 6.1.4).

###### What does the evidence say?

Data on housing and homelessness were not reported apart from the information given above.

#### Category 1B: Low intensity case management

We identified five studies(26, 47, 64, 74, 77) that examined the effect of low intensity case management compared to usual services (26, 64), another form of low intensity case management (47), or an intervention that included neither housing programs nor case management(74, 77). The studies were conducted in the USA or the UK and participants were recruited between 1991 and 1996 (26, 64, 77)or between 2006 and 2009 (74). Date of recruitment was not reported in one study (47).

The studies varied in terms of how the intervention was described. Studies were included in this category of interventions if the case management was included as part of the intervention, but the case management component was (a) not described as being intensive (e.g. assertive community treatment, intensive case management), or (b) was described as being or using components of brokered case management.

In the first study (47), the case management services included an occupational therapist consultant and participants were seen weekly for medication monitoring and money management. In the second study (64), the intervention was described as differing greatly according to the individual case manager in terms of time and services offered. At minimum, each participant received a needs assessment and the assessment with the person's carer (all participants were diagnosed with long‐term mental disorders), assistance in meeting the identified needs, and monitoring of the participant's progress. The third study (74) examined the effect of three interventions: community reinforcement approach, motivational enhancement therapy and strengths based case management. We have chosen to focus on the case management intervention as the intervention group for this review. The case management intervention included case managers linking participants with resources in the community, securing needed services, focusing on the clients’ strengths and giving the client high degree of responsibility. The fourth study (77) examined the effect of case management which was a hybrid between brokered case management and full‐services models. There was a focus on linking patients with services (medical, psychiatric, social, legal and social), arranging appointments and accompanying participants to appointments. In the fifth study (26), case management was provided for an average of 3 months and included ordinary case management services (not described) and provision of immediate tangible resources (e.g. transport tokens, food vouchers, medical care and rent deposits).

The case management interventions were compared to usual services (26, 64), case management without an occupational therapist(47), brief contact (77), or two other interventions that did not included case management or housing programs(74).

##### 1.B.1. Low intensity case management compared to usual services

We found two studies that compared low intensity case management to usual services (26, 64) in the USA.

Usual services were described as services that are usually provided to individuals with substance abuse disorders after discharge from rehabilitation (26) or services that clientshad been receiving prior to study enrolment(64).

The target populations in the two studies differed (individuals with long term mental illness and individuals with substance abuse disorders), which dictated the type of usual services the comparison groups received.

###### Primary outcomes: Housing status and homelessness

In the first study (64),participants in the intervention group reported a mean of 44.3 days in better housing during the 14 months prior to follow‐up compared to 32.3 days for the control group. The intervention group also reported a mean of 15.1 days in worse housing compared to 33.4 days for the control group for the same time period. There was not enough information to assess the difference between groups.

In the second study (26), participants in the intervention group increased their residential stability by 9 days during the 60 days prior to the 12 month follow‐up interview. No information was reported for the control group.

The results and quality assessments for low intensity case management compared to usual services are summarized in Table 7. The complete GRADE evidence profile is shown in Appendix 8, Table 8.1.5.

**Table 7:** Summary of findings table for effects of low intensity case management vs usual services (Marshall 1995, Sosin 1995)

**Patient or population**: adults with mental illness or substance abuse problems **Setting**: USA **Intervention**: low intensity case management **Comparison**: usual services					
Outcomes	Anticipated absolute effects^*^ (95% CI)		Relative effect (95% CI)	№ of participants (studies)	Quality of the evidence (GRADE)
	**Risk with usual services**	**Risk with low intensity case management**			
Number of days in better housing assessed with: Unclear follow up: 14 months	The mean number of days in better housing was **32.3** days	The mean number of days in better housing in the intervention group was 12 days more (CI not reported)	‐	80 (1 RCT)	⨁◯◯◯ VERY LOW ^1^ ^,^ ^2^
Number of days in worse housing assessed with: unclear follow up: 14 months	The mean number of days in worse housing was **33.4** days	The mean number of days in worse housing in the intervention group was 18,3 days fewer (CI not reported)	‐	80 (1 RCT)	⨁◯◯◯ VERY LOW ^1^ ^,^ ^2^
Number of days in stable housing during past 60 days assessed with: self‐report follow up: 12 months	The mean number of days in stable housing during past 60 days was **36.0** days	The mean number of days in stable housing during past 60 days in the intervention group was 5,7 days more (CI not reported)	‐	191 (1 RCT)	⨁◯◯◯ VERY LOW ^1^ ^,^ ^3^
*The risk in the intervention group (and its 95% confidence interval) is based on the assumed risk in the comparison group and the **relative effect** of the intervention (and its 95% CI).**CI:** Confidence interval;**MD:** Mean difference					

1. Risk of performance bias. Inadequate reporting of methods for dealing with missing data and blinding.

2. Fewer than 400 participants. Unkown confidence interval.

3. Risk of selection bias and attrition bias. Inadequate reporting of blinding methods.

###### What does the evidence say?

It is uncertain whether low intensity case management compared to usual services improves housing stability and/or reduces homelessness (very low certainty evidence).

##### 1.B.2. Low intensity case management with an occupational therapist compared to low intensity case management without an occupational therapist

We found one study (47)that compared low intensity case management to low intensity case management in the USA. In this study the comparison condition was identical to the intervention, but with a regular case manager instead of an Occupational Therapist (OT) as case manager.

###### Primary outcomes: Housing status

The authors of the study measured and report how the participants’ current housing situation differs from their ideal housing standards according to an unspecified 13‐point scale. The mean for the intervention group at 12 months was 1.04 below their ideal and for the control group 1.71 below their ideal housing situation. The authors state that the average variance from ideal housing was lower at 12 months than at baseline for the intervention group (t(24)=‐2.16, p=0.04) but there was no difference for the control group from baseline to 12 months.

The results and quality assessments for low intensity case management (with OT) vs low intensity case management (no OT) for homeless adults with mental illness are summarized in Table 8. The complete GRADE evidence profile is shown in Appendix 8, Table 6.1.6.

**Table 8:** Summary of findings table for effects of low intensity case management (with Occupational therapist) vs low intensity case management (no occupational therapist) (Chapleau 2012)

**Patient or population**: homeless adults with mental illness **Setting**: USA **Intervention**: low intensity case management (with OT) **Comparison**: low intensity case management (without OT)					
Outcomes	Anticipated absolute effects^*^ (95% CI)		Relative effect (95% CI)	№ of participants (studies)	Quality of the evidence (GRADE)
	**Risk with low intensity case management**	**Risk with low intensity case management**			
Variation from ideal housing assessed with: 13‐point scale not specified follow up: 12 months	The intervention group reported less variance from ideal housing at 12 months than at baseline. There was no difference in variation from ideal housing for control group from baseline to 12 month follow‐up.		57 (1 RCT)	⨁◯◯◯ VERY LOW ^1^ ^,^ ^2^
*The risk in the intervention group (and its 95% confidence interval) is based on the assumed risk in the comparison group and the **relative effect** of the intervention (and its 95% CI).**CI:** Confidence interval					

1. Risk of performance bias and reporting bias. Inadequate reporting of randomization and allocation concealment methods.

2. Fewer than 400 participants.

###### What does the evidence say?

It is uncertain whether low intensity case management compared to low intensity case management has an effect on the amount of time spent in ideal housing (very low certainty evidence).

##### 1.B.3. Low intensity case management compared to other intervention (no case management or housing component)

We found two studies (74, 77)that compared low intensity case management to other interventions without case management or housing components in the USA.

A total of 460 participants were randomized to either case management (N=183) or another intervention (N=277). The participants were recruited between 1994 and 1996 (77)or between 2006 and 2009 (74).

In the first study (77) the comparison group received brief contact, which is described as one or two sessions with a counsellor with a ratio of approximately 100 participants to one case manager, which involved education about reducing HIV transmission and referrals to other services. The focus of the original study was to investigate brief contact. Case management was used in the control condition. However, we have only reported raw data here, and not the effect size as it was calculated and reported in the original publication. The type of comparison condition thus does not impact the results reported here. In the second study (74), the two comparison interventions were community reinforcement approach (CRA) and motivational enhancement therapy (MET). CRA is described as an operant‐based behavioural intervention and focuses on building up skills (anger management, social and recreational counselling, and refusal skills training) within the community to achieve and maintain sobriety. MET is an adaptation of motivational interviewing and was described as lower frequency treatment compared to the other two interventions.

###### Primary outcome: Homelessness

Both studies reported outcomes related to homelessness. In the first study (77),the authors report the number of participants who reported being homeless at each follow‐up point; however, the number of participants included in the analysis for each follow‐up point is unclear. At 18 months 11.3% of participants in the intervention group and 13.8% participants in the comparison group reported being homeless.

In the second study (74), participants report the mean percentage of days homeless during the 90 days prior to each follow‐up interview. At the 12 month follow‐up participants in the intervention group (N=64) reported 20.51 days (SD=35.13) as homeless compared to 20.85 days (SD=34.95) for participants in the community reinforcement approach group (N=70) and 21.89 days (SD=35.31) for participants in the motivational enhancement therapy group (N=69). All three groups reported fewer days spent homeless leading up to the final interview compared to the period before baseline assessment. There was no difference between the low intensity case management group and either the CRA group (MD=‐0.34, 95%CI=‐12.22 to 11.54) or the MET group (MD=‐1.38, 95%CI=‐13.36 to 10.60).

It is not possible to report the findings from these studies in forest plots given the lack of information reported in the first study (77), and the comparison with two types of control conditions in the second study (74).

The results and quality assessments for low intensity case management compared to another intervention with no case management or housing component for youth and adults with substance abuse problems are summarized in Table 8. The complete GRADE evidence profile is shown in Appendix 8, Table 8.1.7.

**Table 8:** Summary of findings table for effects of low intensity case management vs other intervention (no case management or housing component) (Sorensen 2003, Slesnick 2015)

**Patient or population**: youth and adults with substance abuse problems **Setting**: USA **Intervention**: low intensity case management **Comparison**: other intervention (no case management or housing component)					
Outcomes	Anticipated absolute effects^*^ (95% CI)		Relative effect (95% CI)	№ of participants (studies)	Quality of the evidence (GRADE)
	**Risk with other intervention (no case management or housing component)**	**Risk with low intensity case** **management**			
Number of participants homeless at follow‐up assessed with: Not reported follow up: 18 months	11.3% of participants in the intervention group reported being homeless at 18 month follow‐up compared to 13.8% of participants in the comparison group.			190 (1 RCT)	⨁◯◯◯ VERY LOW ^1^ ^,^ ^2^
Number of days homeless during 90 days prior to follow‐up assessed with: self‐report (Form 90) follow up: 18 months	There was no difference between the low intensity case management group and either the CRA group (MD=‐0.34, 95%CI=‐12.22, 11.54) or the MET group (MD=‐1.38, 95%CI=‐13.36, 10.60).		202 (1 RCT)	⨁◯◯◯ VERY LOW^3^ ^,^ ^4^
*The risk in the intervention group (and its 95% confidence interval) is based on the assumed risk in the comparison group and the **relative effect** of the intervention (and its 95% CI).**CI:** Confidence interval					

1. Risk of performance bias. Inadequate reporting of methods.

2. Fewer than 300 participants.

3. Inadequate reporting of blinding of participants and personnel and outcome assessors.

4. Fewer than 400 participants.

###### What does the evidence say?

It is uncertain whether low intensity case management compared to another intervention with no case management or housing component has an effect for youth and adults with substance abuse problems.

#### Category 1C: Critical time intervention

In all three studies that examined the effect of Critical Time Intervention compared to usual services (56, 72, 79), the active part of the Critical Time Intervention was nine months; however the length of follow‐up and after care activities in the three studies varied.

##### 1.C.1. Critical time intervention compared to usual services

The three included studies targeted either single mothers living with at least one child between 18 months and 16 years and living in shelters with mental illness and/or substance dependence(72), or adults with severe mental illness who are homeless or at‐risk of homelessness (56, 79).

###### Primary outcome: Homelessness

All three of the included studies examined the effect of critical time intervention compared to usual services on homelessness (56, 72, 79).

Results from these studies could not be pooled due to lack of details in reporting of results. In the first study (56), 58 participants from the intervention group and 59 from the control group were included in analyses. Homelessness was measured in two ways. First, participants reported via The Personal History Form ever versus never being homeless in the 18 weeks prior to the last follow‐up interview at 18 months. Fewer participants in the intervention group experienced homelessness during this period (3/58) than in the control group (11/59). The authors controlled for baseline homelessness and used a logistic regression to model the impact of assignment to the intervention group on homelessness during the final 18 weeks of the study and found a that Critical Time Intervention reduced the number of days spent homeless compared to usual services. However, the 95% confidence interval indicates that Critical Time Intervention might increase the number of days homeless (OR=0.22, 95%CI 0.06 to ‐0.88). Secondly, participants reported total number of days homeless during the 18 weeks prior to the 18 month follow‐up interview. Participants in the intervention group reported fewer days homeless (M=6) compared to the control group (M=20) (Poisson regression to control for baseline homelessness, p<0.001). The results from this study also showed that Participants in the Critical Time Intervention experienced fewer days homeless during the study period (1,812 nights) compared to the control group (2,403 nights).

In the second study (79),participants reported the number of nights homeless out of 30 days prior to each monthly interview up to 18 months using the Personal History Form. The authors calculated the mean number of nights across each follow‐up period. The intervention group reported approximately one third the number of nights homeless (M=30) as the control group (M=90) (Diff=‐61, z=2.8, p=.003), Normal approximation, 95% CI ‐105 to ‐19, Nonparametric Bootstrap: 95%CI ‐110 to ‐19). Furthermore, the authors reported that the difference between the two groups seemed to widen between after the active part of the intervention ended (i.e. between 9 and 18 months). This study also reports the number of non‐homelessness nights during the study period (mean number of days reported each month up to 18 month follow‐up). The intervention group reported more nights in housing (not homeless) (M= 508.0, SD=60) than the control group (M=450, SD=139) (MD=58, t=2.64, df=64, p=0.01).

In the third study (72), participants were followed for 15 months. The authors reported the length of time to leave shelter, and the number of days before moving into stable housing. Reports were given using a structured residential follow‐back instrument. More families in the intervention group (N=97) left shelter than in the control group (N=113), and the transition from shelter to housing occurred faster with the intervention group. The intervention group used a mean number of 91.25 days (SD=82.3) to first move into stable housing compared to a mean of 199.15 days (SD=125.4) for control group participants. The majority of the intervention group was rehoused after two to three months compared to five months for the control group.

The results and quality assessments for critical time intervention compared to usual services are summarized in Table 10. The complete GRADE evidence profile is shown in Appendix 8, Table 8.1.8.

**Table 10:** Summary of findings for the effects of critical time intervention vs usual services (Herman 2011, Samuels 2015, Susser 1997)

**Patient or population**: adults who are homeless or at‐risk of becoming homeless, with or without dependent children **Setting**: USA **Intervention**: critical time intervention **Comparison**: usual services					
Outcomes	Anticipated absolute effects^*^ (95% CI)		Relative effect (95% CI)	№ of participants (studies)	Quality of the evidence (GRADE)
	**Risk with usual services**	**Risk with critical time intervention**			
Number of participants who experienced homelessness during study period assessed with: The Personal History Form ‐ dichotomized to never versus ever homeless follow up: 18 months	186 per 1 000	**48 per 1 000** (14 to ‐253)	**OR 0.22** (0.06 to ‐0.88)	117 (1 RCT)	⨁⨁◯◯ LOW ^1^ ^,^ ^2^
Number of days homeless assessed with: The Personal History Form, Total for 18 weeks prior to follow‐up or mean number of days during 30 days prior to each monthly follow‐up interview follow up: 18 months	Participants in the intervention group reported fewer days homeless (M=6) compared to the control group (M=20) (Poisson regression to control for baseline homelessness, p<0.001) (Herman 2011). The intervention group reported approximately one third the number of nights homeless (M=30) as the control group (M=90) (Diff=‐61 (z=2.8, p=.003) (Susser 1997).		‐	213 (2 RCTs)	⨁⨁◯◯ LOW ^3^ ^,^ ^4^
Mean number of nights not homeless over study period assessed with: Personal History Form assessed for 30 days prior to each monthly follow‐up interview follow up: 18 months	The mean mean number of nights not homeless over study period was **450** days	The mean number of nights not homeless over study period in the intervention group was 58 days more (15,17 more to 100,83 more)	‐	96 (1 RCT)	⨁⨁◯◯ LOW ^4^ ^,^ ^5^
Number of days spent homeless over study period Assessed with The Personal History Form Follow‐up: 18 months	Participants in the Critical Time Intervention experienced fewer days homeless (1812 nights) compared to the control group (2403 nights).		‐	117 (1 RCT)	⨁⨁◯◯ LOW ^1^ ^,^ ^2^
Length of time to leave shelter assessed with: Structured residential follow‐back instrument follow up: 15 months	The mean length of time to leave shelter was 199.15 days	The mean length of time to leave shelter in the intervention group was 107,9 days fewer (136,23 fewer to 79,57 fewer)	‐	210 (1 RCT)	⨁⨁◯◯ LOW ^4^ ^,^ ^6^
*The risk in the intervention group (and its 95% confidence interval) is based on the assumed risk in the comparison group and the **relative effect** of the intervention (and its 95% CI).**CI:** Confidence interval;**OR:** Odds ratio;**MD:** Mean difference					

1. Risk of selection bias and performance bias.

2. Fewer than 300 participants.

3. Risk of selection bias in one study. Risk of performance bias in both studies. Inadequate reporting of randomization and allocation concealment methods in one study.

4. Fewer than 400 participants.

5. Risk of performance bias. Inadequate reporting of randomization and allocation concealment methods.

6. Inadequate reporting of blinding methods. Risk of reporting bias.

###### What does the evidence say?

Critical time intervention compared to usual services for adults with mental illness:

- May lead to little or no difference in the number of people who experience homelessness (low certainty evidence).
- May lead to fewer days spent homeless (low certainty evidence).
- May lead to more days spent not homeless (low certainty evidence).
- May reduce the amount of time to leave a shelter (and move to independent housing) (low certainty evidence).

### Category 2: Abstinence‐contingent housing programs

#### Description of the included studies

We found six studies with eight comparisons on the effects of abstinence‐contingent housing programs(26, 58, 66‐68, 75). All of the included studies were conducted in the USA. The data for the included studies were collected between 1991 and 2004. Within the category of abstinence‐contingent housing programs, we identified three subcategories (see Table 11).

**Table 11:** Overview of abstinence‐contingent housing programs comparisons

**Intervention**	**Comparison**
2.A. Abstinence‐contingent housing programs with case management	2.A.1. Usual services
	2.A.2. Case management
2.B. Abstinence‐contingent housing programs with day treatment	2.B.1. Usual services
	2.B.2. Day treatment
	2.B.3. Non‐abstinence‐contingent housing programs with day treatment
	2.B.4. Abstinence‐contingent housing programs with community reinforcement approach

The above interventions are compared to usual services, or other interventions. That is, abstinence‐contingent housing is compared to another active intervention. Table 12 presents an overview of the populations, interventions, comparisons and outcomes in the six included studies. In some studies the duration of the intervention is reported and differs from the longest follow‐up point. In these instances we have reported both the duration of the intervention and the longest follow‐up point.

**Table 12:** Description of studies that evaluated effects of abstinence‐contingent housing

**Study (ref); country**	**Population** **(N, eligibility)**	**Intervention, Duration, FU (FU) in mos (mos), N**	**Comparison, N**	**Primary outcome**
Kertesz 2007(58), USA	N=129 at‐risk or homeless with substance dependence	Abstinence‐contingent housing with day treatment Duration: 6 mosFU: 12 mos N=63	Day treatment only N=66 Non‐abstinence‐contingent housing with day treatment N=66	Proportion of participants in stable housing >45 of previous 60 days
Milby 1996(67), USA	N=176 homeless, substance dependence	Abstinence‐contingent housing, vocational training and work therapy with day treatment Duration: 24 weeks FU: 12 mos N=69	Usual services N=62	Mean number of days housed in previous 60 days
Milby 2003(66), USA	N=141 at‐risk or homeless with substance dependence	Abstinence‐contingent housing, vocational training and work therapy with day treatment Duration: 24 weeks N=72	Day treatment only N=69	Mean number of days housed in previous 60 days
Milby 2010(68), USA	N=206 homeless, substance dependence, mental illness	Abstinence‐contingent housing, vocational training and work therapy with community reinfocement approach Duration: 24 weeks FU: 18 mos N=103	Abstinence‐contingent housing, vocational training and work therapy N=103	Proportion of participants housed more than 40 of previous 60 days
Smith 1998(75), USA	N=106 homeless with alcohol dependence	Abstinence‐contingent housing with community reinforcement approach Duration: varied, minimum 3 weeks FU: 12 mos N=64	Abstinence‐contingent housing with day treatment N=42	Proportion homeless
Sosin 1995, USA(26)	N=299 at‐risk or homeless with substance dependence	Abstinence‐contingent housing with case management Duration: average 6 mos FU: 12 mos N=108	Usual care N=121 Case management only N=70	Number of days housed of previous 60 days

#### Description of the intervention

All of the interventions in the included studies had some component of abstinence‐contingent housing. Abstinence‐contingent housing in the included studies consisted of program‐provided housing for a set period of time (6‐8 months) with or without some rent contributed by the participants after the initial phase. Conditionality of tenancy for the participants consisted of a contract agreeing to abstinence and then regular urine testing to screen for substance use. Housing for participants was not segregated (segregated housing is separated from the general public and only for individuals receiving social assistance).

#### Category 2A: Abstinence‐contingent housing with case management

We found one study (26) with two comparisons that examined the effect of abstinence‐contingent housing with case management in the USA. Participants were recruited from 1991 to 1992 and randomized to one of three groups: abstinence‐contingent housing with the progressive independence model of case management (ACH+CM), the progressive independence model of case management only (CM), or usual services (US).

The abstinence‐contingent housing component consisted of supported housing in low‐income apartment blocks where tenancy was contingent upon following program rules (26). The case management component in this study was described as a “progressive independence model” with a focus on providing immediate tangible resources while supporting further treatment for substance abuse and other relevant problems. Case management was also contingent on following a contract which participants signed before the start of the intervention.

Participants in the case management condition received an average of three months care, while participants in the housing with case management condition received an average of six months of care.

Abstinence‐contingent housing with case management was compared to usual services (26)and case management only (26). Usual services consisted of aftercare services such as referrals to outpatient or inpatient substance abuse agencies or welfare offices.

##### 2.A.1. Abstinence‐contingent housing with case management compared to usual services

One study (26)examined the effect of abstinence‐contingent housing with case management compared to usual services.

###### Primary outcome: Housing stability

Results from the included study(26)show that participants in the intervention group reported more days in housing than participants in the control groupat the 12 month follow‐up interview (MD=6.4, 95%CI= 6.18 to 6.62). The results for abstinence‐contingent housing with case management compared to usual services only are presented in Table 12. The results are controlled for length of time from baseline to the second follow‐up interview, which varied due to difficulties arranging meetings with participants and the number of days in the relevant period spent in a controlled environment (e.g. prison or hospital) since they are not truly homeless or housed during this time. Other control variables such as characteristics which were found to vary across the treatment conditions are also controlled for (being recruited from a particular short‐term program, reported perception of health problems at baseline, access to an automobile, having ever been married, having foster care experience as a child or having lived with one's mother continuously until 18). Not enough information was provided to present the results in a forest plot.

The results and quality assessments for abstinence‐contingent housing with case management compared to usual services are summarized in Table 13. The complete GRADE evidence profile is shown in Appendix 8, Table 8.2.1.

**Table 13:** Summary of findings table for the effects of abstinence‐contingent housing with case management vs usual services (Sosin 1995)

**Patient or population**: adults who are homeless or at‐risk of homelessness with substance abuse problems **Setting**: USA **Intervention**: abstinence‐contingent housing with case management **Comparison**: usual services					
Outcomes	Anticipated absolute effects^*^ (95% CI)		Relative effect (95% CI)	№ of participants (studies)	Quality of the evidence (GRADE)
	**Risk with Usual services**	**Risk with abstinence‐contingent housing with case management**			
Housing stability assessed with: Not reported follow up: 12 months	The mean housing stability was<bold>0</bold> days	The mean housing stability in the intervention group was 6.4 days more (6.18 more to 6.62 more)	‐	323 (1 RCT)	⨁◯◯◯ VERY LOW^1^ ^,^ ^2^
*The risk in the intervention group (and its 95% confidence interval) is based on the assumed risk in the comparison group and the **relative effect** of the intervention (and its 95% CI).**CI:** Confidence interval;**MD:** Mean difference					

1. Risk of selection bias and attrition bias. Inadequate reporting of blinding of participants, personnel and outcome assessors.

2. Fewer than 400 participants.

###### What does the evidence say?

It is uncertain whether abstinence‐contingent housing with case management compared to usual services leads to a difference in number of days spent in in stable housing (very low certainty evidence).

##### 2.A.2. Abstinence‐contingent housing with case management compared to case management

One study (26)compared abstinence‐contingent housing with case management to case management only.

###### Primary outcome: Housing

Results from this study (26)show that participants in the intervention group (N=108) reported a mean increase of 25.6 days housed of the previous 60 days from baseline to 12 months compared to a mean increase of 21.2 days for the comparison group (N=70). Not enough information was reported to determine if there is a difference between groups, or to present the results in a forest plot.

The results and quality assessments for abstinence‐contingent housing with case management compared to case management only is summarized in Table 14. The complete evidence profile is presented in Appendix 8, Table 8.2.2.

**Table 14:** Summary of findings table for the effects of abstinence‐contingent housing with case management services vs case management (Sosin 1995)

**Patient or population**: adults who are homeless or at‐risk of homelessness with substance abuse problems **Setting**: USA **Intervention**: abstinence‐contingent housing with case management **Comparison**: case management					
Outcomes	Anticipated absolute effects^*^ (95% CI)		Relative effect (95% CI)	№ of participants (studies)	Quality of the evidence (GRADE)
	**Risk with case management**	**Risk with abstinence‐contingent housing with case management**			
Change in number of days housed from baseline to follow‐up assessed with: Self‐report follow up: 12 months	The mean change in number of days housed from baseline to follow‐up was<bold>21.2</bold> days	The mean change in number of days housed from baseline to follow‐up in the intervention group was 4.4 days more (CI not reported)	‐	178 (1 RCT)	⨁◯◯◯ VERY LOW^1^ ^,^ ^2^
*The risk in the intervention group (and its 95% confidence interval) is based on the assumed risk in the comparison group and the **relative effect** of the intervention (and its 95% CI).**CI:** Confidence interval;**MD:** Mean difference					

1. Risk of selection bias and attrition bias. Inadequate reporting of blinding of participants, personnel and outcome assessors.

2. Fewer than 400 participants.

###### What does the evidence say?

It is uncertain whether abstinence‐contingent housing with day treatment compared to case management only leads to a difference in the number of days spent in stable housing (very low certainty evidence).

#### Category 2B: Abstinence‐contingent housing with day treatment

Three studies evaluated the effect of abstinence‐contingent housing with day treatment in USA (58, 66, 67).

The abstinence‐contingent housing with day treatment intervention consisted of two general components: housing programs in which tenancy is conditional upon maintained sobriety and/or treatment, and day treatment(58, 66, 67).

In one study with two comparisons (58),participants were required to pay to remain in housing (but were not removed if unable to pay). The housing component in this study was only part of treatment and available for a maximum of six months. No information was available regarding segregation of the housing or whether it was individual or group housing.

In the second study (67),participants’ tenancy in program management housing was contingent on abstinence. No information was provided in this study regarding rent payment, or the form of housing provided.

In the third study (66),participants were moved into rent free and furnished housing provided by the program after achieving abstinence. Participants in this study received segregated group or individual housing. After phase I half of the clients remained in this housing arrangement, and half moved to program‐managed individual houses.

In these three included studies(58, 66, 67), participants in the intervention group received day treatment in the first phase of a two phase intervention. The second phase of the intervention included abstinence‐contingent work therapy with minimum wage which could be used towards rent payments. Some participants also received aftercare (58)(66). Formal treatment ended after six months (58, 67, 68).

Participants in the comparison groups received usual services (67), day treatment only (58, 66), or non‐abstinence‐contingent housing with day treatment (58).

##### 2.B.1. Abstinence‐contingent housing with day treatment compared to usual services

One study compared abstinence‐contingent housing with day treatment to usual services (67).

###### Primary outcome: Homelessness

Results from the included study (67) showed that participants in the intervention group reported a mean of 52 fewer days homeless in the previous 60 days at 12 month follow‐up than in the previous 60 days at baseline. There was no change in number of days homeless for the control group.

The results and quality assessments for abstinence‐contingent housing with day treatment compared to usual services is summarized in Table 15. A complete GRADE evidence profile is shown in Appendix 8, Table 8.2.3.

**Table 15:** Summary of findings table for the effects of abstinence‐contingent housing with day treatment vs usual services (Milby 1996)

**Patient or population**: homeless adults with substance abuse problems **Setting**: USA **Intervention**: abstinence‐contingent housing with day treatment **Comparison**: usual services			
Outcomes	Impact	№ of participants (studies)	Quality of the evidence (GRADE)
Change in number of days homeless in past 60 days from baseline to 12 months assessed with: Personal History Form follow up: 12 months	The mean change in number of days homeless in past 60 days from baseline to 12 months was 0 for the control group. The intervention group had a mean change of 52 fewer days homeless from baseline to 12 months, p=0.026.	131 (1 RCT)	⨁⨁◯◯ LOW ^1^ ^,^ ^2^
*The risk in the intervention group (and its 95% confidence interval) is based on the assumed risk in the comparison group and the **relative effect** of the intervention (and its 95% CI).**CI:** Confidence interval			

1. Risk of performance bias and attrition bias.

2. Less than 400 participants.

###### What does the evidence say?

Abstinence‐contingent housing with day treatment compared to usual services may lead to fewer days spent homeless (low certainty evidence).

##### 2.B.2. Abstinence‐contingent housing with day treatment compared to day treatment

Two studies (58, 66)examined the effect of abstinence‐contingent housing with day treatment compared to day treatment.

Participants in the comparison groups received day treatment only which was similar to the day treatment offered to the intervention group for months 1‐2 and 3‐6(58, 66). These participants were not offered housing.

###### Primary outcomes: Housing stability and homelessness

Two studies examined the effect of abstinence‐contingent housing with day treatment compared to day treatment only on the number of days participants reported being housed during a period of time. In one study (66),participants in both groups reported a greater number days housed of the previous 60 days at 12 months compared to baseline; however, participants in the intervention group showed a greater increase than participants in the control group (MD=18.7 (SE=3.9) compared to MD=16.2 (SE=3.5)) (MD=2.50 [95%CI 1.28 to 3.72]). In the other study (58),participants in the intervention group reported a greater increase in number of days housed from baseline to 12 months (M=17.7 (SD=33.8)) than participants in the control group (M=9.5 (SD=31.0)) (MD=8.20 [95%CI 5.74 to 10.66]).^1^

**Figure 8:**
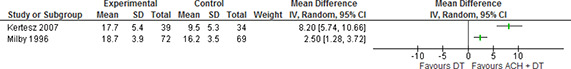
Days in stable housing, 12 months, abstinence‐contingent housing with day treatment vs day treatment only

When the results were pooled using SMD, I^2^=86%. Since this heterogeneity could not be explained, we chose not to pool the results (Figure 8).

The results and quality assessments for abstinence‐contingent housing with day treatment compared to day treatment only is summarized in Table 16. The complete GRADE evidence profile is shown in Appendix 8, Table 8.2.4.

**Table 16:** Summary of findings table of the effects of abstinence‐contingent housing with day treatment vs day treatment (Kertesz 2007; Milby 1996)

**Patient or population**: homeless adults with substance abuse problems **Setting**: USA **Intervention**: abstinence‐contingent housing with day treatment **Comparison**: day treatment					
Outcomes	Anticipated absolute effects^*^ (95% CI)		Relative effect (95% CI)	№ of participants (studies)	Quality of the evidence (GRADE)
	**Risk with day treatment**	**Risk with** **abstinence‐contingent housing with day treatment**			
Changes in mean days housed in past 60 days between baseline and 12 months ‐ self‐report assessed with: Retrospective Interview for Housing, Employment, and Treatment History follow up: 12 months	The mean changes in mean days housed in past 60 days between baseline and 12 months ‐ self‐report was<bold>16.2</bold> days	The mean changes in mean days housed in past 60 days between baseline and 12 months ‐ self‐report in the intervention group was 5.25 days more (0.34 fewer to 10.83 more)	‐	214 (2 RCTs)	⨁◯◯◯ VERY LOW^1^ ^,^ ^2^
Changes in mean days employed in past 60 days between baseline and 12 months assessed with: Retrospective Interview for Housing, Employment, and Treatment History ‐ self report follow up: 12 months	The mean changes in mean days employed in past 60 days between baseline and 12 months was<bold>0</bold> days	The mean changes in mean days employed in past 60 days between baseline and 12 months in the intervention group was 1.62 days more (0.99 fewer to 4.22 more)	‐	214 (2 RCTs)	⨁◯◯◯ VERY LOW ^1^ ^,^ ^2^
*The risk in the intervention group (and its 95% confidence interval) is based on the assumed risk in the comparison group and the **relative effect** of the intervention (and its 95% CI).**CI:** Confidence interval;**MD:** Mean difference					

1. Risk of performance bias and attrition bias. Inadequate reporting of allocation concealment in both studies.

2. Less than 400 participants.

###### What does the evidence say?

It is uncertain whether abstinence‐contingent housing with day treatment compared to day treatment only leads to a difference in number of days spent in stable housing or employed (very low certainty evidence).

##### 2.B.3. Abstinence‐contingent housing with day treatment compared to non‐abstinence‐contingent housing with day treatment

One study examined the effects of abstinence‐contingent housing with day treatment compared to non‐abstinence contingent housing with day treatment (58). The comparison group consisted of an equivalent intervention as the abstinence‐contingent housing group; however, continued tenancy was not dependent on sobriety (i.e. the results of the urine tests). Both groups received the day treatment component.

###### Primary outcome: Housing stability

Results from this study (58)showed that participants in the intervention group reported a greater increase in the number of days in stable housing in the 60 days prior to follow‐up between baseline and follow‐up (12 months) (MD=17.7 (SD=33.8)) than participants in the control group (MD=14.2 (SD=31.7)).

The results and quality assessments for abstinence‐contingent housing day treatment compared to non‐abstinence‐contingent housing with day treatment for housing stability and homelessness are summarized in Table 17. A complete GRADE evidence profile is shown in Appendix 8, Table 8.2.5.

**Table 17:** Summary of findings table for the effects of abstinence‐contingent housing with day treatment vs non‐abstinence‐contingent housing with day treatment (Kertesz 2007)

**Patient or population**: homeless adults with substance abuse **Setting**: USA **Intervention**: abstinence‐contingent housing with day treatment **Comparison**: non‐abstinence contingent housing with day treatment					
Outcomes	Anticipated absolute effects^*^ (95% CI)		Relative effect (95% CI)	№ of participants (studies)	Quality of the evidence (GRADE)
	**Risk with non‐abstinence contingent housing with day treatment**	**Risk with abstinence‐contingent housing with day treatment**			
Days housed ‐ self report Change in mean days housed in past 60 days between baseline and 12 months assessed with: Retrospective Interview for Housing, Employment, and Treatment History follow up: 12 months	The mean days housed ‐ self report Change in mean days housed in past 60 days between baseline and 12 months was **0** days	The mean days housed ‐ self report Change in mean days housed in past 60 days between baseline and 12 months in the intervention group was 3.5 days more (1.22 more to 5.78 more)	‐	82 (1 RCT)	⨁◯◯◯ VERY LOW ^1^ ^,^ ^2^
*The risk in the intervention group (and its 95% confidence interval) is based on the assumed risk in the comparison group and the **relative effect** of the intervention (and its 95% CI).**CI:** Confidence interval;**MD:** Mean difference					

1. Risk of performance bias and attrition bias. Inadequate reporting of allocation concealment.

2. Less than 400 participants.

###### What does the evidence say?

It is uncertain whether abstinence‐contingent housing with day treatment compared to non‐abstinence‐contingent housing with day treatment leads to a difference in number of days spent in stable housing (very low certainty evidence).

##### 2.B.4. Abstinence‐contingent housing with day treatment compared to abstinence‐contingent housing with community reinforcement approach

We found two studies that examined the effect of abstinence‐contingent housing with day treatment compared to abstinence‐contingent housing with community reinforcement approach (68, 75) in the USA.

Participants in one study (68) were provided with a furnished and rent free apartment and vocational training which was contingent on continued sobriety during phase I (weeks 2‐8). In Phase II (weeks 3‐24) participants were required to pay a small amount of rent (not specified) from program provided stipends. Participants who maintained abstinence were moved to a transitional housing program. In Phase III (week 25‐end) continued tenancy in abstinence‐contingent program housing was only available when space was available at a modest rent.

In the other study (75),participants were housed in grant‐supported housing for a maximum of three months contingent on sobriety. However, participants who had secured a job and saved a pre‐set amount of money could stay one additional month.

In both studies, participants in the comparison groups received the same abstinence‐contingent housing, vocational training and work therapy as participants in the intervention group, with the community reinforcement approach in addition.

###### Primary outcomes: Homelessness and stable housing

Homelessness was reported in one study(75). The rate of homelessness for participants in the intervention group (N=64; 13.7%) was lower than for the control group (N=42; 34%) at four months. There was little or no difference between groups (when reported at all) at the other follow‐up points.

Two studies reported outcomes related to stable housing. In one study (75), more participants from the CRA group (62.5%) were paying for housing (rather than staying with friends or in a motel) at the 12 months follow‐up than in the day treatment group (44%) (χ²(1, N=80)=2.73, p<0.10).

In the second study (68),a greater proportion of participants in the abstinence‐contingent housing with CRA group (N=103; 44.7%) were housed more than 40 of the previous 60 days at 18 months than in the abstinence‐contingent housing with day treatment group (N=103; 35.6%). There was also a greater increase in proportion of participants housed 40 of the previous 60 days from baseline to 18 months in the CRA group (36%) than in the day treatment group (25.7%).

Not enough data were reported to assess whether there was a difference in time spent in stable housing between the two groups. Furthermore, the outcomes were reported too differently in the two studies to pool the results.

The results and quality assessments for abstinence‐contingent housing with day treatment compared to abstinence‐contingent housing with community reinforcement approach is summarized in Table 18. A complete GRADE evidence profile is shown in Appendix 8, Table 8.2.6.

**Table 18:** Summary of findings table for the effects of abstinence‐contingent housing with day treatment versus abstinence‐contingent housing with community reinforcement approach (Smith 1998; Milby 2010)

**Patient or population**: homeless adults with substance abuse **Setting**: USA **Intervention**: abstinence‐contingent housing with day treatment **Comparison**: abstinence‐contingent housing with community reinforcement approach					
Outcomes	Anticipated absolute effects^*^ (95% CI)		Relative effect (95% CI)	№ of participants (studies)	Quality of the evidence (GRADE)
	**Risk with abstinence‐contingent housing with community** **reinforcement approach**	**Risk with abstinence‐contingent housing with day treatment**			
Mean decrease in proportion homelessness assessed with: Not reported follow up: 4 months	The rate of homelessness in the intervention group (13.7%) was lower than that in the control group (34%) (χ²(1, N=86)=5.10, p=0.024). There was little or no difference at 12 month follow up.	‐	106 (1 RCT)	⨁◯◯◯ VERY LOW^1^ ^,^ ^2^	
Proportion of participants housed more than 40 of past 60 days assessed with: Retrospective Housing, Employment and Substance Abuse Treatment Interview (RHESAT) follow up: 18 months	A greater proportion of participants in the intervention group (44.7%) were housed more than 40 of the previous 60 days at 18 months than in the control group (35.6%). Furthermore, there was a greater increase in pro‐portion of participants housed 40 of the previous 60 days from baseline to 18 months in the intervention group (36%) than in the control group (25.7%).	‐	206 (1 RCT)	⨁◯◯◯ VERY LOW^2^ ^,^ ^3^	
*The risk in the intervention group (and its 95% confidence interval) is based on the assumed risk in the comparison group and the **relative effect** of the intervention (and its 95% CI).**CI:** Confidence interval					

1. Risk of detection and selection bias. Inadequate reporting of allocation concealment methods.

2. Less than 400 participants.

3. Risk of detection bias, selection bias, and performance bias.

###### What does the evidence say?

It is uncertain whether abstinence‐contingent housing with day treatment compared to abstinence‐contingent housing with community reinforcement has an effect on the amount of time spent homeless or in stable housing (very low certainty evidence).

### Category 3: Non‐abstinence‐contingent housing

#### Description of the included studies

We identified eight studies that evaluated the effect of non‐abstinence‐contingent housing (24, 42, 43, 55, 58, 65, 73, 78). Most of the included studies were from the USA (N=6); however, the largest study was from Canada (N=1). Data for the included studies were collected between 1997 and 2013. Within the category of non‐abstinence‐contingent housing programs, we identified two subcategories (see Table 19).

**Table 19:** Overview of non‐abstinence‐contingent housing program comparisons

3. Non‐abstinence contingent housing programs	3.A. Housing First	3.A.1. Usual services
		3.A.2. Abstinence‐contingent housing
	3.B. Non‐abstinence‐contingent housing with high intensity case management	3.B.1. Usual services
	3.B. Non‐abstinence‐contingent group living arrangements with high intensity case management	3.B.2. Non‐abstinence‐contingent independent apartments with high intensity case management
	3.B. Non‐abstinence‐contingent housing with high intensity case management	3.B.3. Abstinence‐contingent housing with high intensity case management
	3.B. Non‐abstinence‐contingent housing with day treatment	3.B.4. Day treatment

These interventions are compared to usual services or other interventions. Table 20 presents an overview of the populations, interventions, comparisons and outcomes in the included studies.

**Table 20:** Description of studies that evaluated effects of non‐abstinence‐contingent housing

**Study (ref); country**	**Population** **(N, description)**	**Intervention, follow‐up (FU) in months (mos), N**	**Comparison, N**	**Primary outcome**
Aubry 2015(42), Canada	N=2148 homeless, mental illness (high or moderate needs)	Housing first with intensive case management or assertive community treatment FU: 21‐24 mos N=1198	Usual services N=950	Housing stability Homelessness
Basu 2011(43), USA	N=407 homeless, medical illness	Housing first with case management FU: 18 mos N=201	Usual services N=206	Housing stability Days housed
Goldfinger 1999(55), USA	N=118 homeless, mental illness	Staffed group homes with intensive case management FU:18 mos N=63	Independent living with intensive case management N=55	Days homeless Days housed
Kertesz 2007(58), USA	N=132 homeless substance dependence	Non‐abstinence‐contingent housing with day treatment Duration: 6 months FU: 12 mos N=66	Day treatment N=66	Residential history
McHugo 2004(65), USA	N=121 21‐60 years, at‐risk of homeless, mental illness	Supported housing with assertive community treatment (parallel housing) FU: 18 mos N=60	Continuum of care housing with intensive case management (integrated housing) N=61	Residential history
Shern 2000(73), USA	N=168 homeless, mental illness	Intensive case management with temporary program managed shelter housing FU: 24 mos N=91	Usual services N=77	Housing status
Stefancic 2007(78), USA	N=269 chronic shelter use, mental illness	Housing First with assertive community treatment FU: 47 mos N=209	Usual services N=51	Days housed Days not housed
Tsemberis 2004(24), USA	N=225 homeless, mental illness	Housing First with assertive community treatment FU: 24 mos N=99	Continuum of care housing N=126	Homelessness Residential stability

#### Description of the intervention

Non‐abstinence‐contingent housing includes a variety of interventions that provide housing to homeless persons without any conditionality attached to their stays (such as abstinence, treatment attendance, etc.). Four of the included studies examined Housing First (with case management), which encourages early placement in stable housing after staying in transitional housing for a short period of time. The other studies examined supportive housing with assertive community treatment(65), staffed group homes with intensive case management(55), and non‐abstinence‐contingent housing with day treatment (58).

#### Category 3A: Housing First

We found four studies that evaluated the effect of Housing First (24, 42, 43, 78). In Housing First treatment and housing domains are considered as being closely linked, but separate domains. In other words, treatment is encouraged, but refusal does not result in removal from housing. The emphasis in Housing First is on consumers’ choice (i.e. the consumer helps to define and plan goals). A central component is that housing is immediately provided if desired, and tenancy is not contingent on adherence to treatment schedules or sobriety. All four studies had two program requirements: tenants had to pay part (30%) of their income (usually Supplemental Security Income) toward the rent by participating in a money management program, and tenants had to meet with a staff member regularly.

One study had three intervention arms and compared two models of the Housing First program (Pathways to housing and Consortium) to usual services (78). As part of the Housing First interventions, participants were offered the ACT model of case management which involves intense case management with a team of professionals that are available 24 hours a day, seven days a week(24, 42, 78). Participants in the second study (24)received the Pathways to Housing model which adds modifications to standard ACT: a nurse practitioner was added to the team to address health problems, and a housing specialist joined the team to coordinate the housing services (24). In the third study(42),participants were divided according to mental health needs (high or moderate) and while the high needs participants received ACT, the moderate needs participants received intensive case management together with Housing First. In the fourth study (43),participants in the intervention group received Housing First with case management (case managers had less than 20 clients each).

Housing in the included studies was provided as group living arrangements or apartments at single and scattered sites (43), or scattered sites only (24, 42, 78).

The intervention was compared to usual services (42, 43, 78), or abstinence‐contingent housing (24).

##### 3.A.1. Housing First compared to usual services

Three studies (42, 43, 78) examined the effect of Housing First on housing stability and homelessness compared to usual services in Canada (42) and the USA (43, 78).

In all three studies the intervention was compared to usual services. Usual services included having access to other housing and support services through other programs in their communities. In one study, however, (78)two groups of participants received a version of the Housing First intervention ‐ either the Pathways to Housing model which is a well‐established model, but new to this particular community, or the Consortium model, which was made up of a consortium of treatment and housing agencies who had no prior experience of operating Housing First (78). The authors also report differences between these two groups.

The included studies reported number of days homeless, in shelter, in respite care, with family/friends, or in paid housing (43), proportion of time homeless (in shelters or on street) and stably housed (42), housing stability (proportion of time housed) (42),and number of participants in stable housing at end of study (78).

###### Primary outcomes: Housing stability and homelessness

The first study (42) examined housing stability in two ways: proportion of time during the last 6 months of the study that participants reported being housed all of the time, some of the time or none of the time, and percentage of days spent in stable housing for each three month period of follow‐up. Sixty‐two percent of participants receiving Housing First reported being housed all of the time compared to 31% of participants who received usual services; 22% of Housing First participants were housed some of the time and 16% none of the time compared to 23% and 46%, respectively, of usual services participants. For the second outcome, Housing First participants were in stable housing an average of 73% of the time compared to an average of 32% the time for participants who received usual services. We were not able to calculate difference between groups due to insufficient reporting of results in the primary study.

This study (42)also reported proportion of time in different types of shelter over the study period: Participants in the Housing First group spent approximately 12% of time in temporary housing, 6% in emergency shelters, 9% in institutions and 3% on the street compared to participants in the usual service group who spent approximately 33% of time in temporary housing, 16% in emergency shelters, 11% in institutions and 8% on the street. We were not able to calculate difference between groups due to insufficient reporting of results in the primary study.

In the second study (43),number of days homelessness was reported at each three month interval follow‐up point and accumulated over the 18 month study period. The results were then annualized (converted to a rate for one year). Participants in the Housing First group reported fewer days homeless than participants in the usual services group ((MD=‐62.3 (SE=12.4), p<0.05) and more days in paid housing (MD=109.9 (SE=8.7), p<0.05) at 18 month follow‐up.

In the third study(78),103 of 209 participants in the Housing First group were placed in permanent housing at the 20 month follow‐up compared to 13 of 51 participants in the case management only group.

We were unable to pool results from the included studies due to difference in how the outcomes were reported.

The results are shown in Table 21.

**Table 21:** Results for Housing First vs usual services on housing stability and homelessness

**Author, year**	**Outcome**	**Housing First**	**Usual services**	**Results**
**Aubry 2015**	Proportion of days homeless	6% ‐ emergency shelters 9% ‐ institutions 3% ‐ street	33% ‐ emergency shelters 16% ‐ institutions 11% ‐ street	‐
**Aubry 2015**	Proportion of days in stable housing	73%	32%	‐
**Basu 2012**	Number of days homeless (mean (SD))	112 (122) N=201	1.9(18) N=204	MD=110.10, 95%CI=93.05, 127.15
**Basu 2012**	Number of days in paid housing (mean (SD))	121 (120) N=201	183.5 (130) N=204	MD= ‐62.5, 95%CI=‐86.86, ‐38.14
**Stefancic 2007**	Number of clients placed in permanent housing	103/209	13/51	RR=1.93, 95%CI=1.19, 3.15

The results and quality assessments for Housing First compared to usual services are summarized in Table 22. The complete GRADE evidence profile is shown in Appendix 8, Table 8.3.1.

**Table 22:** Summary of findings table for the effects of Housing First with case management compared to usual services (Aubry 2015, Basu 2012, Stefancic 2007)

**Patient or population**: homeless adults with mental or chronic medical illness **Setting**: USA/Canada **Intervention**: Housing First **Comparison**: Usual services					
Outcomes	Anticipated absolute effects^*^ (95% CI)		Relative effect (95% CI)	№ of participants (studies)	Quality of the evidence (GRADE)
	**Risk with Usual services**	**Risk with Housing First**			
Number of days homeless assessed with: Self‐report follow up: 18 months	The mean number of days homeless was **185.3** days	The mean number of days homeless in the intervention group was 62,5 days fewer (86,86 fewer to 38,14 fewer)	‐	405 (1 RCT)	⨁⨁⨁◯ MODERATE ^1^
Proportion of time homeless (shelter, street or public place) assessed with: Self‐report follow up: 24 months	Over the course of the study participants in the Housing First group spent less time homeless (in shelter or on street) (9%) than participants in the control group (24%).			2148 (1 RCT)	⨁⨁⨁◯ MODERATE ^2^
Number of days in paid housing assessed with: Self‐report follow up: 12 months	The mean number of days in paid housing was **1.9** days	The mean number of days in paid housing in the intervention group was 110,1 days more (93,05 more to 127,15 more)	‐	405 (1 RCT)	⨁⨁⨁◯ MODERATE ^1^
Proportion of time housed (stable housing includes any long‐term housing arrangement) assessed with: Residential follow‐back calendar follow up: 24 months	Over the course of the study participants in the Housing First group spent more time stably housed (73%) than participants in the control group (32%).			2148 (1 RCT)	⨁⨁⨁◯ MODERATE ^2^
Number of clients placed in permanent housing assessed with: Unclear follow up: 20 months	255 per 1 000	**492 per 1 000** (303 to 803)	**RR 1.93** (1.19 to 3.15)	260 (1 RCT)	⨁⨁◯◯ LOW ^3^ ^,^ ^4^
*The risk in the intervention group (and its 95% confidence interval) is based on the assumed risk in the comparison group and the **relative effect** of the intervention (and its 95% CI).**CI:** Confidence interval;**MD:** Mean difference;**RR:** Risk ratio					

1. Risk of performance bias.

2. Risk of performance bias and detection bias.

3. Risk of selection bias and attrition bias.

4. Fewer than 300 participants.

###### What does the evidence say?

Housing First compared to usual services:

- Probably reduces the number of days spent homeless (moderate certainty evidence).
- Probably reduces the proportion of time an individual spends homeless (moderate certainty evidence).
- Probably increases the number of days in paid housing (moderate certainty evidence).
- Probably increases the proportion of time in stable housing (moderate certainty evidence).
- May increase the number of people placed in permanent housing after 20 months (low certainty evidence).

###### Subgroup analysis

In one study participants were stratified according to mental health needs (42). The authors conducted sub‐group analyses where participants with high support needs for mental health services (high needs) and participants with moderate support needs for mental health services (moderate needs) were examined separately(42). All five sites are included in the high needs analysis, but only four sites are included in the moderate needs analysis because one site did not separate participants according to need level.

High needs participants received Housing First with Assertive Community treatment while moderate needs participants received Housing First with intensive case management. Both groups were compared to participants who received usual services. For participants with high support needs, those receiving Housing First with assertive community treatment reported a greater mean proportion of time in stable housing over the 24 month study period (71%) than the control group (29%) (adjusted absolute difference AAD=42%, 95%CI 28% to 45%, p<0.01)(42).

For participants with moderate support needs, those receiving Housing First with intensive case management had a higher proportion of days stably housed than the control group across all four included study sites (a summary statistic for the total group of participants across sites was not reported).

Stefancic 2007 (78)also examined the difference between the two models of Housing First included in the study in number of clients placed in permanent housing. Sixty two of 105 participants in the Pathways to Housing group were placed and 52 of 104 in the Consortium group were placed. Housing retention rates were also reported for all participants: at the two‐year follow‐up point 84% of Housing First participants were housed compared to 88.5% of control group participants and after 47 months 68% were still housed compared to 78.3% of control group participants. Results of housing retention between the two Housing First groups shows that 88.5% of Pathways participants were still in housing compared to 79% of Consortium participants and 88.5% after two years and 78.3% of Pathways participants were in housing, 57% of Consortium participants after 47 months.

##### 3.A.2. Housing First compared to abstinence‐contingent housing

One study (24)examined the effect of Housing First compared to abstinence‐contingent housing on homelessness in New York, USA.

###### Primary outcomes: Homelessness and housing stability

As the results indicate, the proportion of time participants spent homeless (public space, on the street or in shelter) was recorded at each 3 month follow‐up period over the course of the study. The Housing First group (N=103) reported less time homeless (F(1, 195)=198, p<0.0001) and more time spent stably housed compared to the usual services group (N=103) at all time points. Housing First participants also reported faster decreases in number of days spent homeless (F(4,137)=10.1, p<0.001) and increases in stably‐housed status (F(4,137)=27.7,p<0.001) compared to the usual services group.

The results and quality assessments for Housing First compared to abstinence‐contingent housing are summarized in Table 23. The complete GRADE evidence profile is shown in Appendix 8, Table 8.3.2.

**Table 23:** Summary of findings table for the effects of Housing First vs abstinence‐contingent housing (Tsemberis 2004)

**Patient or population**: adults with mental illness **Setting**: USA **Intervention**: Housing first **Comparison**: abstinence‐contingent housing					
Outcomes	Anticipated absolute effects^*^ (95% CI)		Relative effect (95% CI)	№ of participants (studies)	Quality of the evidence (GRADE)
	**Risk with abstinence‐contingent housing**	**Risk with Housing first**			
Proportion of time spent homeless assessed with: self‐report follow up: 24 months	Participants in the control group spent more time homeless over the duration of the study than Housing First group overall: F(1,195)=198, p<0.0001.	‐	206 (1 RCT)	⨁◯◯◯ VERY LOW ^1^ ^,^ ^2^	
Proportion of time stably housed assessed with: Self‐report follow up: 24 months	Participants in the Housing First group had faster increases in stably housed status compared to participants in the control condition: F(4, 137)=27.7, p<0.001)	‐	206 (1 RCT)	⨁◯◯◯ VERY LOW ^1^ ^,^ ^2^	
*The risk in the intervention group (and its 95% confidence interval) is based on the assumed risk in the comparison group and the **relative effect** of the intervention (and its 95% CI).**CI:** Confidence interval					

1. Risk of detection bias and attrition bias. Inadequate reporting of randomization, allocation concealment and blinding of participants and personnel.

2. Fewer than 400 participants.

###### What does the evidence say?

It is uncertain if Housing First has an effect on homelessness or housing stability when compared with abstinence‐contingent housing (very low certainty evidence).

#### Category 3B: Non‐abstinence‐contingent housing with treatment

We identified four studies that examined the effect of non‐abstinence‐contingent housing with some form of treatment (case management or day treatment) (55, 58, 65, 73). The studies were conducted in the USA. The interventions in these studies included provision of housing to participants in the treatment group that was not conditional on maintaining sobriety or attending treatment.

One study (55)compared non‐abstinence‐contingent housing in the form of group living arrangement versus independent living. Participants in both groups received housing and some form of case management (intensive case management with house staff for those assigned to group living arrangements and assertive community treatment for participants in the independent living group) (55). Participants in the intervention group could be assigned to one seven group homes which accommodated between six and ten participants and had shared amenities but separate bedrooms. The staffing patterns were similar to traditional group homes with live‐in staff. The participants had an intensive case manager they met with at least once a week. They paid 30% of their income to cover rent and utilities and were encouraged to attend activities at community mental health centres (55).

In the second study (73),participants in the intervention group were offered temporary program managed shelter as well as intensive case management. Only program participants were housed in the shelter. The research team eventually began to develop their own housing as well. Shelter stay was not contingent on treatment or sobriety; however, a small group of participants were eventually required to enter a payee arrangement due to lack of progress and using their income for drug purchases(73).

In the third study (65),the intervention was described as “parallel housing” where participants are offered housing from “mainstream” (i.e. not segregated) options that were owned and operated by community landlords or housing agencies. Participants lived independently and their tenancy was not conditional on treatment participation. The participants are also offered assertive community treatment with high intensity (low client to case manager ratio and case managers are available 24 hours every day).

In the fourth study (58),participants in the intervention group received non‐abstinence contingent housing with day treatment (58). The non‐abstinence‐contingent housing with day treatment intervention consisted of two components: housing programs in which tenancy is not conditional upon maintained sobriety and/or treatment, and day treatment. Participants were required to pay to remain in housing (but were not removed if unable to pay). The housing component was only part of treatment and available for a maximum of six months. No information was available regarding segregation of the housing or whether it was individual or group housing. Participants in the intervention group also received day treatment in the first phase of a two phase intervention. Day treatment lasted between 6.25 hours daily for the first two months of the study. Phase II of the intervention included abstinence‐contingent work therapy with minimum wage. Some participants also received aftercare. Formal treatment ended after six months.

The intervention was compared to usual services (73), non‐abstinence‐contingent housing in independent apartments (55),“integrated housing” (65), or day treatment (58).

##### 3.B.1. Non‐abstinence‐contingent housing with high intensity case management compared to usual services

One study (73) evaluated the effect of non‐abstinence‐contingent housing with high intensity case management compared to usual services on housing stability, homelessness, quality of life and psychological status.

Control group participants were offered usual services provided by the city.

###### Primary outcome: Homelessness

One study (73)evaluated the effect of non‐abstinence‐contingent housing on homelessness and housing. The rate of decline in amount of time spent living on the streets over the 24 months study period was almost twice as great for the intervention group (MD=‐54.9 (SD=36.9) that the control group (MD=‐28.2 (SD=44.5)) (t=4.18, p=0.001). Individuals in the intervention group reported more time in shelters, specifically the program provided respite housing than the control group (MD=23.1 (SD=29.27 compared to MD=2.8 (SD=15.23), p=0.001). While participants in both groups increased the time spent in community housing (including transitional settings, long‐term settings), the rate of increase was almost twice as great for the intervention group (MD=21.0 (SD=30.39)) than the control group (MD=9.9 (SD=32.34)) (t=‐2.27, p=0.025). At the final follow‐up point 38% of the intervention group were in community settings compared to 24% of the control group.

The results and quality assessments for non‐abstinence‐contingent housing with high intensity case management compared to usual services are summarized in Table 24. The complete GRADE evidence profile is presented in Appendix 8, Table 8.3.3.

**Table 24:** Summary of findings table for the effects of non‐abstinence‐contingent housing with high intensity case management vs usual services (Shern 2000)

**Patient or population**: Homeless adults with mental health problems **Setting**: USA **Intervention**: non‐abstinence‐contingent housing with high intensity case management **Comparison**: usual services					
Outcomes	Anticipated absolute effects^*^ (95% CI)		Relative effect (95% CI)	№ of participants (studies)	Quality of the evidence (GRADE)
	**Risk with usual services**	**Risk with non‐abstinence‐contingent housing with high intensity case management**			
Change in proportion of time spent homeless (streets) (Shern 2000) assessed with: self‐report follow up: 24 months	The mean change in proportion of time spent homeless (streets) was **‐28.2** percent	The mean change in proportion of time spent homeless (streets) in the intervention group was 26,7 percent lower (39,21 lower to 14,21 lower)	‐	168 (1 RCT)	⨁⨁◯◯ LOW ^1^ ^,^ ^2^
Change in proportion of time spent in shelter (Shern 2000) assessed with: self‐report follow up: 24 months	The mean change in proportion of time spent in shelter was **2.8** percent	The mean change in proportion of time spent in shelter in the intervention group was 20,3 percent higher (13,38 higher to 27,2 higher)	‐	168 (1 RCT)	⨁⨁◯◯ LOW ^1^ ^,^ ^2^
Change in proportion of time spent in community living (Shern 2000) assessed with: self‐report follow up: 24 months	The mean change in proportion of time spent in community living was **9.9** percent	The mean change in proportion of time spent in community living in the intervention group was 11,1 percent higher (1,5 higher to 20,6 higher)	‐	168 (1 RCT)	⨁⨁◯◯ LOW ^1^ ^,^ ^2^
*The risk in the intervention group (and its 95% confidence interval) is based on the assumed risk in the comparison group and the **relative effect** of the intervention (and its 95% CI).**CI:** Confidence interval;**MD:** Mean difference					

1. Inadequate reporting of allocation concealment measures and blinding.

2. Fewer than 400 participants.

###### What does the evidence say?

Non‐abstinence‐contingent housing with high intensity case management compared to usual services:

- May lead to greater decrease in proportion of time spent homeless or in shelter (low certainty evidence).
- May increase the amount of time in community living arrangements (low certainty evidence).

##### 3.B.2. Non‐abstinence‐contingent group living arrangements with high intensity case management compared to non‐abstinence‐contingent independent apartments with high intensity case management

One study (55)evaluated the effect of non‐abstinence‐contingent group living arrangements with high intensity case management (NACHG) compared to non‐abstinence‐contingent independent apartments with case management (NACHI) on housing stability, homelessness, and satisfaction with life.

Participants in the comparison group were placed in non‐abstinence‐contingent independent apartments. These apartments were efficiency units operated by the local housing authority and participants were offered voluntary weekly group meetings, but not other programming on‐site.

###### Primary outcomes: Housing stability and homelessness

The included study examined the effect of non‐abstinence‐contingent group living arrangements on the number of days homeless during the study period and number of days homeless after rehousing (55). A total of 110 participants were included in the analysis for outcomes measured at final follow‐up (18 months) (intervention N=61; comparison N=49). There was little or no difference in housing status between groups at 18 months. Participants in the intervention group reported a mean of 43 days homeless over 18 months compared to a mean of 78 days for the control group. We could not calculate the difference between groups due to inadequate reporting in the primary study.

The results and quality assessments for non‐abstinence‐contingent group living arrangements with high intensity case management compared to non‐abstinence‐contingent independent apartments with high intensity case management are summarized in Table 25. The complete GRADE evidence profile is shown in Appendix 8, Table 8.3.4.

**Table 25:** Summary of findings table for the effects of non‐abstinence‐contingent group living arrangements with high intensity case management compared to non‐abstinence‐contingent independent apartments with high intensity case management

**Patient or population**: adults who are homeless or at‐risk of becoming homeless **Setting**: USA **Intervention**: non‐abstinence‐contingent group living arrangements with high intensity case management **Comparison**: non‐abstinence‐contingent independent apartments with high intensity case management					
Outcomes	Anticipated absolute effects^*^ (95% CI)		Relative effect (95% CI)	№ of participants (studies)	Quality of the evidence (GRADE)
	**Risk with non‐abstinence‐contingent independent apartments with high intensity case management**	**Risk with non‐abstinence‐contingent group living arrangements with high intensity case management**			
Housing status ‐ housed assessed with: point in time ‐ self‐report, records of the housing facilities, and Department of mental health, weekly logs from case managers follow up: 18 months	755 per 1 000	770 per 1 000 (627 to 951)	RR 1.02 (0.83 to 1.26)	110 (1 RCT)	⨁◯◯◯ VERY LOW ^1^ ^,^ ^2^ ^,^ ^3^
Housing status ‐ not housed assessed with: point in time ‐ self‐report, records of the housing facilities, and Department of mental health, weekly logs from case managers follow up: 18 months	245 per 1 000	230 per 1 000 (118 to 451)	RR 0.94 (0.48 to 1.84)	110 (1 RCT)	⨁◯◯◯ VERY LOW ^1^ ^,^ ^2^ ^,^ ^3^
Total days homeless after rehousing assessed with: self‐report, records of the housing facilities, and Department of mental health, weekly logs from case managers follow up: 18 months	“log [+1]=.99 for 61 study participants in group homes compared with 1.8 for 51 study participants in independent apartments; t=–1.85, df=97 [unequal variances], p<.05, one‐tailed”			112 (1 RCT)	⨁⨁◯◯ LOW ^1,2^
Mean number of days homeless assessed with: self‐report, records of the housing facilities, and Department of mental health, weekly logs from case managers follow up: 18 months	Participants in the group housing intervention reported a mean of 43 days homeless over the 18 month study period compared to a mean of 78 days reported by participants in the independent housing intervention.		112 (1 RCT)	⨁⨁◯◯ LOW ^1,2^	
Number of participants who are homeless (shelter) assessed with: self‐report, records of the housing facilities, and Department of mental health, weekly logs from case managers follow up: 18 months	163 per 1 000	**131 per 1 000** (52 to 325)	**RR 0.80** (0.32 to 1.99)	110 (1 RCT)	⨁◯◯◯ VERY LOW ^1^ ^,^ ^2^ ^,^ ^3^	
Number of participants who are homeless (streets) assessed with: self‐report, records of the housing facilities and Department of mental health, weekly logs from case managers follow up: 18 months	82 per 1 000	**33 per 1 000** (7 to 171)	**RR 0.40** (0.08 to 2.10)	110 (1 RCT)	⨁◯◯◯ VERY LOW ^1^ ^,^ ^2^ ^,^ ^3^	
*The risk in the intervention group (and its 95% confidence interval) is based on the assumed risk in the comparison group and the **relative effect** of the intervention (and its 95% CI).**CI:** Confidence interval;**RR:** Risk ratio					

1. Inadequate reporting of randomization, allocation concealment and blinding.

2. Fewer than 300 participants.

3. Wide confidence interval.

###### What does the evidence say?

Non‐abstinence‐contingent group housing with high intensity case management compared to non‐abstinence‐contingent independent apartments with high intensity case management

- Maylead to fewer days homeless after being rehoused and reduce the number of days spent homeless (low certainty evidence).
- It is uncertain if the intervention has an effect on housing status at 18 months (very low certainty evidence).

##### 3.B.3. Non‐abstinence‐contingent housing with high intensity case management compared to abstinence‐contingent housing with high intensity case management

One study (65) evaluated the effect of non‐abstinence‐contingent housing with high intensity case management compared to abstinence‐contingent housing with high intensity case management on housing stability and homelessness in USA.

In this study (65),the intervention (“parallel housing”) was compared to “integrated housing”. The main difference according to the researchers is (1) housing control: integrated housing is owned or leased by the mental health provider; (2) integration within the community: parallel housing is not segregated housing units while integrated housing is; (3) conditionality: integrated housing is often linked to treatment participation, and (4): live‐in staff: integrated housing sometimes contain live‐in staff.

###### Primary outcomes: Homelessness and housing stability

The included study(65)reported proportion of time functionally homeless (a term used by primary authors to describe both time literally homeless and days in temporary or institutional settings that are preceded and followed by days homelessness) and housing stability (stable housing defined by authors as one's own apartment/house, single room occupancy with or without services, family or friends’ house on a long‐term basis, boarding house, transitional housing or a group home).

Only 121 participants took part in either the intervention (N=60) or the comparison group (N=61). Participants in both groups reduced the number of days functionally homeless from baseline to 18 months, however there was a greater change in number of days homeless among members of the comparison group over the study period (F=6.07, p<0.05, d=‐0.52). At the end of the study 68.1% of participants in the intervention group were in stable housing compared to 85.5 % of comparison group participants (F=5.99, p<0.05, d=0.51).

The results and quality assessments for non‐abstinence‐contingent housing with high intensity case management vs abstinence‐contingent housing with high intensity case management are summarized in Table 26. The complete GRADE evidence profile is shown in Appendix 8, Table 8.3.5.

**Table 26:** Summary of findings table for the effects of non‐abstinence‐contingent housing with high intensity case management vs abstinence‐contingent housing with high intensity case management (McHugo 2004)

**Patient or population**: Adults who are homeless or at risk of becoming homeless **Setting**: USA **Intervention**: Non‐abstinence‐contingent housing with high intensity case management **Comparison**: Abstinence‐contingent housing with high intensity case management					
Outcomes	Anticipated absolute effects^*^ (95% CI)		Relative effect (95% CI)	№ of participants (studies)	Quality of the evidence (GRADE)
	**Risk with Abstinence‐contingent housing with high intensity case management**	**Risk with Non‐abstinence‐contingent housing with high intensity case management**			
Proportion of days homeless assessed with: Residential Follow‐back Calendar follow up: 18 months	There was a greater change in number of days homeless among members of the comparison group over the study period (F=6.07, p<0.05, d=‐0.52).			121 (1 RCT)	⨁◯◯◯ VERY LOW ^1^ ^,^ ^2^
Proportion of days in stable housing assessed with: Residential Follow‐back Calendar follow up: 18 months	At the end of the study 68.1% of participants in the intervention group were in stable housing compared to 85.5 % of comparison group participants (F=5.99, p<0.05, d=0.51).		121 (1 RCT)	⨁◯◯◯ VERY LOW ^1^ ^,^ ^2^
*The risk in the intervention group (and its 95% confidence interval) is based on the assumed risk in the comparison group and the **relative effect** of the intervention (and its 95% CI).**CI:** Confidence interval					

1. Risk of attrition bias. Inadequate reporting of randomization, allocation concealment and blinding.

2. Fewer than 400 participants.

###### What does the evidence say?

It is uncertain whether non‐abstinence‐contingent housing with high intensity case management compared to abstinence‐contingent housing with high intensity case management has an effect on housing stability (very low certainty evidence).

##### 3.B.4. Non‐abstinence‐contingent housing with day treatment compared to day treatment

One study (58)evaluated the effects of non‐abstinence‐contingent housing with day treatment compared to day treatment only on housing stability and homelessness in the USA.

Participants in the control condition received day treatment only with no provision of housing.

###### Primary outcome: Housing stability

The included study (58)reported housing stability as the change in amount of time spent in stable housing from baseline to follow‐up. Complete data 12 months post‐baseline is only available for 116 participants (intervention N=43, comparison N=34). Although participants in both groups increased the amount of time spent in stable housing from baseline to the final follow‐up, the intervention group showed greater gains (MD=14.2 (SD=31.7) than the comparison group (MD=9.5 (SD=31.0)) (MD=4.70 [95%CI‐9.38 to 18.78]).

The results and quality assessments for non‐abstinence‐contingent housing with day treatment compared to day treatment are summarized in Table 27. The complete GRADE evidence profile is presented in Appendix 8, Table 8.3.6.

**Table 27:** Summary of findings table for the effects of non‐abstinence‐contingent housing with day treatment vs day treatment (Kertesz 2007)

**Patient or population**:homeless adults with substance dependence problems **Setting**: USA **Intervention**: non‐abstinence contingent housing with day treatment **Comparison**: day treatment					
Outcomes	Anticipated absolute effects^*^ (95% CI)		Relative effect (95% CI)	№ of participants (studies)	Quality of the evidence (GRADE)
	**Risk with day treatment**	**Risk with non‐abstinence contingent housing with day treatment**			
Changes in mean days housed in past 60 days between baseline and 12 months assessed with: Retrospective Interview for Housing, Employment, and Treatment History follow up: 12 months	The mean changes in mean days housed in past 60 days between baseline and 12 months was **9.5** days	The mean changes in mean days housed in past 60 days between baseline and 12 months in the intervention group was 4,7 days more (9,38 fewer to 18,78 more)	‐	77 (1 RCT)	⨁◯◯◯ VERY LOW ^1^ ^,^ ^2^ ^,^ ^3^
*The risk in the intervention group (and its 95% confidence interval) is based on the assumed risk in the comparison group and the **relative effect** of the intervention (and its 95% CI).**CI:** Confidence interval;**MD:** Mean difference					

1. Risk of selection bias and attrition bias. Inadequate reporting of allocation concealment methods and blinding.

2. Fewer than 300 participants.

3. Wide confidence interval.

###### What does the evidence say?

It is uncertain whether abstinence‐contingent housing with day treatment compared to day treatment only leads to more days in stable housing (very low certainty).

### Category 4: Housing vouchers with case management

#### Description of included studies

We identified four studies with five comparisons that evaluated the effect of housing vouchers with case management (27, 62, 71, 81).

Table 28 presents an overview of the populations, interventions, comparisons and outcomes in the included studies.

**Table 28:** Description of studies that evaluated effects of housing vouchers

**Study (ref); country**	**Population** **(N, description)**	**Intervention, follow‐up (FU) in months (mos), N**	**Comparison, N**	**Primary outcome**
Hurlburt 1996(27), USA	N=362 at‐risk of or homeless, mental illness	Section 8 housing vouchers with case management FU: 18 mos N=181	Case management N=181	Stably housed Homeless
Levitt 2013(62), USA	N=330 families with at least one child, in shelters	Intensive housing placement and case management FU: 12 mos N=138	Usual services N=192	Time to exit /return to shelter Total days spent in shelter
Rosenheck 2003(71), USA	N=460 homeless veterans, mental illnes and/or substance dependence	Section 8 housing vouchers with case management FU: 36 mos N= 182	Usual services N= 188 Intensive case management N= 90	Stably housed Homeless
Wolitski 2010(81), USA	N=630 homeless/unstably housed people living with HIV/AIDS	Section 8 housing vouchers with case management FU: 18 mos N=315	Usual services N=315	Stably housed Proportion homeless > 1 night

#### Description of the intervention

Housing vouchers for the purpose of this review is interventions where the housing component is limited to the provision of financial assistance for housing of the participants choosing. Case management is described above (Category 1).

In the first study (27), 362 participants were assigned to one of four groups: comprehensive case management or traditional case management with or without HUD Section 8 housing certificates (financial assistance). A preliminary analysis of the between group differences showed no correlation between the case management model and housing outcomes, so further analysis was based on the Section 8 housing certificate condition. Therefore the groups were analyzed as following: Comprehensive or traditional case management with HUD Section 8 housing certificates compared to comprehensive or traditional case management without HUD Section 8 housing certificates. Participants in each condition received a range of case management services varying in intensity (time between contact with case managers), case load of case managers (1:22 up to 1:40), and availability (comprehensive case managers were constantly available). The HUD Section 8 housing certificate is a program allowing holders to pay a fixed 30% of their adjusted income for a private rental unit of their choosing. There are no conditions on the tenancy except for that the housing must meet the quality standards of the US Department of Housing and Urban Development and the rent for the unit must be equal or less than fair market rent for the area. The participants in this program received a tailored version of the certificate program with more flexible rules (for example keeping appointments) and with support from housing specialists who assisted with the application process and were sensitive to limitations imposed by severe mental illness.

In the second study (62),participants in the intervention group were enrolled in the *Home to Stay* program. The Home to Stay model was designed to quickly put families into housing and maintain the housing using a time‐limited financial subsidy and temporary support services. At the beginning of the study participants could access 1 year Advantage housing subsidies (three types of locally funded subsidies intended for families with children, clients with disability payments, or employed clients). After three months, clients (participants) were required to contribute 30% of their monthly income and eligibility was restricted to employed (or receiving federal disability payments) adults with children. At the one year mark these subsidies were no longer available for new families and two years after the study began the monthly payments were terminated for all recipients. Initial services in the Home to Stay program was to help families’ secure permanent housing and exist shelter quickly. After they were placed in housing, there was a focus on obtaining employment (income) equal to double the family's rent obligation and/or obtaining a permanent housing subsidy. Participants in this group also received fairly intensive case management services while in shelter. The intervention condition was different than the usual services condition specifically with respect to more frequent case manager contact, smaller caseloads, flexible scheduling, integrated help with financial literacy and continuing the services from shelter into housing.

In the third study (71) the US Department of Housing and Urban Development allocated funds for 1000 vouchers for a program providing housing and case management for literally homeless veterans with mental illness or substance dependence. These participants were offered priority access to the Section 8 housing vouchers (difference between 30% of their adjusted income and the lesser of Fair Market Rent or the unit rent). Case managers put the veterans in contact with the local housing voucher and helped them to locate an apartment, negotiate the lease, furnish and move into the apartment. The case management component was a modified assertive community treatment model (larger caseloads and encouragement of clients to use other Veteran Affairs health services). The intervention was compared to usual services and case management. Participants in the comparison conditions received standard Veteran Affairs homeless services, including short‐term brokered case management, or intensive case management.

In the fourth study (81), participants living with HIV/AIDS were provided with long‐term rental housing assistance. The amount was determined by The Department of Housing and Urban Development (HUD) annually for each metropolitan area. Each person receiving rental assistance was required to pay 30% of this monthly adjusted income. Study‐funded housing referral specialists assisted with finding housing and negotiating leases and participants received referrals to other supportive services.

The interventions were compared to usual services (62, 71, 81), case management (27), or high intensity case management (71).

##### 4.1. Housing vouchers with case management compared to usual services

Two of the three studies that compared housing vouchers with case management to usual services (62, 71, 81)included multiple cities (71, 81). One study included families (62) and one study included adults living with HIV/AIDS.

###### Primary outcomes: Time to exit shelter, stable housing, homelessness

Three of the included studies evaluated the effect of housing vouchers compared to usual services on housing stability and homelessness(62, 71, 81). The studies measure and report these outcomes in such different ways that we are unable to pool results. The following is a narrative summary of the results from the three studies.

In the first study (62), the authors included work‐based subsidies as a covariate in all analyses of differences between the intervention group (N=138) and the control group (N=192). A survival analysis using Cox regression of time to first exist from shelter (at least 30 days away from shelter) shows that the intervention group experienced fewer days to exit shelter (x^2^_1_ = 6.068, 95%CI = 0.589 to 0.942; proportional hazards assumption not violated). The authors also report the time to return to shelter (overnight stay) for those that did return (N=298) and that the intervention group reported longer time to return to shelter than the control group (x^2^_1_ = 6.524, 95% CI = 0.379 to 0.880; proportional hazards assumption not violated).

In the second study (71), data for 182 participants in the intervention group and 188 participants in the control group were reported related to number of days housed during the 90 days prior to each follow‐up. We report the longest follow‐up at 36 months. The intervention group reported more days housed (M=59.39) compared to the control group (M=47.60) (t=4.88, p<0.001). The intervention group also reported fewer days homeless (M=13.05) than the control group (M=20.45) (t=3.56, p<0.001).

In the third study (81), the authors reported the number of participants in their own home, the number living temporarily with others or in transitional settings, or the number with one or more nights homeless during the 90 days prior to follow‐up for the intervention group (N=315) and the control group (N=315). At the 18 month follow‐up interview there were more people from the housing vouchers group living in their own home (82.48) than the control group (50.58), fewer people in the housing vouchers group living temporarily with others or in transitional settings (14.96) than the control group (44.40) and half as many who reported being homeless at least once during the previous 90 days (2.55) than the control group (5.02). It is not possible to calculate the effect size due to lack of information reported in the results from the primary study.

The results and quality assessments for housing vouchers with case management compared to usual services are summarized in Tables 29. The complete GRADE evidence profile is shown in Appendix 8, Table 8.4.1.

**Table 29:** Summary of findings table for the effects of housing vouchers with case management vs usual services (Levitt 2013, Wolitski 2010, Rosenheck 2003)

**Patient or population**: Adults or families who are homeless or at risk of becoming homeless **Setting**: USA **Intervention**: Housing vouchers with case management **Comparison**: Usual services					
Outcomes	Anticipated absolute effects^*^ (95% CI)		Relative effect (95% CI)	№ of participants (studies)	Quality of the evidence (GRADE)
	**Risk with usual services**	**Risk with housing vouchers with case management**			
Time to first exit from shelter assessed with: Not reported follow up: 12 months	The intervention group reported fewer days to exit shelter than the control group x^2^1 = 6.068, 95%CI = 0.589, 0.942		‐	330 (1 RCT)	⨁⨁◯◯ LOW ^1^ ^,^ ^2^
Time to return to shelter assessed with: Not reported follow up: 12 months	The intervention group reported longer time to return to shelter than the control group x^2^1 = 6.524, 95% CI = 0.379, 0.880		‐	330 (1 RCT)	⨁⨁◯◯ LOW ^1^ ^,^ ^2^
Number of days housed during 90 days prior to follow‐up assessed with: Not reported follow up: 36 months	Rosenheck 2003 (Intervention N=182, Control N=188) Intervention: 59.39 days housed, Control: 47.60 days housed. t=4.88, p<0.001		‐	460 (1 RCT)	⨁⨁◯◯ LOW ^1^ ^,^ ^2^
Number of days homeless during 90 days prior to follow‐up assessed with: Not reported follow up: 36 months	(Intervention N=182, Control N=188) Intervention: 13.05 days homeless, Control 20.45 days homeless, t=3.56, p<0.001.		‐	460 (1 RCT)	⨁⨁◯◯ LOW ^1^ ^,^ ^2^
Proportion of participants who were in their own home at follow‐up assessed with: Not reported follow up: range 18 months	More participants from the Intervention group reported being in their own home during the previous 90 days (82.48%; n=315) than in control group (50.58%; n=315)			630 (1 RCT)	⨁⨁◯◯ LOW ^1^ ^,^ ^2^
Proportion of participants who were homeless one or more nights during the 90 days prior to follow‐up assessed with: Not reported follow up: 18 months	A greater proportion of participants in the intervention group reported living in transitional settings or temporarily living with others (14.96%; n=315) compared to the control group (44.40%; n=315).			630 (1 RCT)	⨁⨁◯◯ LOW ^1^ ^,^ ^2^
Proportion of participants who were homeless one or more nights during the 90 days prior to follow‐up assessed with: Not reported follow up: 18 months	A greater proportion of participants in the intervention group reported living in transitional settings or temporarily living with others (14.96%; n=315) compared to the control group (44.40%; n=315).			630 (1 RCT)	⨁⨁◯◯ LOW ^1^ ^,^ ^2^
*The risk in the intervention group (and its 95% confidence interval) is based on the assumed risk in the comparison group and the **relative effect** of the intervention (and its 95% CI).**CI:** Confidence interval					

1. Risk of performance bias and detection bias. Inadequate randomization methods.

2. One small study.

###### What does the evidence say?

Housing vouchers with case management compared to usual services for homeless families:

- May reduce the number of days it takes to leave tempoary shelters and increase the number of days before returning to temporary shelters (low certainty evidence).
- May increase the number of days in stable housing and reduce the number of days spent homeless (low certainty evidence).
- May increase the proportion of people living in their own house, reduce the proportion of people who experience at least one night of homelessness and reduce the proportion of people who live in transitional settings at 18 month follow‐up (low certainty evidence).

##### 4.2. Housing vouchers with case management compared to case management only

We identified two studies that examined the effect of housing vouchers with case management compared to case management (27). The case management component of the intervention varied in intensity. In one study, participants received either comprehensive (high intensity) case management or traditional (low intensity case management) in addition to the housing vouchers while the control group also received one of the two types of case management. Participants in the second study received high intensity case management. We have decided to combine the two studies under a broader heading of case management.

###### Primary outcomes

The first study(27)reported the type of housing maintained by participants, the number in stable housing and how many participants transitioned early (first six months of study) into independent or community housing (defined in this study as family or friend's home or a boarding/halfway house). Approximately twice as many participants in the intervention group maintained independent housing at the 24 month follow‐up (104/181) compared to the comparison group (55/181) (RR=1.89 [95%CI 1.47 to 2.44]). Approximately four times as many participants in the comparison group (44/181) compared to the intervention group (11/181) reported living in community housing at 24 months (RR=0.25 [95% CI 0.13 to 0.47]). More participants in the comparison group were recorded as living in variable housing (unstable, institution, or disengaged from study) (82/181) compared to participants in the intervention group (66/181) (RR=0.80 [95%CI 0.63 to 1.03]). Finally, the authors also measured the proportion of participants who transitioned early into independent and community housing (the first 6 months). The authors reported that participants with housing vouchers stabilized in independent housing faster than participants in the comparison condition and were 8.4 times more likely to obtain independent housing in the first six months of the study (91/115 intervention group participants compared to 25/99 comparison group participants). On the contrary, the comparison group was 3.4 times more likely to obtain other types of community housing in the first six months (28/99 comparison group participants compared to 4/115 intervention group participants).

Results from the second study (71) show that the intervention group reported more (16.9%) days housed (M=59.39) compared to the control group (M=50.81) (t=2.90, <p=0.004) at 36 months. The intervention group also reported fewer days homeless (M=13.05) than the control group (M=20.33) (t=2.87; p=0.004) at 36 months.

The results and quality assessments for housing vouchers with case management compared to case management only are summarized in Table 30, and the complete GRADE evidence profile is shown in Appendix 8, Table 8.4.2.

**Table 30:** Summary of findings table for the effects of housing vouchers with case management vs case management only (Hurlburt 1996, Rosenheck 2003)

**Patient or population**: adults with mental illness **Setting**: USA illness **Intervention**: housing vouchers with case management **Comparison**: case management					
Outcomes	Anticipated absolute effects^*^ (95% CI)		Relative effect (95% CI)	№ of participants (studies)	Quality of the evidence (GRADE)
	**Risk with case management**	**Risk with housing vouchers with case managment**			
Number of participants in independent housing assessed with: case manager records follow up: 24 months	304 per 1 000	**574 per 1 000** (447 to 741)	**RR 1.89** (1.47 to 2.44)	362 (1 RCT)	⨁⨁◯◯ LOW^1^ ^,^ ^3^
Number of participants living in community housing assessed with: case manager records follow up: 24 months	243 per 1 000	**61 per 1 000** (32 to 114)	**RR 0.25** (0.13 to 0.47)	362 (1 RCT)	⨁⨁◯◯ LOW^1^ ^,^ ^3^
Number of participants living in variable housing situations assessed with: case manager records follow up: 24 months	453 per 1 000	**362 per 1 000** (285 to 467)	**RR 0.80** (0.63 to 1.03)	362 (1 RCT)	⨁⨁◯◯ LOW^1^ ^,^ ^3^
Number of days in stable housing assessed with: self‐report follow up: 36 months	Participants in the intervention group reported more days in stable housing than the control group (M=59.39 vs M=50.81), t=2.90, p<0.004		**‐**	272 (1 RCT)	⨁⨁◯◯ LOW^2^ ^,^ ^3^
Number of days spent homeless assessed with: self‐report follow up: 36 months	Participants in the intervention group reported fewer days homeless than the control group (M=13.04 s M=20‐33), t=2.87, p=0.004	**‐**	272 (1 RCT)	⨁⨁◯◯ LOW^2^ ^,^ ^3^
*The risk in the intervention group (and its 95% confidence interval) is based on the assumed risk in the comparison group and the **relative effect** of the intervention (and its 95% CI).**CI:** Confidence interval;**RR:** Risk ratio					

1. Inadequate reporting of methods.

2. Risk of performance bias. Inadequate reporting of methods for blinding of outcome assessors.

3. Fewer than 400 participants.

###### What does the evidence say?

Housing vouchers with case management compared to case management only

- May increase the number of people living in independent housing and reduce the number of people living in community housing (low certainty evidence).
- May increases the number of days spent in stable housing and reduces the number of days spent homeless (low certainty evidence).
- May lead to no difference in the number of people living variable housing situations (low certainty evidence).

### Category 5: Residential treatment

#### Description of included studies

We identified two studies that evaluated the effect of residential treatment (49, 63). Both studies were conducted in the USA. Table 31 presents an overview of the populations, interventions, comparisons and outcomes in the included studies.

**Table 31:** Description of studies that evaluated effects of residential treatment

**Study (ref); country**	**Population** **(N, description)**	**Intervention, follow‐up (FU) in months (mos), N**	**Comparison, N**	**Primary outcome**
Conrad 1998(49), USA	N=358 homeless male veterans with substance dependence and mental illness	Case managed residential care for veterans FU: 24 mos N=178	Standard inpatient treatment and discharge care N=180	Days homeless
Lipton 1988(63), USA	N=52 (49 analyzed) homeless adults with mental illness	Case management and supportive housing FU: 12 mos N=26	Standard post‐discharge services N=23	Days housed Days homeless

#### Description of the intervention

The two studies that evaluated the effect of residential care on homelessness and housing stability (49, 63). The interventions in the included studies are different due to the different populations which they target. In the first study (49), the intervention was divided into two phases: the residential phase (0‐6 months) and the community phase (7‐12 months). During the residential phase participants received case management services, treatment planning and service referral, counselling, and material assistance. During the community phase participants were placed in community living with continued case management and cognitive behavioural therapy and self‐help groups such as Alcoholics and Narcotics Anonymous. Participants were followed up to 24 months, even though the active part of the intervention only lasted 12 months.

In the second study (63), participants were placed in a non‐profit supportive housing program which used single rooms in an urban hotel. This permanent residence provided services such as a furnished room, case management, coordination of public assistance, medication and money management, meals, therapy and referrals to appropriate services. Both the treatment and the longest follow‐up time was 12 months.

##### 5.1. Residential treatment compared to usual services

We found two studies that evaluated the effect of residential care compared to usual services (49, 63).

While both studies compared the intervention to usual services, these services differed due to the different target populations in the studies. In the first study (49), the usual services was inpatient treatment in hospital wards for two to three weeks and included substance abuse education, therapy, self‐help services, medical care, material assistance and referral to appropriate services. Customary community care was provided up to 12 months and included services as needed, half‐way houses and mental health treatment for post‐traumatic stress disorder.

In the second study (63), participants in the usual services condition received standard post‐discharge care, of which one quarter of participants refused. No further information was provided on what this care entailed.

Due to the difference in population, intervention and comparison group characteristics we have not pooled the results. We present a narrative summary of the results from each study below.

###### Primary outcomes: Homelessness and stable housing

Both of the included studies reported the proportion of nights spent homeless ((49, 63). In the first study (49), participants in the intervention group (N=178) reported less homelessness than the control group (N=180) during the 60 days prior to the 24 month follow‐up interview (11% compared to 2% for the control group) (Random effects regression estimate=0.104 (SE=0.037), Z=2.846, p=0.004). In the second study (63), participants in the intervention group reported less time homeless over the 12 month study period (6% SD=22 compared to 46% SD=51; t^2^=2.62, df=31, p=0.019). Furthermore, the authors report that during the study period, participants in the intervention group had a 13% chance of having 30 or more consecutive nights homeless compared to 39% for the control group (x^2^=87.46, df=1, p=0.01).

The first study (63) also reported the proportion of time participants reported being housed. Participants in the intervention group (N=26; 79%, SD=26) reported being in permanent housing more than twice as much as the control group (N=23; 33% SD=36) during the study year (t^2^=4.32, df=32, p=0.0001). Furthermore more than twice as many participants from the intervention group reported being in permanent housing at the 12 month follow‐up interview (69% compared to 30%). Data was not reported for number of nights spent in shelter (63).

The results and quality assessments for residential treatment with case management vs usual services is summarized in Table 32. The complete GRADE evidence profile is shown in Appendix 8, Table 8.5.1.

**Table 32:** Summary of findings table for the effects of residential treatment with case management vs usual services (Conrad 1998, Lipton 1988)

**Patient or population**: adults with mental illness and/or substance abuse issues **Setting**: USA **Intervention**: residential treatment with case management **Comparison**: usual services					
Outcomes	Anticipated absolute effects^*^ (95% CI)		Relative effect (95% CI)	№ of participants (studies)	Quality of the evidence (GRADE)
	**Risk with Usual services**	**Risk with Residential treatment with case management**			
Proportion of nights homeless assessed with: Personal History Form follow up: range 12 months to 24 months	Participants in the intervention group in both studies reported less homelessness than participants in the control group.		‐	407 (2 RCTs)	⨁⨁◯◯ LOW ^1^
Proportion of time in stable housing assessed with: Unclear follow up: 12 months	Participants in the intervention group (N=26; 79%, SD=26) reported being in permanent housing more than twice as much as the control group (N=23; 33% SD=36) during the study year (t2=4.32, df=32, p=0.0001).		‐	49 (1 RCT)	⨁⨁◯◯ LOW ^2^ ^,^ ^3^
Number of participants stably housed at follow‐up assessed with: Unclear follow up: 12 months	More than twice as many participants from the intervention group reported being in permanent housing at the 12 month follow‐up interview (69% compared to 30%).		‐	49 (1 RCT)	⨁⨁◯◯ LOW ^2^ ^,^ ^3^
*The risk in the intervention group (and its 95% confidence interval) is based on the assumed risk in the comparison group and the **relative effect** of the intervention (and its 95% CI).**CI:** Confidence interval					

1. Risk of attrition bias, reporting bias in one study. Inadequate reporting of methods in both studies.

2. Inadequate reporting of methods.

3. Fewer than 400 participants.

###### What does the evidence say?

Residential treatment with case management compared to usual services:

- May reduce the proportion of nights spent homeless (low certainty evidence).
- May increase the proportion of time spent in stable housing (low certainty evidence).
- May increase the number of participants who are in stable housing after one year (low certainty evidence).

## Discussion

In this systematic review we aimed to summarize empirical research assessing the effect of housing programs and case management on improving housing stability and reducing homelessness for individuals who are homeless, or are at‐risk of becoming homeless. We included 43 randomized controlled trials with a total of approximately 10,570 participants. The majority of the studies included adult participants with mental illness and/or substance abuse. All of the studies were assessed as having high risk of bias. Five main groups of interventions were identified: case management, abstinence‐contingent housing, non‐abstinence‐contingent housing, housing vouchers, and residential treatment. The interventions were compared to usual services or another intervention. In practice, this means that all participants received or had access to some type of service.

Within these groups, a total of 28 comparisons assessed housing stability and/or homelessness. In addition, many of the included studies also addressed secondary outcomes such as employment, physical or mental health, quality of life, social support networks, substance abuse and criminal activity.

Overall, the findings suggest that case management and housing programs are consistently more effective than usual services in reducing homelessness and increasing the amount of time spent in stable housing. It is difficult to conclude whether interventions which combine housing with case management are more effective than case management only since only one study included that comparison and this evidence was assessed as having very low certainty.

### Discussion of main results

We included 24 studies that evaluated the effect of case management on housing stability and/or homelessness. Eligibility criteria in the majority of the studies included homeless adults or those at‐risk of becoming homeless, with mental illness and/or substance abuse issues. Three studies included other populations (disadvantaged youth, recently released criminal offenders, and homeless adults with families). Case management is a broad term and includes an array of interventions. For the purpose of this review, we therefore categorized them into either high intensity, where the intervention was described as assertive community treatment or intensive case management, or low intensity, where the intensity was not specified, or where case managers met with participants less than weekly. These interventions were compared with either usual services, another type of case management (of varying intensity), or an intervention that included neither a case management nor a housing component (for example motivational enhancement therapy). Importantly, even comparison group participants who received usual services were offered some type of service, support or treatment. This means that all interventions were, in reality, compared to an active comparison group to some degree.

#### Case management

High intensity case management probably reduces by almost one‐third the number of individuals with mental illness and/or substance abuse problems who report being homeless, and increases by about 25% the number in stable housing 12‐18 months after services are initiated compared to individuals who are offered usual services. It probably leads to little or no difference in the number of people (with mental illness and/or substance abuse, or recently released criminal offenders) who experience some homelessness during a two year period. Furthermore, high intensity case management may lead to a lower mean number of days spent homeless compared to usual services for both adults with mental illness and/or substance abuse problems and homeless adults with families. Taken together these findings suggest that although individuals who receive high intensity case management are probably just as likely to experience some homelessness, overall it may be fewer days total. For this reason, at any given point in time (e.g. follow‐up interview), individuals who receive high intensity case management are less likely to be homeless and more likely to be in stable housing, compared to individuals who are offered usual services.

When compared to low intensity case management, high intensity case management may lead to little or no difference in the number of days spent in stable housing or the number of participants who experience some homelessness.

For many of the outcomes, both the quantity and quality of available evidence was too limited to draw conclusions. Many of these outcomes are related to mean number of days in stable housing or homeless, longest residence, number of moves, number of people who report not moving, and the number of days in better or worse housing.

In summary, it appears as though high intensity case management is better than usual services, but not better than low intensity case management in improving housing stability and reducing homelessness for adults with mental illness and/or substance abuse problems and homeless adults with families. This is perhaps not surprising given the variation in how the case management interventions are designed and implemented. It may indicate that in practice there is not much difference with respect to intensity, for example, between high intensity (ACT and ICM) and low intensity case management interventions. Alternatively, it may suggest that having at least one individual (case manager) guiding and supporting a participant through the number of disjointed services may be more important than the degree of intensity of the intervention.

For the two comparisons which included young people or youth, the results showed that case management (high or low) compared to usual services or another intervention with no housing or case management component may lead to little or no difference in number of days spent homeless, the number who were homeless at follow‐up or the number of moves experienced during a 12‐month period. These results differ slightly from the comparisons which only included adults. Chamberlain and MacKenzie (2004) described the stages which youth go through before they are identified as homeless and argued for prevention and interventions which target these stages: 1) at‐risk as identified by school counsellors, 2) runaways, 3) no longer belonging to the family, and 4) transition to chronicity where there are longer periods of homelessness(84).Chamberlain and MacKenzie (2004) argued that in the later stages, interventions with community placement components are necessary. Participants from both of the included studies (comparing high or low intensity case management to usual services or another intervention with no housing or case management component) included youth in the last stage (homeless or history of homelessness). In one study, the case management condition did not seem to include the community placement component, while in the other study, the comparison groups appeared to include equal or greater community placement components (CRA and MET). This could explain why there were no differences between the groups on housing stability or homelessness for this particular population. Alternatively, youth are often considered much more vulnerable and may just require more intensive case management services than even the high intensity case management models such as ICM and ACT which are intended for adults, currently provide.

Critical time intervention (CTI) may be more effective than usual services at improving housing stability and reducing homelessness for adults with mental illness. Even though individuals who receive CTI may be just as likely to experience *some* homelessness as individuals who receive usual services, they may spend fewer days homeless in total, and take half as long to leave shelter for stable or community housing.

Our findings are largely consistent with those from other reviews of case management for homeless populations(18, 20, 28). Coldwell and Bender (2007) also found that assertive community treatment reduced homelessness among populations with severe mental illness (28). Nelson and colleagues (2007) also found ACT and ICM to be superior to standard care for achieving housing stability among individuals with mental illness (20). Most recently de Vet and colleagues concluded that case management has a positive effect on homeless populations compared to standard care (18). Slesnick and colleagues (2009) summarized the research on youth homelessness and also concluded that comprehensive interventions that address youth and families, rather than single‐issue interventions (such as case management), may be more successful with this particular population (30). This review included a wide variety of study designs and provided an overview of the studies rather than a synthesis of results.

However, our review differs from previous systematic reviews in five main ways: 1) we have included only randomized controlled trials, which are considered the best method for examining the effectiveness of an intervention; 2) we have only included studies which follow participants for at least one year; 3) we have grouped interventions according to low and high intensity and thus we have results for a larger group of interventions rather than individual models of case management (e.g. ACT, ICM); 4) we have pooled the results (continuous and dichotomous separately) where possible which has allowed us to look at the evidence across studies and not conclude based on small sample sizes from individual studies, and; 5) we have applied GRADE to the outcomes and thus provided a more concrete indication of our certainty in the evidence.

#### Abstinence‐contingent housing

Abstinence‐contingent housing combined with day treatment may reduce the number of days spent homeless when compared with usual services; however, we are uncertain of its effects on housing stability and homelessness when compared with other interventions due to very low certainty evidence. Furthermore, we are uncertain of the effects of abstinence‐contingent housing with case management.

#### Non‐abstinence‐contingent housing

We identified two categories of non‐abstinence‐contingent housing: Housing First, and other programs that did not explicitly use the Housing First model.

The Housing First model probably improves housing stability and reduces homelessness compared to usual services. There are no previous systematic reviews that we are aware of that have specifically looked at the effects of Housing First on housing and homelessness. The results from this review indicate 1) that Housing First probably reduces homelessness and increases the number of days in stable housing among adults with mental or chronic medical illness; and 2) may double the number of participants placed in permanent housing within two years.

We are uncertain of the effects of Housing First when compared with abstinence‐contingent housing due to very low certainty evidence. However, there are no indications that Housing First is less effective in reducing homelessness or improving housing stability.

The results discussed here are from studies conducted in the USA and in Canada. The consistency of the above results, which include multiple settings with diverse social welfare, political and economic settings, supports the idea that Housing First can work in a variety of settings.

Non‐abstinence contingent housing programs that did not explicitly employ the Housing First model may also reduce the amount of time spent homeless or living in shelters and increase the amount of time in stable housing compared to usual services. Furthermore, group homes where tenancy is not contingent on treatment adherence or sobriety may reduce the amount of time homeless compared to independent apartments with similar non‐abstinence contingent tenancy.

However, when compared with abstinence‐contingent housing (integrated housing), non‐abstinence contingent housing may be less effective at reducing homelessness and improving housing stability.

We are uncertain of the effect of non‐abstinence contingent housing combined with day treatment compared with day treatment only due to very low certainty evidence.

#### Housing vouchers

All of the included studies were conducted in the USA and thus used Section 8 Housing Vouchers provided by the Department of Housing and Urban Development. These housing vouchers combined with case management are probably more effective in reducing homelessness and improving the amount of time in stable housing than usual services or case management alone for adults with mental illness or HIV. Housing vouchers may help homeless families leave temporary shelters more quickly and stay out of shelters for longer periods of time.

#### Residential treatment with case management

Residential treatment with case management for adults with mental illness and/or substance abuse may be more effective at reducing the amount of time people spend homeless after leaving treatment, and increase both the amount of time spent in stable housing and the proportion of participants who are in stable housing one year after beginning treatment.

### Overall completeness and applicability of the evidence

#### Completeness of the evidence

The identified studies include a fairly good representation of the typical populations which struggle with housing stability (adults with mental illness and/or substance abuse) as well as some relatively smaller portions of the homeless population (families, youth, recently released criminal offenders). The included studies also examined, altogether, all of the interventions which were identified in the protocol for the project. They were compared to both usual services and other interventions. As specified in the inclusion criteria, all of the studies addressed the primary outcomes (homelessness and housing stability) and many of the studies also examined secondary outcomes.

There are, however, three legitimate concerns regarding applicability of the review findings to other contexts. Firstly, usual services may differ substantially from context to context (e.g. between Denmark and the USA, or between states within the USA). Relatively better usual services in a given context may reduce the difference in outcomes between intervention and usual services groups. Secondly, there is a concern regarding the definition of homelessness. In some countries, “homeless” includes “literally homeless,” or people with no shelter (living on the streets). In contexts where homelessness is defined more broadly (anyone in transitional or unstable housing) there may be less of a difference between intervention and control groups for some outcomes.

### Quality of the evidence

Although all 43 of the included studies were randomized controlled trials, all studies with the exception of one were assessed as having high risk of bias. This high risk of bias is due to: risk of selection bias, particularly poor randomization (N=4) or poor allocation concealment procedures (N=4); performance bias (N=21); detection bias (N=12); attrition bias (N=15); or reporting bias (N=2). In 12 studies other risks of bias were also identified, including addition of new participants halfway through the study period without providing details regarding demographics or background, self‐selection of participants during pre‐treatment assessment period or discretionary approval of individuals’ participation in the study by the implementing institutions, participants moving between intervention and control conditions, and treatment diffusion, introduction of new policies which resulted in media attention or impacted “usual services” during the study period, and varying degrees of treatment fidelity as discussed by the primary authors. However, the most common issue across studies was poor reporting of methods, including inadequate reporting of randomization, allocation and blinding methods. In many studies it was not possible to ascertain whether attempts were made to blind participants, personnel or outcome assessors to the assigned intervention condition. It can be assumed, due to the nature of the intervention, that blinding was neither possible nor attempted in most of these studies, and thus we often interpreted unclear reporting for these domains as high risk of bias. We attempted to assess risk of bias separately for subjective and objective outcomes due to the lack of or unclear blinding of participants and personnel, as performance bias is more likely to influence subjective outcomes than objective outcomes. However, there were very few objective outcomes included in the study. When number of days spent homeless or in different housing situations was reported, it was either explicitly indicated that these were self‐report measures using an interview form, or the data collection methods were not described (i.e. no mention of use of administrative records) and we assumed self‐report measures were employed. Some of the secondary outcomes reported in the individual studies used objective measures such as urine analysis; however, we have not graded evidence for any secondary outcomes.

### Strengths and limitations of this review

This review has numerous strengths. Firstly, the findings of this review are based on a rigorous and systematic search of the published and grey literature. Furthermore, identification and selection of relevant studies and publications were carried out by at least two reviewers and based on a priori defined criteria. This was also the case for data extraction, appraisal of the risk of bias in the included studies and grading of the evidence for all outcomes. The published protocol is available at kunnskapssenteret.no. Secondly, we only included randomized controlled trials, thereby including evidence from only the most appropriate study design to answer this review of effectiveness. Thirdly, many of the included studies presented enough data on the difference between groups so that it was possible to statistically estimate the effect of case management or housing programs on housing stability and homelessness. Fourthly, by appraising the methodological quality of the included studies and grading the evidence, we are able to point out clear areas where future research can be improved in terms of design, conduct and reporting. Finally, by including both housing programs and case management interventions, we have provided a comprehensive overview of what is known about the effect of most types of interventions available to prevent or reduce homelessness among homeless or at‐risk groups and a comparison of their relative effectiveness where possible.

However, this review is not without limitations. Firstly, the complex nature of the interventions included in this review have three important consequences: 1) we may have missed relevant interventions in the literature search that were labelled as something else butincluded many or all of the same components of the included interventions; 2) we have grouped interventions together in an attempt to provide the end user with a more clear overview of types of interventions that work – this unavoidably leads to less detail regarding individual interventions, and; 3) the included interventions are likely to have varied greatly in how they were implemented, between study sites and across studies, even where they were reported as having followed a specific model (e.g. Housing First). We have not reported treatment fidelity for the included programs. Treatment fidelity was not systematically reported in the included studies, and was thus left out of our analysis. Secondly, due to archiving problems, we are unable to provide a complete list of reasons for exclusion for studies excluded after being read in full‐text in the first search. Thirdly, for resource reasons, we have not attempted to synthesize, narratively or through meta‐analysis, results for secondary outcomes. Finally, we did not extract data on, or include, cost‐effectiveness data, which is important in making decisions on implementing such large social interventions, nor did we include qualitative research, which is used to examine participants’ perceptions, preferences and/or experiences with interventions.

## Conclusion

In this comprehensive systematic review of 43 randomized controlled trials, we aimed at determining the effect of interventions to improve residential stability and reduce homelessness. We found that housing programs and case management interventions appear to improve housing stability and reduce homelessness compared to usual services. There was no evidence that housing programs or case management resulted in poorer outcomes for homeless or at‐risk individuals than usual services.

### Research gaps

There is a great deal of research available on interventions to improve housing stability and reduce homelessness, as demonstrated by the large number of randomized controlled trials included in this review (and the large number of quasi‐experimental studies excluded). However, the majority of the existing research has been judged to have high risk of bias, mostly due to poor reporting of methods, and lack of blinding of participants, personnel and outcome assessors. Although it is impossible to blind personnel and participants due to the nature of the interventions, the outcome assessors could be blinded. Furthermore, there has been no clear improvement in reporting between the year the first included study was published (1992) and 2015 (the most recent publication). Specifically, details are lacking regarding comparison group conditions, and the reporting of effect estimates within primary studies isinadequate.

Aside from a general need for better conducted and reported studies, there are specific gaps in the research:

- Case management for specific sub‐groups, specifically families and disadvantaged youth
- Abstinence‐contingent housing with case management or day treatment
- Non‐abstinence contingent housing, specifically different living arrangements (group vs independent living)
- Housing First compared to interventions other than usual services (e.g. abstinence‐contingent housing, case management only, housing vouchers)
- All interventions from contexts other than the USA

## Information about this review

### Review authors

**Lead review author**

The lead author is the person who develops and co‐ordinates the review team, discusses and assigns roles for individual members of the review team, liaises with the editorial base and takes responsibility for the on‐going updates of the review.

**Table:** 

**Name:**	**Heather Menzies Munthe‐Kaas**
Title:	Researcher
Affiliation:	Norwegian Institute of Public Health
Address:	PO Box 4404 Nydalen
City, State, Province or County:	Oslo
Post code:	0403 Oslo
Country:	Norway
Phone:	+47 40293266
Mobile:	+47 40293266
Email:	Heather.munthe‐kaas@fhi.no
**Co‐author(s)** (There should be at least one co‐author)	
**Name:**	**Rigmor Berg**
Title:	Department director, Professor
Affiliation:	Norwegian Institute of Public Health
Address:	PO Box 4404 Nydalen
City, State, Province or County:	Oslo
Post code:	0403 Oslo
Country:	Norway
Phone:	+47 90802240
Mobile:	+47 90802240
Email:	Rigmor.berg@fhi.no

**Name:**	**Nora Blaasvær**
Title:	Researcher
Affiliation:	Norwegian Institute of Public Health
Address:	PO Box 4404 Nydalen
City, State, Province or County:	Oslo
Post code:	0403 Oslo
Country:	Norway
Phone:	+47 90802240
Mobile:	+47 90802240
Email:	Nora.blaasvaer@fhi.no

### Roles and responsibilities

Author1 was responsible for the writing of this report. Author 2 and Author 3 contributed to the process of including and excluding studies, critical appraisal, and commenting on the manuscript. Information Retrieval Specialists Ingvild Kirkehei and Lien Nguyen were responsible for the searches conducted in 2014 and 2016 respectively. We would like to acknowledge Sissel Johansen and Karianne Thune Hammerstrøm for their assistance in screening studies from the 2014 search.

### Sources of support

Norwegian Institute of Public Health. This review was commissioned by the Norwegian State Housing Bank.

### Declarations of interest

The authors have no vested interest in the outcomes of this review, nor any incentive to represent findings in a biased manner.

### Plans for updating the review

Heather M. Munthe‐Kaas will be responsible for updating this review as additional evidence and/or funding becomes available.

## Footnotes

1 Kertesz 2007 58. Kertesz SG, Mullins AN, Schumacher JE, Wallace D, Kirk K, Milby JB. Long‐term housing and work outcomes among treated cocaine‐dependent homeless persons. Journal of Behavioral Health Services & Research. 2007;34(1):17‐33. reported standardized deviations, so the review authors converted this number to standard errors since this is a more correct statistic for the data. The data included in this analysis was not available from the publication, but was sent by the study authors to the review team upon request.

## Appendix 1: Glossary

### Table 1.1 Glossary of relevant terms

**Table:**

**Abstinence‐contingent housing**	Housing offered where residents are expected to abstain from alcohol or drugs.
**At‐risk of Homelessness**	People who are living in sub‐standard, unstable or unsafe housing. This includes people who are “couch surfing,” which means they are staying with family or friends, living in trailers, doubled or tripled up in small apartments or living in unsafe and unsanitary conditions (93).
**Broker case management**	A brief approach to case management. The case manager does not provide services, but rather attempts to help clients identify their own needs and broker supportive services (85).
**Case management**	A collaborative process that assesses, plans, implements, coordinates, monitors, and evaluates the options and services required to meet an individual's health needs, using communication and available resources to promote quality, cost‐effective outcomes (91).
**Case Manager**	A healthcare professional who is responsible for coordinating the care delivered to an assigned group of patients based on diagnosis or need. Other responsibilities include patient/family education, advocacy, delays management, and outcomes monitoring and management. Case managers work with people to get the healthcare and other community services they need, when they need them, and for the best value (91).
**Caseload**	The total number of patients followed by a case manager at any point in time (91).
**Community reinforcement approach (CRA)**	“CRA is an operant‐based therapy with the goal to help individuals restructure their environment so that drug use or other maladaptive behaviors are no longer reinforced and other positive behaviors are reinforced… Therapists follow a standard set of core procedures… [which] include topics include (1) a functional analysis of using behaviors, (2) refusal skills training, and (3) relapse prevention (4) job skills, (5) social skills training including communication and problem‐solving skills, (6) social and recreational counseling, (7) anger management and affect regulation” (76), p5.
**Continuum of Care**	The continuum of care matches ongoing needs of the individuals being served by the case management process with the appropriate level and type of health, medical, financial, legal and psychosocial care for services within a setting or across multiple settings (91).
**Continuum of Care**	Federal program stressing permanent solutions to homelessness (HUD)
**Critical time intervention**	Community‐based case management in three phases of three months each. 1) Transition to community ‐ case manager tries to identify needs and form connections. 2) Try out ‐ where case manager and participants test out support system while trying to secure stable housing; 3) Transfer to care – refinements are made to support system to ensure long‐term sustainability and case managers cut down on contact with participants (72).
**Emergency housing**	Short‐term shelter for people in crisis. Some emergency shelters also provide meals and support services to the people who stay there (93).
**Group Home**	A home that is shared by a number of tenants who are generally expected to participate in shared living arrangements and activities. There is usually 24‐hour support staff on site (93).
**Homeless**	**Australia** (Supported Accommodation Assistance Program Act 1994) ‐ A person is homeless if, and only if, he or she has inadequate access to safe and secure housing. **United Kingdom, London** (A statutory definition included in Section 175, 1966 House Act) ‐ Have no accommodation in the UK / elsewhere, Cannot secure entry to accommodation, Are threatened with homelessness within the next 28 days, Have no accommodation which is reasonable for them to occupy **USA** (The McKinney‐Vento Homeless Education Assistance Act, Section 725, defines “homeless children and youths” ‐ (A) means individuals who lack a fixed, regular, and adequate night‐time residence (within the meaning of section 103(a)(1)); and (B) includes— children and youths who are sharing the housing of other persons due to loss of housing, economic hardship, or a similar reason; are living in motels, hotels, trailer parks, or camping grounds due to the lack of alternative adequate accommodations; are living in emergency or transitional shelters; are abandoned in hospitals; or are awaiting foster care placement; (ii) children and youths who have a primary night‐time residence that is a public or private place not designed for or ordinarily used as a regular sleeping accommodation for human beings (within the meaning of section 103(a)(2)(C)); (iii) children and youths who are living in cars, parks, public spaces, abandoned buildings, substandard housing, bus or train stations, or similar settings; and (iv) migratory children (as such term is defined in section 1309 of the Elementary and Secondary Education Act of 1965) who qualify as homeless for the purposes of this subtitle because the children are living in circumstances described in clauses through (iii). USA (adults): four federally defined categories under which individuals and families might qualify as homeless: 1) Literally homeless; 2) Imminent risk of homelessness;3) Homeless under other Federal statutes; and 4)Fleeing/attempting to flee domestic violence.
**Housing First**	Founded on the idea that housing is a basic right. The two core foundations of the program include psychiatric rehabilitation and consumer choice. Individuals are encouraged to define their own needs and goals. Housing is provided immediately by the program if the individual wishes, and there are no contingencies related to treatment or sobriety. The individual is also offered treatment, in the form of an adapted version of Assertive Community treatment (addition of a nurse practitioner to address physical health problems, and a housing specialist) (24).
**Independent Living**	A service delivery concept that encourages the maintenance of control over one's life based on the choice of acceptable options that minimize reliance on others performing everyday activities (91).
**Intensity of Service**	An acuity of illness criteria based on the evaluation/treatment plan, interventions, and anticipated outcomes (91)
**Intensive case management (ICM)**	A thorough, long‐term service to assist clients with serious mental illness (particularly those with psychiatric and functional disabilities and a history of not adhering to prescribed outpatient treatment) by establishing and maintaining linkages with community‐based service providers. ICM typically provides referrals to treatment programs, maintains advocacy for clients, provides counseling and crisis intervention, and assists in a wide variety of other basic services (90).
**Motivational Enhancement Therapy (MET)**	An adaptation of motivational interviewing which includes feedback Motivational interviewing has four principles: “express accurate empathy, develop discrepancy, roll with resistance and support self‐efficacy” (76), p5.
**Non‐abstinence‐contingent housing (wet housing)**	Housing where tenants are not expected to abstain from using alcohol and other drugs, and where entering a rehabilitation program is not a requirement. Tenants have access to recovery services and get to decide if and when they use these services. Wet housing programs follow a harm reduction philosophy. For more on harm reduction see below (93).
**Permanent housing**	Long‐term housing with no maximum length of stay (93).
**Private Market housing**	Traditional rental housing that is run by private landlords rather than a housing program (93).
**Quasi‐experimental study design**	Methods of allocating people to a trial that are not random, but were intended to produce similar groups when used to allocate participants. Quasi‐random methods include: allocation by the person's date of birth, by the day of the week or month of the year, by a person's medical record number, or just allocating every alternate person. In practice, these methods of allocation are relatively easy to manipulate, introducing selection bias. See also random allocation, randomisation (92).
**Randomized controlled trial**	An experiment in which two or more interventions, possibly including a control intervention or no intervention, are compared by being randomly allocated to participants. In most trials one intervention is assigned to each individual but sometimes assignment is to defined groups of individuals (for example, in a household) or interventions are assigned within individuals (for example, in different orders or to different parts of the body) (92).
**Section 8 Housing Vouchers**	Housing Assistance Payment Program (Housing and Community Development Act of 1974) / Housing Choice Voucher Program (Housing and Community Development Act of 1974) (HUD) Case Management: A collaborative process of assessment, planning, facilitation, care+B2:C32 coordination, evaluation, and advocacy for options and services to facilitate an individual's and family's comprehensive health needs through communication and available resources to promote quality (HUD
**Subsidized housing**	Housing that receives funding from the government or community organization. Tenants who live in subsidized housing pay rent that is less than market value (93).
**Supported housing**	Affordable housing where the tenants have access to support services in addition to housing. These services vary and can include: Life skills training: income management, job training, medication management; Medical care; Social activities; Problem substance use rehabilitation programs; Case management (93).
**Transitional housing**	Time‐limited, affordable, supported or independent housing. Tenants can usually remain in transitional housing for up to 2 or 3 years (93).

## Appendix 2: Search strategy

### Search strategy 2016

**Oppdateringssøk nr. 2, 20. januar 2016**

Oppdatering av søk utført i 2010.

Databaser søkt: MEDLINE, PsycINFO, ISI Web of Knowledge, ERIC, CINAHL, Cochrane CENTRAL, Sociological Abstracts, Social Services Abstracts, PubMed.

Applied Social Sciences Index and Abstracts (ASSIA) ble ikke søkt på grunn av manglende tilgang.

Søketreff totalt: 593

Søketreff etter dublettkontroll: 323

**PsycINFO (via Ovid)**

1806 to January Week 2 2016

**Dato**: 20. januar 2016

**Antall treff**: 64

**Kommentarer:** Dette søket er gjort via OVID og ikke i EBSCOHOST som det opprinnelige søket var.

1. runaway behavior/
2. homeless/
3. homeless mentally ill/
4. (evict* or homeless* or “housing excl*” or “living on the street*” or “residential stability” or “stable housing” or “street dwell*” or “Private dwell*” or “Improvised dwell*” or “Shelter dwell*” or “street liv*” or “Street life” or “street youth” or “street children” or “street people” or “marginally housed” or “precarious housing” or runaway* or “Run away from home” or “Running away” or “Ran away” or “Going missing” or “Bag lady” or Houseless* or Unhoused or “without a roof” or Roofless or “rough sleeper” or “rough sleepers” or “Rough sleeping” or Destitute* or “Skid row*” or “Street people” or “Street person*” or “Street youth*” or “Street child” or “Street children” or “Street life” or “Street living” or “Sleeping rough” or “sleep rough” or “rough sleep” or “emergency accommodation” or “temporary accommodation” or “Insecure accommodation” or “overcrowded accommodation” or “sleepers out”).tw.
5. 1 or 2 or 3 or 4
6. (“Housing first” or “Pathways to Housing” or “Homeless Veterans Reintegration Program” or “Access to Community Care and Effective Services and Supports” or “Support* Housing Program” or “Housing and Urban Development Veterans Affairs Supported Housing program” or “HUD‐VASH” or “Sober Transitional Housing and Employment Project” or “sober house placement*” or “Housing ladders” or “Staircase housing” or “low threshold housing” or “Critical Time Intervention”).tw.
7. 5 or 6
8. (quasi‐experimental or quasi‐experiment or quasiexperiment or quasiexperimental or Propensity score or propensity scores or “control group” or “control groups” or “controlled group” or “controlled groups” or “treatment group” or “treatment groups” or “comparison group” or “comparison groups” or “wait‐list” or “waiting list” or “wait‐lists” or “waiting lists” or “intervention group” or “intervention groups” or “experimental group” or “experimental groups” or “matched control” or “matched groups” or “matched comparison” or “experimental trial” or “experimental design” or “experimental method” or “experimental methods” or “experimental study” or “experimental studies” or “experimental evaluation” or “experimental test” or “experimental tests” or “experimental testing” or “experimental assessment” or placebo or “assessment only” or treatment‐as‐usual or “services as usual” or “care as usual” or “usual treatment” or “usual service” or “usual services” or “usual care” or “standard treatment” or “standard treatments” or “standard service” or “standard services” or “standard care” or “traditional treatment” or “traditional service” or “traditional care” or “ordinary treatment” or “ordinary service” or “ordinary services” or “ordinary care” or “comparison sample” or propensity‐matched or control sample or intervention sample or assigned randomly or randomly assigned or random* control*).tw.
9. treatment outcomes/
10. group*.ab.
11. 9 and 10
12. quasi experimental methods/
13. exp experimental design/
14. clinical trials/
15. placebo/
16. random sampling/
17. (“comparative testing” or “control groups” or “experimental groups” or “matched groups” or “quasiexperimental design”).tw.
18. (“random assignment” or “random allocation” or “randomi?ed control*” or “randomi?ed trial” or “randomi?ed design” or “randomi?ed method” or “randomi?ed evaluation” or “randomi?ed test” or “randomi?ed assessment”).tw.
19. (Controlled trial or Control trial or CCT).tw.
20. rct.tw.
21. 18 or 20
22. 8 or 11 or 12 or 13 or 14 or 15 or 16 or 17 or 19 or 21
23. 7 and 22
24. (201410* or 201411* or 201412* or 2015* or 2016*).up,yr.
25. 23 and 24

**CINAHL (via EBSCO)**

**Dato**: 21. januar 2016

**Antall treff**: 67

1 (MH “Homeless Persons”) or (MH “Homelessness”)
2 (AB evict* or homeless* or housing excl* or living on the street* or residential stability or stable housing or street dwell* or Private dwell* or Improvised dwell* or Shelter dwell* or street liv* or Street life or street youth or street children or street people or marginally housed or precarious housing or Housing first or runaway* or Run away from home or Running away or Ran away or Going missing or Bag lady or Houseless* or Unhoused or without a roof or Roofless or rough sleeper or rough sleepers or Rough sleeping or Destitute* or Skid row* or Street people OR Street person* OR Street youth* OR Street child OR Street children OR Street life OR Street living or Sleeping rough or sleep rough or rough sleep or emergency accommodation OR temporary accommodation or Insecure accommodation OR overcrowded accommodation or sleepers out) or (TI evict* or homeless* or housing excl* or living on the street* or residential stability or stable housing or street dwell* or Private dwell* or Improvised dwell* or Shelter dwell* or street liv* or Street life or street youth or street children or street people or marginally housed or precarious housing or Housing first or runaway* or Run away from home or Running away or Ran away or Going missing or Bag lady or Houseless* or Unhoused or without a roof or Roofless or rough sleeper or rough sleepers or Rough sleeping or Destitute* or Skid row* or Street people OR Street person* OR Street youth* OR Street child OR Street children OR Street life OR Street living or Sleeping rough or sleep rough or rough sleep or emergency accommodation OR temporary accommodation or Insecure accommodation OR overcrowded accommodation or sleepers out)
3 AB Housing first OR Pathways to Housing OR Homeless Veterans Reintegration Program OR Access to Community Care and Effective Services and Supports OR Support* Housing Program OR Housing and Urban Development–Veterans Affairs Supported Housing program OR HUD‐VASH OR Sober Transitional Housing and Employment Project OR sober house placement* OR Housing ladders OR Staircase housing OR low threshold housing OR Critical Time Intervention
4 S1 OR S2 OR S3
5 (AB(quasi‐experimental OR quasi‐experiment or quasiexperiment OR quasiexperimental OR Propensity score OR propensity scores OR “control group” OR “control groups” OR “controlled group” OR “controlled groups” OR “treatment group” OR “treatment groups” OR “comparison group” OR “comparison groups” OR “wait‐list” OR “waiting list” OR “wait‐lists” OR “waiting lists” OR “intervention group” OR “intervention groups” OR “experimental group” OR “experimental groups” OR “matched control” OR “matched groups” OR “matched comparison” OR “experimental trial” OR “experimental design” OR “experimental method” OR “experimental methods” OR “experimental study” OR “experimental studies” OR “experimental evaluation” OR “experimental test” OR “experimental tests” OR “experimental testing” OR “experimental assessment” OR placebo OR “assessment only” OR treatment‐as‐usual OR “services as usual” OR “care as usual” OR “usual treatment” OR “usual service” OR “usual services” OR “usual care” OR “standard treatment” OR “standard treatments” OR “standard service” OR “standard services” OR “standard care” OR “traditional treatment” OR “traditional service” OR “traditional care” OR “ordinary treatment” OR “ordinary service” OR “ordinary services” OR “ordinary care” OR “comparison sample” OR propensity‐matched OR control sample OR intervention sample OR assigned randomly OR randomly assigned OR random* control*)) OR (TI(quasi‐experimental OR quasi‐experiment or quasiexperiment OR quasiexperimental OR Propensity score OR propensity scores OR “control group” OR “control groups” OR “controlled group” OR “controlled groups” OR “treatment group” OR “treatment groups” OR “comparison group” OR “comparison groups” OR “wait‐list” OR “waiting list” OR “wait‐lists” OR “waiting lists” OR “intervention group” OR “intervention groups” OR “experimental group” OR “experimental groups” OR “matched control” OR “matched groups” OR “matched comparison” OR “experimental trial” OR “experimental design” OR “experimental method” OR “experimental methods” OR “experimental study” OR “experimental studies” OR “experimental evaluation” OR “experimental test” OR “experimental tests” OR “experimental testing” OR “experimental assessment” OR placebo OR “assessment only” OR treatment‐as‐usual OR “services as usual” OR “care as usual” OR “usual treatment” OR “usual service” OR “usual services” OR “usual care” OR “standard treatment” OR “standard treatments” OR “standard service” OR “standard services” OR “standard care” OR “traditional treatment” OR “traditional service” OR “traditional care” OR “ordinary treatment” OR “ordinary service” OR “ordinary services” OR “ordinary care” OR “comparison sample” OR propensity‐matched OR control sample OR intervention sample OR assigned randomly OR randomly assigned OR random* control*))
6 (quasi‐experimental OR quasi‐experiment or quasiexperiment OR quasiexperimental OR Propensity score OR propensity scores OR “control group” OR “control groups” OR “controlled group” OR “controlled groups” OR “treatment group” OR “treatment groups” OR “comparison group” OR “comparison groups” OR “wait‐list” OR “waiting list” OR “wait‐lists” OR “waiting lists” OR “intervention group” OR “intervention groups” OR “experimental group” OR “experimental groups” OR “matched control” OR “matched groups” OR “matched comparison” OR “experimental trial” OR “experimental design” OR “experimental method” OR “experimental methods” OR “experimental study” OR “experimental studies” OR “experimental evaluation” OR “experimental test” OR “experimental tests” OR “experimental testing” OR “experimental assessment” OR placebo OR “assessment only” OR treatment‐as‐usual OR “services as usual” OR “care as usual” OR “usual treatment” OR “usual service” OR “usual services” OR “usual care” OR “standard treatment” OR “standard treatments” OR “standard service” OR “standard services” OR “standard care” OR “traditional treatment” OR “traditional service” OR “traditional care” OR “ordinary treatment” OR “ordinary service” OR “ordinary services” OR “ordinary care” OR “comparison sample” OR propensity‐matched OR control sample OR intervention sample OR assigned randomly OR randomly assigned OR random* control*)
7 (MH “Quasi‐Experimental Studies”) or (MH “Nonequivalent Control Group”) or (MH “Control Group”) or (MH “Experimental Studies+”) or (MH “Waiting Lists”) or (MH “Matched‐Pair Analysis”) or (MH “Clinical Trials+”) or (MH “Placebos”) or (MH “Random Assignment”) or (MH “Random Sample+”) or (MH “Matched‐Pair Analysis”) or (MH “Case Control Studies”)
8 (MH “Treatment Outcomes”) and (AB group)
9 TI random assignment or TI random allocation or TI randomi?ed control* or TI randomi?ed trial or TI randomi?ed design or TI randomi?ed method or TI randomi?ed evaluation or TI randomi?ed test or TI randomi?ed assessment or TI randomi?ed or (AB random assignment or AB random allocation or AB randomi?ed control* or AB randomi?ed trial or AB randomi?ed design or AB randomi?ed method or AB randomi?ed evaluation or AB randomi?ed test or AB randomi?ed assessment) or (KW random assignment or KW random allocation or KW randomi?ed control* or KW randomi?ed trial or KW randomi?ed design or KW randomi?ed method or KW randomi?ed evaluation or KW randomi?ed test or KW randomi?ed assessment)
10 (MH “Clinical Trials+”)
11 TX Controlled trial or TX Control trial
12 S5 OR S6 OR S7 OR S8 OR S9 OR S10 OR S11
13 S1 OR S2 OR S3 Limiters ‐ Publication Type: Clinical Trial
14 S4 AND S12
15 S13 OR S14 Limiters ‐ Published Date: 20140101‐20160231

**MEDLINE** In‐Process & Other Non‐Indexed Citations, Ovid MEDLINE(R) Daily, Ovid MEDLINE(R) and Ovid OLDMEDLINE(R) 1946 to Present

**Dato**: 20. januar 2016

**Antall treff**: 142

**Kommentarer:** Det opprinnelige søket og oppdateringssøket i 2014 ble gjort i PubMed. Dette oppdateringssøket ble overført til MEDLINE. I tillegg ble det gjort et enkelt søk i PubMed, for å finne studier registrert med «Publication status ahead of print».

1. homeless persons/ or homeless youth/
2. (evict* or homeless* or “housing excl*” or “living on the street*” or “residential stability” or “stable housing” or “street dwell*” or “Private dwell*” or “Improvised dwell*” or “Shelter dwell*” or “street liv*” or “Street life” or “street youth” or “street children” or “street people” or “marginally housed” or “precarious housing” or runaway* or “Run away from home” or “Running away” or “Ran away” or “Going missing” or “Bag lady” or Houseless* or Unhoused or “without a roof” or Roofless or “rough sleeper” or “rough sleepers” or “Rough sleeping” or Destitute* or “Skid row*” or “Street people” or “Street person*” or “Street youth*” or “Street child” or “Street children” or “Street life” or “Street living” or “Sleeping rough” or “sleep rough” or “rough sleep” or “emergency accommodation” or “temporary accommodation” or “Insecure accommodation” or “overcrowded accommodation” or “sleepers out”).tw.
3. (“Housing first” or “Pathways to Housing” or “Homeless Veterans Reintegration Program” or “Access to Community Care and Effective Services and Supports” or “Support* Housing Program” or “Housing and Urban Development–Veterans Affairs Supported Housing program” or “HUD‐VASH” or “Sober Transitional Housing and Employment Project” or “sober house placement*” or “Housing ladders” or “Staircase housing” or “low threshold housing” or “Critical Time Intervention”).tw.
4. 1 or 2 or 3
5. (quasi‐experimental or quasi‐experiment or quasiexperiment or quasiexperimental or Propensity score or propensity scores or “control group” or “control groups” or “controlled group” or “controlled groups” or “treatment group” or “treatment groups” or “comparison group” or “comparison groups” or “wait‐list” or “waiting list” or “wait‐lists” or “waiting lists” or “intervention group” or “intervention groups” or “experimental group” or “experimental groups” or “matched control” or “matched groups” or “matched comparison” or “experimental trial” or “experimental design” or “experimental method” or “experimental methods” or “experimental study” or “experimental studies” or “experimental evaluation” or “experimental test” or “experimental tests” or “experimental testing” or “experimental assessment” or placebo or “assessment only” or treatment‐as‐usual or “services as usual” or “care as usual” or “usual treatment” or “usual service” or “usual services” or “usual care” or “standard treatment” or “standard treatments” or “standard service” or “standard services” or “standard care” or “traditional treatment” or “traditional service” or “traditional care” or “ordinary treatment” or “ordinary service” or “ordinary services” or “ordinary care” or “comparison sample” or propensity‐matched or control sample or intervention sample or assigned randomly or randomly assigned or random* control*).tw.
6. exp Treatment Outcome/
7. (group or groups).tw.
8. 6 and 7
9. Propensity Score/
10. exp Control Groups/
11. exp Case‐Control Studies/
12. exp Matched‐Pair Analysis/
13. exp Randomized Controlled Trials as Topic/
14. Randomized Controlled Trial.pt.
15. exp Random Allocation/
16. (random* or trial or rct).ti.
17. clinical trial.pt.
18. controlled clinical trial.pt.
19. (controlled adj2 trial*).tw.
20. (randomi?ed adj2 trial).tw.
21. 5 or 8 or 9 or 10 or 11 or 12 or 13 or 14 or 15 or 16 or 17 or 18 or 19 or 20
22. 4 and 21
23. (201410* or 201411* or201412* or 2015* or 2016*).ed,yr.
24. 22 and 23
25. remove duplicates from 24

**Cochrane CENTRAL**

**Dato**: 20. januar 2016

**Antall treff**: 78

#1 MeSH descriptor: [Homeless Persons] explode all trees
#2 evict* or homeless* or (housing next excl*) or (“living on the” next street*) or “residential stability” or “stable housing” or (street next dwell*) or (Private next dwell*) or (Improvised next dwell*) or (Shelter next dwell*) or (street next liv*) or “Street life” or “street youth” or “street children” or “street people” or “marginally housed” or “precarious housing” or runaway* or “Run away from home” or “Running away” or “Ran away” or “Going missing” or “Bag lady” or Houseless* or Unhoused or “without a roof” or Roofless or “rough sleeper” or “rough sleepers” or “Rough sleeping” or Destitute* or (Skid next row*) or “Street people” or (Street next person*) or (Street next youth*) or “Street child” or “Street children” or “Street life” or “Street living” or “Sleeping rough” or “sleep rough” or “rough sleep” or “emergency accommodation” or “temporary accommodation” or “Insecure accommodation” or “overcrowded accommodation” or “sleepers out”
#3 “Housing first” or “Pathways to Housing” or “Homeless Veterans Reintegration Program” or “Access to Community Care and Effective Services and Supports” or “Supported Housing Program” or “Support Housing Program” or “Housing and Urban Development–Veterans Affairs Supported Housing program” or “HUD‐VASH” or “Sober Transitional Housing and Employment Project” or “sober house placement*” or “Housing ladders” or “Staircase housing” or “low threshold housing” or “Critical Time Intervention”
#4 #1 or #2 or #3 Publication Year from 2014 to 2016

**Eric via ProQuest**

**Dato:** 20. januar 2016

**Antall treff:** 4

(SU.EXACT.EXPLODE(“Homeless People”) OR (evict* or homeless* or “housing excl*” or “living on the street*” or “residential stability” or “stable hou‐sing” or “street dwell*” or “Private dwell*” or “Im‐provised dwell*” or “Shelter dwell*” or “street liv*” or “Street life” or “street youth” or “street children” or “street people” or “marginally housed” or “precarious housing” or “Housing first” or runaway* or “Run away from home” or “Running away” or “Ran away” or “Going missing” or “Bag lady” or Houseless* or Unhoused or “without a roof” or Roofless or “rough sleeper” or “rough sleepers” or “Rough sleeping” or Destitute* or “Skid row*” or “Street people” or “Street person*” or “Street youth*” or “Street child” or “Street children” or “Street life” or “Street living” or “Sleeping rough” or “sleep rough” or “rough sleep” or “emergency accommodation” or “temporary accommodation” or “Insecure accommodation” or “overcrowded accommodation” or “sleepers out” OR “Housing first” or “Pathways to Housing” or “Homeless Veterans Reintegration Program” or “Access to Community Care and Effective Services and Supports” or “Support* Housing Program” or “Housing and Urban Development–Veterans Affairs Supported Housing program” or “HUD‐VASH” or “Sober Transitional Housing and Employment Project” or “sober house placement*” or “Housing ladders” or “Staircase housing” or “low threshold housing” or “Critical Time Intervention”)) AND

((SU.EXACT(“Comparative Testing”) OR SU.EXACT(“Control Groups”) OR SU.EXACT(“Matched Groups”) OR SU.EXACT(“Experimental Groups”) OR SU.EXACT.EXPLODE(“Quasiexperimental Design”)) OR (quasi‐experiment* or quasiexperiment* or “quasi experiment*” or “propensity scor*” or “control group*” or “controlled group*” or “treatment group*” or “comparison group*” or “wait‐list*” or “waiting list*” or “intervention group*” or “experimental group*” or “matched control*” or “matched group*” or “matched comparison*” or “experimental trial*” or “experimental design*” or “experimental method*” or “experimental stud*” or “experimental evaluation*” or “experimental test*” or “experimental assessment*” or placebo or “assessment only” or “treatment as usual” or “services as usual” or “care as usual” or “usual treatment*” or “usual care” or “usual service” or “usual services” or “standard treatment” or “standard service*” or “standard care” or “traditional treatment” or “traditional service*” or “traditional care” or “ordinary treatment” or “ordinary service*” or “ordinary care” or “comparison sample” or propensity‐matched or “control sample” or “intervention sample” or “assigned randomly” or “randomly assigned” or “random* control*”) OR ab(“treatment outcome” AND Group*) OR (ab(random*) OR ti((random* OR trial))) OR ((“control trial”) or (“controlled trial”) or CCT))

Limits applied published 2014‐2016

**Social Services Abstracts‎ (1979 ‐ current) og Sociological Abstracts‎ (1952 ‐ current) via ProQuest**

**Dato:** 20. januar 2016

**Antall treff:** 29

(SU.EXACT.EXPLODE(“Homelessness”) OR (evict* OR homeless* OR “housing excl*” OR “living on the street*” OR “residential stability” OR “stable hou‐sing” OR “street dwell*” OR “Private dwell*” OR “Im‐provised dwell*” OR “Shelter dwell*” OR “street liv*” OR “Street life” OR “street youth” OR “street children” OR “street people” OR “marginally housed” OR “precarious housing” OR “Housing first” OR runaway* OR “Run away from home” OR “Running away” OR “Ran away” OR “Going missing” OR “Bag lady” OR Houseless* OR Unhoused OR “without a roof” OR Roofless OR “rough sleeper” OR “rough sleepers” OR “Rough sleeping” OR Destitute* OR “Skid row*” OR “Street people” OR “Street person*” OR “Street youth*” OR “Street child” OR “Street children” OR “Street life” OR “Street living” OR “Sleeping rough” OR “sleep rough” OR “rough sleep” OR “emergency accommodation” OR “temporary accommodation” OR “Insecure accommodation” OR “overcrowded accommodation” OR “sleepers out” OR “Housing first” OR “Pathways to Housing” OR “Homeless Veterans Reintegration Program” OR “Access to Community Care and Effective Services and Supports” OR “Support* Housing Program” OR “Housing and Urban Development–Veterans Affairs Supported Housing program” OR “HUD‐VASH” OR “Sober Transitional Housing and Employment Project” OR “sober house placement*” OR “Housing ladders” OR “Staircase housing” OR “low threshold housing” OR “Critical Time Intervention”))

AND

(ab((“quasi‐experimental” OR quasi‐experiment OR quasiexperiment OR quasiexperimental OR “Propensity score” OR “propensity scores” OR “control group” OR “control groups” OR “controlled group” OR “controlled groups” OR “treatment group” OR “treatment groups” OR “comparison group” OR “comparison groups” OR “wait‐list” OR “waiting list” OR “wait‐lists” OR “waiting lists” OR “intervention group” OR “intervention groups” OR “experimental group” OR “experimental groups” OR “matched control” OR “matched groups” OR “matched comparison” OR “experimental trial” OR “experimental design” OR “experimental method” OR “experimental methods” OR “experimental study” OR “experimental studies” OR “experimental evaluation” OR “experimental test” OR “experimental tests” OR “experimental testing” OR “experimental assessment” OR placebo OR “assessment only” OR “treatment‐as‐usual” OR “services as usual” OR “care as usual” OR “usual treatment” OR “usual service” OR “usual services” OR “usual care” OR “standard treatment” OR “standard treatments” OR “standard service” OR “standard services” OR “standard care” OR “traditional treatment” OR “traditional service” OR “traditional care” OR “ordinary treatment” OR “ordinary service” OR “ordinary services” OR “ordinary care” OR “comparison sample” OR “propensity‐matched” OR “control sample” OR “intervention sample” OR “assigned randomly” OR “randomly assigned” OR “random* control*”)) OR ti((“random assignment” OR “random allocation” OR “randomi?ed control*” OR “randomi?ed trial” OR “randomi?ed design” OR “randomi?ed method” OR “randomi?ed evaluation” OR “randomi?ed test” OR “randomi?ed assessment” OR randomi?ed)) OR ab((“random assignment” OR “random allocation” OR “randomi?ed control*” OR “randomi?ed trial” OR “randomi?ed design” OR “randomi?ed method” OR “randomi?ed evaluation” OR “randomi?ed test” OR “randomi?ed assessment” OR randomi?ed)) OR ab((“Controlled trial” OR “Control trial” OR CCT)))

Limits applied published 2014‐2016

**ISI Web of Knowledge**

**Dato:** 20. Januar 2016

**Antall treff:** 180

# 7 #4 AND #3 Refined by: Databases: (WOS) AND PUBLICATION YEARS: (2014 OR 2015 OR 2016)
# 6 #4 AND #3 Refined by: Databases: (WOS)
# 5 #4 AND #3
# 4 TOPIC: ((“comparative testing” or “control groups” or “experimental groups” or “matched groups” or “quasiexperimental design”)) OR TOPIC: ((quasi‐experiment* or quasiexperiment* or “quasi experiment*” or “propensity scor*” or “control group*” or “controlled group*” or “treatment group*” or “comparison group*” or “wait‐list*” or “waiting list*” or “intervention group*” or “experimental group*” or “matched control*” or “matched group*” or “matched comparison*” or “experimental trial*” or “experimental design*” or “experimental method*” or “experimental stud*” or “experimental evaluation*” or “experimental test*” or “experimental assessment*” or placebo or “assessment only” or “treatment as usual” or “services as usual” or “care as usual” or “usual treatment*” or “usual care” or “usual service” or “usual services” or “standard treatment” or “standard service*” or “standard care” or “traditional treatment” or “traditional service*” or “traditional care” or “ordinary treatment” or “ordinary service*” or “ordinary care” or “comparison sample” or propensity‐matched or “control sample” or “intervention sample” or “assigned randomly” or “randomly assigned” or “random* control*”)) OR TOPIC:(“random assignment” or “random allocation” or “randomi?ed control*” or “randomi?ed trial” or “randomi?ed design” or “randomi?ed method” or “randomi?ed evaluation” or “randomi?ed test” or “randomi?ed assessment” OR control trial or controlled trial or CCT)
# 3 #2 OR #1
# 2 TOPIC: ((“Housing first” or “Pathways to Housing” or “Homeless Veterans Reintegration Program” or “Access to Community Care and Effective Services and Supports” or “Support* Housing Program” or “Housing and Urban Development–Veterans Affairs Supported Housing program” or “HUD‐VASH” or “Sober Transitional Housing and Employment Project” or “sober house placement*” or “Housing ladders” or “Staircase housing” or “low threshold housing” or “Critical Time Intervention”))
# 1 TOPIC: ((evict* or homeless* or “housing excl*” or “living on the street*” or “residential stability” or “stable hou‐sing” or “street dwell*” or “Private dwell*” or “Im‐provised dwell*” or “Shelter dwell*” or “street liv*” or “Street life” or “street youth” or “street children” or “street people” or “marginally housed” or “precarious housing” or “Housing first” or runaway* or “Run away from home” or “Running away” or “Ran away” or “Going missing” or “Bag lady” or Houseless* or Unhoused or “without a roof” or Roofless or “rough sleeper” or “rough sleepers” or “Rough sleeping” or Destitute* or “Skid row*” or “Street people” or “Street person*” or “Street youth*” or “Street child” or “Street children” or “Street life” or “Street living” or “Sleeping rough” or “sleep rough” or “rough sleep” or “emergency accommodation” or “temporary accommodation” or “Insecure accommodation” or “overcrowded accommodation” or “sleepers out”))

**PubMed**

**Dato:** 20. januar 2016

**Antall treff:** 29

**Kommentar:** Supplement til MEDLINE‐søk. Enkelt søk for å finne artikler registrert med koden «Published ahead of print»

homeless* AND (random* or trial or control*) AND pubstatusaheadofprint

### Search strategy 2014

**Database: PsycINFO** 1806 to October Week 1 2014

**Dato**: 8. oktober 2014

**Antall treff**: 169

**Kommentarer:** Dette søket er gjort gjennom databasen OVID og ikke i EBSCOHOST som forrige søk var.

**Table:**

**#**	**Searches**	**Results**
1	runaway behavior/	595
2	homeless/	4985
3	homeless mentally ill/	526
4	(evict* or homeless* or “housing excl*” or “living on the street*” or “residential stability” or “stable housing” or “street dwell*” or “Private dwell*” or “Improvised dwell*” or “Shelter dwell*” or “street liv*” or “Street life” or “street youth” or “street children” or “street people” or “marginally housed” or “precarious housing” or runaway* or “Run away from home” or “Running away” or “Ran away” or “Going missing” or “Bag lady” or Houseless* or Unhoused or “without a roof” or Roofless or “rough sleeper” or “rough sleepers” or “Rough sleeping” or Destitute* or “Skid row*” or “Street people” or “Street person*” or “Street youth*” or “Street child” or “Street children” or “Street life” or “Street living” or “Sleeping rough” or “sleep rough” or “rough sleep” or “emergency accommodation” or “temporary accommodation” or “Insecure accommodation” or “overcrowded accommodation” or “sleepers out”).tw.	10342
5	1 or 2 or 3 or 4	10450
6	(“Housing first” or “Pathways to Housing” or “Homeless Veterans Reintegration Program” or “Access to Community Care and Effective Services and Supports” or “Support* Housing Program” or “Housing and Urban Development–Veterans Affairs Supported Housing program” or “HUD‐VASH” or “Sober Transitional Housing and Employment Project” or “sober house placement*” or “Housing ladders” or “Staircase housing” or “low threshold housing” or “Critical Time Intervention”).tw.	236
7	5 or 6	10480
8	(quasi‐experimental or quasi‐experiment or quasiexperiment or quasiexperimental or Propensity score or propensity scores or “control group” or “control groups” or “controlled group” or “controlled groups” or “treatment group” or “treatment groups” or “comparison group” or “comparison groups” or “wait‐list” or “waiting list” or “wait‐lists” or “waiting lists” or “intervention group” or “intervention groups” or “experimental group” or “experimental groups” or “matched control” or “matched groups” or “matched comparison” or “experimental trial” or “experimental design” or “experimental method” or “experimental methods” or “experimental study” or “experimental studies” or “experimental evaluation” or “experimental test” or “experimental tests” or “experimental testing” or “experimental assessment” or placebo or “assessment only” or treatment‐as‐usual or “services as usual” or “care as usual” or “usual treatment” or “usual service” or “usual services” or “usual care” or “standard treatment” or “standard treatments” or “standard service” or “standard services” or “standard care” or “traditional treatment” or “traditional service” or “traditional care” or “ordinary treatment” or “ordinary service” or “ordinary services” or “ordinary care” or “comparison sample” or propensity‐matched or control sample or intervention sample or assigned randomly or randomly assigned or random* control*).tw.	176491
9	treatment outcomes/	25657
10	group*.ab.	654168
11	9 and 10	7266
12	quasi experimental methods/	121
13	exp experimental design/	48139
14	clinical trials/	7958
15	placebo/	3872
16	random sampling/	612
17	(“comparative testing” or “control groups” or “experimental groups” or “matched groups” or “quasiexperimental design”).tw.	18612
18	(“random assignment” or “random allocation” or “randomi?ed control*” or “randomi?ed trial” or “randomi?ed design” or “randomi?ed method” or “randomi?ed evaluation” or “randomi?ed test” or “randomi?ed assessment”).tw.	25077
19	(Controlled trial or Control trial or CCT).tw.	15202
20	rct.tw.	2050
21	18 or 20	25779
22	8 or 11 or 12 or 13 or 14 or 15 or 16 or 17 or 19 or 21	224204
23	7 and 22	681
24	limit 23 to yr="2010 ‐Current”	169

**Database:** CINAHL interface ‐ EBSCOhost Research Databases

**Dato**: 9. oktober 2014

**Antall treff**: 158

**Antall treff etter duplikatkontroll:** 124

**Kommentarer:** Fikk færre totale treff enn i det originale søket. Finner ikke noen feilkilde til tross for flere ulike fremgangsmetoder og konferering med kollegaer.

**Table:**

#	Search	Results
1	(MH “Homeless Persons”) or (MH “Homelessness”)	4,887
2	(AB evict* or homeless* or housing excl* or living on the street* or residential stability or stable housing or street dwell* or Private dwell* or Improvised dwell* or Shelter dwell* or street liv* or Street life or street youth or street children or street people or marginally housed or precarious housing or Housing first or runaway* or Run away from home or Running away or Ran away or Going missing or Bag lady or Houseless* or Unhoused or without a roof or Roofless or rough sleeper or rough sleepers or Rough sleeping or Destitute* or Skid row* or Street people OR Street person* OR Street youth* OR Street child OR Street children OR Street life OR Street living or Sleeping rough or sleep rough or rough sleep or emergency accommodation OR temporary accommodation or Insecure accommodation OR overcrowded accommodation or sleepers out) or (TI evict* or homeless* or housing excl* or living on the street* or residential stability or stable housing or street dwell* or Private dwell* or Improvised dwell* or Shelter dwell* or street liv* or Street life or street youth or street children or street people or marginally housed or precarious housing or Housing first or runaway* or Run away from home or Running away or Ran away or Going missing or Bag lady or Houseless* or Unhoused or without a roof or Roofless or rough sleeper or rough sleepers or Rough sleeping or Destitute* or Skid row* or Street people OR Street person* OR Street youth* OR Street child OR Street children OR Street life OR Street living or Sleeping rough or sleep rough or rough sleep or emergency accommodation OR temporary accommodation or Insecure accommodation OR overcrowded accommodation or sleepers out)	6,313
3	AB Housing first OR Pathways to Housing OR Homeless Veterans Reintegration Program OR Access to Community Care and Effective Services and Supports OR Support* Housing Program OR Housing and Urban Development–Veterans Affairs Supported Housing program OR HUD‐VASH OR Sober Transitional Housing and Employment Project OR sober house placement* OR Housing ladders OR Staircase housing OR low threshold housing OR Critical Time Intervention	115
4	S1 OR S2 OR S3	6,319
5	(AB(quasi‐experimental OR quasi‐experiment or quasiexperiment OR quasiexperimental OR Propensity score OR propensity scores OR “control group” OR “control groups” OR “controlled group” OR “controlled groups” OR “treatment group” OR “treatment groups” OR “comparison group” OR “comparison groups” OR “wait‐list” OR “waiting list” OR “wait‐lists” OR “waiting lists” OR “intervention group” OR “intervention groups” OR “experimental group” OR “experimental groups” OR “matched control” OR “matched groups” OR “matched comparison” OR “experimental trial” OR “experimental design” OR “experimental method” OR “experimental methods” OR “experimental study” OR “experimental studies” OR “experimental evaluation” OR “experimental test” OR “experimental tests” OR “experimental testing” OR “experimental assessment” OR placebo OR “assessment only” OR treatment‐as‐usual OR “services as usual” OR “care as usual” OR “usual treatment” OR “usual service” OR “usual services” OR “usual care” OR “standard treatment” OR “standard treatments” OR “standard service” OR “standard services” OR “standard care” OR “traditional treatment” OR “traditional service” OR “traditional care” OR “ordinary treatment” OR “ordinary service” OR “ordinary services” OR “ordinary care” OR “comparison sample” OR propensity‐matched OR control sample OR intervention sample OR assigned randomly OR randomly assigned OR random* control*)) OR (TI(quasi‐experimental OR quasi‐experiment or quasiexperiment OR quasiexperimental OR Propensity score OR propensity scores OR “control group” OR “control groups” OR “controlled group” OR “controlled groups” OR “treatment group” OR “treatment groups” OR “comparison group” OR “comparison groups” OR “wait‐list” OR “waiting list” OR “wait‐lists” OR “waiting lists” OR “intervention group” OR “intervention groups” OR “experimental group” OR “experimental groups” OR “matched control” OR “matched groups” OR “matched comparison” OR “experimental trial” OR “experimental design” OR “experimental method” OR “experimental methods” OR “experimental study” OR “experimental studies” OR “experimental evaluation” OR “experimental test” OR “experimental tests” OR “experimental testing” OR “experimental assessment” OR placebo OR “assessment only” OR treatment‐as‐usual OR “services as usual” OR “care as usual” OR “usual treatment” OR “usual service” OR “usual services” OR “usual care” OR “standard treatment” OR “standard treatments” OR “standard service” OR “standard services” OR “standard care” OR “traditional treatment” OR “traditional service” OR “traditional care” OR “ordinary treatment” OR “ordinary service” OR “ordinary services” OR “ordinary care” OR “comparison sample” OR propensity‐matched OR control sample OR intervention sample OR assigned randomly OR randomly assigned OR random* control*))	102,329
6	(quasi‐experimental OR quasi‐experiment or quasiexperiment OR quasiexperimental OR Propensity score OR propensity scores OR “control group” OR “control groups” OR “controlled group” OR “controlled groups” OR “treatment group” OR “treatment groups” OR “comparison group” OR “comparison groups” OR “wait‐list” OR “waiting list” OR “wait‐lists” OR “waiting lists” OR “intervention group” OR “intervention groups” OR “experimental group” OR “experimental groups” OR “matched control” OR “matched groups” OR “matched comparison” OR “experimental trial” OR “experimental design” OR “experimental method” OR “experimental methods” OR “experimental study” OR “experimental studies” OR “experimental evaluation” OR “experimental test” OR “experimental tests” OR “experimental testing” OR “experimental assessment” OR placebo OR “assessment only” OR treatment‐as‐usual OR “services as usual” OR “care as usual” OR “usual treatment” OR “usual service” OR “usual services” OR “usual care” OR “standard treatment” OR “standard treatments” OR “standard service” OR “standard services” OR “standard care” OR “traditional treatment” OR “traditional service” OR “traditional care” OR “ordinary treatment” OR “ordinary service” OR “ordinary services” OR “ordinary care” OR “comparison sample” OR propensity‐matched OR control sample OR intervention sample OR assigned randomly OR randomly assigned OR random* control*)	128,240
7	(MH “Quasi‐Experimental Studies”) or (MH “Nonequivalent Control Group”) or (MH “Control Group”) or (MH “Experimental Studies+”) or (MH “Waiting Lists”) or (MH “Matched‐Pair Analysis”) or (MH “Clinical Trials+”) or (MH “Placebos”) or (MH “Random Assignment”) or (MH “Random Sample+”) or (MH “Matched‐Pair Analysis”) or (MH “Case Control Studies”)	222,832
8	(MH “Treatment Outcomes”) and (AB group)	20,378
9	TI random assignment or TI random allocation or TI randomi?ed control* or TI randomi?ed trial or TI randomi?ed design or TI randomi?ed method or TI randomi?ed evaluation or TI randomi?ed test or TI randomi?ed assessment or TI randomi?ed or (AB random assignment or AB random allocation or AB randomi?ed control* or AB randomi?ed trial or AB randomi?ed design or AB randomi?ed method or AB randomi?ed evaluation or AB randomi?ed test or AB randomi?ed assessment) or (KW random assignment or KW random allocation or KW randomi?ed control* or KW randomi?ed trial or KW randomi?ed design or KW randomi?ed method or KW randomi?ed evaluation or KW randomi?ed test or KW randomi?ed assessment)	51,519
10	(MH “Clinical Trials+”)	123,522
11	TX Controlled trial or TX Control trial	37,551
12	S5 OR S6 OR S7 OR S8 OR S9 OR S10 OR S11	273,792
13	S1 OR S2 OR S3Limiters ‐ Publication Type: Clinical Trial	54
14	S4 AND S12	477
15	S13 OR S14	477
16	S13 OR S14Limiters ‐ Published Date: 20100101‐20141231	158

**Database:** PubMed

**Dato**: 9. oktober 2014

**Antall treff**: 293

**Antall treff etter duplikatkontroll:** 209

**Kommentarer:** Det er ikke lenger mulig å hente opp limits I PubMed som det er gjort i det originale søket. For å få et tilnærmet likt resultat er filter på [Publication type] lagt til søkeordene i etterkant. Usikkert om dette helt repliserer resultatene men det er så nærme vi kommer med dagens søkestruktur.

**Table:**

Search	Query	Items found
#1	Search (Homeless Youth OR Homeless Persons[MeSH Major Topic])	5304
#2	Search (evicted[tiab] or eviction[tiab] or homeless*[tiab] or housing excluded[tiab] or housing exclusion[tiab] or living on the street*[tiab] or residential stability[tiab] or stable housing[tiab] or street dwelling[tiab] or street dwellers[tiab] or Private dwelling[tiab] or Improvised dwelling[tiab] or improvised dwellings[tiab] or Shelter dwellers[tiab] or shelter dwellings[tiab] or street liv*[tiab] or Street life[tiab] or street youth[tiab] or street children[tiab] or street people[tiab] or marginally housed[tiab] or precarious housing[tiab] or Housing first[tiab] or runaway*[tiab] or Run away from home[tiab] or Running away[tiab] or Ran away[tiab] or Going missing[tiab] or Bag lady[tiab] or Houseless*[tiab] or Unhoused[tiab] or without a roof[tiab] or Roofless[tiab] or rough sleep*[tiab] or Destitute*[tiab] or Skid row*[tiab] or Street people[tiab] or street person*[tiab] or street youth[tiab] or street child*[tiab] or street life[tiab] or street living[tiab] or sleeping rough[tiab] or sleepers rough[tiab] or emergency accommodation[tiab] or temporary accommodation[tiab] or insecure accommodation[tiab] or overcrowded accomodation[tiab] or sleepers out[tiab])	9465
#3	Search (#1 or #2)	10319
#4	Search (Housing first[tiab] OR Pathways to Housing[tiab] OR Homeless Veterans Reintegration Program[tiab] OR Access to Community Care and Effective Services and Supports[tiab] OR Support* Housing Program[tiab] OR Housing and Urban Development–Veterans Affairs Supported Housing program[tiab] OR HUD‐VASH[tiab] OR Sober Transitional Housing and Employment Project[tiab] OR sober house placement*[tiab] OR Housing ladders[tiab] OR Staircase housing[tiab] OR low threshold housing[tiab] OR Critical Time Intervention[tiab])	96
#5	Search (#3 or #4)	10345
#6	Search (((“Propensity Score”[Mesh] OR “Control Groups”[Mesh]) OR “Case‐Control Studies”[Mesh]) OR “Matched‐Pair Analysis”[Mesh])	673582
#7	Search (quasi‐experimental[tiab] OR quasi‐experiment[tiab] or quasiexperiment[tiab] OR quasiexperimental[tiab] OR Propensity scor*[tiab] OR control group[tiab] OR control groups[tiab] OR controlled group[tiab] OR controlled groups[tiab] OR treatment group[tiab] OR treatment groups[tiab] OR comparison group[tiab] OR comparison groups[tiab] OR wait‐list[tiab] OR waiting list[tiab] OR wait‐lists[tiab] OR waiting lists[tiab] OR intervention group[tiab] OR intervention groups [tiab] OR experimental group[tiab] OR experimental groups[tiab] OR matched control[tiab] OR matched groups[tiab] OR matched comparison[tiab] OR experimental trial[tiab] OR experimental design[tiab] OR experimental method[tiab] OR experimental methods[tiab] OR experimental study[tiab] OR experimental studies[tiab] OR experimental evaluation[tiab] OR experimental test[tiab] OR experimental tests[tiab] OR experimental testing[tiab] OR experimental assessment[tiab] OR placebo[tiab] OR assessment only[tiab] OR treatment‐as‐usual[tiab] OR services as usual[tiab] OR care as usual[tiab] OR usual treatment[tiab] OR usual service[tiab] OR usual services[tiab] OR usual care[tiab] OR standard treatment[tiab] OR standard treatments[tiab] OR standard service[tiab] OR standard services[tiab] OR standard care[tiab] OR traditional treatment[tiab] OR traditional service[tiab] OR traditional services[tiab] OR traditional care[tiab] OR ordinary treatment[tiab] OR ordinary therapy[tiab] OR ordinary service[tiab] Or ordinary services[tiab] OR ordinary care[tiab] OR comparison sample[tiab] OR propensity‐match*[tiab] OR control sample[tiab] OR intervention sample[tiab] OR assigned randomly[tiab] OR randomly assigned[tiab])	721984
#8	Search ((“Treatment Outcome”[Mesh] AND (group[tiab] OR groups[tiab])))	175979
#9	Search (#6 or #7 or #8)	1425399
#10	Search (“Randomized Controlled Trials as Topic”[Mesh] OR “Randomized Controlled Trial “[Publication Type] OR “Random Allocation”[MeSH] OR randomized controlled trial[tiab] OR randomised controlled trial[tiab] OR (randomised[ti] AND trial[ti]) OR (randomized[ti] AND trial[ti]) OR RCT[ti])	540437
#11	Search (Meta‐Analysis) OR Randomized Controlled Trial	533359
#12	Search (#10 or #11)	601130
#16	Search ((#10 or #11)) Filters: Meta‐Analysis; Randomized Controlled Trial	422145
#17	Search Clinical Trial	994819
#18	Search (Controlled trial[tiab] or Control trial[tiab] or CCT[tiab])	76387
#19	Search (#17 or #18)	1013512
#20	Search (#17 or #18) Filters: Clinical Trial	767334
#21	Search (((#3 or #4))) AND ((#6 or #7 or #8))	863
#22	Search (((#3 or #4))) AND ((((#10 or #11))) AND ((Meta‐Analysis[ptyp] OR Randomized Controlled Trial[ptyp])))	273
#23	Search (((#3 or #4))) AND (((#17 or #18)) AND Clinical Trial[ptyp])	408
#24	Search (#21 or #22 or #23)	1118
#25	Search (#21 or #22 or #23) Filters: Humans	1069
#26	Search #25	1069
#27	Search #25 Filters: Publication date from 2010/01/01 to 2015/12/31	293

**Database: Cochrane via Wiley**

**Dato**: 10. oktober 2014

**Antall treff**: 108

**Antall treff etter duplikatkontroll**: 35

Kommentar: MeSH termen [homless persons] explode inneholder MeSH termen [homeless youth]

Cochrane Reviews (13)

All

Review

Protocol

Other Reviews (8)

Trials (80)

Methods Studies (0)

Technology Assessments (2)

Economic Evaluations (5)

Cochrane Groups (0)

**Table:**

**ID**	**Search**	**Hits**
#1	MeSH descriptor: [Homeless Persons] explode all trees	223
#2	homeless*:ti,ab,kw	398
#3	(“Housing first” or “Pathways to Housing” or “Homeless Veterans Reintegration Program” or “Access to Community Care and Effective Services and Supports” or “Support* Housing Program” or “Housing and Urban Development–Veterans Affairs Supported Housing program” or “HUD‐VASH” or “Sober Transitional Housing and Employment Project” or “sober house placement*” or “Housing ladders” or “Staircase housing” or “low threshold housing” or “Critical time intervention”):ti,ab,kw	36
#4	#1 or #2 or #3 Publication Year from 2010 to 2014	108
#5	#1 or #2 or #3	402

**Database:** Eric via Ebscohost

**Dato:** 10. oktober 2014

**Antall treff:** 16

**Antall treff etter duplikatkontroll:** 8

**Kommentar:** Søk 7 er endret fra originalsøket da det ikke var mulig å få treffene til å stemme overens. Dette søket er noe videre, men det ser ikke ut til at det har bidratt til å gi treff til trefflisten.

**Table:**

**#**	**Query**	**Results**
1	DE homeless people	1,892
2	TX evict* or homeless* or “housing excl*” or “living on the street*” or “residential stability” or “stable hou‐sing” or “street dwell*” or “Private dwell*” or “Im‐provised dwell*” or “Shelter dwell*” or “street liv*” or “Street life” or “street youth” or “street children” or “street people” or “marginally housed” or “precarious housing” or “Housing first” or runaway* or “Run away from home” or “Running away” or “Ran away” or “Going missing” or “Bag lady” or Houseless* or Unhoused or “without a roof” or Roofless or “rough sleeper” or “rough sleepers” or “Rough sleeping” or Destitute* or “Skid row*” or “Street people” or “Street person*” or “Street youth*” or “Street child” or “Street children” or “Street life” or “Street living” or “Sleeping rough” or “sleep rough” or “rough sleep” or “emergency accommodation” or “temporary accommodation” or “Insecure accommodation” or “overcrowded accommodation” or “sleepers out”	3,161
3	TX “Housing first” or “Pathways to Housing” or “Homeless Veterans Reintegration Program” or “Access to Community Care and Effective Services and Supports” or “Support* Housing Program” or “Housing and Urban Development–Veterans Affairs Supported Housing program” or “HUD‐VASH” or “Sober Transitional Housing and Employment Project” or “sober house placement*” or “Housing ladders” or “Staircase housing” or “low threshold housing” or “Critical Time Intervention”	11
4	S1 OR S2 OR S3	3,163
5	DE “comparative testing” or “control groups” or “experimental groups” or “matched groups” or “quasiexperimental design”	10,328
6	TX quasi‐experiment* or quasiexperiment* or “quasi experiment*” or “propensity scor*” or “control group*” or “controlled group*” or “treatment group*” or “comparison group*” or “wait‐list*” or “waiting list*” or “intervention group*” or “experimental group*” or “matched control*” or “matched group*” or “matched comparison*” or “experimental trial*” or “experimental design*” or “experimental method*” or “experimental stud*” or “experimental evaluation*” or “experimental test*” or “experimental assessment*” or placebo or “assessment only” or “treatment as usual” or “services as usual” or “care as usual” or “usual treatment*” or “usual care” or “usual service” or “usual services” or “standard treatment” or “standard service*” or “standard care” or “traditional treatment” or “traditional service*” or “traditional care” or “ordinary treatment” or “ordinary service*” or “ordinary care” or “comparison sample” or propensity‐matched or “control sample” or “intervention sample” or “assigned randomly” or “randomly assigned” or “random* control*”	33,104
7	AB “treatment outcome” AND AB group	111
8	S5 OR S6 OR S7	34,880
9	TX “random assignment” or “random allocation” or “randomi?ed control*” or “randomi?ed trial” or “randomi?ed design” or “randomi?ed method” or “randomi?ed evaluation” or “randomi?ed test” or “randomi?ed assessment”	2,328
10	TX ((“control trial”) or (“controlled trial”) or CCT)	1,117
11	S4 AND S8	49
12	S4 AND S9	4
13	S4 AND S10	1
14	S11 OR S12 OR S13	51
15	S11 OR S12 OR S13 Limiters ‐ Date Published: 20100101‐20131231	16

**Database:** Social Services Abstracts‎ (1979 ‐ current)og Sociological Abstracts‎ (1952 ‐ current) via ProQuest

**Dato:** 10. oktober 2014

**Antall treff:** 41

**Antall treff etter duplikatkontroll:** 21

**Table:**

**Set#**	**Searched for**	**Results**
S1	SU.EXACT.EXPLODE(“Homelessness”)	5041*
S2	Searched for: ab((evict* or homeless* or “housing excl*” or “living on the street*” or “residential stability” or “stable housing” or “street dwell*” or “Private dwell*” or “Improvised dwell*” or “Shelter dwell*” or “street liv*” or “Street life” or “street youth” or “street children” or “street people” or “marginally housed” or “precarious housing” or runaway* or “Run away from home” or “Running away” or “Ran away” or “Going missing” or “Bag lady” or Houseless* or Unhoused or “without a roof” or Roofless or “rough sleep*” or Destitute* or “Skid row*” or “Street people” OR “Street person*” OR “Street youth*” OR “Street child” OR “Street children” OR “Street life” OR “Street living” or “Sleep* rough” or “rough sleep*” or “emergency accommodation” OR “temporary accommodation” or “Insecure accommodation” OR “overcrowded accommodation” or “sleepers out”))	7860*
S3	Searched for: ab((“Housing first” OR “Pathways to Housing” OR “Homeless Veterans Reintegration Program” OR “Access to Community Care and Effective Services” OR “Support* Housing Program” OR “Housing and Urban Development–Veterans Affairs Supported Housing program” OR HUD‐VASH OR “Sober Transitional Housing” OR “sober house placement*” OR “Housing ladders” OR “Staircase housing” OR “low threshold housing” OR “Critical Time Intervention”))	75°
S4	Searched for: SU.EXACT.EXPLODE(“Homelessness”) OR ab((evict* OR homeless* OR “housing excl*” OR “living on the street*” OR “residential stability” OR “stable housing” OR “street dwell*” OR “Private dwell*” OR “Improvised dwell*” OR “Shelter dwell*” OR “street liv*” OR “Street life” OR “street youth” OR “street children” OR “street people” OR “marginally housed” OR “precarious housing” OR runaway* OR “Run away from home” OR “Running away” OR “Ran away” OR “Going missing” OR “Bag lady” OR Houseless* OR Unhoused OR “without a roof” OR Roofless OR “rough sleep*” OR Destitute* OR “Skid row*” OR “Street people” OR “Street person*” OR “Street youth*” OR “Street child” OR “Street children” OR “Street life” OR “Street living” OR “Sleep* rough” OR “rough sleep*” OR “emergency accommodation” OR “temporary accommodation” OR “Insecure accommodation” OR “overcrowded accommodation” OR “sleepers out”)) OR ab((“Housing first” OR “Pathways to Housing” OR “Homeless Veterans Reintegration Program” OR “Access to Community Care and Effective Services” OR “Support* Housing Program” OR “Housing and Urban Development–Veterans Affairs Supported Housing program” OR HUD‐VASH OR “Sober Transitional Housing” OR “sober house placement*” OR “Housing ladders” OR “Staircase housing” OR “low threshold housing” OR “Critical Time Intervention”))	8272*
S5	Searched for: ab((“quasi‐experimental” OR quasi‐experiment or quasiexperiment OR quasiexperimental OR “Propensity score” OR “propensity scores” OR “control group” OR “control groups” OR “controlled group” OR “controlled groups” OR “treatment group” OR “treatment groups” OR “comparison group” OR “comparison groups” OR “wait‐list” OR “waiting list” OR “wait‐lists” OR “waiting lists” OR “intervention group” OR “intervention groups” OR “experimental group” OR “experimental groups” OR “matched control” OR “matched groups” OR “matched comparison” OR “experimental trial” OR “experimental design” OR “experimental method” OR “experimental methods” OR “experimental study” OR “experimental studies” OR “experimental evaluation” OR “experimental test” OR “experimental tests” OR “experimental testing” OR “experimental assessment” OR placebo OR “assessment only” OR “treatment‐as‐usual” OR “services as usual” OR “care as usual” OR “usual treatment” OR “usual service” OR “usual services” OR “usual care” OR “standard treatment” OR “standard treatments” OR “standard service” OR “standard services” OR “standard care” OR “traditional treatment” OR “traditional service” OR “traditional care” OR “ordinary treatment” OR “ordinary service” Or “ordinary services” OR “ordinary care” OR “comparison sample” OR “propensity‐matched” OR “control sample” OR “intervention sample” OR “assigned randomly” OR “randomly assigned” OR “random* control*”))	13720*
S6	Searched for: ti((“random assignment” or “random allocation” or “randomi?ed control*” or “randomi?ed trial” or “randomi?ed design” or “randomi?ed method” or “randomi?ed evaluation” or “randomi?ed test” or “randomi?ed assessment” or randomi?ed))	932°
S7	Searched for: ab((“random assignment” OR “random allocation” OR “randomi?ed control*” OR “randomi?ed trial” OR “randomi?ed design” OR “randomi?ed method” OR “randomi?ed evaluation” OR “randomi?ed test” OR “randomi?ed assessment” OR randomi?ed))	3197°
S8	Searched for: ab((“Controlled trial” or “Control trial” or CCT))	752°
S9	Searched for: ab((“quasi‐experimental” OR quasi‐experiment OR quasiexperiment OR quasiexperimental OR “Propensity score” OR “propensity scores” OR “control group” OR “control groups” OR “controlled group” OR “controlled groups” OR “treatment group” OR “treatment groups” OR “comparison group” OR “comparison groups” OR “wait‐list” OR “waiting list” OR “wait‐lists” OR “waiting lists” OR “intervention group” OR “intervention groups” OR “experimental group” OR “experimental groups” OR “matched control” OR “matched groups” OR “matched comparison” OR “experimental trial” OR “experimental design” OR “experimental method” OR “experimental methods” OR “experimental study” OR “experimental studies” OR “experimental evaluation” OR “experimental test” OR “experimental tests” OR “experimental testing” OR “experimental assessment” OR placebo OR “assessment only” OR “treatment‐as‐usual” OR “services as usual” OR “care as usual” OR “usual treatment” OR “usual service” OR “usual services” OR “usual care” OR “standard treatment” OR “standard treatments” OR “standard service” OR “standard services” OR “standard care” OR “traditional treatment” OR “traditional service” OR “traditional care” OR “ordinary treatment” OR “ordinary service” OR “ordinary services” OR “ordinary care” OR “comparison sample” OR “propensity‐matched” OR “control sample” OR “intervention sample” OR “assigned randomly” OR “randomly assigned” OR “random* control*”)) OR ti((“random assignment” OR “random allocation” OR “randomi?ed control*” OR “randomi?ed trial” OR “randomi?ed design” OR “randomi?ed method” OR “randomi?ed evaluation” OR “randomi?ed test” OR “randomi?ed assessment” OR randomi?ed)) OR ab((“random assignment” OR “random allocation” OR “randomi?ed control*” OR “randomi?ed trial” OR “randomi?ed design” OR “randomi?ed method” OR “randomi?ed evaluation” OR “randomi?ed test” OR “randomi?ed assessment” OR randomi?ed)) OR ab((“Controlled trial” OR “Control trial” OR CCT))	15225*
S10	Searched for: (SU.EXACT.EXPLODE(“Homelessness”) OR ab((evict* OR homeless* OR “housing excl*” OR “living on the street*” OR “residential stability” OR “stable housing” OR “street dwell*” OR “Private dwell*” OR “Improvised dwell*” OR “Shelter dwell*” OR “street liv*” OR “Street life” OR “street youth” OR “street children” OR “street people” OR “marginally housed” OR “precarious housing” OR runaway* OR “Run away from home” OR “Running away” OR “Ran away” OR “Going missing” OR “Bag lady” OR Houseless* OR Unhoused OR “without a roof” OR Roofless OR “rough sleep*” OR Destitute* OR “Skid row*” OR “Street people” OR “Street person*” OR “Street youth*” OR “Street child” OR “Street children” OR “Street life” OR “Street living” OR “Sleep* rough” OR “rough sleep*” OR “emergency accommodation” OR “temporary accommodation” OR “Insecure accommodation” OR “overcrowded accommodation” OR “sleepers out”)) OR ab((“Housing first” OR “Pathways to Housing” OR “Homeless Veterans Reintegration Program” OR “Access to Community Care and Effective Services” OR “Support* Housing Program” OR “Housing and Urban Development–Veterans Affairs Supported Housing program” OR HUD‐VASH OR “Sober Transitional Housing” OR “sober house placement*” OR “Housing ladders” OR “Staircase housing” OR “low threshold housing” OR “Critical Time Intervention”))) AND (ab((“quasi‐experimental” OR quasi‐experiment OR quasiexperiment OR quasiexperimental OR “Propensity score” OR “propensity scores” OR “control group” OR “control groups” OR “controlled group” OR “controlled groups” OR “treatment group” OR “treatment groups” OR “comparison group” OR “comparison groups” OR “wait‐list” OR “waiting list” OR “wait‐lists” OR “waiting lists” OR “intervention group” OR “intervention groups” OR “experimental group” OR “experimental groups” OR “matched control” OR “matched groups” OR “matched comparison” OR “experimental trial” OR “experimental design” OR “experimental method” OR “experimental methods” OR “experimental study” OR “experimental studies” OR “experimental evaluation” OR “experimental test” OR “experimental tests” OR “experimental testing” OR “experimental assessment” OR placebo OR “assessment only” OR “treatment‐as‐usual” OR “services as usual” OR “care as usual” OR “usual treatment” OR “usual service” OR “usual services” OR “usual care” OR “standard treatment” OR “standard treatments” OR “standard service” OR “standard services” OR “standard care” OR “traditional treatment” OR “traditional service” OR “traditional care” OR “ordinary treatment” OR “ordinary service” OR “ordinary services” OR “ordinary care” OR “comparison sample” OR “propensity‐matched” OR “control sample” OR “intervention sample” OR “assigned randomly” OR “randomly assigned” OR “random* control*”)) OR ti((“random assignment” OR “random allocation” OR “randomi?ed control*” OR “randomi?ed trial” OR “randomi?ed design” OR “randomi?ed method” OR “randomi?ed evaluation” OR “randomi?ed test” OR “randomi?ed assessment” OR randomi?ed)) OR ab((“random assignment” OR “random allocation” OR “randomi?ed control*” OR “randomi?ed trial” OR “randomi?ed design” OR “randomi?ed method” OR “randomi?ed evaluation” OR “randomi?ed test” OR “randomi?ed assessment” OR randomi?ed)) OR ab((“Controlled trial” OR “Control trial” OR CCT)))	201°
S11	Searched for: (SU.EXACT.EXPLODE(“Homelessness”) OR ab((evict* OR homeless* OR “housing excl*” OR “living on the street*” OR “residential stability” OR “stable housing” OR “street dwell*” OR “Private dwell*” OR “Improvised dwell*” OR “Shelter dwell*” OR “street liv*” OR “Street life” OR “street youth” OR “street children” OR “street people” OR “marginally housed” OR “precarious housing” OR runaway* OR “Run away from home” OR “Running away” OR “Ran away” OR “Going missing” OR “Bag lady” OR Houseless* OR Unhoused OR “without a roof” OR Roofless OR “rough sleep*” OR Destitute* OR “Skid row*” OR “Street people” OR “Street person*” OR “Street youth*” OR “Street child” OR “Street children” OR “Street life” OR “Street living” OR “Sleep* rough” OR “rough sleep*” OR “emergency accommodation” OR “temporary accommodation” OR “Insecure accommodation” OR “overcrowded accommodation” OR “sleepers out”)) OR ab((“Housing first” OR “Pathways to Housing” OR “Homeless Veterans Reintegration Program” OR “Access to Community Care and Effective Services” OR “Support* Housing Program” OR “Housing and Urban Development–Veterans Affairs Supported Housing program” OR HUD‐VASH OR “Sober Transitional Housing” OR “sober house placement*” OR “Housing ladders” OR “Staircase housing” OR “low threshold housing” OR “Critical Time Intervention”))) AND (ab((“quasi‐experimental” OR quasi‐experiment OR quasiexperiment OR quasiexperimental OR “Propensity score” OR “propensity scores” OR “control group” OR “control groups” OR “controlled group” OR “controlled groups” OR “treatment group” OR “treatment groups” OR “comparison group” OR “comparison groups” OR “wait‐list” OR “waiting list” OR “wait‐lists” OR “waiting lists” OR “intervention group” OR “intervention groups” OR “experimental group” OR “experimental groups” OR “matched control” OR “matched groups” OR “matched comparison” OR “experimental trial” OR “experimental design” OR “experimental method” OR “experimental methods” OR “experimental study” OR “experimental studies” OR “experimental evaluation” OR “experimental test” OR “experimental tests” OR “experimental testing” OR “experimental assessment” OR placebo OR “assessment only” OR “treatment‐as‐usual” OR “services as usual” OR “care as usual” OR “usual treatment” OR “usual service” OR “usual services” OR “usual care” OR “standard treatment” OR “standard treatments” OR “standard service” OR “standard services” OR “standard care” OR “traditional treatment” OR “traditional service” OR “traditional care” OR “ordinary treatment” OR “ordinary service” OR “ordinary services” OR “ordinary care” OR “comparison sample” OR “propensity‐matched” OR “control sample” OR “intervention sample” OR “assigned randomly” OR “randomly assigned” OR “random* control*”)) OR ti((“random assignment” OR “random allocation” OR “randomi?ed control*” OR “randomi?ed trial” OR “randomi?ed design” OR “randomi?ed method” OR “randomi?ed evaluation” OR “randomi?ed test” OR “randomi?ed assessment” OR randomi?ed)) OR ab((“random assignment” OR “random allocation” OR “randomi?ed control*” OR “randomi?ed trial” OR “randomi?ed design” OR “randomi?ed method” OR “randomi?ed evaluation” OR “randomi?ed test” OR “randomi?ed assessment” OR randomi?ed)) OR ab((“Controlled trial” OR “Control trial” OR CCT))) AND yr(2010‐2019)	41°

* Duplicates are removed from your search, but included in your result count.

° Duplicates are removed from your search and from your result count.

**Database: Web of Science**

**Dato:** 13. oktober 2014

**Antall treff:** 435

**Antall treff etter duplikatkontroll:** 297

**Table:**

**Set**	**Results**	**Search**
# 1	**Approximately** **19,193**	**TOPIC:** (Homeless* or Homelessness) *Timespan=All years* *Search language=Auto*
# 2	**Approximately** **66,880**	**TOPIC:** ((housing excl* or living on the street* or residential stability or stable housing or street dwell* or Private dwell* or Improvised dwell* or Shelter dwell* or street liv* or Street life or street youth or street children or street people or marginally housed or precarious housing or Housing first or runaway* or Run away from home or Running away or Ran away or Going missing or Bag lady or Houseless* or Unhoused or without a roof or Roofless or rough sleeper or rough sleepers or Rough sleeping or Destitute* or Skid row* or Street people OR Street person* OR Street youth* OR Street child OR Street children OR Street life OR Street living or Sleeping rough or sleep rough or rough sleep or emergency accommodation OR temporary accommodation or Insecure accommodation OR overcrowded accommodation or sleepers out)) *Timespan=All years* *Search language=Auto*
# 3	**Approximately** **31,468**	**TOPIC:** (evict* or homeless* or “housing excl*” or “living on the street*” or “residential stability” or “stable hou‐sing” or “street dwell*” or “Private dwell*” or “Im‐provised dwell*” or “Shelter dwell*” or “street liv*” or “Street life” or “street youth” or “street children” or “street people” or “marginally housed” or “precarious housing” or “Housing first” or runaway* or “Run away from home” or “Running away” or “Ran away” or “Going missing” or “Bag lady” or Houseless* or Unhoused or “without a roof” or Roofless or “rough sleeper” or “rough sleepers” or “Rough sleeping” or Destitute* or “Skid row*” or “Street people” or “Street person*” or “Street youth*” or “Street child” or “Street children” or “Street life” or “Street living” or “Sleeping rough” or “sleep rough” or “rough sleep” or “emergency accommodation” or “temporary accommodation” or “Insecure accommodation” or “overcrowded accommodation” or “sleepers out”) *Timespan=All years* *Search language=Auto*
# 4	**Approximately** **15,295**	**TITLE:** (evict* or homeless* or “housing excl*” or “living on the street*” or “residential stability” or “stable hou‐sing” or “street dwell*” or “Private dwell*” or “Im‐provised dwell*” or “Shelter dwell*” or “street liv*” or “Street life” or “street youth” or “street children” or “street people” or “marginally housed” or “precarious housing” or “Housing first” or runaway* or “Run away from home” or “Running away” or “Ran away” or “Going missing” or “Bag lady” or Houseless* or Unhoused or “without a roof” or Roofless or “rough sleeper” or “rough sleepers” or “Rough sleeping” or Destitute* or “Skid row*” or “Street people” or “Street person*” or “Street youth*” or “Street child” or “Street children” or “Street life” or “Street living” or “Sleeping rough” or “sleep rough” or “rough sleep” or “emergency accommodation” or “temporary accommodation” or “Insecure accommodation” or “overcrowded accommodation” or “sleepers out”) *Timespan=All years* *Search language=Auto*
# 5	**264**	**TOPIC:** (“Housing first” or “Pathways to Housing” or “Homeless Veterans Reintegration Program” or “Access to Community Care and Effective Services and Supports” or “Support* Housing Program” or “Housing and Urban Development–Veterans Affairs Supported Housing program” or “HUD‐VASH” or “Sober Transitional Housing and Employment Project” or “sober house placement*” or “Housing ladders” or “Staircase housing” or “low threshold housing” or “Critical Time Intervention”) *Timespan=All years* *Search language=Auto*
# 6	**124**	**TITLE:** (“Housing first” or “Pathways to Housing” or “Homeless Veterans Reintegration Program” or “Access to Community Care and Effective Services and Supports” or “Support* Housing Program” or “Housing and Urban Development Veterans Affairs Supported Housing program” or “HUD‐VASH” or “Sober Transitional Housing and Employment Project” or “sober house placement*” or “Housing ladders” or “Staircase housing” or “low threshold housing” or “Critical Time Intervention”) *Timespan=All years* *Search language=Auto*
# 7	**Approximately** **6,950**	**TITLE:** (housing excl* or living on the street* or residential stability or stable housing or street dwell* or Private dwell* or Improvised dwell* or Shelter dwell* or street liv* or Street life or street youth or street children or street people or marginally housed or precarious housing or Housing first or runaway* or Run away from home or Running away or Ran away or Going missing or Bag lady or Houseless* or Unhoused or without a roof or Roofless or rough sleeper or rough sleepers or Rough sleeping or Destitute* or Skid row* or Street people OR Street person* OR Street youth* OR Street child OR Street children OR Street life OR Street living or Sleeping rough or sleep rough or rough sleep or emergency accommodation OR temporary accommodation or Insecure accommodation OR overcrowded accommodation or sleepers out) *Timespan=All years* *Search language=Auto*
# 8	**Approximately** **84,323**	#7 OR #6 OR #5 OR #4 OR #3 OR #2 OR #1 *Timespan=All years* *Search language=Auto*
# 9	**607**	**TITLE:** (“comparative testing” or “control groups” or “experimental groups” or “matched groups” or “quasiexperimental design”) *Timespan=All years* *Search language=Auto*
# 10	**Approximately** **128,323**	**TOPIC:** (“comparative testing” or “control groups” or “experimental groups” or “matched groups” or “quasiexperimental design”) *Timespan=All years* *Search language=Auto*
# 11	**Approximately** **1,804,246**	**TOPIC:** (quasi‐experiment* or quasiexperiment* or “quasi experiment*” or “propensity scor*” or “control group*” or “controlled group*” or “treatment group*” or “comparison group*” or “wait‐list*” or “waiting list*” or “intervention group*” or “experimental group*” or “matched control*” or “matched group*” or “matched comparison*” or “experimental trial*” or “experimental design*” or “experimental method*” or “experimental stud*” or “experimental evaluation*” or “experimental test*” or “experimental assessment*” or placebo or “assessment only” or “treatment as usual” or “services as usual” or “care as usual” or “usual treatment*” or “usual care” or “usual service” or “usual services” or “standard treatment” or “standard service*” or “standard care” or “traditional treatment” or “traditional service*” or “traditional care” or “ordinary treatment” or “ordinary service*” or “ordinary care” or “comparison sample” or propensity‐matched or “control sample” or “intervention sample” or “assigned randomly” or “randomly assigned” or “random* control*”) *OR* **TITLE:**(quasi‐experiment* or quasiexperiment* or “quasi experiment*” or “propensity scor*” or “control group*” or “controlled group*” or “treatment group*” or “comparison group*” or “wait‐list*” or “waiting list*” or “intervention group*” or “experimental group*” or “matched control*” or “matched group*” or “matched comparison*” or “experimental trial*” or “experimental design*” or “experimental method*” or “experimental stud*” or “experimental evaluation*” or “experimental test*” or “experimental assessment*” or placebo or “assessment only” or “treatment as usual” or “services as usual” or “care as usual” or “usual treatment*” or “usual care” or “usual service” or “usual services” or “standard treatment” or “standard service*” or “standard care” or “traditional treatment” or “traditional service*” or “traditional care” or “ordinary treatment” or “ordinary service*” or “ordinary care” or “comparison sample” or propensity‐matched or “control sample” or “intervention sample” or “assigned randomly” or “randomly assigned” or “random* control*”) *Timespan=All years* *Search language=Auto*
# 12	**Approximately** **997,570**	**TOPIC:** (“random assignment” or “random allocation” or “randomi?ed control*” or “randomi?ed trial” or “randomi?ed design” or “randomi?ed method” or “randomi?ed evaluation” or “randomi?ed test” or “randomi?ed assessment”) *OR* **TOPIC:** (control trial or controlled trial or CCT) *OR* **TITLE:** (control trial or controlled trial or CCT) *OR* **TITLE:** (“random assignment” or “random allocation” or “randomi?ed control*” or “randomi?ed trial” or “randomi?ed design” or “randomi?ed method” or “randomi?ed evaluation” or “randomi?ed test” or “randomi?ed assessment”) *Timespan=All years* *Search language=Auto*
# 13	**Approximately** **2,228,257**	#12 OR #11 OR #10 OR #9 *Timespan=All years* *Search language=Auto*
# 14	**Approximately** **4,883**	#13 AND #8 *Timespan=All years* *Search language=Auto*
# 15	**1,485**	#13 AND #8 **Refined by: RESEARCH DOMAINS:** (SOCIAL SCIENCES) *Timespan=All years* *Search language=Auto*
# 16	**435**	#13 AND #8 **Refined by: RESEARCH DOMAINS:** (SOCIAL SCIENCES) AND **PUBLICATION YEARS:** (2013 OR 2012 OR 2011 OR 2014 OR 2010) *Timespan=All years* *Search language=Auto*

Databasen ASSIA har vi ikke tilgang til slik at vi ikke får gjort et oppdateringsøk I denne basen.

### Search strategy 2010

**Table:**

**ASSIA via CSA 2010‐02‐15** **Insatser för att minska hemlöshet** **Sökning gjord av Maja Kärrman Fredriksson och Hanna Olofsson i samarbete med Sten Anttila**			
Söknr	Termtyp *)	Söktermer	Antal ref. **)
1.	DE	“homelessness” or “homeless adolescent girls” or “homeless boys” or “homeless children” or “homeless elderly people” or “homeless families” or “homeless men” or “homeless mentally ill men” or “homeless mentally ill people” or “homeless mentally ill women” or “homeless mentally ill young people” or “homeless mothers” or “homeless older people” or “homeless people” or “homeless pregnant women” or “homeless women” or “homeless young men” or “homeless young people” or “homeless young women”	1829
2.	KW	evict* or homeless* or “housing excl*” or “living on the street*” or “residential stability” or “stable housing” or “street dwell*” or “Private dwell*” or “Improvised dwell*” or “Shelter dwell*” or “street liv*” or “Street life” or “street youth” or “street children” or “street people” or “marginally housed” or “precarious housing” or “Housing first” or runaway* or “Run away from home” or “Running away” or “Ran away” or “Going missing” or “Bag lady” or Houseless* or Unhoused or “without a roof” or Roofless or “rough sleeper” or “rough sleepers” or “Rough sleeping” or Destitute* or “Skid row*” or “Street people” OR “Street person*” OR “Street youth*” OR “Street child” OR “Street children” OR “Street life” OR “Street living” or “Sleeping rough” or “sleep rough” or “rough sleep” or “emergency accommodation” OR “temporary accommodation” or “Insecure accommodation” OR “overcrowded accommodation” or “sleepers out”	3156
3.	KW	“Housing first” OR “Pathways to Housing” OR “Homeless Veterans Reintegration Program” OR “Access to Community Care and Effective Services and Supports” OR “Support* Housing Program” OR “Housing and Urban Development–Veterans Affairs Supported Housing program” OR “HUD‐VASH” OR “Sober Transitional Housing and Employment Project” OR “sober house placement*” OR “Housing ladders” OR “Staircase housing” OR “low threshold housing” OR “Critical Time Intervention”	21
4.		1 or 2 or 3	3157
5.	DE	(“control groups” or “experimental treatment” or “placebos” or “propensity” or “random sampling” or “random testing” or “randomization” or “samples” or “waiting lists”)	505
6.	FT(AB)	“treatment outcome” near group	22
7.	KW	KW=(quasi‐experiment* or quasiexperiment* or (quasi experiment*)) or KW=((propensity scor*) or (control group*) or (controlled group*)) or KW=((treatment group*) or (comparison group*) or wait‐list*) or KW=((waiting list*) or (intervention group*) or (experimental group*)) or KW=((matched control*) or (matched group*) or (matched comparison*)) or KW=((experimental trial*) or (experimental design*) or (experimental method*)) or KW=((experimental stud*) or (experimental evaluation*) or (experimental test*)) or KW=((experimental assessment*) or placebo or (assessment only)) or KW=((treatment as usual) or (services as usual) or (care as usual)) or KW=((usual treatment*) or (usual care) or (usual service)) or KW=((usual services) or (standard treatment) or (standard service*)) or KW=((standard care) or (traditional treatment) or (traditional service*)) or KW=((traditional care) or (ordinary treatment) or (ordinary service*)) or KW=((ordinary care) or (comparison sample) or propensity‐matched) or KW=((control sample) or (intervention sample) or (assigned randomly)) or KW=((randomly assigned) or (random* control*))	15685
8.		5 or 6 or 7	15848
9.		KW=((“random assignment”) or (“random allocation”) or (“randomi?ed control*”)) or KW=((“randomi?ed trial”) or (“randomi?ed design”) or (“randomi?ed method”)) or KW=((“randomi?ed evaluation”) or (“randomi?ed test”) or (“randomi?ed assessment”))	5180
10.		KW=((“control trial”) or (“controlled trial”) or CCT)	2839
11.		4 and 8	90
12.		4 and 9	21
13.		4 and 10	12
14.		11 or 12 or 13	**94 (**95 innan dublettkontroll i ASSIA)

*ASSIA:*

*)

DE= Kontrollerade ämnesord från ASSIA:s thesaurus

KW=Fritexttermer som söks samtidigt i Title (TI), Abstract (AB), Descriptor (DE), och Identifier (ID) fälten

FT = Fritextterm/er

**Table:**

**CINAHL via EBSCO 2010‐02‐15** **Insatser för att minska hemlöshet** **Sökning gjord av Maja Kärrman Fredriksson och Hanna Olofsson i samarbete med Sten Anttila**			
Söknr	Termtyp *)	Söktermer	Antal ref. **)
1.		(MH “Homeless Persons”) or (MH “Homelessness”)	3406
2.		(AB evict* or homeless* or housing excl* or living on the street* or residential stability or stable housing or street dwell* or Private dwell* or Improvised dwell* or Shelter dwell* or street liv* or Street life or street youth or street children or street people or marginally housed or precarious housing or Housing first or runaway* or Run away from home or Running away or Ran away or Going missing or Bag lady or Houseless* or Unhoused or without a roof or Roofless or rough sleeper or rough sleepers or Rough sleeping or Destitute* or Skid row* or Street people OR Street person* OR Street youth* OR Street child OR Street children OR Street life OR Street living or Sleeping rough or sleep rough or rough sleep or emergency accommodation OR temporary accommodation or Insecure accommodation OR overcrowded accommodation or sleepers out) or (TI evict* or homeless* or housing excl* or living on the street* or residential stability or stable housing or street dwell* or Private dwell* or Improvised dwell* or Shelter dwell* or street liv* or Street life or street youth or street children or street people or marginally housed or precarious housing or Housing first or runaway* or Run away from home or Running away or Ran away or Going missing or Bag lady or Houseless* or Unhoused or without a roof or Roofless or rough sleeper or rough sleepers or Rough sleeping or Destitute* or Skid row* or Street people OR Street person* OR Street youth* OR Street child OR Street children OR Street life OR Street living or Sleeping rough or sleep rough or rough sleep or emergency accommodation OR temporary accommodation or Insecure accommodation OR overcrowded accommodation or sleepers out) or (TI evict* or homeless* or housing excl* or living on the street* or residential stability or stable housing or street dwell* or Private dwell* or Improvised dwell* or Shelter dwell* or street liv* or Street life or street youth or street children or street people or marginally housed or precarious housing or Housing first or runaway* or Run away from home or Running away or Ran away or Going missing or Bag lady or Houseless* or Unhoused or without a roof or Roofless or rough sleeper or rough)	3591
3.		AB Housing first OR Pathways to Housing OR Homeless Veterans Reintegration Program OR Access to Community Care and Effective Services and Supports OR Support* Housing Program OR Housing and Urban Development–Veterans Affairs Supported Housing program OR HUD‐VASH OR Sober Transitional Housing and Employment Project OR sober house placement* OR Housing ladders OR Staircase housing OR low threshold housing OR Critical Time Intervention	47
4.		1 or 2 or 3	4654
5.		(quasi‐experimental OR quasi‐experiment or quasiexperiment OR quasiexperimental OR Propensity score OR propensity scores OR “control group” OR “control groups” OR “controlled group” OR “controlled groups” OR “treatment group” OR “treatment groups” OR “comparison group” OR “comparison groups” OR “wait‐list” OR “waiting list” OR “wait‐lists” OR “waiting lists” OR “intervention group” OR “intervention groups” OR “experimental group” OR “experimental groups” OR “matched control” OR “matched groups” OR “matched comparison” OR “experimental trial” OR “experimental design” OR “experimental method” OR “experimental methods” OR “experimental study” OR “experimental studies” OR “experimental evaluation” OR “experimental test” OR “experimental tests” OR “experimental testing” OR “experimental assessment” OR placebo OR “assessment only” OR treatment‐as‐usual OR “services as usual” OR “care as usual” OR “usual treatment” OR “usual service” OR “usual services” OR “usual care” OR “standard treatment” OR “standard treatments” OR “standard service” OR “standard services” OR “standard care” OR “traditional treatment” OR “traditional service” OR “traditional care” OR “ordinary treatment” OR “ordinary service” OR “ordinary services” OR “ordinary care” OR “comparison sample” OR propensity‐matched OR control sample OR intervention sample OR assigned randomly OR randomly assigned OR random* control*)	133751
6.		(MH “Quasi‐Experimental Studies”) or (MH “Nonequivalent Control Group”) or (MH “Control Group”) or (MH “Experimental Studies+”) or (MH “Waiting Lists”) or (MH “Matched‐Pair Analysis”) or (MH “Clinical Trials+”) or (MH “Placebos”) or (MH “Random Assignment”) or (MH “Random Sample+”) or (MH “Matched‐Pair Analysis”) or (MH “Case Control Studies”)	148303
7.		(MH “Treatment Outcomes”) and (AB group)	12404
8.		5 or 6 or 7	225058
9.		TI random assignment or TI random allocation or TI randomi?ed control* or TI randomi?ed trial or TI randomi?ed design or TI randomi?ed method or TI randomi?ed evaluation or TI randomi?ed test or TI randomi?ed assessment or TI randomi?ed or (AB random assignment or AB random allocation or AB randomi?ed control* or AB randomi?ed trial or AB randomi?ed design or AB randomi?ed method or AB randomi?ed evaluation or AB randomi?ed test or AB randomi?ed assessment) or (KW random assignment or KW random allocation or KW randomi?ed control* or KW randomi?ed trial or KW randomi?ed design or KW randomi?ed method or KW randomi?ed evaluation or KW randomi?ed test or KW randomi?ed assessment)TI random assignment or TI random allocation or TI randomi?ed control* or TI randomi?ed trial or TI randomi?ed design or TI randomi?ed method or TI randomi?ed evaluation or TI randomi?ed test or TI randomi?ed assessment or TI randomi?ed or (AB random assignment or AB random allocation or AB randomi?ed control* or AB randomi?ed trial or AB randomi?ed design or AB randomi?ed method or AB randomi?ed evaluation or AB randomi?ed test or AB randomi?ed assessment) or (KW random assignment or KW random	30760
10.		(MH “Clinical Trials+”)	81036
11.		TX Controlled trial or TX Control trial	33210
12.		11 or 12	100604
13.		4 Limiters ‐ Publication Type: Clinical Trial	53
14.		4 and 8	498
15.		4 and 9	43
16.		4 and 12	111
17.		13 or 14 or 15 or 16	**500**

*)

DE = Descriptor (fastställt ämnesord i databasen)

FT/default fält = fritextsökning i fälten för “all authors, all subjects, all keywords, all title info (including source title) and all abstracts”

FT/TI, AB = fritextsökning i fälten för titel och abstract

ZX = Methodology

+ = Termen söks inklusive de mer specifika termerna som finns underordnade

**Table:**

**Cochrane Library via Wiley Interscience 2010‐02‐10** **Insatser för att minska hemlöshet** **Sökning gjord av Maja Kärrman Fredriksson och Hanna Olofsson i samarbete med Sten Anttila**			
Söknr	Termtyp *)	Söktermer	Antal ref. **)
Population			
1.	MeSH	(“Homeless Youth”[Mesh] OR “Homeless Persons”[Mesh])	CDSR/0 DARE/4 CENTRAL/151 METHODS STUDIES/ HTA/0 EED/31
2.	FT (TI, KW, AB)	Homeless*	CDSR/5 DARE/4 CENTRAL/275 METHODS STUDIES/6 HTA/0 EED/31
Intervention			
3.	FT (TI, KW, AB)	”Housing first” OR ”Pathways to Housing” OR ”Homeless Veterans Reintegration Program” OR ”Access to Community Care and Effective Services and Supports” OR ”Support* Housing Program” OR ”Housing and Urban Development–Veterans Affairs Supported Housing program” OR ”HUD‐VASH” OR ”Sober Transitional Housing and Employment Project” OR ”sober house placement*” OR ”Housing ladders” OR ”Staircase housing” OR ”low threshold housing” OR “Critical time intervention”	CDSR/ DARE/ CENTRAL/11 HTA/ EED/
4.		1 OR 2 OR 3	CDSR/**5** DARE/**4** CENTRAL/**276** METHODS STUDIES/6 HTA/0 EED/31

*)

MeSH = Medical subject headings (fastställda ämnesord i Medline/PubMed, som även används i Cochrane library)

FT = Fritextterm/er

Explode = Termen söks inklusive de mer specifika termerna som finns underordnade

Only this term = Endast den termen söks, de mer specifika, underordnade termerna utesluts

**)

CDSR = The Cochrane Database of Systematic Reviews

CENTRAL=Cochrane Central Register of Controlled Trials

DARE = Database of Abstracts of Reviews of Effects

HTA = Health Technology Assessment Database

EED = NHS Economic Evaluation Database

**Table:**

**ASSIA via CSA 2010‐02‐16** **Insatser för att minska hemlöshet** **Sökning gjord av Maja Kärrman Fredriksson och Hanna Olofsson i samarbete med Sten Anttila**			
Söknr	Termtyp *)	Söktermer	Antal ref. **)
Population			
1.	DE	“homeless people”	1514
2.	KW	evict* or homeless* or “housing excl*” or “living on the street*” or “residential stability” or “stable hou‐sing” or “street dwell*” or “Private dwell*” or “Im‐provised dwell*” or “Shelter dwell*” or “street liv*” or “Street life” or “street youth” or “street children” or “street people” or “marginally housed” or “precarious housing” or “Housing first” or runaway* or “Run away from home” or “Running away” or “Ran away” or “Going missing” or “Bag lady” or Houseless* or Unhoused or “without a roof” or Roofless or “rough sleeper” or “rough sleepers” or “Rough sleeping” or Destitute* or “Skid row*” or “Street people” or “Street person*” or “Street youth*” or “Street child” or “Street children” or “Street life” or “Street living” or “Sleeping rough” or “sleep rough” or “rough sleep” or “emergency accommodation” or “temporary accommodation” or “Insecure accommodation” or “overcrowded accommodation” or “sleepers out”	2606
Intervention			
3.	KW	“Housing first” or “Pathways to Housing” or “Homeless Veterans Reintegration Program” or “Access to Community Care and Effective Services and Supports” or “Support* Housing Program” or “Housing and Urban Development–Veterans Affairs Supported Housing program” or “HUD‐VASH” or “Sober Transitional Housing and Employment Project” or “sober house placement*” or”Housing ladders” or “Staircase housing” or “low threshold housing” or “Critical Time Intervention”	6
4.		1 or 2 or 3	2609
Studiedesign‐kvasiexperimentella			
5.	DE	“comparative testing” or “control groups” or “experimental groups” or “matched groups” or “quasiexperimental design”	5804
6.	KW	quasi‐experiment* or quasiexperiment* or “quasi experiment*” or “propensity scor*” or “control group*” or “controlled group*” or “treatment group*” or “comparison group*” or “wait‐list*” or “waiting list*” or “intervention group*” or “experimental group*” or “matched control*” or “matched group*” or “matched comparison*” or “experimental trial*” or “experimental design*” or “experimental method*” or “experimental stud*” or “experimental evaluation*” or “experimental test*” or “experimental assessment*” or placebo or “assessment only” or “treatment as usual” or “services as usual” or “care as usual” or “usual treatment*” or “usual care” or “usual service” or “usual services” or “standard treatment” or “standard service*” or “standard care” or “traditional treatment” or “traditional service*” or “traditional care” or “ordinary treatment” or “ordinary service*” or “ordinary care” or “comparison sample” or propensity‐matched or “control sample” or “intervention sample” or “assigned randomly” or “randomly assigned” or “random* control*”	24397
7.		AB=(“treatment outcome” NEAR group)	14
8.		5 or 6 or 7	25883
Studiedesign‐RCT			
9.	KW	“random assignment” or “random allocation” or “randomi?ed control*” or “randomi?ed trial” or “randomi?ed design” or “randomi?ed method” or “randomi?ed evaluation” or “randomi?ed test” or “randomi?ed assessment”	1043
Studiedesign‐Controlled trials			
10.	KW	((“control trial”) or (“controlled trial”) or CCT)	439
11.		4 and 8	**32**
12.		4 and 9	0
13.		4 and 10	0

*)

DE= Kontrollerade ämnesord från ERIC:s thesaurus

KW=Fritexttermer som söks samtidigt i Title (TI), Abstract (AB), Descriptor (DE), och Identifier (ID) fälten

FT = Fritextterm/er

**Table:**

**PsycInfo via EBSCO 100209** **Insatser för att minska hemlöshet** **Sökning gjord av Hanna Olofsson och Maja Kärrman Fredriksson i samarbete med Sten Anttila**			
Söknr	Termtyp *)	Söktermer	Antal ref. **)
1.		DE Homeless or DE Homeless Mentally Ill or DE Runaway behaviour	3894
2.		TX evict* or TX homeless* or TX housing excl* or TX living on the street* or TX residential stability or TX stable housing or TX street dwell* or TX Private dwell* or TX Improvised dwell* or TX Shelter dwell* or TX street liv* or TX Street life or TX street youth or TX street children or TX street people or TX marginally housed or TX precarious housing or TX Housing first or TX runaway* or TX Run away from home or TX Running away or TX Ran away or TX Going missing or TX Bag lady or TX Houseless* or TX Unhoused or TX without a roof or TX Roofless or TX rough sleeper or rough sleepers or Rough sleeping or TX Destitute* or TX Skid row* or TX Street people OR TX Street person* OR TX Street youth* OR TX Street child OR TX Street children OR TX Street life OR TX Street living or TX Sleeping rough or sleep rough or TX rough sleep or TX emergency accommodation OR TX temporary accommodation or TX Insecure accommodation OR TX overcrowded accommodation or TX sleepers out	8589
3.		1 OR 2	8589
4.		TX Housing first OR TX Pathways to Housing OR TX Homeless Veterans Reintegration Program OR TX Access to Community Care and Effective Services and Supports OR TX Support* Housing Program OR TX Housing and Urban Development–Veterans Affairs Supported Housing program OR TX HUD‐VASH OR TX Sober Transitional Housing and Employment Project OR TX sober house placement* OR TX Housing ladders OR TX Staircase housing OR TX low threshold housing OR TX Critical Time Intervention	144
5.		3 OR 4	8622
Studiedesign –Kvasiexperimentella			
6.	TX	(quasi‐experimental OR quasi‐experiment or quasiexperiment OR quasiexperimental OR Propensity score OR propensity scores OR “control group” OR “control groups” OR “controlled group” OR “controlled groups” OR “treatment group” OR “treatment groups” OR “comparison group” OR “comparison groups” OR “wait‐list” OR “waiting list” OR “wait‐lists” OR “waiting lists” OR “intervention group” OR “intervention groups” OR “experimental group” OR “experimental groups” OR “matched control” OR “matched groups” OR “matched comparison” OR “experimental trial” OR “experimental design” OR “experimental method” OR “experimental methods” OR “experimental study” OR “experimental studies” OR “experimental evaluation” OR “experimental test” OR “experimental tests” OR “experimental testing” OR “experimental assessment” OR placebo OR “assessment only” OR treatment‐as‐usual OR “services as usual” OR “care as usual” OR “usual treatment” OR “usual service” OR “usual services” OR “usual care” OR “standard treatment” OR “standard treatments” OR “standard service” OR “standard services” OR “standard care” OR “traditional treatment” OR “traditional service” OR “traditional care” OR “ordinary treatment” OR “ordinary service” OR “ordinary services” OR “ordinary care” OR “comparison sample” OR propensity‐matched OR control sample OR intervention sample OR assigned randomly OR randomly assigned OR random* control*)	152053
7.		(DE Treatment outcomes) and AB group*	4933
Studiedesign –RCT			
8.		TI random assignment or TI random allocation or TI randomi?ed control* or TI randomi?ed trial or TI randomi?ed design or TI randomi?ed method or TI randomi?ed evaluation or TI randomi?ed test or TI randomi?ed assessment or TI randomi?ed or (AB random assignment or AB random allocation or AB randomi?ed control* or AB randomi?ed trial or AB randomi?ed design or AB randomi?ed method or AB randomi?ed evaluation or AB randomi?ed test or AB randomi?ed assessment) or (KW random assignment or KW random allocation or KW randomi?ed control* or KW randomi?ed trial or KW randomi?ed design or KW randomi?ed method or KW randomi?ed evaluation or KW randomi?ed test or KW randomi?ed assessment)	14755
Studiedesign –Controlled trials			
9.		TX Controlled trial or TX Control trial or TX CCT	117221
10.		6 OR 7 OR 8 OR 9	159069
11.		5 AND 10	533
12.		11 AND Limits humans	**532**^2^

2 En del brus om sömnlöshet…

*)

DE = Descriptor (fastställt ämnesord i databasen)

FT/default fält = fritextsökning i fälten för “all authors, all subjects, all keywords, all title info (including source title) and all abstracts”

FT/TI, AB = fritextsökning i fälten för titel och abstract

ZX = Methodology

+ = Termen söks inklusive de mer specifika termerna som finns underordnade

**Table:**

**PubMed via NCBI 2010‐02‐09** **Insatser för att minska hemlöshet** **Sökning gjord av Maja Kärrman Fredriksson och Hanna Olofsson i samarbete med Sten Anttila**			
Söknr	Termtyp *)	Söktermer	Antal ref. **)
Population			
1.	MeSH	(“Homeless Youth”[Mesh] OR “Homeless Persons”[Mesh])	4986
2.	FT(TIAB)	evicted[tiab] or eviction[tiab] or homeless*[tiab] or housing excluded[tiab] or housing exclusion[tiab] or living on the street*[tiab] or residential stability[tiab] or stable housing[tiab] or street dwelling[tiab] or street dwellers[tiab] or Private dwelling[tiab] or Improvised dwelling[tiab] or improvised dwellings[tiab] or Shelter dwellers[tiab] or shelter dwellings[tiab] or street liv*[tiab] or Street life[tiab] or street youth[tiab] or street children[tiab] or street people[tiab] or marginally housed[tiab] or precarious housing[tiab] or Housing first[tiab] or runaway*[tiab] or Run away from home[tiab] or Running away[tiab] or Ran away[tiab] or Going missing[tiab] or Bag lady[tiab] or Houseless*[tiab] or Unhoused[tiab] or without a roof[tiab] or Roofless[tiab] or rough sleep*[tiab] or Destitute*[tiab] or Skid row*[tiab] or Street people[tiab] or street person*[tiab] or street youth[tiab] or street child*[tiab] or street life[tiab] or street living[tiab] or sleeping rough[tiab] or sleepers rough[tiab] or emergency accommodation[tiab] or temporary accommodation[tiab] or insecure accommodation[tiab] or overcrowded accomodation[tiab] or sleepers out[tiab]^3^	6865
3.		1 OR 2	8134
Intervention			
4.	FT(TIAB)	Housing first[tiab] OR Pathways to Housing[tiab] OR Homeless Veterans Reintegration Program[tiab] OR Access to Community Care and Effective Services and Supports[tiab] OR Support* Housing Program[tiab] OR Housing and Urban Development–Veterans Affairs Supported Housing program[tiab] OR HUD‐VASH[tiab] OR Sober Transitional Housing and Employment Project[tiab] OR sober house placement*[tiab] OR Housing ladders[tiab] OR Staircase housing[tiab] OR low threshold housing[tiab] OR Critical Time Intervention[tiab]^4^	42
5.		3 OR 4	8148
Studiedesign‐kvasiexperimentella			
6.		((“Propensity Score”[Mesh] OR “Control Groups”[Mesh]) OR “Case‐Control Studies”[Mesh]) OR “Matched‐Pair Analysis”[Mesh]	448124
7.		quasi‐experimental[tiab] OR quasi‐experiment[tiab] or quasiexperiment[tiab] OR quasiexperimental[tiab] OR Propensity scor*[tiab] OR control group[tiab] OR control groups[tiab] OR controlled group[tiab] OR controlled groups[tiab] OR treatment group[tiab] OR treatment groups[tiab] OR comparison group[tiab] OR comparison groups[tiab] OR wait‐list[tiab] OR waiting list[tiab] OR wait‐lists[tiab] OR waiting lists[tiab] OR intervention group[tiab] OR intervention groups [tiab] OR experimental group[tiab] OR experimental groups[tiab] OR matched control[tiab] OR matched groups[tiab] OR matched comparison[tiab] OR experimental trial[tiab] OR experimental design[tiab] OR experimental method[tiab] OR experimental methods[tiab] OR experimental study[tiab] OR experimental studies[tiab] OR experimental evaluation[tiab] OR experimental test[tiab] OR experimental tests[tiab] OR experimental testing[tiab] OR experimental assessment[tiab] OR placebo[tiab] OR assessment only[tiab] OR treatment‐as‐usual[tiab] OR services as usual[tiab] OR care as usual[tiab] OR usual treatment[tiab] OR usual service[tiab] OR usual services[tiab] OR usual care[tiab] OR standard treatment[tiab] OR standard treatments[tiab] OR standard service[tiab] OR standard services[tiab] OR standard care[tiab] OR traditional treatment[tiab] OR traditional service[tiab] OR traditional services[tiab] OR traditional care[tiab] OR ordinary treatment[tiab] OR ordinary therapy[tiab] OR ordinary service[tiab] Or ordinary services[tiab] OR ordinary care[tiab] OR comparison sample[tiab] OR propensity‐match*[tiab] OR control sample[tiab] OR intervention sample[tiab] OR assigned randomly[tiab] OR randomly assigned[tiab]^5^	513603
8.		(“Treatment Outcome”[Mesh] AND (group[tiab] OR groups[tiab]))	108628
9.		6 OR 7 OR 8	982804
Studiedesign‐RCT			
10.		Limits: Meta‐Analysis, Randomized Controlled Trial	302673
11.		“Randomized Controlled Trials as Topic”[Mesh] OR “Randomized Controlled Trial “[Publication Type] OR “Random Allocation”[MeSH] OR randomized controlled trial[tiab] OR randomised controlled trial[tiab] OR (randomised[ti] AND trial[ti]) OR (randomized[ti] AND trial[ti]) OR RCT[ti]	393309
12.		10 OR 11	407864
Studiedesign‐Controlled trials			
13.		Limits: Clinical Trial	590056
14.		Controlled trial[tiab] or Control trial[tiab] or CCT[tiab]	45128
15.		13 OR 14	602516
16.		5 AND 9	638
17.		5 AND 12	234
18.		5 AND 15	324
19.		16 OR 17 OR 18 Limits: Humans	**819**

3 Följande termer gav inga träffar: housing excluded[tiab], housing exclusion[tiab], Improvised dwelling[tiab], improvised dwellings[tiab], shelter dwellings[tiab], marginally housed[tiab], Run away from home[tiab], Going missing[tiab], without a roof[tiab], sleepers rough[tiab], overcrowded accomodation[tiab], sleepers out[tiab].

4 Följande termer gav inga träffar: Pathways to Housing[tiab], Homeless Veterans Reintegration Program[tiab], Access to Community Care and Effective Services and Supports[tiab], Housing and Urban Development‐Veterans Affairs Supported Housing program[tiab], Sober Transitional Housing and Employment Project[tiab], housing ladder[tiab], Housing ladders[tiab], Staircase housing[tiab], low threshold housing[tiab].

5 Följande termer gav inga träffar: ordinary service[tiab], ordinary services[tiab], assigned randomly[tiab].

*)

MeSH = Medical subject headings (fastställda ämnesord i Medline/PubMed)

FT = Fritextterm/er

SB = PubMeds filter

för systematiska översikter (systematic[sb])

för alla MeSH‐indexerade artiklar (medline[sb])

Tiab= söker i title‐ och abstractfälten

Exp = Termen söks inklusive de mer specifika termerna som finns underordnade

NoExp = Endast den termen söks, de mer specifika, underordnade termerna utesluts

MAJR = MeSH Major Topic (termen beskriver det huvudsakliga innehållet i artikeln)

**Table:**

**Sociological Abstracts & Social Services Abstract via CSA 100204** **Insatser för att minska hemlöshet** **Sökning gjord av Hanna Olofsson och Maja Kärrman Fredriksson i samarbete med Sten Anttila**			
Söknr	Termtyp *)	Söktermer	Antal ref. **)
1.		DE=(homelessness or runaways or “skid row” or squatters)	3840
2.		AB=(evict* or homeless* or “housing excl*” or “living on the street*” or “residential stability” or “stable housing” or “street dwell*” or “Private dwell*” or “Improvised dwell*” or “Shelter dwell*” or “street liv*” or “Street life” or “street youth” or “street children” or “street people” or “marginally housed” or “precarious housing” or runaway* or “Run away from home” or “Running away” or “Ran away” or “Going missing” or “Bag lady” or Houseless* or Unhoused or “without a roof” or Roofless or “rough sleep*” or Destitute* or “Skid row*” or “Street people” OR “Street person*” OR “Street youth*” OR “Street child” OR “Street children” OR “Street life” OR “Street living” or “Sleep* rough” or “rough sleep*” or “emergency accommodation” OR “temporary accommodation” or “Insecure accommodation” OR “overcrowded accommodation” or “sleepers out”)	5416
3.		AB=(“Housing first” OR “Pathways to Housing” OR “Homeless Veterans Reintegration Program” OR “Access to Community Care and Effective Services” OR “Support* Housing Program” OR “Housing and Urban Development–Veterans Affairs Supported Housing program” OR HUD‐VASH OR “Sober Transitional Housing” OR “sober house placement*” OR “Housing ladders” OR “Staircase housing” OR “low threshold housing” OR “Critical Time Intervention”)^6^	20
4.		1 OR 2 OR 3	6021
5.		(AB=(“quasi‐experimental” OR quasi‐experiment or quasiexperiment OR quasiexperimental OR “Propensity score” OR “propensity scores” OR “control group” OR “control groups” OR “controlled group” OR “controlled groups” OR “treatment group” OR “treatment groups” OR “comparison group” OR “comparison groups” OR “wait‐list” OR “waiting list” OR “wait‐lists” OR “waiting lists” OR “intervention group” OR “intervention groups” OR “experimental group” OR “experimental groups” OR “matched control” OR “matched groups” OR “matched comparison” OR “experimental trial” OR “experimental design” OR “experimental method” OR “experimental methods” OR “experimental study” OR “experimental studies” OR “experimental evaluation” OR “experimental test” OR “experimental tests” OR “experimental testing” OR “experimental assessment” OR placebo OR “assessment only” OR “treatment‐as‐usual” OR “services as usual” OR “care as usual” OR “usual treatment” OR “usual service” OR “usual services” OR “usual care” OR “standard treatment” OR “standard treatments” OR “standard service” OR “standard services” OR “standard care” OR “traditional treatment” OR “traditional service” OR “traditional care” OR “ordinary treatment” OR “ordinary service” Or “ordinary services” OR “ordinary care” OR “comparison sample” OR “propensity‐matched” OR “control sample” OR “intervention sample” OR “assigned randomly” OR “randomly assigned” OR “random* control*”)	8527
Studiedesign‐ RCT			
6.		TI=(“random assignment” or “random allocation” or “randomi?ed control*” or “randomi?ed trial” or “randomi?ed design” or “randomi?ed method” or “randomi?ed evaluation” or “randomi?ed test” or “randomi?ed assessment” or randomi?ed) or AB=(“random assignment” or “random allocation” or “randomi?ed control*” or “randomi?ed trial” or “randomi?ed design” or “randomi?ed method” or “randomi?ed evaluation” or “randomi?ed test” or “randomi?ed assessment”) or AB=(“random assignment” or “random allocation” or “randomi?ed control*” or “randomi?ed trial” or “randomi?ed design” or “randomi?ed method” or “randomi?ed evaluation” or “randomi?ed test” or “randomi?ed assessment”)	1071
Studiedesign‐ CCT			
7.		AB=(“Controlled trial” or “Control trial” or CCT)	273
8.		6 OR 7 OR 8	8945
9.		4 AND 8	132 (**122** unika)
10.		Limiters ‐ Population Group: Human	
11.			
12.			
13.			
14.			

6 Eftersom SA & SSAs KW‐sökning innefattar även referenslistorna, så gjordes fritextssökningen endast i abstract.

*)

DE= Kontrollerade ämnesord från ASSIA:s thesaurus

KW=Fritexttermer som söks samtidigt i Title (TI), Abstract (AB), Descriptor (DE), och Identifier (ID) fälten

FT = Fritextterm/er

## Appendix 3: Additional tables

### Table 3.1. Characteristics of case management models

**Table:**

** **	**Standard Case Management**	**Broker Case management**	**Intensive Case Management**	**Assertive Community Treatment**	**Critical Time Intervention**
**Focus of services**	Coordination of services	Purchase and coordination of services	Comprehensive approach	Comprehensive approach	Targeted to continuity of care
**Duration of services**	Time limited	Time limited	Ongoing	Ongoing	Time limited
**Average caseload, no.**	35		15	15	25
**Outreach**	No	No	Yes	Yes	Yes
**Coordination or service priviosn**	Coordination	Coordination	Service provision	Service provision	Service provision and coordination
**Responsibility for clients’ care**	Case manager	Case manager	Case manager	Multidiscplinary team	Case manager
**Importance of client‐case manager relationship**	Somewhat important	Not important	Important	Important	Important
**Intensity of services**	Low	Low	High	High	High

## Appendix 4: Secondary outcomes

### Table 5.1. High intensity case management compared to usual services for adults with mental illness and/or substance abuse problems – secondary outcomes

**Table:**

**Study**	**Outcome**	**Longest follow‐up**	**Intervention**	**Comparison**	**Result**
Employment outcomes					
Cox 1998 (50)	Days of employment (mean, SD) Number of days in past 30 days worked	18 months	N=105 3.1 ± 7.4	N=82 2.5± 6.3	Group effect: ns Time effect: F=3.85, 2/184 df, p=.023)
Lehman 1997 (60)	Employment QOLI – employment related subscales Range: 1‐7	12 months	N=77 NR	N=75 NR	No between group differences
Morse 1992 (39)(39)	Monthly income (Mean $ (SD)) – final score	12 months	N=37 498 (897)	N=65 405.54 (735.27)	Calculated effect size for comparison group after combining two control groups (Final score) MD=92.45, SE=173.39, p=0.57
Nordentoft 2010 (70)	No work and not in Education (number of events, %) (N=482 (%))	12 months	N=227 107 (42)	N=192 121 (53)	OR=0.31 (95%CI 0.2,0.5), p=0.01
Rosenheck 2003 (71)	Employment Addiction Severity Index ‐ Employment index score (mean)	36 months	N=90 0.187	N=188 0.187	t(2v3) = 0.01, p= .99
Rosenheck 2003 (71)	Employment – self report Number of days worked in past 30 days (mean)	36 months	N=90 6.82	N=188 6.71	t(2 vs 3)=0.13, p=0.89
Rosenheck 2003 (71)	Total income Amount of income (mean $)	36 months	N=90 684	N=188 717	t(2 vs 3)=0.67, p=0.50
**Physical health outcomes**					
Bell 2015 (44)	Death – dichotomous (any=1, none=0) % Records from	Up to 24 months	N=557 Unadjusted difference (pre to post) 7	N=563 Unadjusted difference (pre to post) 8	Unadjusted p=0.68
Bell 2015 (44)	Death – aOR (95 CI%)	Up to 24 months	N=557 NR	N=563 NR	Adjusted (for risk score, age, race/ethnicity, sex, serious mental illness, alcohol and drug treatment need, weighted by number of months of eligibility during postperiod) Difference in difference OR=0.65 [0.39, 1.09],p=0.10
Lehman 1997 (60)	General health Medical outcome study 36‐item short form health survey (MOS SF‐36) (adjusted for race mean (SEM)) Range: 0‐100, 100=best	12 months	N=77 45.9 (1.7)	N=75 39.1 (1.8)	Adjusted mean for race as covariate 12 mos: ANCOVA F=7.45, df=1.147, p=.01 (In favour of ACT group) ANCOVA for SF‐36 total score revealed significant group and time effects, but no significant group x time effect.
Lehman 1997 (60)	Physical functioning Medical outcome study 36‐item short form health survey (MOS SF‐36) (adjusted for race mean (SEM)) Range: 0‐100, 100=best	12 months	N=77 84.5 (3.5)	N=75 83.6 (3.7)	ns
Lehman 1997 (60)	Bodily pain Medical outcome study 36‐item short form health survey (MOS SF‐36) (adjusted for race mean (SEM)) Range: 0‐100, 100=best	12 months	N=77 68.7 (4.6)	N=75 64.7 (4.8)	ns
Nordentoft 2010 (70)	Death (number of events, %) (N=547 (%)	12 months	N=227 1 (0.4)	N=192 3 (1)	OR=0.32 (95%CI 0.03, 3.2), p=0.3
Rosenheck 2003 (71)	Health Addiction Severity Index ‐ Medical index score (mean)	36 months	N=90 0.28	N=188 0.27	ICM vs SC t= 0.15, p= .88
Mental health outcomes					
Bond 1990 (45)	Areas of difficulty (areas of difficulty checklist‐20 item)	12 months	NR	NR	t(53)=2.64, p<.05 in favour of ACT, multivariate analysis on 3 subscales: F(3,51)=3.14,p<.05 in favour of ACT
Garety 2006 (54)	Overall functioning (mean, SD) Global Assessment of Function (GAF) Range: 0‐100	18 months	Baseline (N=56): 46.5 (15.3) 18 mos (N=54): 64.1 (15.3)	Baseline (N=43): 42.2 (14.8) 18 mos (N=44): 55.3 (15.1)	ANCOVA= ‐8.72 (95%CI 15.46 to ‐1.98), p=.01 With inverse probability weights Coefficient (95%CI), p ‐8.38 (‐15.61 to ‐1.16), 0.02
Garety 2006 (54)	Relapse (full or partial) (%, n/N)	18 months	N=61 30%, 18/61	N=61 48%, 29/61	OR (95%CI) =0.46 (0.21 to 0.97), p=0.042 (excludes those who never recovered) Adjusted OR (95%CI) = 0.55 (0.24 to 1‐26), p=1.57 (adjusted for sex, past episode and ethnicity)
Garety 2006 (54)	Symptoms of schizophrenia (mean, SD) The Positive and Negative Syndrome Scale (PANSS) ‐ total Range: 30‐210 item, higher score indicates greater symptom severity	18 months	N=55 51.2 (15.2)	N=44 58.9 (14.2)	ANCOVA =5.74 (95%CI ‐0.30 to 11.79), p=0.06 With inverse probability weights Coefficient (95%CI), p 4.90 (‐0.96 to10.76), p=0.10
Garety 2006 (54)	Positive symptoms of schizophrenia (mean, SD) The Positive and Negative Syndrome Scale (PANSS) ‐ positive Range: 7‐49, higher score indicates greater symptom severity	18 months	N=55 11.8 (5.1)	N=44 14.0 (5.9)	ANCOVA =1.32 (95%CI ‐1.01 to 3.65), p=0.26 With inverse probability weights Coefficient (95%CI), p 1.42 (‐0.73 to 3.56), 0.19
Garety 2006 (54)	Negative symptoms of schizophrenia (mean, SD) The Positive and Negative Syndrome Scale (PANSS) ‐ Negative Range: 7‐49, higher score indicates greater symptom severity Follow‐up: 18 mos	18 months	N=55 11.9 (5.1)	N=44 14.8 (5.4)	ANCOVA =2.30 (95%CI 0.02 to 4.57), p= 0.048 With inverse probability weights Coefficient (95%CI), p 1.41 (‐0.97 to 3.79), 0.24
Garety 2006 (54)	General symptoms of schizophrenia (mean, SD) The Positive and Negative Syndrome Scale (PANSS) ‐ General Range: 16‐112, higher score indicates greater symptom severity	18 months	N=55 27.4 (7.6)	N=44 30.2 (7.0)	ANCOVA = 2.19 (95%CI ‐0.75 to 5.13), p=0.14 With inverse probability weights Coefficient (95%CI), p 1.97 (‐0.87 to 4.82), 0.17
Garety 2006 (54)	Depression (mean, SD) Calgary Depression Scale Range: 0‐27	18 months	N=55 2.7 (3.3)	N=44 2.7 (3.5)	ANCOVA= 0.93 (95%CI ‐0.47 to 2.33), p=0.19 With inverse probability weights Coefficient (95%CI), p 0.82 (‐0.62 to 2.36), 0.25
Killaspy 2006 (59)	Psychiatric symptoms Expanded Brief Psychiatric Rating Scale (EBPRS) (mean SD) Range: 27‐72, higher score indicates greater symptom severity	18 months	32.9 (9.0)	33.5 (8.6)	MD= ‐0.6 (95%CI ‐3.3, 2.1), p=.66
Lehman 1997 (60)	Mental illness symptoms Colorado Symptom Index (CSI) – total (adjusted for race mean (SEM)) Range: 15‐70, lower score indicates more symptoms	12 months	N=77 4.12 (SEM=0.11)	N=75 3.77 (SEM=0.11)	ANCOVA F=5.04, df=1.121, p=.03 ANCOVA for CSI total score revealed significant group and time effects, but no significant group x time effect.
Morse 1992 (39)	Psychiatric symptoms (mean Brief Symptoms Inventory score (SD))	12 months	N=37 0.95 (0.76)	N=65 0.09 (0.74)	Calculated by combining two control groups MD=0.86, SE=0.15, p=0.000
Morse 2006 (69)(69)	Mental health Brief Psychiatric Rating Sale (mean (SD)) Range: 1‐168, higher score indicates greater symptom severity	24 months	N=100 1.78 (0.51)	N=49 1.86 (0.60)	Calculated by combining two intervention groups MD=‐0.08 (SE=0.10), p=0.40
Nordentoft 2010 (70)	Positive psychotic symptoms (number of events where score >3, %) (N=417 (%)) Scale for Assessment of Positive Symptoms (SAPS) Range: 0‐5, higher score indicates greater severity	12 months	N=227 23 (10)	N=192 39 (20)	OR=0.35 (95%CI 0.2, 0.6), p=0.001
Nordentoft 2010 (70)	Negative psychotic symptoms (number of events where score >3, %) (N=417 (%)) Scale for Assessment of Negative Symptoms (SANS) Range: 0‐5, higher score indicates greater severity	12 months	N=227 50 (22)	N=192 67 (35)	OR=0.49 (95%CI 0.3,0.8), p=0.002
Nordentoft 2010 (70)	Functioning (Symptom) Global assessment of functioning‐Symptom (S530) (number of events where score <30, %) (N=476 (%)) Range: 1‐100, higher score indicates better functioning	12 months	N=227 24 (10)	N=192 38 (17)	OR=0.55 (95%CI 0.3, 1.0), p= 0.04
Nordentoft 201 Nordentoft 2010 (70)0	Functioning (Disability) Global assessment of functioning‐Symptom (GAF‐D530) (number of events where score <30, %) (N=476 (%)) Range: 1‐100, higher score indicates better functioning	12 months	N=227 17 (7)	N=192 22 (9)	OR=0.71 (95%CI 0.4, 1.4), p=0.3
Rosenheck 2003 (71)	Mental health Addiction Severity Index ‐ Psychiatric index score (mean)	36 months	N=90 0.26	N=188 0.24	ICM vs SC t= 0.95, p= .34
Substance use outcomes					
Bell 2015 (44)	Drug/alcohol treatment Including residential, outpatient, opiate substitution Records from Division of Behavioral Health and Recovery (DBHR)	Up to 24 months	Unadjusted difference (pre to post) 1	Unadjusted difference (pre to post) 2	Unadjusted p=0.43 Adjusted (for risk score, age, race/ethnicity, sex, serious mental illness, alcohol and drug treatment need, weighted by number of months of eligibility during postperiod) Difference in difference OR=0.92 [0.65, 1.30], p=0.62
Cox 1998 (50)	Days of drinking (mean, SD) Number of days in past 30 days used alcohol Follow‐up: 18 mos	18 months	N=108 Baseline: NA 6 mos: 102 ± 65 12 mos: 78 ± 64 18 mos: 70 ± 58	N=85 Baseline: NA 6 mos: 123 ± 57 12 mos: 97 ± 62 18 mos: 99 ± 60	Group effect: F=6.97, 1/190 df, p=.009 Time effect: F=4.43, 2/190 df, p=.013) t‐test effect size (18 months) = 0.32 (small) In favour of ICM
Cox 1998 (50)	Alcohol composite score (mean, SD) Addiction severity Index – alcohol subscale Range: 0‐9, lower score indicates less severity	18 months	N=108 Baseline: 0.67 ± 0.23 6 mos: 0.45 ± 0.30 12 mos: 0.41 ± 0.31 18 mos: 0.39 ± 0.29	N=85 Baseline: 0. 67 ± 0.21 6 mos: 0.53 ± 0.26 12 mos: 0.46 ± 0.29 18 mos: 0.46 ± 0.30	Group effect: F=4.90, 1/190 df, p=.028 Time effect: F=3.49, 2/190 df, p=.032) In favour of ICM
Killaspy 2006 (59)	Alcohol use Self‐report (mean number of participants reporting alcohol use/N)	18 months	25/124	21/115	x^2^=0.14, p=.71
Killaspy 2006 (59)	Illicit drug use Self‐report (mean number of participants reporting illicit drug use/N)	18 months	29/124	25/115	x^2^=0.64, p=.42
Morse 1992 (39)	Alcohol consumption (mean (SD) ounces per week) National Institute on Alcohol Abuse and Alcoholism Index	12 month	N=37 2.83 (9.11)	N=65 0.74 (1.63)	Calculated after combining control groups MD=2.09, SE=1.5, p=0.07
Morse 2006 (69)	Substance use – interviewer rating 2 5‐point scales (drug and alcohol) Range: Higher score indicates greater severity	24 months	N=100 2.73 (1.20)	N=49 2.62 (1.15)	Calculated by combining means and SD from two intervention groups MD=0.11 (SE=0.20) p=0.60
Morse 2006 (69)	Substance use – self report Days of substance use in past 90 days	24 months	N=100 7.07 (8.47)	N=49 6.42 (7.84)	Calculated by combining means and SD from two intervention groups MD=0.65 (SE=1.40), p=0.65
Nordentoft 2010 (70)	Substance misuse or dependence syndrome present (number of events, %) Unclear (self report or urine tests) (N=507 (%))	12 months	N=227 41 (16)	N=192 53 (22)	OR=0.54 (95%CI 0.3, 0.9, p= 0.03
Rosenheck 2003 (71)	Alcohol use – self report Number of days drank to intoxication (mean)	36 months	1.95	1.71	t(2 vs 3)=0.55, p=0.58
Rosenheck 2003 (71)	Alcohol use Addiction severity index – alcohol index score (ASI) (mean)	36 months	N=90 0.151	N=188 0.121	ICM vs SC t= 1.59, p=.11
Rosenheck 2003 (71)	Drug use Addiction severity index – drug index score (ASI) (mean)	36 months	N=90 0.065	N=188 0.063	ICM vs SC t= 0.26, p= .79
Quality of life outcomes					
Garety 2006 (54)	Quality of life (means, SD) Manchester Short Assessment of Quality of life Range: 12‐84, higher scores indicate better quality of life	18 months	N=52 59.2 (12.6)	N=40 53.3 (12.4)	ANCOVA= ‐5.96 (95%CI ‐11.19 to ‐0.74), p=0.026 With inverse probability weights Coefficient (95%CI), p ‐6.60 (‐11.59 to ‐1.61), 0.010
Garety 2006 (54)	Support network (mean, SD) Significant Others Scale (self report number and type of people who provide practical and emotional support)	18 months	N=57 2.4 (1.20)	N=50 1.71 (1.06)	T=2.77, df=84, p=0.01
Killaspy 2006 (59)	Quality of life Manchester Short Assessment of Quality of Life (MANSA) (mean, SD) Range: 0–126, low score better)	18 months	N=91 4.5 (1.0)	N=67 4.4 (0.9)	MD= 0.1 (95%CI ‐0.2, 0.4), p=0.56
Social support/functioning outcomes					
Killaspy 2006 (59)	Social functioning Life Skills Profile (mean, SD) Range: high scores indicate high levels of life skills	18 months	N=124 119 (16.4)	N=115115 (19.7)	MD= 4.3 (95%CI ‐0.3, 8.9), p=0.07
Killaspy 2006 (59)	Engagement Homeless Engagement and Acceptance Scale (HEAS) (mean, SD) Range: high score indicates higher levels of engagement)	18 months	N=124 9.1 (3.3)	N=115 8.0 (3.8)	MD= 1.1 (95%CI 0.1, 1.9), p=.03
Lehman 1997 (60)	Social functioning Medical outcome study 36‐item short form health survey (MOS SF‐36) (adjusted for race mean (SEM)) Range: 0‐100, 100=best	12 months	N=77 42.7 (2.1)	N=75 42.4 (2.3)	ns
Morse 1992 (39)	Alienation Unspecified measurement tool (mean (SD))	12 months	N=37 0.74 (0.29)	N=65 0.74 (0.30)	Calculated after combining control groups MD=‐0.002, SE=0.06, p=0.97
Morse 1992 (39)	Interpersonal adjustment Personality and Social Network Adjustment Scale (mean score (SD))	12 months	N=37 2.88 (0.75)	N=65 2.92 (0.63)	Calculated after combining control groups MD=‐0.04, SE=0.15, p=0.75
Morse 1992 (39)	Natural network Modified version of Barrera's (1980) Arizona Social Support Interview Schedule (ASSIS) (avg score (SD)) Range: 1‐9, higher score indicate more support average score equally weighted at 3, 6, 9 and 12 month follow‐up	12 months	N=37 4.08 (3.75)	N=65 4.9815 (4.8382)	Calculated after combining control groups MD=‐0.90, SE=0.86, p=0.33
Morse 1992 (39)	Professional network Modified version of Barrera's (1980) Arizona Social Support Interview Schedule (ASSIS) (avg score (SD)) Range: 1‐9, higher score indicate more support average score equally weighted at 3, 6, 9 and 12 month follow‐up	12 months	N=37 2.58 (3.41)	N=65 1.4946 (1.9009)	Calculated after combining control groups MD=1.09, SE=0.61, p=0.04
Morse 1997 (40)	Natural network (avg score (SD)) – self rated Modified version of Barrera's (1980) Arizona Social Support Interview Schedule (ASSIS) Range: 1‐9, higher score indicate more support	18 months (average score at 15 and 21 month assessments)	ACT‐CW: 2.32 (1.84) ACT: 2.08 (1.46)	0.98 (1.15)	No significant difference No effect of time or group by time.
Morse 1997 (40)	Professional network (avg score (SD)) – self rated Modified version of Barrera's (1980) Arizona Social Support Interview Schedule (ASSIS) Range: 1‐9, higher score indicate more support	18 months (average score at 15 and 21 month assessments)	ACT‐CW: 3.43 (2.72) ACT: 3.61 (2.69)	4.02 (3.11)	F(2,126)=10.47, p<.0001 Post‐hoc analysis indicated that both ACT teams had larger professional networks than the brokered condition. No effect of time or group by time.
Morse 1997 (40)	Material assistance (avg score (SD)) (number of people who provided material assistance, e.g. money, transport) Range: 4‐12, higher scores indicating greater satisfaction	18 months (average score at 15 and 21 month assessments)	ACT‐CW: 1.54 (1.40) ACT: 1.49 (1.17)	0.84 (0.90)	F(2,122)=7.20, p<.001 Post‐hoc analysis indicate that clients in the ACT‐CW condition reported significantly more material support than clients in both the ACT‐Only and brokered conditions; clients in the ACT‐Only condition reported having more material support than clients in the brokered condition.
Morse 1997 (40)	Advice (avg score (SD)) (number of people who gave advice) Range: 4‐12, higher scores indicating greater satisfaction	18 months (average score at 15 and 21 month assessments)	ACT‐CW: 1.88 (1.65) ACT: 1.91 (1.63)	1.57 (1.60)	No significant effect
Morse 1997 (40)	Emotional support (avg score (SD)) (number of people who provided emotional support) Range: 4‐12, higher scores indicating greater satisfaction	18 months (average score at 15 and 21 month assessments)	ACT‐CW: 2.13 (1.81) ACT: 1.63 (1.61)	1.32 (1.25)	No significant effect
Morse 1997 (40)	Recreation (avg score (SD)) (number of people with whom one socialized) Range: 4‐12, higher scores indicating greater satisfaction	18 months (average score at 15 and 21 month assessments)	ACT‐CW: 2.42 (2.23) ACT: 2.60 (2.37)	2.46 (2.87)	No significant effect
Morse 1997 (40)	Conflict (avg score (SD)) (number conflictual relationships in the network) Range: 4‐12, higher scores indicating greater satisfaction (number of people who gave advice)	18 months (average score at 15 and 21 month assessments)	ACT‐CW: 1.06 (1.08) ACT: 1.32 (1.18)	1.24 (1.46)	No significant effect
Morse 1997 (40)	Alienation (avg score (SD)) Alienation scale (Bahr & Caplow 1973) – mean item scores on alienation scales Range: 0‐1, higher scores indicating more alienation	18 months (average score at 15 and 21 month assessments)	ACT‐CW: 0.64 (0.32) ACT: 0.61 (0.31)	0.67 (0.29)	No significant effect
Morse 1997 (40)	Interpersonal Adjustment (avg score (SD)) Personality and Social Network Adjustment Scale (Clark 1968) – mean scores across four items (get along with same and opposite sex, family and others in general) assessment) Range: 0‐4, higher scores indicate better relations	18 months (average score at 15 and 21 month assessments)	ACT‐CW: 2.92 (0.60) ACT: 2.73 (0.60)	Baseline: 2.77 (0.79) 6 mos: 2.71 (0.68) 18 mos: 2.73 (0.72)	No significant effect
Morse 1997 (40)	Network satisfaction (avg score (SD)) Modified version of Barrera's (1980) Arizona Social Support Interview Schedule (ASSIS)) ‐ total satisfaction score Range: 4‐12, higher scores indicating greater satisfaction	18 months (average score at 15 and 21 month assessments)	ACT‐CW: 9.09 (2.14) ACT: 9.35 (1.80)	9.18 (2.10)	No significant effect
Morse 1997 (40)	Natural network (avg score (SD)) – interviewer rated Modified version of Barrera's (1980) Arizona Social Support Interview Schedule (ASSIS) Range: 1‐9, higher score indicate more support	18 months (average score at 15 and 21 month assessments)	ACT‐CW: 3.44 (2.02) ACT: 4.00 (1.89)	3.42 (1.80)	No significant differences. Interivewer's rating of the adequacy of natural support correlated 0.30 with clients report of number people providing material support.
Morse 1997 (40)	Professional network (avg score (SD)) – interviewer rated Modified version of Barrera's (1980) Arizona Social Support Interview Schedule (ASSIS) Range: 1‐9, higher score indicate more support	18 months (average score at 15 and 21 month assessments)	ACT‐CW: 5.78 (2.09) ACT: 6.54 (2.08)	3.66 (2.10)	F(2, 118)=19.51, p<.0001 Interviewrs believed that cilents in both of the CT conditions had better professional support than clients in brokered case management. Interivewer's rating of the adequacy of support from professionals correlated 0.51 with amount of contact with the program, 0.30 with clients report of number people providing material support and .22 with the number of people providing emotional support.
Rosenheck 2003 (71)	Social network size Number of people they feel close to (mean)	36 months	N=90 9.3	N=188 10.1	t=0.88, p=0.38
Rosenheck 2003 (71)	Social contacts Number of social contacts (mean)	36 months	N=90 30.4	N=188 36.5	t=1.74, p=0.08
Rosenheck 2003 (71)	Social support Number of people who would support (mean)	36 months	N=90 6.54	N=188 7.11	t=1.15, p=0.25
Rosenheck 2003 (71)	Satisfaction with family relations Subjective Quality of Life Interview – family score (mean) Range: 1‐7, higher score indicates greater quality of life	36 months	N=90 4.16	N=188 4.25	t=0.62, p=0.53
Criminal activity outcomes					
Bell 2015 (44)	Criminal arrests – dichotomous (any=1, none=0), % participants Arrest records from state patrol	up to 24 months	Unadjusted difference (pre to post) 0	Unadjusted difference (pre to post) 3	Unadjusted p=0.22 Adjusted (for risk score, age, race/ethnicity, sex, serious mental illness, alcohol and drug treatment need, weighted by number of months of eligibility during postperiod) Difference in difference OR=0.81 [0.52, 1.28], p=0.38
Bell 2015 (44)	Criminal arrests ‐ mean number per 1000 mos (SD), n Arrest records from state patrol	up to 24 months	Unadjusted difference (pre to post) ‐2 (58)	Unadjusted difference (pre to post) ‐4 (95)	Unadjusted p=0.38 Adjusted (for risk score, age, race/ethnicity, sex, serious mental illness, alcohol and drug treatment need, weighted by number of months of eligibility during postperiod) Difference in difference n= 4.7 [‐3.8, 13.2], p=0.28
Bell 2015 (44)	Criminal convictions – dichotomous (any=1, none=0), % Arrest records from state patrol	up to 24 months	Unadjusted difference (pre to post) 0	Unadjusted difference (pre to post) ‐4	Unadjusted p=0.01 Adjusted (for risk score, age, race/ethnicity, sex, serious mental illness, alcohol and drug treatment need, weighted by number of months of eligibility during postperiod) Difference in difference OR=0.1.95 [1.10, 3.44], p=0.02
Bell 2015 (44)	Criminal convictions ‐ mean number per 1000 mos (SD), n Arrest records from state patrol	up to 24 months	Unadjusted difference (pre to post) ‐9 (73)	Unadjusted difference (pre to post) ‐16 (110)	Unadjusted p<0.01 Adjusted (for risk score, age, race/ethnicity, sex, serious mental illness, alcohol and drug treatment need, weighted by number of months of eligibility during postperiod) Difference in difference n= 8.9 [‐1.5, 19.3], p=0.09
Bond 1990 (45)	Police contact during 6 months prior to follow‐up	12 month	4/34 (12%)	11/22 (50%)	x^2^(1)=9.96, p<.01
Bond 1990 (45)	Arrests during 6 months prior to follow‐up	12 month	3/34	1/22	Not reported
Clarke 2000 (48)	Number of clients arrested (%) Follow‐up: unknown	24 months	14/114 (12%)	9/49 (18%)	ns
Cox 1998 (50)	Number of days to first arrest (mean, SE) Follow‐up: unknown	24 months	588 days (SE=38, 95%CI 513, 662)	554 days (SE=80, 95%CI 391‐705)	Breslow4.03, df=1, p<.05
Killaspy 2006 (59)	Arrested (mean number of participants arrested/N)	18 months	23/124	25/115	x^2^=0.38, p=.54
Killaspy 2006 (59)	Prison (mean number of participants in prison/N)	18 months	3/124	4/115	x^2^=0.24, p=.63
Lehman 1997 (60)	In jail Number of days in jail (adjusted for race mean, SEM)	12 months	N=77 Adjusted (SEM) Mean days = 9.0 (6.1)	N=75 Adjusted (SEM) Mean days = 19.3 (6.2)	ACT subjects spent on average 53% fewer days in jail, ns.
Rosenheck 2003 (71)	Arrests – major crimes Number of arrests (mean)	36 months	N=90 0.20	N=188 0.23	ICM vs SC t= 0.82, p= .41
Rosenheck 2003 (71)	Arrests – minor crimes Number of arrests (mean)	36 months	N=90 0.21	N=188 0.22	ICM vs SC t= 0.27, p= .79
Satisfactioon with life					
Bond 1990 (45)	Quality of life (Life Satisfaction checklist‐32 item)	12 month	NR	NR	t(50)=1.76, p<.10 in favour of ACT
Lehman 1997 (60)(60)	Life satisfaction Quality of life (QOLI)—life satisfaction subscales (subjective measures) Range: 1‐7 per scale	12 months	N=77 NR	N=75 NR	MANCOVA F=1.19, df=8.103, p=.32
Lehman 1997 (60)	Satisfaction with family relations QOLI subscale Range: 1‐7	12 months	N=77 NR	N=75 NR	No between group differences
Lehman 1997 (60)	Satisfaction with social relations QOLI subscale Range: 1‐7	12 months	N=77 NR	N=75 NR	No between group differences
Rosenheck 2003 (71)	Satisfaction with housing Subjective Quality of Life Interview – housing score (mean) Range: 1‐7, higher score indicates greater satisfaction	36 months	4.02	4.12	t(2 vs 3)=0.90, p=0.37
Rosenheck 2003 (71)	Satisfaction with disposable income Subjective Quality of Life Interview – finances score (mean) Range: 1‐7, higher score indicates greater quality of life	36 months	2.93	3.12	t(2 vs 3)=1.41, p=0.16
Rosenheck 2003 (71)	Satisfaction with health Subjective Quality of Life Interview – health score (mean) Range: 1‐7, higher score indicates greater quality of life	36 months	4.18	4.36	t(2 vs 3)=1.60, p=0.11
Rosenheck 2003 (71)	Satisfaction with social relations Subjective Quality of Life Interview – social score (mean) Range: 1‐7, higher score indicates greater quality of life	36 months	4.04	4.20	t(2 vs 3)=1.38, p=0.17

### Table 4.2. High intensity case management compared to usual services for disadvantaged youth – secondary outcomes

**Table:**

**Study**	**Outcome**	**Longest follow‐up**	**Intervention**	**Comparison**	**Result**
Grace 2014 (46)	Employment Income dollars from employment during previous 12 mos (past 12 mos) (mean $ (SD))	24 months	N=196 24 mos: 2562 (10180)	N=174 24 mos: 1392 (4250)	F=2.85, n^2^_p_=0.009
Grace 2014 (46) (Borland 2013(86))	Employed at anniversary of entry to trial ‐ self‐report (mean nr participants) YP^4^ Interview data	24 months	N= 235 M=0.15	N=187 M=0.12	Diff=0.03, t=0.70
Grace 2014 (46) (Borland 2013(86))	Self‐rated health – good (mean nr participants) YP^4^ Interview data	24 months	N= 235 M=0.44	N=187 M=0.53	Diff=‐0.09, t=1.25
Grace 2014 (46) (Borland 2013(86))	Self‐rated health – bad (mean nr participants) YP^4^ Interview data	24 months	N= 235 M=0.18	N=187 M=0.14	Diff=0.04, t=0.79
Grace 2014 (46) (Borland 2013(86))	Self‐rated well‐being – good (mean nr participants) YP^4^ Interview data	24 months	N= 235 M=0.44	N=187 M=0.57	Diff=‐0.13, t=1.86
Grace 2014 (46) (Borland 2013(86))	Self‐rated well‐being – bad (mean nr participants) YP^4^ Interview data	24 months	N= 235 M=0.17	N=187 M=0.20	Diff=‐0.03, t=0.44
Grace 2014 (46) (Borland 2013(86))	Has support from family and communities – self‐report (mean nr participants) YP^4^ Interview data	24 months	N= 235 M=0.92	N=187 M=0.95	Diff=‐0.03, t=0.64
Grace 2014 (46) (Borland 2013(86))	Participates in community activities – self‐report (mean nr participants) YP^4^ Interview data	24 months	N= 235 M=0.25	N=187 M=0.34	Diff=‐0.09, t=1.40

### Table 4.3. High intensity case management compared to usual services for homeless adults with families – secondary outcomes

**Table:**

**Study**	**Outcome**	**Longest follow‐up**	**Intervention (N=54)**	**Comparison (N=51)**	**Result**
Nyamathi 2015 (83)	Rearrest – self report Number (%) who reported having been arrested at least once during observation period	12 months	94 (56.6%)	101 (54.3%)	X^2^ p value =0.693
Nyamathi 2015 (83)	Reincarceration– self report Number who reported having been arrested at least once during observation period	12 months	97 (58.4%)	108 (58.1%)	X^2^ p value = 0.997
Nyamathi 2015 (83)	Drug use – self report Texas Christian University Drug History form (number (%) who used marijuana, stimulants or heroin during study)	12 months	Marijuana: 82 (49.4%) Stimulants: 77 (46.4%) Heroin: 12 (7.2%)	Marijuana: 85 (45.7%) Stimulants: 95 (51.1%) Heroin: 24 (12.9%)	X^2^ p value = 0.780
Nyamathi 2015 (83)	Employment status – self report Number (%) who reported having had part‐time or full‐time employment during observation period	12 months	Full time: 24 (14.5%) Part time: 29 (17.5%) Unemployed: 113 (68.1%)	Full time: 35 (18.6%) Part time: 28 (14.9%) Unemployed: 125 (66.5%)	X^2^ p value = 0.357
Nyamathi 2015 (83)	Health condition – self report Single item 5‐point scale (fair/poor/good/very good/excellent) dichotomized to fair/poor vs very good/excellent) Number (%) who report having good/excellent or poor/fair health during observation period	12 months	Good/excellent: 131 (78.9%) Poor/fair: 35 (21.1%)	Good/excellent: 133 (70.7%) Poor/fair: 55 (29.3%)	X^2^ p value = 0.166
Toro 1997 (80)	Employment – self report Income from work on Housing, Income, and Services timeline (mean $ (SD))	18 months	485 (1,905)	920 (2,438)	Condition x time F=0.26, ns
Toro 1997 (80)	Physical health Physical Health Symptoms Checklist (mean score (SD)) Range: unclear	18 months	2.31 (2.73)	2.78 (3.03)	Condition x time F=0.00, ns
Toro 1997 (80)	Psychological symptoms – self report Symptom Checklist – 90‐ Revised (SCL‐90‐R) (mean score (SD)) Range: unclear	18 months	0.32 (0.42)	0.51 (0.53)	Condition x time F=0.02, ns
Toro 1997 (80)	Psychological symptoms Brief Psychiatric Rating scale‐90‐R (mean score (SD)) Range: 27‐72 higher score indicates greater symptom severity	18 months	24.0 (2.9)	25.4 (3.4)	Condition x time F=5.54, p<.05
Toro 1997 (80)	Stress The Modified Life Events Interview, 88 items (mean score (SD)) Range: higher score indicates higher number of stressful events	18 months	8.3 (4.8)	10.4 (5.8)	Condition x time F=04.69, p<.05
Toro 1997 (80)	Social support Social Network Interview (mean score (SD)) Range: 0‐1 (maximum 12), high score indicates more social relationships	18 months	‐0.69 (2.05)	0.33 (2.44)	Condition x time F=0.75, ns
Toro 1997 (80)	Social support – family network size and frequency of contacts Social Network Interview‐Family (mean score (SD)) Range: 0‐1 (maximum 12), high score indicates more social relationships	18 months	‐0.48 (1.34)	‐0.21 (1.55)	Condition x time F=2.83, ns
Toro 1997 (80)	Perceived social support – self report Interpersonal support evaluation list (ISEL) (mean score SD)) Range: higher score indicates more perceived social support	18 months	120.8 (18.4)	116.3 (19.3)	Condition x time F=1.32, ns
Toro 1997 (80)	Alcohol use ‐ self report Drinking Index (mean score (SD)) Range: range of average number of drinks consumed daily over last year	18 months	0.90 (1.92)	0.68 (1.44)	Condition x time F=0.04, ns
Toro 1997 (80)	Self‐efficacy – self report Self‐efficacy scale (SES) (mean score (SD)) Range: higher score indicates greater self‐efficacy	18 months	6.03 (0.80)	5.74 (1.01)	Condition x time F= 0.51, ns

### Table 4.4. High intensity case management compared to low intensity case management: secondary outcomes

The three included studies reported outcomes related to mental health (52, 53), substance abuse, criminal activity, quality of life and social support. Results are presented in table X in Appendix X.

**Table:**

**Study**	**Outcome**	**Longest follow‐up**	**Intervention**	**Comparison**	**Result**
Employment outcomes					
Morse 1997 (40)	Income – self report Monthly income from employment, panhandling or entitlements (mean USD$ (SD))	18 months	ACT‐CW: 508.23 (215.41) ACT: 523.57 (244.37)	506.21 (496.68)	No significant difference found between groups on income.
Mental health outcomes					
Morse 1997 (40)	Psychiatric symptoms – Anxiety‐Depression Brief Psychiatric Rating Scale (BPRS)‐relevant items (mean score (SD)) Range: 5‐35, higher score indicates more symptoms)	18 months	ACT‐CW: 9.85 (4.75) ACT: 11.49 (5.73)	11.39 (5.21)	No significant difference found
Morse 1997 (40)	Psychiatric symptoms – hostility‐suspicion Brief Psychiatric Rating Scale (BPRS) – relevant items (mean score (SD)) Range: 3‐21, higher score indicates more symptoms	18 months	ACT‐CW: 5.36 (2.47) ACT: 5.60 (2.67)	6.18 (3.28)	No significant difference found
Morse 1997 (40)	Psychiatric symptoms –thought disorder Brief Psychiatric Rating Scale (BPRS) – relevant items (mean score (SD)) Range: 5‐35, higher score indicates more symptoms	18 months	ACT‐CW: 8.29 (4.28) ACT: 7.42 (3.86)	10.44 (6.26)	Significant treatment group effect, F=3.91, df=2, 123, p<.023
Morse 1997 (40)	Psychiatric symptoms – withdrawal‐elevated mood Brief Psychiatric Rating Scale (BPRS) – relevant items (mean score (SD)) Range: 6‐42, higher score indicates more symptoms	18 months	ACT‐CW: 9.91 (3.37) ACT: 9.19 (2.76)	10.59 (3.77)	Marginally significant treatment group effect, p<.065
Morse 1997 (40)	Psychiatric symptoms – unusual activity level Brief Psychiatric Rating Scale (BPRS) – relevant items (mean score (SD)) Range: 5‐35, higher score indicates more symptoms	18 months	ACT‐CW: 7.58 (3.31) ACT: 7.30 (2.73)	8.97 (3.96)	Significant treatment group effect, F=3.61, df=2, 123, p<.03
Essock 2006 (53)	Psychiatric symptoms (mean, SD) Expanded Brief Psychiatric Rating scale. Range: 24 to 168, higher scores indicating more symptoms	36 months	N=99 M=43.07 (11.23)	N=99 M=43.24 (12.55)	MD=‐0.17 (SE=1.69), p=0.92*
Essock 2006 (53)	Overall functioning (mean, SD) Global Assessment Scale Range: 1 to 100, higher scores indicating better functioning	36 months	N=99 M=42.94 (9.21)	N=99 M=42.56 (9.89)	MD=0.38 (SE=1.36), p=0.78*
Drake 1998 (52)	Psychiatric symptoms Expanded Brief Psychiatric Rating Scale (BPRS)) (mean score (SD)) Range: 27‐72	36 months	N=105 40.89 (10.82)	N=98 41.11 (11.69)	MD=‐0.22 (SE=1.58), p=0.89
Morse 1997 (40)	Self‐esteem Rosenburg scale (short form) (mean score (SD)) Range: 0‐3, higher score indicates greater self‐esteem	18 months	ACT‐CW: 1.98 (0.59) ACT:1.89 (0.48)	Baseline: 1.93 (0.49) 18 mos:1.84 (0.51)	No significant difference found
Substance use outcomes					
Morse 1997 (40)	Substance abuse – self report Mean number of days abused substances in past month (SD)	18 months	ACT‐CW: 1.71 (4.92) ACT: 3.05 (6.05)	Baseline: 6.59 (9.91) 18 mos: 4.24 (7.50)	No significant difference found
Essock 2006 (53)	Alcohol abuse – self‐report (mean, SD) Number of days of alcohol use in past 6 months Range: score of 3 or higher indicates abuse)	36 months	N=99 M=33.95 (47.88)	N=99 M=30.14 (49.83)	MD=3.81 (SE=6.95), p=0.58*
Essock 2006 (53)	Alcohol abuse (mean, SD) Alcohol Use Scale Range: 1.5, higher scores indicate more severe dependence. Scores are reported only for clients who scored 3 or higher at baseline.	36 months	N=99 M=2.65 (1.05)	N=99 M=2.8 (1.34)	MD=‐0.15 (SE=0.17), p=0.38*
Essock 2006 (53)	Drug abuse‐ self‐report Number of days of drug use in the past 6 months Range: score of 3 or higher indicates abuse	36 months	N=99 M=31.77 (47.92)	N=99 M=32.06 (49.14)	MD=‐0.29 (SE=6.90), p=0.97*
Essock 2006 (53)	Drug abuse (mean, SD) Drug Use Scale Range: 1.5, higher scores indicate more severe dependence. Scores are reported only for clients who scored 3 or higher at baseline.	36 months	N=99 M=2.85 (1.35)	N=99 M=2.9 (1.34)	MD=‐0.05 (SE=0.19), p=0.79*
Essock 2006 (53)	Substance abuse Substance Abuse Treatment Scale. Range: 1 to 8, higher scores indicating more progress toward substance use remission and recovery.	36 months	N=99 M=4.45 (1.79)	N=99 M=4.35 (2.14)	MD=0.10 (SE=0.28), p=0.72*
Drake 1998 (52)	Alcohol use (mean, SD) Days used in past 6 months)	36 months	N=75 46.40 (53.60)	N=68 43.60 (57.30)	MD=2.8 (SE=9.3), p=0.76
Drake 1998 (52)	Alcohol use (mean, SD) Alcohol use Scale (AUS) Range: 5‐point scale, higher score indicates greater dependence (only those with alcohol use at baseline included)	36 months	N=83 2.64 (1.12)	N=73 2.77 (1.18)	MD=‐0.13 (SE=0.18), p=0.48
Drake 1998 (52)	Drug use (mean, SD) Days used in past 6 months)	36 months	N=45 38.20 (54.70)	N=40 51.50 (67.20)	MD=‐13.30 (SE=13.39), p=0.32
Drake 1998 (52)	Drug use (mean, SD) Drug Use Scale (DUS) Range: 5‐point scale, higher score indicates greater dependence (only those with drug use at base line included)	36 months	N=47 2.58 (1.23)	N=43 2.78 (1.16)	MD=‐0.20 (SE=0.25), p=0.43
Drake 1998 (52)	Substance abuse recovery (mean, SD) Substance Abuse Treatment Scale (SATS) Range: 8 point scale indicating recovery, higher score indicates more progression)	36 months	N=105 5.03 (1.92)	N=98 4.92 (1.89)	MD= 0.11 (SE=0.27), p=0.68
Quality of life outcomes					
Essock 2006 (53)	General Life Satisfaction Scale (mean, SD) Quality of Life Interview) Range: 1 to 7, higher scores indicating more satisfaction with life in general	36 months	N=99 M=4.75 (1.62)	N=99 M=4.75 (1.55)	MD=0.00 (SE=0.23), p=1.0*
Drake 1998 (52)	Life satisfaction Quality of life Interview (QOLI – life satisfaction) (mean score (SD)) Range: 1 to 7, higher scores indicating more satisfaction with life in general	36 months	N=105 4.56 (1.23)	N=98 4.46 (1.29)	MD=0.01 (SE=0.18), p=0.57
Social support/functioning outcomes					
Drake 1998 (52)	Social contact Quality of life Interview (QOLI – life satisfaction) (mean score (SD)) Range: 1 to 7, higher scores indicating more satisfaction with life in general:	36 months	N=105 2.72 (1.03)	N=98 2.70 (0.85)	MD=0.02 (SE=0.13), p=0.88
Drake 1998 (52)	Family contact Quality of life Interview (QOLI – life satisfaction) mean, SD Range: 1 to 7, higher scores indicating more satisfaction with life in general:	36 months	N=105 3.26 (0.94)	N=98 3.25 (0.93)	MD=0.01 (SE=0.13), p=0.94
Criminal activity outcomes					
Essock 2006 (53)	Criminal – self‐report Mean number of days spent in jail (SD)	36 months	N=99 NR	N=99 NR	N=99 NR

^*^Calculated by review authors

### Table 4.5. High intensity case management compared to other intervention: Secondary outcomes

**Table:**

**Study (ref)**	**Outcome**	**Longest follow‐up**	**Comparison**	**Intervention**	**Effect size**
Nyamathi 2015 (83)	Rearrest – self report Number (%) who reported having been arrested at least once during observation period	12 months	94 (56.6%)	104 (58.8%)	X^2^ p value =0.693
Nyamathi 2015 (83)	Reincarceration– self report Number who reported having been arrested at least once during observation period	12 months	97 (58.4%)	103 (58.2%)	X^2^ p value = 0.997
Nyamathi 2015 (83)	Drug use – self report Texas Christian University Drug History form (number (%) who used marijuana, stimulants or heroin during study)	12 months	Marijuana: 82 (49.4%) Stimulants: 77 (46.4%) Heroin: 12 (7.2%)	Marijuana: 85 (48.0%) Stimulants: 80 (45.2%) Heroin: 22 (12.4%)	X^2^ p value = 0.780
Nyamathi 2015 (83)	Employment status – self report Number (%) who reported having had part‐time or full‐time employment during observation period	12 months	Full time: 24 (14.5%) Part time: 29 (17.5%) Unemployed: 113 (68.1%)	Full time: 21 (12.0%) Part time: 24 (13.7%) Unemployed: 130 (74.3%)	X^2^ p value = 0.357
Nyamathi 2015 (83)	Good/Excellent perceived health – self report Single item 5‐point scale (dichotomized to good or bad) Number (%)	12 months	Good/excellent: 131 (78.9%)	Good/excellent: 135 (77.1%)	X^2^ p value = 0.166
Nyamathi 2015 (83)	Poor/fair perceived health– self report Single item 5‐point scale (dichotomized to good or bad) Number (%)	12 months	Poor/fair: 35 (21.1%)	Poor/fair: 40 (22.9%)	X^2^ p value = 0.166

### Table 4.6. High intensity case management (with consumer case managers) compared to high intensity case management (with non‐consumer case managers)

**Table:**

**Study**	**Outcome**	**Longest follow‐up**	**Intervention**	**Comparison**	**Result**
Solomon 1994 (76)	Arrests	24 months	Six clients reported being arrested.
Solomon 1994 (76)	Employment	24 months	Only two clients reported working for pay at both the 1‐year and 2‐year interviews. Eighty never worked for pay during the 2‐year period.
Solomon 1994 (76)	Social network size (mean (SD)) Assessment tool not described Range: (0‐11), Higher scores indicate more positive outcomes	24 months	2.46 (2.72)	2.32 (2.09)	Not statistically significant
Solomon 1994 (76)	Psychiatric symptom severity Brief Psychiatric Rating Scale (BPRS) score (mean (SD)) (24‐61), Higher scores indicate more severe symptoms Follow‐up: 12, 24 mos	24 months	27.44 (3.98)	26.15 (3.27)	Not statistically significant
Solomon 1994 (76)	Subjective quality of life Lehman's Quality of Life Index (QoLI) score (mean (SD)) Range: 2.59‐6.94, Higher scores indicate more positive outcomes	24 months	5.06 (0.58)	5.03 (0.87)	Not statistically significant
Solomon 1994 (76)	Interpersonal contact Assessment tool not described (mean (SD)) Range: 4‐30, Higher scores indicate more positive outcomes	24 months	14.35 (6.16)	16.21 (6.15)	Not statistically significant
Solomon 1994 (76)	Social functioning Range. 0‐13, Higher scores indicate more positive outcomes	24 months	3.11 (2.09)	3.89 (2.36)	Not statistically significant

### Table 4.7. Low intensity case management compared to usual services ‐ Secondary outcomes

In one study (64) participants in the intervention group reported slightly more days in employment than the control group, but this difference was not significant (no numbers reported9. There was also no difference in psychiatric and social care needs, quality of life, social behaviour, or deviant behaviour between the two groups at the 14 month follow‐up.

In the other study (26) sosin participants in the intervention group reported 2.5 days less alcohol and drug consumption between baseline and 12 month follow‐up (statistically significant). No data was reported for the control group.

**Table:**

**Study**	**Outcome**	**Longest follow‐up**	**Intervention**	**Comparison**	**Effect size**
Marshall 1995 (64)	Employment status ‐ any (mean no. days in employment) Range: NA	14 months	Mann‐Whitney *U*=726, p=0.40 Subjects in the case‐mangement group spent slightly more days in employment than expected, whereas subjects in the control group spent slightly fewer days than expected, but no significant difference between groups
Marshall 1995 (64)	Employment status – paid/training (mean no. days in employment) Range: Follow‐up: 14 mos	14 months	Mann‐Whitney *U*=733, p=0.67 No significant difference between groups
Marshall 1995 (64)	Psychiatric and social care needs (score (n)) Modified version of MRC Needs for Care Schedule Range: Not reported	14 months	N=40 1.3 (31)	N=40 1.3 (30)	MD (95% CI) = ‐0.07 (‐0.97 to 0.84), F=0.02 (Estimate of change that would represent clinically relevant difference = 1.0) Significant falls in the number of needs for psychiatric/medical care and social care in both groups (F=18.7, p<0.001) but no significant different between groups.
Marshall 1995 (64)	Quality of life – self‐report (score (n)) Lehman Quality of Life Interview Range: not reported	14 months	N=40 0.2 (31)	N=40 0.2 (27)	MD (95% CI) = 0.00 (‐0.42 to 0.42), F=0.19 (Estimate of change that would represent clinically relevant difference = 1.0)
Marshall 1995 (64)	Social behaviour (score (n)) REHAB general behaviour Range: not reported	14 months	N=40 7.5 (31)	N=40 4.9 (31)	MD (95% CI) = 4.3 (‐4.9 to 13.4), F=0.87 (Estimate of change that would represent clinically relevant difference = 15)
Marshall 1995 (64)	Deviant behaviour (score (n)) REHAB deviant behaviour Range: not reported	14 months	N=40 0.42 (31)	N=40 0.19 (30)	MD (95% CI) = 0.3 (0.15 to 0.46), F=8.42, p<0.01 (Estimate of change that would represent clinically relevant difference = 0.5)
Marshall 1995 (64)	Social behaviour – self‐report (score (n)) Social Integration Questionnaire Range: not reported	14 months	N=40 0.2 (31)	N=40 0.2 (30)	MD (95% CI) = ‐0.07 (‐0.27 to 0.13), F=1.36 (Estimate of change that would represent clinically relevant difference = 0.5)
Marshall 1995 (64)	Psychiatric symptoms (score (n)) Manchester Scale Range: not reported	14 months	N=40 0.1 (31)	N=40 ‐0.8 (30)	MD (95% CI) = 0.75 (‐1.0 to 2.54), F=0.26 (Estimate of change that would represent clinically relevant difference = 2)
Sosin 1995 (26)	Alcohol and drug use – self‐report (mean (SD) reported days of use)	12 months	Ordinary least squares regression (binary variables represent CM and CM+H) CM+H: One‐tailed t‐test = ‐1.999 (‐1.89), p<.05 CM: Two‐tailed t‐test = ‐2.461 (‐2.01), p<.05 CM decreases reported average days of alcohol and drug consumption by a modest, but statistically significant 2.5 days; CM+H decreases the variable by a statistically significant 2 days. When sample selection bias is controlled for using lambda: CM+H: One‐tailed t‐test = ‐2.316 (‐2.07), p<.05 CM: Two‐tailed t‐test = ‐2.534 (‐2.08), p<.05

### Table 4.8. Low intensity case management (with OT) compared to low intensity case management ‐ Secondary outcomes

**Table:**

**Study**	**Outcome**	**Longest follow‐up**	**Intervention**	**Comparison**	**Effect size**
Chapleau 2012 (47)	No secondary outcomes reported

### Table 4.9. Low intensity case management compared to other intervention (no case management or housing component) ‐ Secondary outcomes

**Table:**

**Study**	**Outcome**	**Longest follow‐up**	**Intervention**	**Comparison**	**Effect size**
Slesnick 2015 (74)	Drug use – self report Percent days any drug use except tobacco and alcohol of 90 days prior to last use of illicit drugs Form 90 ‐ (mean (SD))	12 months	N=60 46.30 (38.86)	CRA (N=60): 46.30 (38.86) MET (N=67) 49.21 (40.97)	No significant different between groups
Slesnick 2015 (74)	Alcohol use Percent days any alcohol use of 90 days prior to last use of alcohol Form 90 (mean (SD))	12 months	N=60 9.37 (18.58) *d* [95%CI]=‐0.292 (‐0.61, 0.04]	CRA (N=69): 6.66 (11.82) MET (N=67): 8.94 (18.41)	No significant different between groups
Slesnick 2015 (74)	Alcohol use – urine analysis Average standard ethanol content (mean (SD))	12 months	N=60 1.78 (2.82)	CRA (N=69): 1.89 (3.91) MET(N=67): 1.65 (3.24)	No significant different between groups
Slesnick 2015 (74)	Depressive symptoms Beck Depression Inventory‐II (mean score (SD)) Range: 0‐63. higher scores indicate higher levels of depressive symptoms	12 months	N=56 8.42 (11.11)	CRA (N=62): 12.74 (12.63) MET (N=62): 7.96 (10.46)	No significant different between groups
Slesnick 2015 (74)	Internalizing problems Youth Self‐Report of the Child Behavior Checklist (YSR). Range.3 point Likert scale. higher scores indicate more problem behaviors	12 months	N=64 15.39 (10.78)	CRA (N=70): 17.19 (12.37) MET (N=68): 17.92 (11.79)	Significant difference between CM and MET in favour of CM
Slesnick 2015 (74)	Externalizing problems Youth Self‐Report of the Child Behavior Checklist (YSR). Range.3 point Likert scale. higher scores indicate more problem behaviors	12 months	N=64 13.37 (9.76)	CRA (N=70): 13.76 (9.79) MET (N=68): 16.99 (10.18)	Significant difference between CM and MET in favour of CM
Slesnick 2015 (74)	Task‐oriented coping Coping Inventory for Stressful Situations (CISS) (mean (SD)) Range: not specified	12 months	N=58 54.69 (15.51)	CRA (N=70): 53.04 (16.04) MET (N=64): 55.86 (12.22)	No significant difference between groups
Slesnick 2015 (74)	Emotion‐oriented coping Coping Inventory for Stressful Situations (CISS) (mean (SD)) Range: not specified	12 months	N=56 42.27 (13.33)	CRA (N=64): 44.80(14.61) MET (N=65): 45.42 (13.21)	No significant difference between groups
Slesnick 2015 (74)	Avoidance‐oriented coping Coping Inventory for Stressful Situations (CISS) (mean (SD)) Range: not specified	12 months	N=60 50.35 (13.48)	CRA (N=66): 50.23 (14.07) MET (N=64): 52.08 (10.83)	No significant difference between groups
Slesnick 2015 (74)	Victimization experience – self report Number participants assaulted during last 3 mos (yes ‐ mean (SD). no – mean (SD))	12 months	Yes = 16 (17.58%) No = 48 (52.75%)	CRA Yes = 15 (16.13%) No = 54 (58.06%) MET Yes = 18 (20.93%) No = 50 (58.14%)	No significant difference between groups
Sorensen 2003 (77)	Substance use – drug (self‐report) Addiction Severity Index – drug composite score (mean (SD)) Range: : 0‐1, lower score indicates less severity	18 months	N=92 0.1 (0.1)	N=98 0.1 (0.1)	No significant difference between groups
Sorensen 2003 (77)	Substance use – alcohol (self‐report) Addiction Severity Index – alcohol composite score (mean (SD)) Range: : 0‐1, lower score indicates less severity	18 months	N=92 0.1 (0.16)	N=98 0.1 (0.19)	No significant difference between groups
Sorensen 2003 (77)	Physical health – overall health status Health Status Questionnaire (HSQ) (mean (SD)) Range: 0‐100	18 months	N=92 31.7 (23.13)	N=98 42.2 (28.29)	No significant difference between groups
Sorensen 2003 (77)	Physical health – ASI medical Addiction Severity Index – medical composite score (mean (SD)) Range: 0‐1, lower score indicates less severity	18 months	N=92 0.5 (0.32)	N=98 0.4 (0.32)	No significant difference between groups
Sorensen 2003 (77)	Psychological status – BDI Beck Depression Inventory score (mean (SD)) Range: 0‐63. higher scores indicate higher levels of depressive symptoms	18 months	N=92 19.8 (10.93)	N=98 18.1 (11.32)	No significant difference between groups
Sorensen 2003 (77)	Psychological status – ASI psychiatric Addiction Severity Index – psychiatric composite score (mean (SD)) Range: 0‐1, lower score indicates less severity	18 months	N=92 0.3 (0.26)	N=98 0.2 (0.25)	No significant difference between groups
Sorensen 2003 (77)	Quality of living situation – social support network Interpersonal Support Evaluation List (ISEL (mean (SD)) Range: Not specified which version or if scoring 0‐3 or 1‐4	18 months	N=92 36.1 (11.53)	N=98 38.9 (11.31)	No significant difference between groups
Sorensen 2003 (77)	Quality of living situation– ASI psychiatric Addiction Severity Index – psychiatric composite score (mean (SD)) Range: 0‐1, lower score indicates less severity	18 months	N=92 0.9 (0.19)	N=98 0.9 (0.13)	No significant difference between groups
Sorensen 2003 (77)	Quality of living situation– ASI legal Addiction Severity Index – legal composite score (mean (SD)) Range: 0‐1, lower score indicates less severity	18 months	N=92 0.1 (0.18)	N=98 0.1 (0.16)	No significant difference between groups
Sorensen 2003 (77)	Quality of living situation– ASI family Addiction Severity Index – family composite score (mean (SD)) Range: 0‐1, lower score indicates less severity	18 months	N=92 18 mos: NR	N=98 0.0 (0.18)	No significant difference between groups

### Table 4.10. Critical time intervention compared to usual services ‐ Secondary outcomes

One study included outcomes related to social support (Herman 2011). Participants in the intervention group reported significantly better perceived quality of family relationships than the control group at the 18 month interview (b=0.61, SE=0.30, p=0.04 using a mixed effects regression model). Two studies included outcomes related to mental health (72, 79). There were no difference between groups with respect to Global Severity Index scores, or according to the Positive and Negative Syndrome Scale. Table XX in Appendix XX provides a more detailed description of the results for secondary outcomes.

**Table:**

**Study**	**Outcome**	**Longest follow‐up**	**Intervention**	**Comparison**	**Effect size**
Susser 1997 (79) (Herman 2000 (87))	Psychiatric symptom severity – negative symptoms Positive and Negative Syndrome Scale (PANSS) (mean score (SD)) Range: 7‐49, higher score indicates greater severity	6 months	N=38 (complete data at baseline and 6 mos) Baseline: 18.7 (6.1) 6 mos: 16.1 (5.7)	N=38 (complete data at baseline and 6 mos) Baseline: 17.7 (5.6) 6 mos: 18.7 (7.0)	F(1, 73)=5.7, p=.02
Susser 1997 (79) (Herman 2000 (87))	Psychiatric symptom severity – positive symptoms Positive and Negative Syndrome Scale (PANSS) (mean (SD)) Range: 7‐49, higher score indicates greater severity	6 months	N=38 (complete data at baseline and 6 mos) Baseline: 17.1 (7.7) 6 mos: 14.9 (6.0)	N=38 (complete data at baseline and 6 mos) Baseline: 17.1 (6.1) 6 mos: 14.4 (4.8)	F(1, 73)=0.2, p=.64
Susser 1997 (79) (Herman 2000 (87))	Psychiatric symptom severity – general psychopathology Positive and Negative Syndrome Scale (PANSS) (mean (SD)) Range: 7‐49, higher score indicates greater severity	6 months	N=38 (complete data at baseline and 6 mos) Baseline: 39.3 (11.7) 6 mos: 32.8 (8.8)	N=38 (complete data at baseline and 6 mos) Baseline: 37.1 (11.6) 6 mos: 34.1 (10.5)	F(1, 73)=0.5, p=.48

### Table 4.11. Abstinence‐contingent housing with case management compared to usual services ‐ secondary outcomes

**Table:**

**Study**	**Outcome**	**Longest follow‐up**	**Intervention**	**Comparison**	**Effect size**
Sosin 1995 (26)		Alcohol and drug use – self‐report (mean (SD) reported days of use)	12 months	CM+H: One‐tailed t‐test = ‐1.999 (‐1.89), p<.05 CM+H decreases the variable by a statistically significant 2 days.

### Table 4.12. Abstinence‐contingent housing with case management compared to case management only ‐ secondary outcomes

**Table:**

**Study**	**Outcome**	**Longest follow‐up**	**Intervention**	**Comparison**	**Effect size**
Sosin 1995 (26)	Alcohol and drug use – self‐report (mean (SD) reported days of use)	12 months	Difference between abstinence‐contingent housing with case management and case management only were not reported		

### Table 4.13. Abstinence‐contingent housing with day treatment compared to usual services – secondary outcomes

**Table:**

**Study**	**Outcome**	**Longest follow‐up**	**Intervention**	**Comparison**	**Effect size**
Milby 1996 (67)	Alcohol use (mean) Addiction Severity Index ‐ number days in past 30 days alcohol used	12 months	Intervention clients reported a greater reduction in alcohol use over the last 30 days than control clients (p=.026). EC had 8 days fewer of reported alcohol use in the past 30 days from baseline to 12‐months with no difference in the UC group.		
Milby 1996 (67)	Drug use – urine tests Proportion/median positive cocaine toxicologies	12 months	Percent cocaine‐positive urine toxicologies revealed a significant difference between the two treatment groups across all time points (p=.0003). Intervention group had 4% fewer positive cocaine toxicologies at 12 months than control group.		
Milby 1996 (67)	Employment – self report (mean) Addiction Severity Index ‐ number days employed in past 30 days	12 months	The longitudinal difference between groups was not statistically significant across all time points (p=.504).		

### Table 4.14. Abstinence‐contingent housing with day treatment compared to day treatment only – secondary outcomes

**Table:**

**Study**	**Outcome**	**Longest follow‐up**	**Intervention**	**Comparison**	**Effect size**
Kertesz 2007 (58)	Mean number of days employed of past 60 days between baseline and 12 months Retrospective Interview for Housing, Employment, and Treatment History ‐ Self‐report	12 months	MD=12.3 (SD=31.9)	MD=9.09 (SD=32.6)	Not reported
Kertesz 2007 (58)	Number of days in employment	12 months	N=39 M=12.3 (SD=5.1)	N=34 M=9.1 (SD=5.6)	MD=3.20 (95%CI=0.73, 5.67)
Kertesz 2007 (58)	Number of days in paid employment	12 months	N=39 M=12.3 (SD=5.1)	N=34 M=14.8 (SD=4.5)	MD= ‐2.50 (95%CI=‐4.59, ‐0.41))
Milby 2003 (66)	Employment Increase in mean days employed in last 60 days (mean, (SE))	12 months	10.2 (SE=3.8) t(278)= 5.11, p=.003	9.7 (SE=2.9) t(278)= 2.79, p=.006	F(2, 278)= 0.48, p=.62
Milby 2003 (66)	Drug abstinence prevalence Proportion of participants who did not use drugs in last 30 days (point estimate) Addiction Severity Index‐Drugs	12 months	36%	29%	F(3, 417)= 2.3, p=.08
Milby 2003 (66)	Number of days in employment	12 months	N=72 M=10.2 (SD=3.8)	N=69 M=9.7 (SD=2.9)	MD=0.50 (95%CI= ‐0.61, 1.61)

### Table 4.14. Abstinence‐contingent housing with day treatment compared to non‐abstinence‐contingent housing with day treatment – secondary outcomes

**Table:**

**Study**	**Outcome**	**Longest follow‐up**	**Intervention**	**Comparison**	**Effect size**
Kertesz 2007(58)	Mean number of days employed of past 60 days between baseline and 12 months Retrospective Interview for Housing, Employment, and Treatment History ‐ Self‐report	12 months	MD=12.3 (SD=31.9)	MD= 14.8 (SD=29.7)	Not reported

### Table 4.15. Abstinence‐contingent housing with day treatment compared to abstinence‐contingent housing with community reinforcement approach – secondary outcomes

**Table:**

**Study**	**Outcome**	**Longest follow‐up**	**Intervention**	**Comparison**	**Effect size**
Milby 2010 (68)	Employment – self report (% employed more than 40 of past 60 days) Retrospective Housing, Employment and Substance Abuse Treatment Interview	18 months	28.0	21.6	No between group differences (P>0.06 at all time points)
Milby 2010 (Milby 2008)	Abstinence (mean consecutive weeks abstinent) Urine test Range: 0‐52	12 months	19.18 (SD=16.0)	13.9, SD=12.6	Unadjusted analysis: diff=5.28 weeks, p=.009 Adjusted analysis (for age difference at baseline): 5.2 weeks, p=.013 (not clinically important)
Smith 1998 (75)	Employment Proportion of people with jobs (mean) (n=74)	12 months	NR	NR	No between‐group differences in employment status at any time point.
Smith 1998 (75)	Number of days employed in past 30 days (Addiction Severity Index – Employment)	12 months	NR	NR	NR
Smith 1998 (75)	Mean number of drinking days per week	12 months	NR	NR	F(1, 94)= 9.03, p=.0034
Smith 1998 (75)	Peak alcohol blood content		NR	NR	F(1, 94)= 6.19, p=.0146
Smith 1998 (75)	Mean total number of drinks per week		NR	NR	F(1, 94)= 5.75, p=.0184

### Table 4.16. Housing First compared to usual services – secondary outcomes

One study (42)evaluated the effect of Housing First compared to usual services on community functioning and quality of life. Although community functioning and quality of life improved for all participants, there was a greater increase for participants in the Housing First groups on both measures (Aubry 2015). Further detail regarding secondary outcomes for subgroups (high needs and moderate needs) are available in Table X in Appendix XX.

Another study (43)also reported quality of life outcomes using the Aids Clinical Trial Group SF21 instrument. Results show that participants in the Housing First group reported slightly better physical functioning (MD=53.6 (95%CI 49.2 to 60.0)) than the control group (MD=52.2 (95%CI46.9 to 57.4)) but that this was not significant (p=0.68). Participants in the Housing First group also reported slightly better mental health (M=57.0 (95%CI 52.8 to 61.3)) than the control group (M=54 (95%CI 49.1 to 58.9) but that this was not significant (p=0.35). There were no significant difference between the groups on criminal arrests or number of days in jail, but there were significant differences on number of convictions and days in prison in favour of the treatment group (p<0.10).

**Table:**

**Study**	**Outcome**	**Longest follow‐up**	**Intervention**	**Comparison**	**Effect size**
Aubry 2015 (42) (final report (88))	Quality of Life Index (QOLI 20) – self report	24 months	improvements in quality of life were somewhat greater in the Housing First group than the comparison group
Aubry 2015 (42) (final report (88))	Community functioning, Multnomah Community Ability Scale (MCAS)	24 months	improvements in community functioning were somewhat greater in the Housing First group than the comparison group
Basu 2011(43)	Mortality (n, %) Follow‐up: 18 mos	18 months	25, 12%	23, 11%	n.s.
Basu 2011(43) (Sadowski 2009 (89))	Quality of life – physical functioning (unadjusted) (mean, SD) Aids Clinical Trial Group SF21 instrument (ACTG) – physical functioning subscale Range: 0 – 100, higher scores indicate better health	18 months	M=53.6 (95%CI 49.2, 60.0)	M=52.2 (95%CI 46.9, 57.4)	P=.68 (adjusted scores still not significant)
Basu 2011(43) (Sadowski 2009 (89))	Quality of life – mental health (unadjusted) (mean, SD) Aids Clinical Trial Group SF21 instrument (ACTG) – physical functioning subscale Range: 0 – 100, higher scores indicate better health	18 months	M=57.0 (95%CI 52.8, 61.3)	M=54.0 (95%CI 49.1, 58.9)	P=.35 (adjusted scores still not significant)
Basu 2011(43)	Number of arrests (no. of times sent to jail) (unadjusted) (mean, SD) Public access websites for local jails/state prisons	18 months	0.21 (0.4)	0.26 (0.5)	MD=‐0.05 (SE=0.04), ns
Basu 2011(43)	Number of days in jail ‐ unadjusted (mean, SD) Public access websites for local jails/state prisons	18 months	17.9 (50)	13.9 (40)	MD=4.06 (SE=4.5), ns
Basu 2011(43)	Number of convictions ‐ unadjusted (no. times sent from jail to prison) (mean, SD) Public access websites for local jails/state prisons	18 months	0.03 (0.2)	0.07 (0.2)	MD= ‐0.03 (SE=0.01), p<.10
Basu 2011(43)	Number of days in prison – unadjusted (mean, SD) Public access websites for local jails/state prisons	18 months	6.0 (32)	13.8 (50)	MD= ‐7.73 SE=4.2), p<.10
Basu 2011(43)	Substance abuse (treatment visits) – unadjusted (mean, SD)	18 months	20.2 (58)	7.9 (33)	MD=12.24 (SE=4.7), p<.05
Basu 2011(43)	Substance abuse (days in residential substance abuse treatment) – unadjusted (mean, SD)	18 months	3.5 (11)	11.1 (36)	MD= ‐7.51 (SE=2.6), p<.05

### Table 4.17. Housing First compared to abstinence‐contingent housing – secondary outcomes

**Table:**

**Study**	**Outcome**	**Longest follow‐up**	**Intervention**	**Comparison**	**Effect size**
Tsemberis 2004 (24)	Alcohol use – self report Drug and Alcohol Follow‐Back Calendar (mean nr. drinks consumed each day during 6 mos period,) (mean)	24 months	Repeated‐measures analyses showed no significant differences in either alcohol use between the 2 groups by time condition (F4,136=1.1, P=.35)
Tsemberis 2004 (24)	Drug use – self report Drug and Alcohol Follow‐Back Calendar (mean no. days drugs were used during 6 mos period,	24 months	Repeated‐measures analyses showed no significant differences in drug use between the 2 groups by time condition F4,136=.98, P=.42)
Tsemberis 2004 (24) (Greenwood 2005 (90))	Psychiatric symptoms Colorado Symptom Index (mean score (SD)) Range: 15‐70, lower score indicates more symptoms	24 months	Repeated‐measures analyses showed no significant differences psychiatric symptoms between the 2 groups by time condition (F4,137=.348, P=.85). Program assignment did not predict psychiatric symptoms (t<1) (36 months) (Greenwood 2005).

### Table 4.18. Non‐abstinence‐contingent housing with high intensity case management compared to usual services – secondary outcomes

**Table:**

**Study**	**Outcome**	**Longest follow‐up**	**Intervention N=91**	**Comparison** **N=77**	**Effect size**
Shern 2000(73)	Quality of life Lehman's Quality of Life Scale – overall ((mean (SD) change in score)	24 months	1.19 (1.99)	‐0.02 (1.65)	t=‐4.21, p=.001
Shern 2000(73)	Psychological status Colorado Symptom Index (mean (SD) change in score)	24 months	‐0.28 (0.69)	0.04 (0.72)	T=2.74, p=.007 The experimental subjects reported significantly greater reductions in anxiety, depression, and though disturbances than did control group participants (t=2.41, p<.001).

### Table 4.19. Non‐abstinence‐contingent group living arrangements with high intensity case management compared to non‐abstinence‐contingent independent apartments with high intensity case management ‐ secondary outcomes

**Table:**

**Study**	**Outcome**	**Longest follow‐up**	**Intervention**	**Comparison**	**Effect size**
Goldfinger 1999 (55) (Schutt 1997 (91))	Satisfaction with residence overall ‐ self report (mean) Range: 1‐4 scale, higher scores indicate more satisfaction	18 months	2.2	2.5	t‐test of difference p<0.05
Goldfinger 1999 (55) (Schutt 1997 (91))	Satisfaction with life – self report (mean) Lehman Quality of Life Interview Range: 1‐7, higher score indicates greater satisfaction.	18 months	4.8	5.0	

### Table 4.20. Non‐abstinence‐contingent housing with high intensity case management compared to abstinence‐contingent housing with high intensity case management – secondary outcomes

**Table:**

**Study**	**Outcome**	**Longest follow‐up**	**Intervention**	**Comparison**	**Effect size**
McHugo 2004(65)	Psychiatric symptoms – self report (mean (SD)) Colorado symptom index (CSI) Range: 0‐60, higher score indicates greater distress	18 months	18.7 (11.0)	14.6 (11.5)	F=1.31, d=‐0.38
McHugo 2004(65)	General life satisfaction Quality of Life scale (QOLI) – overall life satisfaction subscale (mean (SD)) Range: 1‐7, higher score indicates greater satisfaction	18 months	4.9 (1.3)	5.4 (1.3)	F=0.23, d=0.41
McHugo 2004(65)	Days of alcohol use in past 6 mos – self report (mean (SD)) Time‐line Follow‐back calendar	18 months	24.5 (40.4)	29.7 (52.2)	F=0.52, d=0.03
McHugo 2004(65)	Days of illicit drug use in past 6 mos – self report (mean (SD)) Time‐line Follow‐back calendar	18 months	31.7 (60.9)	22.3 (58.0)	F=0.95, d=‐0.37

### Table 4.21. Non‐abstinence‐contingent housing with day treatment vs day treatment – secondary outcomes

**Table:**

**Study**	**Outcome**	**Longest follow‐up**	**Intervention**	**Comparison**	**Effect size**
Kertesz 2007 (58)	Change in number of days employed between baseline and 12 mnths (MD (SD))	12 months	MD=14.8 (SD=29.7)	MD=9.1 (SD=32.6	Not reported

### Table 4.22. Housing vouchers with case management compared to usual services – secondary outcomes

**Table:**

**Study**	**Outcome**	**Longest follow‐up**	**Intervention**	**Comparison**	**Effect size**
Wolitski 2010 (81)	Depression Center for Epidemiologic Studies Depression Scale CES‐D) (mean) Range: Items were scored on a scale of 1—rarely (\1 day) to 4—most days (5–7 days), higher score indicate greater depressed mood.	18 months	10.7	10.8	F=2.73, p=0.0429
Wolitski 2010 (81)	Perceived stress – self report 10–item Perceived Stress Scale (mean) Range: 1—never to 5—very often scale, higher total scores indicated more perceived stress	18 months	26.5	27.1	F=2.94, p=0.0334
Wolitski 2010 (81)	Mental health Medical Outcomes Study Short Form‐36 v.2 (SF‐36) ‐ general mental health, social functioning and role/emotional functioning subscales (mean) Range: Higher scores indicated better mental health/ functioning.	18 months	44.0	43.2	F=1.26, p=0.2878
Wolitski 2010 (81)	Physical health Medical Outcomes Study Short Form‐36 v.2 (SF‐36) Higher scores indicated better self‐perceived physical health.	18 months	43.9	44.6	F=4.33, p=0.0055
Wolitski 2010 (81)	CD4 below 200 (% participants) Blood specimens	18 months	20.7	: 22.8	F=0.11, p=0.9564
Wolitski 2010 (81)	Detectable HIV‐1 viral load (% participants) Blood specimens	18 months	57.0	63.4	F=0.41, p=0.7479
Rosenheck 2003 (71)	Alcohol use – self report Number of days drank to intoxication (mean)	36 months	1.46	1.71	t(1 vs 3)=0.73, p=0.46
Rosenheck 2003 (71)	Alcohol use Addiction severity index – alcohol index score (ASI) (mean)	36 months	N=182 0.12	N=188 0.121	HUD‐VASH vs SC t=0.34, p=.73
Rosenheck 2003 (71)	Drug use Addiction severity index – drug index score (ASI) (mean)	36 months	N=182 0.061	N=188 0.063	HUD‐VASH vs SC t= 0.21, p= .83
Rosenheck 2003 (71)	Mental health Addiction Severity Index ‐ Psychiatric index score (mean)	36 months	N=182 0.25	N=188 0.24	HUD‐VASH vs SC t=0.34, p=.73
Rosenheck 2003 (71)	Health Addiction Severity Index ‐ Medical index score (mean)	36 months	N=182 0.26	N=188 0.27	HUD‐VASH vs SC t= 0.39, p=.69
Rosenheck 2003 (71)	Employment Addiction Severity Index ‐ Employment index score (mean)	36 months	N=182 0.191	N=188 0.187	HUD‐VASH vs SC t= 0.20, p= .84
Rosenheck 2003 (71)	Employment – self report Number of days worked in past 30 days (mean)	36 months	6.96	6.71	t(1 vs 3)=
Rosenheck 2003 (71)	Legal Addiction Severity Index ‐ Legal index score (mean)	36 months	0.061	0.087	t(1 vs 3)=1.92, p=0.06
Rosenheck 2003 (71)	Total income Amount of income (mean $)	36 months	656	717	t(1 vs 3)=1.56, p=0.12
Rosenheck 2003 (71)	Social network size Number of people they feel close to (mean)	36 months	11.6	10.1	t(1 vs 3)=2.02, p=0.04
Rosenheck 2003 (71)	Social contacts Number of social contacts (mean) Follow‐up: 36 months	36 months	39.1	36.5	t(1 vs 3)=0.91, p=0.36
Rosenheck 2003 (71)	Social support Number of people who would support (mean)	36 months	7.85	7.11	t(1 vs 3)=1.83, p=0.07
Rosenheck 2003 (71)	Quality of life Subjective Quality of Life Interview – overall (mean) Range: 9‐63, higher score indicates greater quality of life)	36 months	N=182 4.31	N=188 4.18	HUD‐VASH vs SC t= 1.09, p= .28
Rosenheck 2003 (71)	Satisfaction with family relations Subjective Quality of Life Interview – family score (mean) Range: 1‐7, higher score indicates greater quality of life	36 months	4.49	4.25	t(1 vs 3)=2.02, p=0.04
Rosenheck 2003 (71)	Satisfaction with disposable income Subjective Quality of Life Interview – finances score (mean) Range: 1‐7, higher score indicates greater quality of life	36 months	3.26	3.12	t(1 vs 3)=1.31, p=0.19
Rosenheck 2003 (71)	Satisfaction with health Subjective Quality of Life Interview – health score (mean) Range: 1‐7, higher score indicates greater quality of life	36 months	4.50	4.36	t(1 vs 3)= 1.54,p=0.12
Rosenheck 2003 (71)	Satisfaction with social relations Subjective Quality of Life Interview – social score (mean) Range: 1‐7, higher score indicates greater quality of life	36 months	4.31	4.20	t(1 vs 3)=1.25, p=0.21
Rosenheck 2003 (71)	Arrests – major crimes Number of arrests (mean)	36 months	N=182 0.23	N=188 0.23	HUD‐VASH vs SC t= 0.10, p= .92
Rosenheck 2003 (71)	Arrests – minor crimes Number of arrests (mean)	36 months	N=182 0.22	N=188 0.22	HUD‐VASH vs SC t= 0.22, p= .82

### Table 4.23. Housing vouchers with case management compared to high intensity case management

**Table:**

**Study**	**Outcome**	**Longest follow‐up**	**Intervention**	**Comparison**	**Effect size**
Rosenheck 2003 (71)	Alcohol use – self report Number of days drank to intoxication (mean)	36 months	1.46	1.95	t(1 vs 2)=1.17, p=0.24
Rosenheck 2003 (71)	Alcohol use Addiction severity index – alcohol index score (ASI) (mean)	36 months	N=182 0.12	N=90 0.151	HUD‐VASH vs ICM t= 1.90, p=.06
Rosenheck 2003 (71)	Drug use Addiction severity index – drug index score (ASI) (mean)	36 months	N=182 0.061	N=90 0.065	HUD‐VASH vs ICM t= 0.44, p=.66
Rosenheck 2003 (71)	Mental health Addiction Severity Index ‐ Psychiatric index score (mean)	36 months	N=182 0.25	N=90 0.26	HUD‐VASH vs ICM t= 0.69, p= .49
Rosenheck 2003 (71)	Health Addiction Severity Index ‐ Medical index score (mean)	36 months	N=182 0.26	N=90 0.28	HUD‐VASH vs ICM t= 0.47, p= .63
Rosenheck 2003 (71)	Employment Addiction Severity Index ‐ Employment index score (mean)	36 months	N=182 0.191	N=90 0.187	HUD‐VASH vs ICM t= 0.17, p= .86
Rosenheck 2003 (71)	Employment – self report Number of days worked in past 30 days (mean)	36 months	6.96	6.82	t(1 vs 2)= t(1 vs 3)= t(2 vs 3)=
Rosenheck 2003 (71)	Legal Addiction Severity Index ‐ Legal index score (mean)	36 months	0.061	0.063	t(1 vs 2)=0.14, p=0.89
Rosenheck 2003 (71)	Total income Amount of income (mean $)	36 months	656	684	t(1 vs 2)=0.59, p=0.55
Rosenheck 2003 (71)	Social network size Number of people they feel close to (mean)	36 months	11.6	9.3	t(1 vs 2)=2.52, p=0.01
Rosenheck 2003 (71)	Social contacts Number of social contacts (mean) Follow‐up: 36 months	36 months	39.1	30.4	t(1 vs 2)=2.50, p=0.01
Rosenheck 2003 (71)	Social support Number of people who would support (mean)	36 months	7.85	6.54	t(1 vs 2)=2.65, p=0.008
Rosenheck 2003 (71)	Quality of life Subjective Quality of Life Interview – overall (mean) Range: 9‐63, higher score indicates greater quality of life)	36 months	N=182 4.31	N=90 3.92	HUD‐VASH vs ICM t= 2.64, p< .009
Rosenheck 2003 (71)	Satisfaction with family relations Subjective Quality of Life Interview – family score (mean) Range: 1‐7, higher score indicates greater quality of life	36 months	4.49	4.16	t(1 vs 2)=2.28, p=0.02
Rosenheck 2003 (71)	Satisfaction with disposable income Subjective Quality of Life Interview – finances score (mean) Range: 1‐7, higher score indicates greater quality of life	36 months	3.26	2.93	t(1 vs 2)=2.50, p=0.01
Rosenheck 2003 (71)	Satisfaction with health Subjective Quality of Life Interview – health score (mean) Range: 1‐7, higher score indicates greater quality of life	36 months	4.50	4.18	t(1 vs 2)=2.87, p=0.004
Rosenheck 2003 (71)	Satisfaction with social relations Subjective Quality of Life Interview – social score (mean) Range: 1‐7, higher score indicates greater quality of life	36 months	4.31	4.04	t(1 vs 2)=2.42, p=0.02
Rosenheck 2003 (71)	Arrests – major crimes Number of arrests (mean)	36 months	N=182 0.23	N=90 0.20	HUD‐VASH vs ICM t= 0.79, p= .43
Rosenheck 2003 (71)	Arrests – minor crimes Number of arrests (mean)	36 months	N=182 0.22	N=90 0.21	HUD‐VASH vs ICM t= 0.46, p= .64

### Table 4.24. Residential treatment compared to usual services: Secondary outcomes

**Table:**

**Study**	**Outcome**	**Longest follow‐up**	**Comparison**	**Intervention**	**Effect size**
Conrad 1998(49)	Alcohol dependency (mean, SD) Addiction Severity Index – alcohol subscale Range: 0‐9, lower score indicates less severity	24 months	NR	NR	Random effects regression estimate = 0.037 (SE=0.017), Z=2.151, p=.032
Conrad 1998(49)	Employment (mean, SD) Addiction Severity Index – employment subscale Range: 0‐9, lower score indicates less severity	24 months	NR	NR	Random effects regression estimate = 0.003 (SE=0.015), Z=0.1888, p=.851
Conrad 1998(49)	Medical (mean, SD) Addiction Severity Index – medical subscale Range: 0‐9, lower score indicates less severity	24 months	NR	NR	Random effects regression estimate = 0.005 (SE=0.016), Z=0.289, p=.773
Conrad 1998(49)	Drug dependency (mean, SD) Addiction Severity Index – drug subscale Range: 0‐9, lower score indicates less severity	24 months	NR	NR	Random effects regression estimate = 0.024 (SE=0.017), Z=1.377, p=.169
Conrad 1998(49)	Legal (mean, SD) Addiction Severity Index – legal subscale Range: 0‐9, lower score indicates less severity	24 months	NR	NR	Random effects regression estimate = ‐0.006 (SE=0.024), Z=‐0.229, p=.819
Conrad 1998(49)	Psychiatric (mean, SD) Addiction Severity Index – psychiatric subscale Range: 0‐9, lower score indicates less severity	24 months	NR	NR	Random effects regression estimate = ‐0.005 (SE=0.016), Z=‐0.295, p=.768
Lipton 1998(63)	Illness severity (mean) Structured Clinical Interview (SCI) Range: higher score indicates greater symptom severity	12 months	1.07	1.49	Ns.
Lipton 1998(63)	Social support Frequency of contact with family	12 months	NR	NR	NR
Lipton 1998(63)	Criminal Frequency of contact with police	12 months	NR	NR	NR

## Appendix 5. List of excluded studies

### Table 5.1. List of excluded studies

**Table:**

**Study** **First author** **(reference no.)**	**Cause for exclusion of study**
Archie, 2006	No evaluation, commentary
Baer, 2005	Irrelevant outcome
Baier, 1996	Irrelevant design
Barrenger 2014	qualitative study
Beach 2013	No control group
Bell, 1994	Irrelevant outcome
Benston 2015	Systematic review
Birnie, 2010	Study design unclear ‐ no response from author
Blankertz, 1994	Irrelevant outcome
Bloom, 2002	Same population as Susser 1997. No additional information.
Bomalaski, 1999	Irrelevant design
Borland, 2013	Lacking information, no answer from author
Bradford 2005	<12 months follow up
Braine, 2004	Irrelevant outcome
Braine, 2005	Irrelevant outcome
Brunette, 2001	Irrelevant outcome
Buchanan, 2006	Irrelevant outcome
Buchanan, 2009	Irrelevant outcome
Bucher, 2008	Irrelevant outcome
Buchholz 2010	Homelessness as an intervention
Burger, 2000	Irrelevant outcome
Burnam, 1995	Irrelevant outcome (<12 months)
Burns, 1995	Review
Calsyn, 1998	Irrelevant outcome
Calsyn, 2000	Quasi‐experimental design.
Calsyn, 2003	Review
Calsyn, 2004	Irrelevant outcome, same population as Morse 2006
Calsyn, 2005	Irrelevant outcome, same population as Morse 2007
Calsyn, 2006	Irrelevant outcome, same population as Morse 2008
Cameron, 2009	Irrelevant design
Caplan, 2006	Irrelevant outcome
Carr, 1998	Irrelevant outcome
Cauce, 1994	Irrelevant outcome
Chandler, 1996	Irrelevant outcome
Chandler, 1996a	No outcome statistics
Chinman, 2000	Irrelevant design; irrelevant comparison
Chinman, 2000a	Quasi‐experimental design.
Chu, 2002	Irrelevant outcome
Ciaranello, 2006	Irrelevant outcome
Clark, 1994	Irrelevant outcome
Coady, 2007	Irrelevant outcome
Compton, 2003	Irrelevant comparison: CM+ outpatient commitment vs CM
Conrad, 1998	Irrelevant outcome
Cox, 1993	Irrelevant outcome
Cruwys 2014	no control group, no housing outcomes
Cunningham, 2007	Irrelevant outcome
Dalinger 2007	not matched at baseline
Dasinger, 2007	Quasi‐experimental design.
Davidson, 2004	Irrelevant outcome
Davis, 2006	Irrelevant outcome; irrelevant comparison
De Leon, 2000	Irrelevant outcome
Deering, 2009	Irrelevant outcome
Dixon, 1997	Irrelevant outcome
Dixon, 2009	Irrelevant outcome
Drake, 1993	Irrelevant outcome
Duwe 2012	no housing outcome
Egbewale Bolaji 2012	Systematic review
Erdem 2015	< 12 months follow‐up
Erickson, 1995	Irrelevant outcome (<12 months)
Essock, 2006	Irrelevant outcome
Fekete, 1998	Irrelevant population: not homeless, not clearly stated as “at‐risk”
Felton, 1995	Irreelvant comparison: ICM+peer vs ICM+professionals vs ICM only
Ferguson 2012	no housing outcome
Fichter, 2001	Irrelevant outcome
Fletcher 2013	no housing outcome
Forchuc, 2008a	Irrelevant outcome (no data)
Forchuk, 2008	Irrelevant outcome, <12 months follow up
Foster, 2007	irrelevant outcome
Fowler 2011	quasi experimental study design
Fowler 2014	<12 months follow up
Fowler 2014	<12 months follow up
Freddolino, 1992	Irrelevant outcome (no data)
French 2010	no housing outcome
French, 1999	Irrelevant outcome
French, 1999a	Irrelevant outcome
French, 2002	Irrelevant outcome
Friedmann 2013	<12 months follow up
Gabrielian 2013	no housing outcome
Geller 2014	not a study
Gewirtz 2015	no housing outcomes
Giesbrecht 2015	full text not available
Gilmer 2010	Quasi‐experimental design
Gilmer, 2009	Irrelevant outcome
Gozdzik 2015	no housing outcomes
Grace 2014	No matching at baseline. Adjusted analysis only.
Graham‐Jones, 2004	Irrelevant outcome (<12 months)
Grigg 2005	Irrelevant design
Grigg, 2008	Irrelevant design
Gulcur, 2007	Irrelevant outcome
Guo 2014	Intervention aimed at keeping adolescents living with family
Guo 2015	no housing outcomes
Guo 2016	no housing outcomes
Hanratty 2011	quasi experimental study design
Harpaz‐Rotem 2011	No matching at baseline. Adjusted analysis only.
Helfrich 2011	<12 months follow up
Henwood 2015	mixed methods, not randomized
Herinckx, 1997	Irrelevant outcome; same population as Clarke 2000
Herman, 2000	Irrelevant outcome
Hersh 2011	Irrelevant outcome
Hickert 2011	Irrelevant design
Holter 1998	Same population as Susser 1997. No new information.
Housing homeless…	Irrelevant design
Howard 2010	no housing outcome
Hser, 2006	Irrelevant outcome
Hultman, 1995	Irrelevant outcome
Humphreys, 1998	Irrelevant outcome
Hwang 2011	No matching at baseline. Adjusted analysis only.
Jacob 2012	Length of follow‐up not reported
Jarrett 2012	<12 months follow up
Jason 2015	no housing outcomes
Johnsen, 1999	Irrelevant outcome
Kaplan, 1997	Irrelevant design
Kashner, 2002	Irrelevant comparison
Katz 2015	Not a study
Kerby, 1993	Irrelevant outcome
Kirby, 1999	Irrelevant outcome
Klein, 1996	Irrelevant outcome
Koffarnus 2011	Irrelevant outcome
Koffarnus 2013	Irrelevant outcome
Korr, 1995	Irrelevant outcome
Kosa, 2007	Irrelevant outcome
Lafave, 1996	Irrelevant population: not homeless or clearly at risk of becoming homeless
Lako 2013	<12 months follow up
Lam, 1995	Irrelevant outcome (<12 months)
Lam, 1998	Irrelevant outcome
Lam, 1999	Irrelevant outcome (<12 months)
Lam, 2000	Irrelevant outcome
Langle, 2006	Irrelevant outcome
Lapham, 1993	Irrelevant outcome (no data)
Lapham, 1995	Irrelevant outcome
Larimer, 2009	Irrelevant outcome (<12 months)
Lattimore 2013	quasi experimental study design
Lcingle, 2006	Irrelevant outcome (Langle 2006)
Leff, 2009	Review
Lehman, 1995	Irrelevant design
Lutze 2014	Quasi‐experimental design
Maguire, 2012	Lacking information, no answer from author
Malcolm, 2004	Irrelevant outcome
Management of…	Irrelevant design
Maone, 2008	Irrelevant outcome (no data)
Marcenko, 1994	Irrelevant outcome
Marcshall, 1995	Irrelevant outcome
Marcus 2012	Related to Rosenheck 2003, but no additional information
Mares 2011	quasi experimental study design
Masson, 2004	Irrelevant outcome
McCormack 2013	no housing outcomes, <12 months follow up
McGeary, 1999	Irrelevant outcome
McGlynn, 1993	Irrelevant outcome (same population as Burnam 1995)
McGuire 2011	adjusted analysis, not matched baseline characteristics
Miescher, 1996	Irrelevant outcome
Milby, 2000	Irrelevant outcome (<12 months) (same population as Milby 2003)
Milby, 2001	Irrelevant outcome
Milby, 2002	Irrelevant outcome (no data)
Milby, 2004	Irrelevant outcome
Milby, 2008	Irrelevant outcome
Morris, 2001	Irrelevant design
Morrissette, 2000	Irrelevant design
Murdoch 2011	no housing outcomes, wrong intervention
Nunez 2013	No matching at baseline. Adjusted analysis only.
Nuttbrock, 1997	Irrelevant design
Nuttbrock, 1998	Irrelevant design
Nuttbrock, 2002	Irrelevant intervention
Nyamathi, 2009	Irrelevant outcome
Odom, 2006	Irrelevant outcome
Orwin, 1994	Irrelevant design
Orwin, 1998	Irrelevant outcome
Orwin, 1999	Irrelevant outcome
Orwin, 2000	Irrelevant outcome
O'Toole 2010	no housing outcome
O'Toole 2015	<12 months follow up
Padgett 2010	no outcome on housing stability
Padgett 2011	no outcome on housing stability
Padgett, 2006	Irrelevant outcome (same population as Tsemberis 2004)
Parker 2012	<12 months follow up
Parsell 2014	no control group
Patterson 2013	qualitative
Piat, 2009	Irrelevant outcome (no data)
Pope, 1993	Irrelevant outcome
Raczynski, 1993	Irrelevant outcome
Rad 2010	not empirical
Rahav, 1995	Irrelevant outcome
Rapp, 2006	Irrelevant outcome (no data)
Reback 2010	no housing outcome
Rich, 2005	Irrelevant outcome (<12 months)
Rivas‐Vazquez, 2009	Irrelevant outcome
Rosenblum, 2002	Irrelevant outcome (<12 months)
Rosenblum, 2005	Irrelevant outcome
Rosenheck, 1997	Irrelevant outcome (<12 months)
Rosenheck, 1998	Irrelevant design
Rosenheck, 2003b	Irrelevant design
Rosenheck, 2007	Irrelevant design
Rotheram‐Borus, 2009	Irrelevant intervention; irrelevant design
Sacks, 2003	Irrelevant outcome
Sacks, 2004	Irrelevant outcome
Sadler, 2007	Irrelevant outcome (no data)
Sadow, 1993	Irrelevant outcome
Savage, 2008	Irrelevant design
Schonfeld, 2000	Irrelevant design
Schoppelrey, 2002	Irrelevant outcome
Schumacher, 1995	Irrelevant design
Schumacher, 1995a	Irrelevant design
Schumacher, 2000	Irrelevant outcome
Schumacher, 2001	Irrelevant design (no article/report)
Schumacher, 2002	Irrelevant outcome
Schumacher, 2003	Irrelevant outcome
Schutt, 1997	Irrelevant outcome
Schutt, 2007	Irrelevant outcome
Seidman, 2003	Irrelevant outcome
Sheridan, 1993	Irrelevant outcome
Shern, 1997	Review
Shumway, 2008	Irrelevant outcome
Simboli, 1996	Irrelevant design (comparison)
Skinner, 2005	Irrelevant outcome
Skobba 2013	Irrelevant design, no pre measurements
Skobba, 2008	Irrelevant outcome
Slesnick 2012	no control group
Slesnick 2013	<12 months follow up
Slesnick 2013	no housing outcome
Slesnick, 2005	Irrelevant outcome
Slesnick, 2006	Irrelevant outcome
Slesnick, 2007	Irrelevant outcome
Slesnick, 2008	Irrelevant outcome (<12 months)
Slesnick, 2009	Irrelevant outcome
Smelson 2013	no housing outcome
Smith, 1995	Irrelevant outcome
Smith, 2001	Irrelevant outcome
Solomon, 1994	Irrelevant outcome
Solomon, 1995	Irrelevant outcome
Sosin 2012	no housing outcome
Srebnik 2013	no housing outcome
Stahler, 1993	Irrelevant outcome
Stahler, 1995	Irrelevant outcome (<12 months)
Stahler, 2005	Irrelevant outcome
Starks 2012	no housing outcome
Stecher, 1994	Irrelevant outcome
Stevens, 1997	Irrelevant outcome (<12 months)
Tavecchio, 1999	Irrelevant design
Taylor 2014	Quasi experimental (propensity score matching)
The invisible children… the plight of the homeless teenager	Irrelevant design
Thompson 2011	no housing outcome
Tollett, 1992	Irrelevant outcome
Tollett, 1995	Irrelevant outcome
Tomita 2011	No extra inormation for Herman 2011
Tommasello, 2006	Irrelevant design
Tracy, 2007	Irrelevant outcome
Tsai 2010	No matching at baseline. Adjusted analysis only.
Tsai 2011	no control group
Tsemberis, 2003	Population same as Shern 2000 & Tsemberis 2004
Tyler 2014	No housing outcome
United States 1984	Irrelevant design
Upshur 2015	< 12 months follow‐up
van der Poel, 2006	Irrelevant design
Vet 2013	duplicate
von Rad 2010	duplicate
Vuchinich, 2009	Irrelevant outcome; same population as Milby 2005 & Kertesz 2007
Wade 2009	No matching at baseline. Adjusted analysis only.
Washington, 2009	Irrelevant outcome (<12 months)
Washington, 2009a	Irrelevant outcome (no data)
Wechsberg, 2004	Irrelevant outcome (<12 months)
Weissman, 2005	Irrelevant design
Westermeyer 2013	Quasi‐experiemental design
Willenbring, 1990	Irrelevant outcome
Winn 2013	wrong study design, no housing outcome
Witbeck, 2000	Irrelevant outcome
Wolff, 1997	Population same as Morse 1997. No new information.
Yanos, 2007	Irrelevant outcome; population same as Tsemberis 2004

## Appendix 6: Risk of bias in included studies

### Table 6.1: Risk of bias in included studies

**Table:**

**Author, year (Study ID)**	**Selection bias** **(random sequence generation)**	**Selection bias** **(allocation concealment)**	**Performance** **bias**	**Detection bias –** **subjective** **outcomes**	**Detection bias ‐** **objective outcomes**	**Incomplete** **Outcome data**	**Reporting** **bias**	**Other bias**
Aubry 2015 (42)	Low risk	Low risk	High risk	High risk	High risk	Low risk	Low risk	Low risk
Basu 2011 (43)	Low risk	Low risk	HR	High risk	Low risk	Low risk	Low risk	Low risk
Bell 2015 (44)	Unclear	Unclear	Unclear	Unclear	Low risk	Low risk	Low risk	Low risk
Bond 1990 (45)	Low risk	Low risk	Unclear	High risk	High risk	High risk	Low risk	Low risk
Grace 2014(46)	High risk	High risk	High risk	High risk	Unclear	High risk	Low risk	High risk
Chapleau 2012 (47)	Unclear	Unclear	High risk	High risk	Low risk	High risk	Low risk	High risk
Clarke 2000 (48)	Unclear	Unclear	Unclear	Unclear	Unclear	Unclear	Low risk	Low risk
Conrad 1998 (49)	Unclear	Unclear	Unclear	Unclear	Unclear	Low risk	Low risk	Low risk
Cox 1998 (50)	Unclear	Unclear	Unclear	High risk	High risk	High risk	Low risk	Low risk
Drake 1998 (52)	Unclear	Unclear	Unclear	Unclear	Unclear	Low risk	Low risk	Low risk
Essock 2006 (53)	Low risk	Unclear	Unclear	High risk	High risk	Low risk	Low risk	Low risk
Garety 2006 (54)(54)	Low risk	Low risk	High risk	High risk	High risk	Low risk	Low risk	Low risk
Goldfinger 1999 (55)	Unclear	Unclear	Unclear	High risk	Unclear	Low risk	Low risk	Low risk
Herman 2011 (56)	Low risk	High risk	High risk	High risk	Low risk	Low risk	Low risk	Low risk
Hurlburt 1996 (27)	Unclear	Unclear	Unclear	Unclear	Unclear	Unclear	Low risk	Low risk
Kertesz 2007 (58)	Unclear	Unclear	Unclear	Unclear	Low risk	High risk	High risk	Low risk
Killaspy 2006 (59)	Low risk	Low risk	High risk	High risk	High risk	Low risk	Low risk	Unclear
Lehman 1997 (60)	Low risk	Unclear	High risk	High risk	Unclear	Low risk	Low risk	High risk
Levitt 2013 (62)	High risk	Low risk	High risk	High risk	High risk	Low risk	Low risk	High risk
Lipton 1988 (63)	Unclear	Unclear	Unclear	Unclear	Unclear	High risk	High risk	High risk
Marshall 1995 (64)	Low risk	Low risk	High risk	High risk	Unclear	Unclear	Low risk	High risk
McHugo 2004 (65)	Unclear	Unclear	Unclear	Unclear	Unclear	High risk	Low risk	Low risk
Milby 1996 (67)	Low risk	Unclear	High risk	High risk	Low risk	High risk	Low risk	Low risk
Milby 2003 (66)	Unclear	Unclear	Unclear	Unclear	Low risk	Low risk	Low risk	High risk
Milby 2010 (68)	Low risk	High risk	High risk	High risk	Low risk	Low risk	Low risk	Low risk
Morse 1992 (39)	Unclear	Unclear	High risk	High risk	Low risk	Low risk	Low risk	Low risk
Morse 1997 (40)	Unclear	Unclear	High risk	High risk	Unclear	Low risk	Low risk	Low risk
Morse 2006 (69)	Unclear	Unclear	High risk	High risk	High risk	High risk	Low risk	High risk
Nordentoft 2010 (70)	Low risk	Low risk	High risk	High risk	High risk	High risk	Low risk	Low risk
Nyamathi 2015 (83)	Low risk	Unclear	Unclear	Unclear	Unclear	Low risk	Low risk	Low risk
Rosenheck 2003 (71)	Low risk	Low risk	High risk	High risk	Unclear	Low risk	Low risk	Low risk
Samuels 2015 (72)	Low risk	Low risk	Unclear	Unclear	Unclear	High risk	Low risk	High risk
Shern 2000 (73)	Unclear	Unclear	High risk	High risk	Unclear	Low risk	Low risk	Low risk
Slesnick 2015 (74)	Low risk	Low risk	Unclear	Unclear	Unclear	Low risk	Low risk	Low risk
Smith 1998 (75)	High risk	Unclear	Unclear	Unclear	High risk	Low risk	Low risk	High risk
Solomon 1995 (76)	Unclear	Unclear	Unclear	Unclear	Unclear	High risk	Low risk	Low risk
Sorensen 2003 (77)	Unclear	Unclear	High risk	High risk	Low risk	Unclear	Low risk	Low risk
Sosin 1995 (26)	High risk	High risk	Unclear	Unclear	Unclear	High risk	Low risk	Low risk
Stefancic 2007 (78)	Unclear	Unclear	Unclear	Unclear	Unclear	High risk	Low risk	High risk
Susser 1997 (79)	Unclear	Unclear	High risk	High risk	Low risk	Low risk	Low risk	Low risk
Toro 1997 (80)	Unclear	Unclear	Unclear	High risk	High risk	Low risk	Low risk	Low risk
Tsemberis 2004 (24)	Unclear	Unclear	Unclear	High risk	High risk	High risk	Low risk	Low risk
Wolitski 2010 (81)	Low risk	Unclear	High risk	High risk	Unclear	Low risk	Low risk	Low risk

## Appendix 7: Characteristics of included studies

**Table:**

**Aubry 2015 (42)**	
**Methods**	Non‐blindied RCT, Randomized N=2148, Housing First: N=1198, Usual services: N=950 5 Canadian cities: Vancouver, British Columbia; Winnipeg, Manitoba; Toronto, Ontario; Montreal, Quebec; and Moncton, New Brunswick (not stratified according to need). Data collected between October 2009 and June 2011
**Participants**	**Eligibility**: Legal age of majority, absolutely homeless or precariously housed, presence of a mental illness with or without a concurrent substance use disorder (evaluated using the Mini International Neuropsychiatric Interview). Excluded if not legally residing in Canada or were a current client of an ACT or ICM team. High needs if they had a current psychotic disorder or bipolar disorder based on the MINI, an MCAS score of 62 or lower, indicating at least moderate disability and at least one of the following: 2 or more hospitalizations for mental illness in any 1 of the last 5 years, recent arrest or incarceration, or comorbid substance use based on the MINI. All other participants were classified as having moderate needs. Sample: 34% with psychotic disorder, 71% with nonpsychotic disorder. 67% also diagnosed with substance use problem, more than 90% reported having 1 or more chronic physical health condition. 82% absolutely homeless, 18% precariously housed, living in rooming houses or single‐room occupancy hotels and having experienced 2 episodes of homelessness in last year. Average total time homeless =58 months
**Interventions**	Housing first + Assertive community treatment (ACT) or Intensive Case Management (ICM) [BN1] · Recovery oriented culture · Based on consumer choice for all services · Only requirements: income paid directly as rent; visited at a minimum once a week for pre‐determined periods of follow‐up supports · Rent supplements in private market: participants pay 30% or less of their income or the shelter portion of welfare · Treatment and support services voluntary clinicians/providers based off site · Legal rights to tenancy (no head leases with agency rather than individual) · No conditions on housing readiness · Program facilitates access to housing stock · Apartments are independent living settings primarily in scattered sites · Services individualised, including cultural adaptations · Reduce the negative consequences of substance use · Availability of furniture and possibly maintenance services · Tenancy not tied to engagement in treatment **ACT** · Recovery‐oriented ACT team, with participant/staff ratio of 10:1 or less and includes a psychiatrist and a nurse · Program staff are closely involved in hospital admissions and discharges · Available 24 h crisis coverage Assessment of program fidelity conducted during the study found good fidelity overall, with 78% of the 38 fidelity scale items rated higher than 3 on a 4‐point scale on the second fidelity assessment, 24 to 29 months after the start of the programs (30). (+ ICM) **Conditionality of tenancy:** Positive drug/alcohol test transported to a shelter or other housing and wage was lowered **Housing provision:** Scattered‐site supportive housing, low‐barrier permanent housing in independent units. Participants paid up to 30% of their income toward rent with monthly rent supplement of 375‐600 USD. **Segregation:** No **Case management intensity:** Tested 3xweek (wks 1‐24), 1xweek (25‐52), once bimonthly (52‐78) ***(+ ACT)*** ***Conditionality of tenancy*:** compliance with rental lease and weekly visits with case manager ***Housing provision*:** self‐contained units in a single building with common areas and meals provided. Mostly private‐market scattered‐site units. ***Segregation*:** NA ***Case management intensity*:** Client staff ratio: 12:1 onsite support staff including psychiatrist, social worker, nurse, peer support, pharmacy and activity planning **Usual services** Participants had access to housing and support services through other programs in their respective communities, such as group homes, congregate supportive housing, and mental health support services, including other ICM programs. Conditionality of tenancy: NA Housing provision: NA Segregation: NA Case management intensity: NA
**Outcomes**	**Stably housed**: days in stable residence (Residential time‐line follow‐back intventory) **Other**: QoL (EuroQoL 5 Dimensions) employment, offence rates (property crimes, alcohol and drug related crimes, violent crimes, community integration (psychological integration, physical integration) **Measurement**: assessment at 9, 12, 18, 24 months
Basu 2012 (43)	
**Methods**	Randomized N=407, Analyzed N=405, CM (N=201) vs UC (N=204), Chicago, Ill, USA Participants enrolled from Sept 2003 to May 2006 with follow‐up provided through December 2007. 2 primary study sites (a public teaching hospital and a private, non‐profit hospital), 2 respite sites, 10 housing agencies.
**Participants**	**Eligibility:** At least 18 years of age, fluent in English or Spanish, without stable housing during the 30 days prior to hospitalization, were not the guardian of minor children needing housing, and had at least one of 15 specified chronic medical illnesses documented in the medical record (hypertension or diabetes requiring medication, thromboembolic disease, renal failure, cirrhosis, congestive heart failure, myocardial infarction, atrial or ventricular arrhythmias, seizures within the past year or requiring medication for control, asthma or emphysema requiring at least 1 ER visit or hospitalization in the past 3 years, cancer, gastrointestinal tract bleeding (other than from peptic ulcer disease), chronic pancreatitis, HIV ‐ chosen for the increased mortality risk they pose for homeless). Patients were ineligible if their hospital physician found them incapable of self‐care upon hospital discharge **Sample description:** Age (Mean (SD)): CM=47 (8.2), UC=46 (9.1) Gender (% male): CM= 74%, UC = 79% Ethnicity (% African American): CM= 81%, UC = 76% Substance abuse Alcohol intoxication past 30 days (%): CM= ‐ 43%, UC = 37% Drug use past 30 days (%): CM= 60%, UC = 58% Mental illness (%): Major Depression: CM = 40%, UC = 45%, Other depression: CM = 33%, UC = 33%, Panic disorder: CM =15%, UC = 18%, Other anxiety: CM =40%, UC =45% Homeless status (% streets last 30 days): Tx= 41%, Control=48% Criminal: NR Other: Veteran (%): CM= 9%, UC=10%, HIV (%): CM= 37%, UC=35% No stat sig diff bw populations at baseline
**Interventions**	**Case management:** from on‐site intervention social worker (provision of transitional housing at respite care centres, subsequent placement in stable housing, and case management) CM provided on‐site at primary study sites, respite care facilities and stable housing cites. **Conditional tenancy:** not reported **Housing provision:** Housing first model ‐ options provided by 10 agencies offering group or single living. Housing decisions based on availability, sex, sobriety, HIV status and geographic preference. **Segregation:** not reported **Case management intensity:** participants had contact with case managers at least biweekly. Case manager had weekly team meetings to coordinate the housing, social service and medical care needs **Usual services:** referred back to original hospital social worker, usual discharge planning services with no continued relationship after discharge. Typically provided transportation to an overnight shelter. People with HIV had access to case management after discharge through another program, others had access to general case management services. access to respite/stable housing was unaffected by participation in the study. **Conditionality of tenanc** y: NA **Housing provision**: NA **Segregation**: NA **Case management**: NA
**Outcomes**	**Homeless**: days homeless, days in respite, shelter, other housing **Other**: Quality of life, days in residential substance abuse treatment, substance abuse treatment visits, days in jail or prison Follow up at 1, 3, 6, 9, 12, 18 months
Bell 2015 (44)	
**Methods**	Non‐blinded RCT, Randomized N=1380, Analysed N=1120, KCCP: N=557, UC: N=563 King County, Washington State, USA, 2008‐2011
**Participants**	**Eligibility** : Enrolment in the Medicaid categorically Needy program, King County, WA resident, evidence of at least one chronic physical condition and a mental health problem, substance abuse disorder, or both recorded in state administrative databases, predicted future health care costs at least 50% higher than those of average Medicaid supplemental security income (SSI) recipient **Sample:** Age (mean (SD)): KCCP: 51 (11) UC: 51 (10), Gender (% male): KCCP: 48%, UC: 46%, Ethnicity (% African American): KCCP: 26%, UC: 27%, Mental illness (serious at baseline %): KCCP: 49%, UC: 50%, Substance abuse (need for alcohol/drug tx at baseline, %): KCCP: 44%, UC: 49%, Homeless status (mean days homeless per 100 mos (SD)): KCCP: 11(30), UC: 13(33), Criminal (mean arrests per 1000 mos (SD)): KCCP: 18 (59), UC: 22(94), Other: NA
**Interventions**	**King County Care Partners (nurse‐led case management) (KCCP):** Participants received intensive care management from a team comprised of 3 fulltime registered nurses, two social workers with drug/alcohol treatment training and a BSc level chemical dependency counsellor. All participants completed a 60‐90 minute comprehensive in‐person assessment of their medical and social needs and set goals. The care manager joined participant at one or more clinic appointments with primary care provider. Care management teams provided participant with chronic disease self‐management coaching; frequent in‐person and phone monitoring; connection to community resources; and coordination of care across the medical/mental health systems. **Usual Care (UC):** Participants received Medicaid‐covered case as usual. Control group members offered intervention after 1 year waiting period (only 5 individuals accepted)
**Outcomes**	**Homelessness**: Mean number of homeless months per 1000 months, Proportion of participants with any homeless months **Other**: Criminal arrests, Death Measurements were taken at 24 month follow‐up
Bond 1990 (45)(45)	
**Methods**	**Semi‐blinded RCT**, Randomized N=?, Analyzed N=88, ACT (n_1_=45) vs. DI+AC (n_2_=43)
**Participants**	**Eligibility**: homeless, mental illness **Sample description**: mean age 34, women 43%, Schizophrenia 38%, Schizoaffective disorder 30%, Affective disorder 22%, Personality disorder 5%, Other mental illness 7%, Alcohol abuse 18%, Drug abuse 8%, 7‐9% of participants were not domiciled at baseline, **Location:** Chicago, Illinois, USA
**Interventions**	**Assertive Community Treatment (ACT)** No indication that the program deviated from the original model. The program was not closely connected with a housing program. *Conditionality of tenancy*: not applicable *Housing provision*: not applicable *Segregation*: not applicable *Case management intensity*: (high) caseload 1:10, persistent & continuous approach **Drop‐in centers (DI) and aftercare services (AC)** DI‐centers provide informal meeting places to experience fellowship, food, and recreation. Less demanding expectations than most day treatment programs. Clients are not “admitted” or “discharged”, they are not required to participate in specific groups, to be actively involved in rehabilitation, or even to attend regularly. The DI‐centers offer a range of social and recreational opportunities, have self‐help ethos in which clients play a major role in club decision‐making. Clients‐staff ratios are high, but with no requirements for frequent contacts. The DI‐center, a readily accessible, low‐expectation drop‐in program, was conducted at the agency's main location. It had an average daily attendance of over 50 clients during the study period. The program operated during late afternoon and evening hours and on weekends, with several professional staff on site at all times when the program was open. Staff responsibilities were primarily to facilitate group activities, intervene in the case of major disruptions and crisis, converse with clients, and make referrals if necessary. The DI‐center was staffed by a team of five paid workers, supplemented by numerous volunteers and trainees. The full‐time coordinator was a highly experienced master's‐level social worker. The remaining four positions (constituting two full‐time equivalents) were filled by a master's‐level social worker with several year's of experience; a bachelor's‐level artist who had also worked for many years as an inpatient psychiatric aide; an advanced graduate student in clinical psychology who had worked in the field for several years; and a bachelor's‐level schoolteacher, active in the local chapter of the Alliance for the Mentally Ill. The program was not closely connected with a housing program. *Conditionality of tenancy*: not applicable *Housing provision*: not applicable *Segregation*: not applicable *Case management intensity*: medium, caseload <1:20, available at weekends
**Outcomes**	**Stably housed**: stable community housing at intake (group home, hotel/SRO, supervised apartment, unsupervised apartment), at follow up (own apartment, intermediate care facility, halfway house, board‐and‐care facility). **Homeless**: Not in stable community housing at intake (undomiciled, with parents, crisis housing, nursing home, other), at follow up (with relatives, streets, hospital, deceased, unknown). **Measurement**: Interviews. Assessments at intake and 12 months follow up. **Other**: contact with police (arrests), quality of life, areas of difficulty, hospitalization, life satisfaction index
Chapleau 2012 (47)	
**Methods**	RCT. Randomized N=57, Analyzed, N=57, CM‐OT (N=29) vs CM‐TNOT (N=28 midsize, Midwestern city, USA
**Participants**	**Eligibility**: Diagnosed with severe mental illness, homeless or at risk of homelessness **Sample**: Age (mean (SD)): Tx= 47.31(12.34), Control= 45.53 (9.21) Gender (% female): Tx= 55%, Control=54% Ethnicity: NR Substance abuse: Tx=12% active substance abuse, 67% history of substance abuse; Control= 24% with active substance abuse, 88% history of substance abuse. Mental illness (%): Tx= 48% schizophrenia, 25% depressive or bipolar disorders, Control= 58% schizophrenia, 27% depressive or bipolar disorders. Homeless status: NR Criminal: NR
**Interventions**	**Case management services with Occupational Therapist consultant (CM‐OT)** ‐ Occupational therapists were added to regular case management to stabilize or improve housing status and achieve client goal attainment. ‐ OT consultant used Canadian Occupational Performance measure (COPM) to provide each client with the opportunity to identify personally‐meaningful goals for case management intervention. ‐ All clients received indepth evaluation by OT consultant – assessing cognitive functioning ‐ OT consultant and client collaborated to determine and prioritize treatment goals. ‐ Experiemental group considered to receive regular on‐going contact with the OT consultant both through case manager and directly in groups, individual meetings and home‐based services. ‐ In order to address the treatment goals the clients identified during the evaluation process, the OT consultant developed and facilitated weekly activitiy groups and cshared client pgress and relevant client‐group observatrions with case managers during weekly staff meetings. ‐ Group topics included diabetes education, life skills management, exercise, relaxation, crafts, gardening and therapeutic horseback riding. Additionally the OT consultant modified the grocery shopping outing which had consisted of dropping the clients off at the entrance of the store and waiting for them to complete their purchases before returning them to the drop in center. The outing developed into agroup format which included assisting the clients in preparing their lists prior to the trip, teaching money management skills and techniques (use of coupons and newpaper inserts of weekly sales) and accompanying them throughout the store to provided needed cues or assistance to complete the shopping task. *Conditionality of tenancy: NA* *Housing provision: NA* *Segregation: NA* *Case management intensity: seen once weekly for medication monitoring and money management* **Case management services with traditional non‐Occupational Therapist (CM‐TNOT)** ‐ All clients received indepth evaluation by OT consultant – assessing cognitive functioning ‐ OT consultant and client collaborated to determine and prioritize treatment goals. ‐ Received traditional non‐OT case management services with minimal contact from the OT consultant. ‐ Run by private community mental health center under collaborative agency partnership between mental health agency and homeless center. Community support program relied on case management services in which clients were generally seen once weekly for medication monitoring and money management by a paraprofessional case manager, most of whom were recent college graduates with limited work experience in mental health or any other field. Education ranged from GED to masters in social work and experience as case manager ranged from less than one month to more than 8 years. None of case managers were licensed or certified professionals in nursing, social work and none had formal education or certification in case management. ‐ High caseloads and frequent staff turnover ‐ Case managers generally functioned as primary therapists for the client addressing basic needs money management, and safety. ‐ Case managers were able to bill for either providing services to clients or teaching them needed skills, but because state funding allowed for a higher reimbursement rate for providing services rather than teaching skills, case managers were generally encouraged to probide services (e.g. drive them to appointments instead of team them how to use public transportation) ‐ Lack of rehabilitative approach. *Conditionality of tenancy: NA* *Housing provision: NA* *Segregation: NA* *Case management intensity:* *seen once weekly for medication monitoring and money management; High caseloads and frequent staff turnover*
**Outcomes**	Housing status (13‐point scale): Incarceration, State psychiatric hospital, psychiatric hospital, nursing home, street homeless, other head of household homeless, motel, sheltered homeless, room and board assistance, transitional housing, group home, other head of household independent, independent
Clarke 2000 (48)	
**Methods**	**Non‐blinded RCT** Randomized N=178, begun treatment N=163, CACT+ACT (n_1_=114) vs. UC (n_2_=49)
**Participants**	**Eligibility**: mental illness, at risk of becoming homeless, **Sample description**: Age (mean): 40 Gender (% women): 0% Ethnicity (% African American): 75% Mental illness (%): 66%, dual disorder 25%, mental health varied from fairly healthy to severe mental problems Substance abuse (%): alcohol dependence 47%, cocaine dependence: 38%, heroine dependence: 15%, dependence on two or more substances Homeless status: 100% homeless Criminal: NR **Location**: Portland, Oregon, USA
**Interventions**	**Consumer staffed Assertive Community Treatment (CACT) or Assertive Community Treatment (ACT)** Staff were hired, trained, and supervised by a local consumer‐run mental health agency, which also administratively operated the two programs. Each team consisted of four full‐time and one part‐time case manager, one of whom was the team leader. Staff members on the consumer‐staffed team were self‐identified mental health consumers with a *DSM‐III‐R* axis I diagnosis. Over the life of the project, the majority of the staff on this team had a diagnosis of bipolar disorder (50 percent); other diagnoses included major depression, schizoaffective disorder, and cyclothymia. Most members of the consumer‐staffed team held a bachelor's degree. The consumer‐staffed team had on average more previous experience in the mental health field (8.6 years, compared with four years). No indication of any deviation from the original ATC‐model regarding the second team. *Conditionality of tenancy*: no direct information but non‐conditionality is indicated …a comprehensive array of services for meeting client needs; supported housing based on consumers choice. *Housing provision*: no specific information *Segregation*: no information *Case management intensity*: very high, caseload 1:4.6 and 1:5.4, availbility 24/7 **Usual Care** Services from agencies in the Portland metropolitan area, most subjects received services from community mental health centers and smaller, more specialized agencies. *Conditionality of tenancy*: not applicable *Housing provision*: not applicable *Segregation*: not applicable *Case management intensity*: low, case load 1:26.9, availability 24/7
**Outcomes**	**Homeless**: not defined, at least one episode of homelessness **Stably housed**: not homeless (extrapolated), not defined **Other:** Arrests ‐ number of days to first arrest, total number of clients arrested **Measurement**: assessments at intake and 12 months follow up.
Conrad 1998 (49)	
**Methods**	**Semi‐blinded RCT** Randomized N=?, Analyzed N=358, CMRC (n_1_=178) vs. VA Care (n_2_=180)
**Participants**	**Eligibility**: male veterans, homeless, drug/alcohol dependence, possible concurrent mental illness **Sample description**: mean age 40, women 0%, alcohol dependence 47%, cocaine dependence: 38%, heroine dependence: 15%, dependence on two or more substances 66%, dual disorder 25%, mental health varied from fairly healthy to severe mental problems **Location**: Chicago, Illinois, USA
**Interventions**	**Case Managed Residential Care for Veterans (CMRC)** Residential phase: (month 1‐6) case management (assessment & evaluation, service planning, service linkage, service monitoring); residential housing (treatment planning); substance abuse counseling; sobriety monitoring (relapse prevention training, basic skills training), vocational services, housing placement (self‐help services, material assistance e.g. bus fare), and referral to multiple support services. Community phase: (month 7‐11) community living, continued case management. *Conditionality of tenancy*: possibly during residential phase, probably not during community phase *Housing provision*: care giver provided during residential phase, probably not during community phase *Segregation*: probably during residential phase, probably not during community phase *Case management intensity*: partly high; 1:10 during residential phase, 1:25‐30 during community phase **Customary VA Care** Customary inpatient treatment, wards (14‐21 days): substance abuse education, group therapy, self‐help services, recreational/occupational therapy, medical and other health care, material assistance (e.g. bus fare), referral to multiple support services. Customary community care, VA and community outpatient settings (12 months): other services as needed, half‐way houses. *Conditionality of tenancy*: probably abstinence contingent when in inpatient wards, possibly in community outpatient settings *Housing provision*: probably care giver provided during the inpatient treatment, possibly in community outpatient settings *Segregation*: probably during inpatient treatment, possibly in in community outpatient settings *Case management intensity*: no specific information
**Outcomes**	**Stably housed**: not homeless (extrapolated) **Homeless**: literal homelessness (indoor public place, subway or bus, abandoned building, car or other private vehicle, and outdoor place) **Other:** medical, legal, drug, alcohol symptoms ‐ Addiction severity (Addiction severity index) Employment **Measurement**: number of nights previous 60 days, any night homeless. Binary outcomes extrapolated from consitnuous outcomes. Assements at intake, 3, 6, 9, 12, 18, and 24 months follow up.
Cox 1998 (50)	
**Methods**	**Non‐blinded RCT** Randomized N=?, Analyzed N=298, ICM (n_1_=150) vs. UC (n_2_=148)
**Participants**	**Eligibility**: homeless (or at risk) high frequency users of detox center **Sample description**: Age (mean): 42.9 Gender (% women): 19% Ethnicity (% African American): 17% Mental illness (%):NR Substance abuse (%):alcohol only as 1st or 2nd choice 72%, alcohol as 1st or 2nd choice 95%, heroin as 1st or 2nd choice 11%, coke/crack as 1st or 2nd choice 9%. Homeless status (mean total months homeless): 69.4 ± 86.7 mos Criminal: NR **Location**: Seattle, Washington, USA,
**Interventions**	**Intensive Case Management (ICM)** No indication of deviation from standard ICM. Long‐term open ended, outreach service focused on system advocacy and linkage activities. Clinically oriented, aiming to strengthen client's social and personal skills and encourage increased client autonomy. Clients helped determine which need and interests would be served first. Clients were expected to take as strong a role as they were capable of in addressing their problems. Generic treatment program goals: stabilize client's financial condition and housing status, encourage substance use reduction. *Conditionality of tenancy*: no specific information regarding conditionality of housing, yet provision of the service was not conditional on client behavior and there was no requirement that clients maintained sobriety in order to continue in the program. *Housing provision*: no specific information, yet In practice, a large portion of the case manager's time was spent in acquiring housing for clients, and an even larger portion was spent in helping maintaining them in housing once it was arranged. *Segregation*: no specific information *Case management intensity*: medium, case load 1:15, frequency and duration of contacts according to clients needs and capacities **Usual Care** No information, but control subjects were free to seek treatment from other sources in any way they wished, and some did. *Conditionality of tenancy*: no information *Housing provision*: no information *Segregation*: no information *Case management intensity*: no information
**Outcomes**	**Stably housed**: nights in own residence past 60 days (hotel/motel, own SRO room, own apartment, etc) **Homeless**: nights unhoused in last 60 days (shelters, outside, in abandoned buildings, etc). **Other**: alcohol use, employment **Measurement**: Interviews guided by Personal History Form (PHF). Binary outcome extrapolated from continuous outcome. Assessments at intake, 6, 12 and 18 months follow up.
Drake 1998 (52)	
**Methods**	**Singel‐blinded RCT** Randomized N=223, Analyzed N=203, ACT‐I (n_1_=105) vs. SCM+ (n_2_=98)
**Participants**	**Eligibility**: mental illness, dependence or abuse of alcohol and/or other drugs, at risk or homeless (homelessness not required) **Sample description**: Age (mean): 34 Gender (% women): 25.6% Ethnicity (% white): 96.4% (our calculation) Mental illness (%):schizophrenia 53%, schizoaffective disorder: 22.4%, bipolar disorder 24.2%, Substance abuse (%):alcohol abuse 73% (AUS score m=3.3 sd=1.0), drug abuse 42% (DUS score m=2.3 sd=1.3) Homeless status: 1.8% (our calculation) Criminal: NR **Location**: New Hampshire, USA
**Interventions**	**Assertive Community Treatment (ACT‐I) with integrated mental health and substance abuse treatment** No indication of any deviation from original program. Service provided, in the community, assertive engagement, high intensity of services, small case loads, continuous 24‐hour responsibility, team approach, multidisciplinary team, close work with support system, continuity of staffing, direct substance abuse treatment by members of the team, use of stage‐wise dual‐disorders model, dual‐disorders groups, and an exclusive team focus on patients with dual disorders. *Conditionality of tenancy*: not applicable *Housing provision*: not applicable *Segregation*: not applicable *Case management intensity*: high, case load 1:12, availability 24/7 **Standard Case Management with most ACT‐principles (SCM+)** Standard case management incorporating most ACT principles, multidisciplinary SCM teams emphasized a team approach, delivered services in the community, worked with the client's support system, and vigorously addressed concurrent SUD. *Conditionality of tenancy*: not applicable *Housing provision*: not applicable *Segregation*: not applicable *Case management intensity*: low, case load 1:25
**Outcomes**	**Stably housed**: number of days past 6 months (180 days) living in stable community residences, including all independent living situations and community‐based group homes, but excluding hospital and jail stays, days homeless and in other institutional settings, such as shelters or nursing homes. . **Homeless**: not stably housed (extrapolated) **Other**: Life satisfaction (QOLI subscale), substance use, substance use treatment progression, community integration (QOLI subscale) **Measurement**: detailed chronological assessment of housing history and institutional stays, using a self‐report calendar supplemented by outpatient records and hospital records. Binary outcome extrapolated from continuous outcome. Assessments at intake, 6, 12, 18, 24, 30 and 36 months follow up.
Essock 2006 (53)	
**Methods**	Multi‐site RCT. Referred N=382, Randomized N=205, Analyzed N=198 ACT N=99 (Site 1: N=50, Site 2: N=49), SCM N=99 (Site 1:N=50, Site 2: N=49) Participants enrolled August 1993 ‐ July 1998
**Participants**	Eligibility: Major psychotic disorder (schizophrenia, schizoaffective disorder, bipolar disorder, or major depression with psychotic features); had an active substance use disorder (abuse or dependence on alcohol or other drugs within the past 6 months); had high service use in the past two years (two or more of the following: psychiatric hospitalizations, stays in a psychiatric crisis or respite program, emergency department visits, or incarcerations); were homeless or unstably housed; had poor independent living skills; did not have any pending legal charges, medical conditions, or mental retardation that would preclude participation; were scheduled for discharge to community living if they were an inpatient; and were willing to provide written informed consent. Sample: Age (mean): ACT: 36.4±7.9 years; SCM 36.6±7.7 Gender (% men): ACT: 71%, SCM: 73% Ethnicity (% African American): ACT: 59%, SCM: 51% Hispanic: ACT: 16%, SCM: 12%: Non‐hispanic Caucasion: ACT: 22%, SCM: 32%; other: ACT: 2%, SCM: 5% Mental illness (%): ACT: 72% schizophrenia and related disorder, 17% Affective disorder, 10% other, SCM: 81% schizophrenia and related disorder, 16% affective disorder, 2% other Substance abuse (%): ACT: 73% Alcohol abuse, 87% drug abuse, SCM: 76% alcohol abuse, 76% drug abuse (only significant difference between groups was on Substance abuse treatment scale) Homeless status (No. days spent in stable residence in past year, mean±SD): ACT: 154.0±151.9, SCM: 138±144.8 Criminal: not reported
**Interventions**	Assertive Community Treatment Assertive community treatment teams were trained by study authors. Training emphasized the essential features of ACT: lower staff to client ratio (1:10/15), delivery of most services in the community, shared caseload, 24 hours responsibility for clients, direct provision of most services. Training also included key components of integrated treatment: direct substance abuse treatment by members of the team, use of a stage wise co‐occurring disorders model, treatment groups for clients with co‐occurring disorders, and an exclusive team focus on clients with co‐occurring disorders. *Conditionality of tenancy*: NA *Housing provision: NA* *Segregation: NA* *Case management intensity*: 1:10/15 Standard Clinical Case Management (SCM) Teams composed of clinicians from different disciplines and emphasized a team approach where team members carried individual caseloads but discussed clients and reviewed cases together. Deliver at least some services in the community, clinicians worked with clients’ support systems and address substance use disorders, twice as heavy caseloads, provided fewer services directly. *Conditionality of tenancy*: NA *Housing provision: NA* *Segregation: NA* *Case management intensity*: 1:25 approximately
**Outcomes**	Housing status: number of days spent in a stable residence in the past year Other: Substance abuse: Alcohol use scale, number of days of alcohol use in past 6 months (self‐report) (score of 3 or higher indicates abuse), number of days of drug use in the past 6 months (self‐report) (score of 3 or higher indicates abuse), Substance Abuse Treatment Scale. Possible scores range from 1 to 8 with higher scores indicating more progress toward substance use remission and recovery. Psychiatric symptoms – Expanded Brief Psychiatric Rating scale. Possible scores range from 24 to 168 with higher scores indicating more symptoms Global Assessment Scale. Possible scores range from 1 to 100, with higher scores indicating better functioning General Life Satisfaction Scale (from Quality of Life Interview) Possible scores range from 1 to 7, with higher scores indicating more satisfaction with life in general
Garety 2006 (54)(54)	
**Methods**	**Single‐blinded RCT** Randomized N=144, Analyzed N=128, EOT (n_1_=71) vs. UC (n_2_=73) Participants recruited Jan 2000‐Oct 2001
**Participants**	**Eligibility**: mental illness, at risk of becoming homeless (homelessness not required), age 16‐40 **Sample Description**: Age (mean): 26 Gender (% women): 35% Ethnicity (% ethnic minority): > 50% Mental illness (%):schizophrenia 69% Substance abuse (%):patients with primary alcohol or drug addiction were excluded Homeless status: NR Criminal: NR **Location**: London, UK
**Interventions**	**Early onset team (EOT)** A case management like multi‐disciplinary team, 1 team leader, 1 part time consultant (2 sessions), 1 trainee psychiatrist, 1 half‐time clinical psychologist, 1 occupational therapist, 4 community psychiatric nurses, 2 healthcare assistants. Based on principles of assertive outreach, single point of access for all the mental health and social welfare needs of patients, extended‐hours service 5 days a week (8am‐8pm), open at weekends and holidays (9am‐17pm). Intervention specially adapted for a group with early psychosis and followed protocols and manuals from the Early Psychosis Prevention and Intervention Centre and, for cognitive‐behavioral therapy, pilot work conducted locally. Mix of medication management, cognitive‐behavioral therapy, vocational input and family intervention according to individual need. Emphasis on helping patient retain or recover functional capacity to return to study or work, to resume leisure pursuits and retain or re‐establish supportive networks. Family and carers support group established as was a social activity program open to all patients in the service. Staff were selected who had an interest in working with younger people and who were sensitive to the needs and concerns of the local minority ethnic population. *Conditionality of tenancy*: not applicable *Housing provision*: not applicable *Segregation*: not applicable *Case management intensity*: high, case load 1:10, availability less than 24/7 (not in vivo) **Usual care (UC)** Services provided through five mental health teams, each providing a range of assessments, treatments and continuing care to geographically defined sector. Sector teams comprised psychiatrists, psychiatric nurses, occupational therapists and part‐time clinical psychologists. Each of these sector community teams was associated with inpatient facilities on one of the three hospital sites. *Conditionality of tenancy*: not applicable *Housing provision*: not applicable *Segregation*: not applicable *Case management intensity*: no information (no assertive outreach)
**Outcomes**	**Stably housed**: “full recovery”, not clearly a relevant outcome **Homeless**: not stably housed (extrapolated) **Other:** Clinical state ‐ The Positive and negative Syndrome Scale; Overall functioning – The Global Assessment of Function; Depression – The Calgary Depression Rating Scale; Quality of life – The Manchester Short Assessment of Quality of Life **Measurement**: case note records, assessment at intake and 18 months follow up
Goldfinger 1999 (55)	
**Methods**	**Non‐blinded RCT** Randomized N= 118, Analyzed N=110, ECH+ICM (n_1_=53) vs. IL+ICM (n_2_=47) Participants recruited Jan 1991‐March 1992
**Participants**	**Eligibility**: homeless, mental illness **Sample description**: Age (mean): 38 Gender (% women): 28% Ethnicity (% African American): 41% Mental illness (%): schizophrenia 45%, schizoaffective disorder 17%, bipolar disorder 14%, major depression for 13%, 88 people with significant lifetime secondary axis I Substance abuse: 44 people identified as abusing alcohol or other drugs Homeless status: NR Criminal (% arrested/jailed at least once): 77% **Location**: Boston, Massachusetts, USA
**Interventions**	**Evolving consumer household staffed group homes with intensive case management (ECH+ICM)** No indication of deviation of ICM from original program. ECH is a shared housing arrangement intending to maximize independence and minimize the presumed risks of independent living. ECH staff members are trained to promote resident independence. Staff time is gradually reduced as the residents learned how to manage their house themselves. Residents are encouraged to take the lead in establishing their own house rules. House staff offered advice and support in this process. Other goals: reduce isolation, provide paraprofessional monitoring of the residents’ clinical condition, offer skills training in managing the house (e.g. paying the bill, negotiating with the landlord). All tenants paid rent, which they had not had to do in the homeless shelter. All had some sort of income support, and rents (including utilities) were set as a proportion (about one‐third) of that benefit amount. *Conditionality of tenancy*: Permanent secure housing without the requirement of treatment compliance, 30 percent of income is paid for rent and utilities at all placement sites, all are encouraged to participate in community mental health center programs after they were housed. Residents were required to maintain behavior that met landlord or co‐resident agreements. *Housing provision*: care giver provision of housing, Massachusetts Department of Mental Health made 120 units of housing available for the project. *Segregation*: probably not segregated housing, one‐ or two single room apartments in public housing subsidized by the Boston Housing Authority. *Case management intensity*: medium, case load 1:15, meetings > once a week. **Independent living with intensive case management (IL+ICM)** No indication of any deviation from original ICM program. Independent apartments, efficiency units operated by the Boston Housing Authority, apartment sites offered a voluntary weekly group but no other on‐site programming or clinical staff, residents assigned to IL apartments received a variety of support services from the Department of Mental Health. *Conditionality of tenancy*: no detailed information (probably same as ECH), all study participants paid 30 percent of income for rent and utilities at all placement sites, all are encouraged to participate in community mental health center programs after they were housed, residents are required to maintain behavior that met landlord or co‐resident agreements, tenants paid rent, had some sort of income support. *Housing provision*: care giver provision of housing, Massachusetts Department of Mental Health made 120 units of housing available for the project *Segregation*: probably not segregated housing, one‐ or two single room apartments in public housing subsidized by the Boston Housing Authority. *Case management intensity*: medium, case load 1:15, meetings > once a week.
**Outcomes**	**Stably housed**: housed (broad definition, including “original housing assignment”, “other independent living setting”, “other supported housing setting”), other more narrow alternatives any of these categories separately. **Homeless**: “not housed” (broad definition, including hospitals, jails), “streets or shelters” (narrow definition), or “streets” (most narrow definition). **Other**: Satisfaction with life in general (Lehman QoL), Psychiatric symptoms **Measurement**: the number of days of homelessness for the entire follow‐up period. Number of days of homelessness was recorded for the entire follow‐up period. Study participants’ housing status was identified using their self‐report, records of the housing facilities involved in the research project, records of the Department of Mental Health, and weekly logs completed by the case managers. From these sources, a housing timeline was constructed, indicating the time spent by each study participant in project housing, in other community housing, in shelters, on the streets, and in institutional settings such as hospitals, detoxification centers, or jails, as well as the housing status at the end of the follow‐up period. These data were compiled even for study participants who left project‐sponsored housing or withdrew from active participation in follow‐up research. Data available for intake and 18 months follow up.
Grace 2014 (46)	
**Methods**	**Multi‐site RCT**. N=396 ICM: N=222, US: N=174 Participants recruited between 2005 and 2006.
**Participants**	**Eligibility**: aged 18‐35, in receipt of an allowance, homeless or with a history of homelessness and ‘disadvantaged’ as evidence by eligibility for the personal support program, job placement, employment and training program or intensive support‐customised assistance. **Sample**: Age (mean): Tx= 23.24, Control= 22.92 Gender (% male):65% Ethnicity (Indigenous (%)): Tx= 9%, Control=2% Substance abuse: NR Mental illness: NR Homeless status: NR Criminal (Ex‐offender (%)): Tx=44%; Control=24% **Location**: Cheltenham, Bendigo, Frankston, Inner Melbourne, Australia,
**Interventions**	**Intensive case management** YP4 ‐ Refers to young people and the four key aspects of the trial: purpose (employment), place (accommodation), personal support, and proof (research). YP4's joined up service delivery centred on intensive client‐centred case management, involving direct provision of a range of services as well as the brokering of additional services, all through a single point of contact—the YP4 case manager. J group participants were eligible for joined up services for 18 months to 2.5 years. During this time, there was no time limit, no amount of service limit, and no cessation of eligibility because of success in reaching goals. Thus the intervention was not standardized in terms of duration and intensity. The defining feature was that J group participants remained eligible for joined‐up services, and were entitled to re‐engage with those services at any time during the service delivery phase of the trial. At the end of the service delivery phase of the trial, J group reverted to being eligible for standard services. *Conditionality of tenancy: NA* *Housing provision: NA* *Segregation: NA* *Case management intensity: Number of contacts with case manager over first two years averaged 23 (twice a month). Case manager allocated relatively little time per participant (6‐8 case managers total at any time)* **Usual services** Participants remained eligible for standard services available in the community, including housing, employment, counselling, and health services, but without the joining up and single point of contact that were characteristics of the YP4 joined up services that were available to the intervention group. The mode of service delivery was the key difference between the two groups. Standard service delivery involved clients in complex circumstances receiving multiple and potentially uncoordinated services from different providers. *Conditionality of tenancy: NA* *Housing provision: NA* *Segregation: NA* *Case management intensity: NA*
**Outcomes**	**Housing** (number of moves, housing status at follow‐up, housing stability, homelessness events) **Other:** Employment, community engagement, self‐reported health and well‐being (1 and 2 years) **Measurement**: 12, 24, 36 months
Herman 2011 (56)	
**Methods**	**RCT**. Randomized N=183, Analyzed N=150, CTI (N=77) vs USO (N=73) Participants recruited 2002‐2006
**Participants**	**Eligibility**: 1) currently living in one of the two designated transitional residences following hospitalization during the four‐year recruitment period (2002–2006) and discharged from the residence before the end of this period; 2) a lifetime DSM‐IV diagnosis of a psychotic disorder (codes 295.xx, 296.xx and 298.9); 3) homeless at the index hospitalization or an episode of homelessness within eighteen months preceding this admission; and 4) spent their first night after leaving the transitional residence in New York City in a place other than a jail or a hospital (so that all subjects were at equal risk of homelessness during the observation period and those assigned to the CTI condition would be accessible to the CTI worker). **Sample:** Age (Mean, SD): 37.5 ± 9.5 years Gender: 71% men Ethnicity: 62% African‐American. Mental illness: 61% schizophrenia, 35% schizoaffective disorder. Substance abuse: 53% substance dependence 27% substance abuse without dependence. Homeless status: 79% two or more previous homeless episodes 34% five or more homeless episodes Criminal: NR **Location:** NYC, **USA**
**Interventions**	**Critical Time Intervention (9‐month Case management)** ‐ While living in the transitional residence, all participants received basic discharge planning services and access to psychiatric treatment. After discharge, participants in both conditions received a range of “usual” community‐based services depending on the individual's needs, preferences and living situation. These services usually included various types of case management and clinical treatment. 12 participants (8%) were assigned to mandatory outpatient treatment and/or assertive community treatment programs. ‐ In addition to the services noted above, participants randomly assigned to the experimental condition received nine months of CTI following discharge from the transitional residence. Post‐discharge housing arrangements were typically coordinated by discharge planning staff located at the transitional residence. These arrangements ranged from community residences and other structured programs to supported apartments and independent housing, either alone or with family members. Neither CTI workers nor research staff were involved in determining the initial housing arrangement for individuals in either condition. Some individuals left the transitional residence “against medical advice” and returned to shelters or the streets but were nonetheless retained in the study. ‐ CTI is a nine‐month case management intervention delivered in three phases, each of which lasts approximately three months (see Table 1). o Phase one–transition to the community–focuses on providing intensive support and assessing the resources that exist for the transition of care to community providers. Ideally, the CTI worker will have already begun to engage the client in a working relationship before he or she moves into the community. This is important because the worker will build on this relationship to effectively support the client following discharge from the institution. The CTI worker generally makes detailed arrangements in only the handful of areas seen as most critical for community survival of that individual. o Phase two—try out– is devoted to testing and adjusting the systems of support that were developed during phase one. By now, community providers will have assumed primary responsibility for delivering support and services, and the CTI worker can focus on assessing the degree to which this support system is functioning as planned. In this phase, the worker will intervene only when modification in the system is needed or when a crisis occurs. o Phase three—transfer of care– focuses on completing the transfer of responsibility to community resources that will provide long‐term support. One way in which CTI differs from services typically available during transitional periods is that the transfer of care process is not abrupt; instead, it represents the culmination of work occurring over the full nine months. ‐ CTI was delivered by three workers trained by several of the model developers. Two were bachelors level employees of the NYS Office of Mental Health re‐assigned to this project from their regular duties. The third worker, who also performed some supervisory activities, was a more experienced worker who had previously delivered CTI in an earlier trial. Weekly supervision was carried out by clinically trained staff experienced in the model. *Conditionality of tenancy: NA* *Housing provision: Post‐discharge housing arrangements were typically coordinated by discharge planning staff located at the transitional residence. These arrangements ranged from community residences and other structured programs to supported apartments and independent housing, either alone or with family members. Neither CTI workers nor research staff were involved in determining the initial housing arrangement for individuals in either condition*. *Segregation: NA* *Case‐management intensity: Not reported* **Usual services** ‐ While living in the transitional residence, all participants received basic discharge planning services and access to psychiatric treatment. After discharge, participants in both conditions received a range of “usual” community‐based services depending on the individual's needs, preferences and living situation. These services usually included various types of case management and clinical treatment. 12 participants (8%) were assigned to mandatory outpatient treatment and/or assertive community treatment programs. ‐ Those assigned to the control condition received usual services only. ‐ Neither CTI workers nor research staff were involved in determining the initial housing arrangement for individuals in either condition. *Conditionality of tenancy: NA* *Housing provision: Same as intervention group* *Segregation: NA* *Case‐management intensity: NA*
**Outcomes**	**Homeless**: Homelessness during the last three follow‐up intervals (18 weeks) of the study dichotomous) **Other:** Family relationships, Community integration **Measurement**: 6, 12, 18 months
Hurlburt 1996 (27)	
**Methods**	**Non‐blinded RCT** Randomized N=?, Analyzed N=362, 4 original groups were collapsed into 2, CM+S8 (n_1_=181) vs. CM (n_2_=181),
**Participants**	**Eligibility**: homeless or at severe risk, mentally ill, requirements for Section 8 program **Sample description**: Age (mean): (extrapolated) 37, Range: 18 ‐ <50 Gender (% women): 33.1% Ethnicity (% White): 63% Mental illness (%): schizophrenia 55.4%, bipolar disorder 16.3%, major depression 28.3%, Substance abuse (%): alcohol abuse or dependence: 24%, drug abuse or dependence 20% Homeless status: Less than one year: 32%; More than one year: 67 Criminal: NR **Location**: San Diego, California, USA
**Interventions**	**Case management and Section 8 program (CM+S8)** Comprehensive case management or the traditional level of case management available in San Diego County. Comprehensive case management was provided by a mental health service under contract with the county and differed from the traditional condition in several respects. Comprehensive case managers had a smaller case loads, were constantly available, and had higher salaries. They took a formal team approach to working with clients, attempted to establish housing support groups for participants in housing, and tried to work with clients on finding employment. Section 8 is a federal program allowing certificate holders to pay a fixed 30% of their income for a private rental unit. Section 8 certificates do not require that individuals live in special low‐income housing, but encourages private housing in the community that meets their personal needs and the constraints of their income. Two requirements: housing must meet the quality standards of HUD (the US Department of Housing and Urban Development) and the rent for the unit must be equal or less than fair market rent for the area. Application process tailored to meet needs of mentally ill individuals. Single housing specialists, sensitive to the limitations imposed by severe mental illness, process applications. Structure rules (e.g. keeping appointments) are relaxed. Case managers worked closely with participants as they navigated the Section 8 application process, selected living arrangements, and moved into independent housing. A formal team approach to was taken working with clients, attempted to establish housing support groups for participants in housing, and tried to work with clients on finding employment. *Conditionality of tenancy*: no conditionality except standards rules for ordinary tenants *Housing provision*: apartments provided on the market and Section 8 vouchers used *Segregation*: probably no segregation *Case management intensity*: low, comprehensive CM case load 1:22 and availability 24/7, or standard CM, case load 1:40 **Case management only** Comprehensive case management or the traditional level of case management available in San Diego County. Comprehensive case management was provided by a mental health service under contract with the county and differed from the traditional condition in several respects. Comprehensive case managers had a smaller case loads, were constantly available, and had higher salaries. They took a formal team approach to working with clients, attempted to establish housing support groups for participants in housing, and tried to work with clients on finding employment. *Conditionality of tenancy*: not applicable *Housing provision*: not applicable *Segregation*: not applicable *Case management intensity*: comprehensive CM case load 1:22 and availability 24/7, or standard CM, case load 1:40
**Outcomes**	**Stably housed**: The stable housing category included clients consistently living independently in apartments, and those who had consistent community housing but were not living independently. At least 80% of the days in one of the first three two‐months intervals were spent in independent housing, and at least 80% of the days reported between month 7 and 24 were spent in independent housing or at least 90% of the days reported between the months 19 and 24 were spent in independent housing, and at least 80% of the days in each of the last three two‐month intervals were spent in independent housing. **Homeless:** those neither stably, variably, nor institutionalized reporting data for 12 months or more. *Institutionalized*: Hospital or skilled facility, jail/prison. *Episodically institutionalized*: spent at least 10% of four months intervals between month 7 and month 24 in an institution. *Consistently institutionalized*: at least 90% of days between month 7 and 24 in an institutional setting. *Variably housed*: spent time in some type of housing, and no more one two‐month interval between month 7 and month 24 were more than 10% of time spent in no housing or at least 90% of days between month 7 and 24 were spent in some housing. **Other:** Alcohol use, Drug use **Measurement**: Housing status was assessed over a two‐year period using monthly housing information provided by case managers. Sixty‐day calendar self‐report data in the interviews provided a supplement to case managers’ reports. Criteria for each housing situation included that at least 80% of the days reported between months 7 and 24 were spent in a specific type of housing (e.g., independent housing) or that at least 90% of all reported days between months 19 and 24 were spent in a specific type of housing. Data available at intake and at 24 months follow up.
Kertesz 2007 (58)	
**Methods**	**Single‐blinded RCT** Randomized N=196, Analyzed N=138, ACH (n_1_=63) vs. NAHC (n_2_=66) vs. NH (n_3_=66), Participants were recruited from September 1994 to November 2001
**Participants**	**Eligibility**: homeless, dependent on cocaine **Sample description**: mean age 39 (extrapolated), women 24% The majority of participants identified as African American (91% extrapolated by review authors), male (76%) and had mental illness (75% with Axis I disorder, and 51% with Axis II disorder, extrapolated by review authors). Approximately 14% of participants reported being homeless more than 45 of the 60 days prior to baseline assessments, and 62% reported having criminal convictions during their lifetime. **Location**: Birmingham, Alabama, USA
**Interventions**	**Abstinent‐contingent housing (ACH)** with day treatment ACH groups were charged $161 per month to remain in program housing. Funds could be earned through work therapy but participants were not removed from housing if they failed or were unable to pay. In Phase I, participants received furnished, rent‐free, abstinence‐contingent housing (i.e., a furnished apartment with flatware) after two consecutive drug‐free urine tests. This housing was a treatment intervention (maximum 6 months) and not a permanent housing program. Twice‐weekly urine testing was required of all participants. For abstinence‐contingent housing participants, a positive urine test caused immediate removal from housing and transportation to a shelter; with two consecutive clean urines, the subject could return to program housing. All participants were eligible to seek housing referrals from the host agency or any other agency in the city. The first 6 months of treatment included a combination of day treatment and paid work therapy developed over two previous trials funded by the National Institute on Drug Abuse, provided at BHC under direction of the investigators. This program was divided into phase I (day treatment, months 1‐2), Phase II (work therapy and aftercare, months 3‐6), and an additional 6 months of once‐weekly aftercare group meetings and individual counseling, if desired. Phases I and II were designed to build a nondrug‐use‐based repertoire of activities, rewards, and sources of self‐efficacy. *Conditionality of tenancy*: housing is contingent on sobriety, on leaving urine samples, and on no severe misbehaviour *Housing provision*: no information *Segregation*: no information *Case management intensity*: no information on whether services were organized in the form of case management. **Non‐abstinent‐contingent housing (NACH) with day treatment** NAHC participants received equivalent program as abstinence‐contingent housing participants in a different neighborhood after offering two urine samples, regardless of results. Housing program was similar to abstinence‐contingent housing, non‐abstinence‐contingent housing groups were charged $161 per month to remain in program housing. Funds could be earned through work therapy but participants were not removed from housing if they failed or were unable to pay. In Phase I, non‐abstinence‐contingent housing participants received furnished, rent‐free, abstinence‐contingent housing (i.e., a furnished apartment with flatware). Non‐abstinence‐contingent housing participants remained in housing as long as they provided two urines per week, regardless of result. This housing was a treatment intervention (maximum 6 months) and not a permanent housing program. The first 6 months of treatment included a combination of day treatment and paid work therapy developed over two previous trials funded by the National Institute on Drug Abuse, provided at BHC under direction of the investigators. This program was divided into phase I (day treatment, months 1‐2), Phase II (work therapy and aftercare, months 3‐6), and an additional 6 months of once‐weekly aftercare group meetings and individual counseling, if desired. Phases I and II were designed to build a nondrug‐use‐based repertoire of activities, rewards, and sources of self‐efficacy. *Conditionality of tenancy*: housing is contingent of leaving urine samples, and on no severe misbehaviour *Housing provision*: no information *Segregation*: no information *Case management intensity*: no information on whether services were organized in the form of case management. **Day treatment (no housing)** NH Participants were free to seek their own accommodations while receiving the same outpatient treatment program, and they typically stayed in residential recovery homes or shelters. Participants were eligible to seek housing referrals from the host agency or any other agency in the city. The first 6 months of treatment included a combination of outpatient treatment and paid work therapy developed over two previous trials funded by the National Institute on Drug Abuse, provided at BHC under direction of the investigators. This program was divided into phase I (day treatment, months 1‐2), Phase II (work therapy and aftercare, months 3‐6), and an additional 6 months of once‐weekly aftercare group meetings and individual counseling, if desired. Phases I and II were designed to build a nondrug‐use‐based repertoire of activities, rewards, and sources of self‐efficacy. *Conditionality of tenancy*: not applicable *Housing provision*: not applicable *Segregation*: not applicable *Case management intensity*: no information on whether services were organized in the form of case management.
**Outcomes**	**Stably housed**: days spent in the following settings: own apartment/house, parent/guardian's apartment/house, own single‐resident occupancy (SRO), boarding house, or board and care facility, group home and long‐term alcohol/drug free facility. Settings such as shelter, treatment, or recovery program (including those within shelters), corrections/halfway house, hospital, jail/prison, did not qualify. **Other**: Employment **Measurement**: Principle outcomes were binary indicators of stable housing and stable employment, based on Participants’ responses to a 60‐day recall instrument derived from the widely‐used, reliable Personal History Form, administered at 12 months. Binary outcome extrapolated from percentage of time spent in specific housing situations. Data availbale at intake, 2, 6 and 12 months follow up.
Killaspy 2006 (59)	
**Methods**	**Non‐blinded permutated block randomized assignment (block size=8)** Randomized N=251, Analyzed N 251 (ITT), N=166 (TOT), ACT (n_1_=127) vs. UC (n_1_=124)
**Participants**	**Eligibility**: mental illness, substance misuse, at risk of becoming homeless (implicated) **Sample description**: mean age 39 (extrapolated), women 40%, schizophrenia 53%, schizoaffective 13%, bipolar affective 4%, delusional disorder 3%, major depression 1%, other diagnosis 6%, no data on substance abuse, Details regarding homelessness at baseline was not reported **Location**: London, UK
**Interventions**	**Assertive community treatment (ACT)** Assertive Community Treatment, REACT‐team. No indication of deviations from original model. Contacts with clients in the form of assertive engagement, i.e. multiple attempts, flexible and various approaches (e.g. briefing, offering practical support, leisure activities). Commitment to care in the form of no drop‐out policy, continue to try to engage in long term care. Team approach, all members work with clients, team sources of skills rather than outside agencies, in vivo appointment. *Conditionality of tenancy*: not applicable *Housing provision*: not applicable *Segregation*: not applicable *Case management intensity*: high, case load 1:12 (max), 80‐100 per team, up to daily meeting frequency. **Usual care (UC)** Community mental health teams. Office‐based appointments and/or home visits. Case management like case management style, little “sharing” of work with clients between team members, weekly frequency of team meetings, “brokerage” sources of skills, referral to outside agencies for advice (e.g. social security benefits, housing). *Conditionality of tenancy*: not applicable *Housing provision*: not applicable *Segregation*: not applicable *Case management intensity*: low, 1:35 (max), 300‐350 per team, not information on availability
**Outcomes**	**Stably housed**: not homeless, extrapolated **Homelessness**: not defined, is neither primary nor secondary outcome… “serious incident pas 18 months” **Other**: quality of life, social function, engagement, drug use, alcohol use, arrested/in prison **Measurement**: No details on measurement, homeless engagement acceptance scale, and case notes number of persons homeless past 18 months. Data avialable at intake and 18 months follow up.
Lehman 1997 (60)	
**Methods**	**Non‐blinded RCT** Randomized N=152, Analyzed N=126, ACT (n_1_=77) vs. UC (n_1_=75) Participants recruited 1991‐ 1992
**Participants**	**Eligibility**: homeless, mental illness (if only at risk of becoming homeless, excluded) **Sample description**: mean age 38, women 33%, schizophrenia 45%, schizoaffective disorder 14%, bipolar disorder 20%, depressive disorder 8%, other Axis I disorder 13%, co‐morbid substance use disorder 71%, three quarters of participants had been homeless more than one year **Location**: Baltimore, Maryland, USA
**Interventions**	**Assertive community treatment (ACT) and enhance housing opportunities** The ACT‐team: 12 full‐time equivalent staff, including a program director with a masters’ degree in social work, a full‐time psychiatrist and medical director, 6 clinical case managers (social workers, psychiatric nurses, and rehabilitation counselors), 2 consumer advocates, a secretary receptionist, a part‐time family outreach worker from the alliance for the Mentally Ill of metropolitan Baltimore, and a part‐time nurse practitioner to treat chronic medical problems. Each patient was assigned to a “mini‐team” consisting of a clinical case manager, an attending psychiatrist, and a consumer advocate. The entire ACT team, including the consumer advocates, worked together in decision making and each staff member was acknowledged about most of the patients. Team work was fostered through daily sign‐out rounds and twice‐weekly treatment planning meetings. Enhanced housing opportunities: 40 additional Urban Development Section 8 vouchers were allocated by the city wide mental health authority for the project and were available to the subjects on first‐come first‐served basis. Also, the grant expanded a transitional homeless shelter by 10 beds to provide more access to immediate temporary shelter for the subjects. This shelter program provided case management for the comparison clients. Hence, the experiment occurred in a somewhat enriched housing environment that which existed for other homeless persons in Baltimore. *Conditionality of tenancy*: possibly no conditionality for Section 8 vouchers *Housing provision*: possibly market provision for Section 8 vouchers *Segregation*: possibly non‐segregation for Section 8 vouchers *Case management intensity*: high, case load 1:10, availability 24/7 **Usual care (UC)** Public mental health system encompasses community mental health centers operating under a nonprofit, private, local mental health authority. Several community‐based psychiatric inpatient and emergency facilities, including those affiliated with major teaching institutions, provide acute inpatient and crisis oriented care. A variety of community agencies focus specifically on serving the homeless. Health Care for the Homeless offers outreach, advocacy, case management, primary health care, and walk‐in mental health counseling and some long‐term outpatient mental health care. The goal is to encourage homeless persons to seek health care and to facilitate their transition to mainstream health care services. The provider network for homeless persons also includes several privately run shelters, missions, and soup kitchens. Enhanced housing opportunities: 40 additional Urban Development Section 8 vouchers were allocated by the city wide mental health authority for the project and were available to the subjects on first‐come first‐served basis. Also, the grant expanded a transitional homeless shelter by 10 beds to provide more access to immediate temporary shelter for the subjects. This shelter program provided case management for the comparison clients. Hence, the experiment occurred in a somewhat enriched housing environment that which existed for other homeless persons in Baltimore. *Conditionality of tenancy*: possibly no conditionality for Section 8 vouchers *Housing provision*: possibly market provision for Section 8 vouchers *Segregation*: possibly non‐segregation for Section 8 vouchers *Case management intensity*: a variety is indicated, brokered non‐intensive case management as well as ACT offered to some subjects
**Outcomes**	**Stably housed**: no detailed definition, community housing **Homeless**: no detailed definition, separate data on “homeless on streets” and “homeless in shelters” respectively, but can be collapsed **Other**: Life satisfaction (Quality of life (QOLI)), mental illness symptoms (Colorado Symptom Index CSI), health (Medical outcome study 36‐item short form health survey (SF‐36)), costs **Measurement**: mean days in housing location past year (365 days) after intake, no further information. Binary outcomes extrapolated from continuous outcomes. Data available at intake and 12 months follow up.
Levitt 2013 (62)	
**Methods**	**RCT**. N= 330, ICM+H (N=138) vs CM+H (N=192) Participants recruited 2010‐2012
**Participants**	**Eligibility** families with at least 1 custodial child living in the New York City family shelter system who were certified for Advantage subsidies and who had either (1) at least 2 prior stays in that system in the previous 5 years (episodic) or (2) at least 1 prior stay in that system in the previous 5 years that ended with the family moving into subsidized housing (recidivist). **Sample** Age (mean (SD)): Tx=33.5 (8.7), Control=33.9 (7.2) No other sample characteristics reported **Location:** NYC, USA
**Interventions**	**Home to stay – Intensive case management** ‐ Designed to rapidly obtain and maintain housing for episodic and recidivist homeless families through intensive, temporary support services coupled with a time‐limited housing subsidy. ‐ Partnership between a NYC charity, non profit service providers and NYC government. ‐ Services focused on 3 strategies: o Moving families out of shelter rapidly using a locally funded, temporary housing subsidy o Securing sufficient household income to enable families to pay market rent on expiration of the subsidy o Connecting families to community‐based services that would help them to maintain housing stability after the termination of Home to stay services. ‐ Caseworkers met with client families at homeless shelters to encourage them to voluntarily enroll in Home to Stay services. Each enrolled family was assigned a single caseworker who followed them from shelter into permanent housing to ensure continuity services across that transitional period. ‐ Initial services focused on helping families to secure permanent housing and exit shelter as quickly as possible. ‐ Once client families were placed in housing, services focused on their obtaining a monthly household income equal to at least 200% of the family's rent obligation, obtaining a permanent housing subsidy, or both within 1 year of shelter exit. ‐ Because the availability of permanent subsidies was extremely limited, services primarily focused on maximizing income from public benefits for all eligible household members and obtaining or increasing employment income for all adult household members. ‐ Common elements of the case management model for all service teams included caseloads of 10 to 15 client families per worker, early and aggressive engagement to enroll clients while they were in shelter, flexible scheduling that accommodated clients’ other time demands, individualized service plans informed by an assessment of clients’ needs and strengths and were developed collaboratively with clients, financial literacy services integrated into case management, and frequent contact appropriate to clients’ needs (beginning with at least 4 contacts per months, including at least 1 in situ face‐to‐face contact. ‐ Home to stay clients also remained on the caseloads of their standard services caseworkers and housing specialists. ‐ The program elements differentiating home to stay from standard services were more frequent client contact, smaller caseloads, flexibly scheduling, integrated financial literary services, and continuity of services across the transitional period from shelter into housing. *Conditionality of tenancy: Not reported* *Housing provision: time‐limited housing subsidy. Help securing permanent housing and exiting shelter* *Segregation: NA* *Case management intensity: more frequent client contact, smaller caseloads (10‐15 families per caseworker), flexible scheduling* **Standard services – case management** ‐ Social service staffing and delivery vary widely across the 150 NYC DHS family shelters, but shelter caseworkers are typically assigned mixed caseloads of approximately 25 families. Caseworker qualifications are determined by each shelter provider, and caseworkers receive on‐the‐job training through the provider agency, augmented by specialized DHS‐provided training. Caseworkers are generally supervised by more experience staff, each of whom oversees 4 or more caseworkers; many large shelters also employ a director of social services. ‐ Homeless families with children entering shelter are placed into apartment‐style units for the duration of their stay. ‐ Families are assigned caseworkers who work in collaboration with other city agencies to encourage and assist families in accessing public benefits, pursuing employment, and obtaining supportive services and with shelter housing specialists to locate appropriate, permanent housing. ‐ Caseworkers and families work together to develop detailed plans for exiting shelter and returning to self‐sufficiency. ‐ Caseworkers meet with clients biweekly to review their progress, reassess and address any potential barriers to employment or housing, and make referrals for any required services. Once permanent housing is obtained and a client family moves out of shelter, services from the homeless shelter cease, although community‐based prevention and aftercare are available should the client family require further services *Conditionality of tenancy: Not reported* *Housing provision: Placed in shelter* *Segregation: NA* *Case management intensity:25 families per caseworkermore frequent client contact, smaller caseloads, flexible scheduling*
**Outcomes**	**Stably housed**: Time to exit shelter, total days in shelter, time to return to shelter, prevalence of exiting shelter into subsidized housing **Measurement**: Unclear if case report data or Self report, 12 months
Lipton 1988 (63)	
**Methods**	**Non‐blinded RCT** Randomized N=49, Analyzed N=35, CM+SH (n_1_=26) vs. UC (n_2_=23) Participants recruited 1983.
**Participants**	**Eligibility**: homeless, mental illness **Sample description**: Age (mean): 37 Gender (% women): 35% Ethnicity (% African American): NR Mental illness (%):schizophrenic disorder 82%, affective disorders 2%, personality disorders 8%, other psychotic disorders 8% Substance abuse (%): NR Homeless status (mean duration of most recent homeless episode): 21.1 months Criminal: NR **Location**: NYC, USA
**Interventions**	**Individualized case management and supportive housing (CM+SH)** A non‐profit permanent supportive housing program located in a renovated single‐room occupancy hotel in NYC. Through linkage with city, state and voluntary agencies, the residence provides an integrated and comprehensive array of services to chronic mentally ill patients who are homeless or at risk of becoming homeless. Provided: a furnished room, individualized case management, coordination of public assistance or Social Security benefits, medication monitoring, money management, meals, activity therapy, and, when appropriate, referrals to psychosocial and rehabilitation programs. Through a collaboration with a hospital on‐site psychiatric treatment is provided, and hospital admissions are facilitated when clinically indicated. *Conditionality of tenancy*: no information *Housing provision*: by care givers *Segregation*: yes, category housing *Case management intensity*: no information, probably clinical case management, possibly intensive **Standard post‐discharge care (UC)** Refusal of discharge assistance 26%, shelters 26%, adult home 17%, state hospital 22%, custody of friends 4%, unspecified 4%. *Conditionality of tenancy*: not applicable *Housing provision*: not applicable *Segregation*: not applicable *Case management intensity*: no information
**Outcomes**	**Stably housed**: not homeless (extrapolated) **Homeless**: Sleeping in a public place, such as a street, shelter, or transportation terminal, or in some other location perceived to be temporary by the patient. **Other**: Psychiatric illness severity (SCI) **Measurement**: Measured by questionnaire, number of nights spent in specific locations past 12 months, no further information. 30 or more consecutive nights homeless post discharge. Binary outcome extrapolated from continuous outcomes. Data available at intake, 6 and 12 months follow up.
Marshall 1995 (64)	
**Methods**	**RCT**. Randomized N=80, Analyzed N=80, CM: N=40, C: N=40 Participants recruited in 1991.
**Participants**	**Eligibility**: recruited from shelters/hostels, severe, persistent, psychiatric disorder, were homeless (roofless, or living in a night shelter or hostel for the homeless); at risk of homelessness (ie, facing a threat of eviction or having a recent history of homelessness, or frequent changes of accommodation); living in accommodation which was temporary, or supported (such as a group home), or of poor quality; were coping badly, experiencing social isolation, or causing disturbances; and were not clients of another case‐management service. **Sample description**: Age (mean): 10% 20‐29 years old, 21.8 % 30‐39 years old, 27.7% 40‐49 years old, 17.5% 50‐59 years old, 22.5% over 60 years old (calculated by review authors) Gender (% men): 85% (calculated by review authors) Ethnicity (% African American): Not reported Mental illness: 73.8% schizophrenia and related disorder, 11.3% mood disorders, 6.3% personality disorder, 5% neurotic disorders, 3.8% organic disorders (calculated by review authors) Substance abuse (%): not reported Homeless status: 47.5% in hostels for homeless, 13.8% staffed group homes, 12.5% unstaffed group home, 8.8% night shelter or sleeping rough, 7.5% supported flat, own flat, poor quality bedsit, 1.3% with family (calculated by review authors) Criminal: not reported **Location:** Oxford, UK
**Interventions**	**Case management** Case‐managers chose how much time to offer each subject. As a minimum, each was offered an assessment of need from a case manager, a discussion of the findings of this assessment with the subject's carer, intervention from the case‐manager to meet needs that were identified, monitoring of the subject's progress by the case‐manager, and further assistance should needs arise. In addition, case‐managers were free to choose how far they would personally assist the subject with transport, counselling, organisation of activity programs, assistance with completion of forms, crisis intervention, help with finding accommodation, assistance with benefits, finding work or places on training courses, and help with obtaining furnishings and domestic appliances. The extent to which case‐managers should act as advocates was likewise an individual choice. *Conditionality of tenancy*: NA *Housing provision: NA* *Segregation: NA* *Case management intensity*: varies between clients/case managers – gave a M=21.6 hours (SD=32.4) hours to 36 subjects that completed study **Usual care** Subjects continued to receive any assistance that they had been receiving before the study. Staff working with subjects were at liberty to obtain any further care they saw fit. However, no control subjects were taken on by the study case‐management team, or by any other case‐management team. *Conditionality of tenancy: NA* *Housing provision: NA* *Segregation: NA* *Case management: NA*
**Outcomes**	**Housing status:** days in better/worse accommodation **Other**: Needs for psychiatric and social care – modified version of the MRC Needs for Care Schedule; Quality of life (Self report) – Quality of Life Interview; Employment status (QoL); Psychiatry symptom severity (Manchester scale) Social behaviour (self report) – Social Integration Questionnaire; Social behaviour (observed) – Rehabilitation Evaluation hall and Baker (REHAB) scale **Measurement:** 7, 14 months
McHugo 2004 (65)	** **
**Methods**	**Non‐blinded RCT** Randomized N=125, Analyzed N=121‐102, PHS+ACT (n_1_=60) vs. IHS+ICM (n_2_=61)
**Participants**	**Eligibility**: homeless, severe mental illness **Sample description**: mean age 39, women 52%, schizophrenia 73%, mood disorder 27%, Illicit drug use (past 6 months no days 42%, any days 58%), alcohol use (past 6 months, no days 66%, any days 34%). The majority (85%) of participants were homeless at the start of the study with an average of almost 52 months homelessness during their lifetime. Criminal past was not reported. **Location**: Washington DC, USA
**Interventions**	**Parallel housing services with assertive community treatment (PHS+ACT)** The program is close to the “supported housing model” and was implemented by several multidisciplinary teams, the services were implemented by mobile outreach teams from three mental health agencies that operated in distinct regions in DC, and the program more closely resembled a traditional supported housing approach. ACT is likely to use a shared‐caseload approach. PHS and IHS provided similar case management services. PHS had consistently higher ratings than IHS on team approach, psychiatrist on staff, nurse on staff, and vocational specialist on staff. IHS had higher ratings than PHS on individualized substance abuse treatment and dual‐disorder treatment groups. *Conditionality of tenancy*: continued tenancy is not contingent upon participation in clinical services and there is no live‐in support staff *Housing provision*: the consumer selects the housing from “mainstream” options that are owned and managed by community landlords or housing agencies *Segregation*: housing is integrated within the community; that is, mental health consumers are not segregated in housing *Case management intensity*: high, case load 1:15, availability 24/7 (is indicated) **Integrated housing services with intensive clinical case management (IHS+ICM)** The program is close to the “continuum housing model” and was implemented by several multidisciplinary teams, the services were implemented by five teams within a single provider agency in DC, and the program included aspects of the traditional continuum model. Clinical case management is less likely to use a shared‐caseload approach. PHS and IHS provided similar case management services. PHS had consistently higher ratings than IHS on team approach, psychiatrist on staff, nurse on staff, and vocational specialist on staff. IHS had higher ratings than PHS on individualized substance abuse treatment and dual‐disorder treatment groups. *Conditionality of tenancy*: the mental health provider often links housing with treatment participation and some of the congregate housing units contain live‐in staff *Housing provision*: some of the housing is owned or leased by the mental health provider *Segregation*: housing units are in apartment buildings in which all (or a majority) of the units are occupied by mental health consumers *Case management intensity*: high, case load 1:15, availability 24/7 (is indicated)
**Outcomes**	**Stably housed**: one's own apartment or house, a single room occupancy (SRO; no services), a supportive SRO (services on site), a parent/guardian's apartment or house (long term), another family member's apartment or house (long term), someone else's apartment or house (long term), a boarding house or board‐and‐care facility, a transitional housing program (long term), and a group home. **Homeless**: Functional homelessness: all days of literal homelessness, days in temporary and institutional settings that are preceded by literal homelessness. A person who spent 30 days in a homeless shelter (literal homelessness), then entered a psychiatric hospital for 10 days (institutional setting), and then returned to the streets would be considered to have been functionally homeless for the entire time. A person who lived in an apartment, had a brief hospital stay, and then returned to the apartment would be considered to have spent no time functionally homeless. **Other:** quality of life, psychiatric symptoms, alcohol use, drug use **Measurement**: Time spent in specific residential settings was determined using the Residential Follow‐back Calendar. Respondents reported the number of nights spent in each residential setting in which they had lived during the recall period, as well as the reasons for each move and the household composition. Residential settings were coded into 34 subcategories, and for analysis, they were aggregated into four mutually exclusive and exhaustive categories: literal homelessness, temporary settings, institutional settings, and stable housing. The number of days in each category was then divided by the number of days in the recall period for each participant, thereby converting each category's value (e.g., number of days in stable housing) into a proportion (e.g., proportion of days in stable housing). Data is proportion of days in functional homelessness past 90 days (raw data n:s, m:s and sd:s, binary outcomes are extrapolated). Data available at intake, 6, 12 and 18 months follow up.
Milby 1996 (67)	
**Methods**	**Single‐blind RCT** Randomized N=176, Analyzed N=131, EC (n1=69) vs. UC+12‐step (n2=62) Participants recruited between 1990 and 1991
**Participants**	**Eligibility**: homeless, substance abuse **Sample description**: mean age 36, women 21%, alcohol as primary drug of abuse 24%, cannabis as primary drug of abuse 2%, crack/cocaine as primary drug of abuse 72%, heroine as primary drug of abuse 1%, no information mental illness or criminal activity, approximately 14 months of homelessness before the study (ACH+DT M=13.2 (SD=17.8; US M=14.1 (SD=18.3)). More than a third of participants identified as veterans (35%). **Location**: Birmingham, Alabama, USA
**Interventions**	Enhanced Care (EC) Birmingham model Day treatment, abstinent contingent work therapy, and housing opportunities. Months 1‐2: day treatment. Months 3‐6: day treatment + abstinent contingent work therapy. Month 7‐: clients encouraged, aftercare groups provided by the program or other agencies. Conditionality of tenancy: occupancy of program‐managed housing (and work therapy) was contingent on drug‐free urine toxicologies obtained randomly once a week or on demand Housing provision: housing was provided and managed by the program Segregation: program provided housing appears to be segregated on the level of apartments, but there is no information about the location of houses or building blocks Case management intensity: services are not provided through explicit “case management”, there is no information on case load or other aspects of intensity Usual care (UC) Twice weekly, 12‐Step oriented, individual and group counseling, medical evaluation and treatment and/or referral for identified medical conditions. Referrals for housing and vocational services to other agencies, with counselors serving as case managers. UC was provided indefinitely with no specified end point. Less frequent aftercare visits for continued counseling and support were provided as needed. Education about AIDS and a monthly social support activity. Conditionality of tenancy: probably a variety Housing provision: probably a variety Segregation: probably a variety, other agencies Case management intensity: case managers served as counselors, which indicates some kind of clinical case management, no other information
**Outcomes**	Homeless: Number of days participants report being homeles sin the 60 days prior to the 12 month follow‐up interview. Measurement: interviews based on personal history form (PHF) concerning past days homeless previous 60 days. Test‐retest reliability was assessed with ICC above 0.60. Follow‐up points were 2‐months ±30 days, 6‐months ±50 days, and 12‐months ±70 days. Figures estimated graphically. Data available at intake, 2, 6 and 12 months follow up.
Milby 2003 (66)	
**Methods**	**Single‐blind RCT** Baseline N=141 (110+31), Analyzed N=100, DT+ (n_1_=72) vs. DT (n_2_=69) Participants recruited between April 1995 and May 1996
**Participants**	**Eligibility** homeless, cocaine abuse or dependence, non‐psychotic mental disorder **Sample description** mean age 38, women 28%, alcohol (prim. abuse) 17%, cannabis (prim. abuse) 1%, Cocaine (prim. abuse) 81%, opiates (prim. abuse) 2%. No information n mental illness. **Location** Birmingham, Alabama, USA
**Interventions**	**Day treatment with abstinent contingent housing and work therapy (DT+)***Months 1‐2*: participant governed morning meetings, process groups, AIDS education, relapse prevention training, goal development, goal review, assertiveness training, role play, weekend planning, reinforcement exposure and planning, recreation outing group, 12 steps, relaxation, recreation goal development and review, individual counseling, psychological evaluation, and urine monitoring (twice weekly). Formulation of housing and employment goals. After 2 consecutive weeks of abstinence ‐ immediate move to program provided rent free furnished apartment or unit in group home. *Months 3‐6*: after care (group therapy utilizing goals and psycho‐education content from phase 1), abstinent contingent work therapy, 50% of clients remained in original housing (from phase 1) and 50% moved to program managed individual houses. *Conditionality of tenancy*: abstinent contingent housing *Housing provision*: housing provided by care giver *Segregation*: yes four group houses and one 12 room apartment *Case management intensity*: not applicable **Day treatment only (DT)***Months 1‐2*: participant governed morning meetings, process groups, AIDS education, relapse prevention training, goal development, goal review, assertiveness training, role play, weekend planning, reinforcement exposure and planning, recreation outing group, 12 steps, relaxation, recreation goal development and review, individual counseling, psychological evaluation, and urine monitoring (twice weekly). *Months 3‐6*: after care (group therapy utilizing goals and psycho‐education content from phase 1). *Conditionality of tenancy*: not applicable *Housing provision*: not applicable *Segregation*: not applicable *Case management intensity*: not applicable
**Outcomes**	**Stably housed** Not homeless (possibly), no statistics on homelessness, binary outcomes based on extrapolations from continuous outcomes and problematic baseline data. **Homeless** Someone who lacks a fixed regular, and adequate nighttime residence, including: those whose primary nighttime residence are shelters or other temporary accommodations; public or private places not designed for or ordinarily used as, a regular sleeping accommodation for human beings; or someone at immediate risk of becoming homeless. (McKinney Act Criteria) **Measurement** …by sections of the retrospective interview for housing, employment, and treatment history (RHETRO) derived from the personal history form (PHF) with well documented psychometric properties. Binary data extrapolated from continuous data. Data available intake and 12 months follow up.
**Notes**	No case management comparisons
Milby 2010 (68)	
**Methods**	Non‐blinded RCT. N=206, Tx (n_1_=103) vs Control (n_2_=103) Participants were recruited between 2001 and 2004
**Participants**	Eligibility: McKinney act criteria incl. homeless, cocaine dependence, psychological distress and willingness to participate and no plans to move for 18 months Sample: The majority of participants identified as African American (93‐96%). Participants struggled with alcohol (10%), cannabis (9‐10%) and cocaine (6‐7%). Details on mental illness, homeless status and criminal background were not reported Location: Birmingham, Alabama, USA.
**Interventions**	Contingency management +: Participants were immediately provided with a furnished and rent free apartment which was contingent on continued sobriety during phase I (weeks 2‐8). Urine tests were carried out regularly and within six hours of a positive test participants were moved to shelter and could only return to their apartment after three consecutive negative urine tests. Participants began receiving vocational training immediately (four days a week for 3.5 hours per day). In Phase II (weeks 3‐24) participants were required to pay a small amount of rent (not specified) from program provided stipends. Participants who maintained abstinence were moved to a transitional housing program funded by the national housing department (HUD). In Phase III continued tenancy in abstinence‐contingent program housing was only available when space was available at a modest rent. Contingency management: Control group participants received the same abstinence‐contingent housing, vocational training and work therapy as participants in the intervention group, but were not offered day treatment based on the community reinforcement approach.
**Outcomes**	Stable housing: Proportion of participants housed more than 40 of the previous 60 days Employment ‐ Proportion of participants employed more than 40 of past 60 days Abstinence ‐ mean number of drinking days per week Outcomes measured at 6, 12, 18 month
Morse 1992 (39)	
**Methods**	**Non‐blinded RCT** Randomized N=177, Analyzed N=103, ACT (n_1_=52) vs. DI (n_2_=62) vs. OC (n_3_=63) Participants recruited 1988‐1989
**Participants**	**Eligibility**: Homeless, mentally ill **Sample description**: mean age 34, woman 42%, schizophrenic disorder 30%, major depression 21%, bipolar disorder 8%, other psychotic disorder 5%, not listed diagnosis (axis I) 15%alcohol abuse 12%, other drug abuse 4%, no diagnosis 5%, axis I & substance abuse 23%, participants reported that it had been approximately 17 months since they last lived at a stable address, Criminal past: NR **Location**: St Louis, Missouri, USA
**Interventions**	**Assertive community treatment (ACT)** Adapted to meet special problems associated with homelessness by conducting outreach to shelters to engage reluctant and suspicious clients and prioritizing client needs for basic survival and permanent housing. Program emphasized client advocacy, client factors, contribute to low levels of service to homeless, encouraging individual change, helping clients to form ongoing therapeutic relationship, to learn better ways to cope with problems, linking with psychiatric medication services, teaching community living skills and interpersonal skills, and providing crisis intervention. Environmental change through casework advocacy to obtain resources from agencies addressing client's welfare, housing, and health needs. Staff intervened with persons in the client's environment, such as landlords or shelter providers, to encourage more positive reactions to clients. Support included e.g. providing transportation, medication management, money management, and payee service, and ongoing assistance in keeping apartments clean. *Conditionality of tenancy*: not applicable, yet involuntary treatment *Housing provision*: not applicable, “non‐reject” policy no other information *Segregation*: not applicable, “community living” no other information *Case management intensity*: high, case load 1:10, provision of community‐based services for an unlimited time **Daytime drop (DI) in centers for homeless mentally ill persons** One center exclusively for women, another open to men and women (vast majority of its clients were men). Centers provided homeless people respite from life on the street during the daytime, when the emergency shelters were closed, and offered food, clothing, showers, and some recreational opportunities such as card playing. *Conditionality of tenancy*: not applicable *Housing provision*: not applicable *Segregation*: not applicable *Case management intensity*: low, case load 1:40 **Traditional outpatient clinic (OC)** Treatment provided as a mental health clinic. Program offered psycho therapy, psychiatric medication, and assistance in obtaining social services. *Conditionality of tenancy*: not applicable *Housing provision*: not applicable *Segregation*: not applicable *Case management intensity*: no information
**Outcomes**	**Stably Housed**: extrapolated (not homeless) **Homeless**: literary homeless (emergency shelters, parks, bus depots, other public places). **Other**: employment (monthly income), Psychiatric symptoms (Brief Symptom Inventory), Alienation, interpersonal adjustment (Personality and Social Network Adjustment Scale (Clark 1968) – four items re. how get along with same and opposite sex, family and others in general)), Self‐esteem – Rosenberg self‐esteem scale (short form), Alcohol abuse (ounces consumed per week),Professional and natural support networks **Measurement**: interviews on homeless nights past month (30 days), no further information. Binary outcomes extrapolated from continuous outcomes. Data available at intake and 12 months follow up.
Morse 1997 (40)	
**Methods**	**Non‐blinded RCT** Randomized N=135, Analyzed N=135 Participants recruited 1990‐1993
**Participants**	**Eligibility**: homeless, at risk, mental illness **Sample description**: mean age 35, women 42%, schizophrenia 66%, recurrent depression 15%, bipolar disorder 13%, atypical psychosis 12%, delusional or paranoid disorder 3%, dementia 1%, no information on alcohol/drug abuse, Criminal: NR **Location**: St Louis, Missouri, USA
**Interventions**	**Assertive community treatment (ACT)** Program included intensive individualized treatment: homeless outreach, engagement methods, assisting with basic needs, developing service plans following priorities stated by each client, individual treatment activities, building therapeutic alliance, linking clients with medication services, helping clients cope with symptoms and solve practical problems in daily living, teaching them community skills, support in obtaining and maintaining housing, monitoring medications, providing payee and money management services, and assisting with transportation. Teams: 5‐7 persons, backgrounds in psychology, social work, and counseling. *Conditionality of tenancy*: not applicable *Housing provision*: not applicable *Segregation*: not applicable *Case management intensity*: high, case load 1:10, no psychiatric nurse on staff, psychiatrist available only 2 h/week. Most medication services obtained through linkage with private or clinic‐based psychiatrists, no information on availability **Assertive community treatment with community workers (ACT‐CW)** Similar to ACT above, but clients also assigned to paraprofessional community workers. CW‐role: assist with activities of daily living, be available for leisure activities. CW spent more time with the client in the latter phases of treatment, after initial stabilization. *Conditionality of tenancy*: not applicable *Housing provision*: not applicable *Segregation*: not applicable *Case management intensity*: high, case load 1:10, no psychiatric nurse on staff, psychiatrist available only 2 h/week. Most medication services obtained through linkage with private or clinic‐based psychiatrists, no information on availability **Brokered case management condition (BCM)** BCM role: develop an individualized service plan for the client, arrange for and purchase mental health and psychosocial services from various service providers, monitor the quality of purchased services, and adjust the mix of services based on the client's changing needs. BCM: more office based than CMs on the assertive community treatment team. BCMs rarely went into emergency shelters, made home visits, or accompanied their clients to other agencies and potential housing sites. *Conditionality of tenancy*: not applicable *Housing provision*: not applicable *Segregation*: not applicable *Case management intensity*: low, case load 1:85, no information on availability
**Outcomes**	**Stably housed**: staying in a boarding home, public housing, or their own apartment. **Homeless**: no definition (not stably housed) **Other**: employment (monthly income), mental health (Brief psychiatric rate scale), Substance abuse **Measurement**: clients reported how many days they were literally homeless, precariously housed, and stably housed. Outcome variable: mean number of days in stable housing per month. Data mostly extracted from Wolff 1997. Binary outcomes are extrapolated from continuous outcomes. Data available at intake and 18 months follow up.
Morse 2006 (69)	
**Methods**	**Non‐blinded RCT** Randomized N=196, Analyzed N=149, IACT (n_1_=46) vs. ACT (n_2_=54), UC (n_3_=49) Participants recruited 1998‐2003
**Participants**	**Eligibility**: literally homeless, severe mental illness, substance use disorder **Sample description**: mean age 40, women 20%, schizophrenia 48%, schizoaffective disorder 19%, atypical psychotic disorder 11%, bipolar disorder 11%, major depression‐recurrent disorder 9%, delusional disorder 2%, substance dependence disorder (alcohol and/or drugs) 46%, substance abuse disorder (alcohol and/or drugs) 64%, alcohol only disorder 40%, drug only diagnosis 18%, both drug and alcohol disorder 42%, cocaine or crack 34%, cannabis 19%, participants reported a mean of 13 days homeless in the month previous to baseline **Location**: St Louis, Missouri, USA
**Interventions**	**Integrated treatment and Assertive Community Treatment (IACT)** Key components: (a) assertive outreach, which is needed to engage many dual disorder individuals into treatment; (b) motivational interventions, which are needed to gradually help individuals who are not committed to abstinence to develop personal goals for substance abuse recovery; (c) a stages‐of‐treatment approach, which includes the following phases: engagement, persuasion, active treatment, relapse prevention; (d) cognitive behavioral counseling, which helps people develop skills for an abstinent life style; and (e) interventions to strengthen social networks supportive of recovery. Interventions take a long term, culturally competent and comprehensive perspective, and can be combined with various types of mental health services, such as residential programs, and assertive community treatment. The IACT team had a substance abuse specialist on staff and provided substance abuse services directly as a part of the ACT team. These services included individual substance abuse counseling and bi‐weekly treatment groups. *Conditionality of tenancy*: not applicable *Housing provision*: not applicable *Segregation*: not applicable *Case management intensity*: probably high, no detailed information **Assertive community treatment only (ACT)** One agency had a psychosocial rehabilitation day treatment on site which was also used by some of the participants. The other agency operated its transitional housing facility which was used by some of the participants. Team received training and follow‐up consultation from project personnel regarding ACT treatment principles and practices. The ACT team was instructed to refer clients to other community providers for outpatient or individual substance abuse services and to 12‐steps groups. *Conditionality of tenancy*: not applicable *Housing provision*: not applicable *Segregation*: not applicable *Case management intensity*: probably high, no detailed information **Standard care control (UC)** Participants were shown a list of community agencies that provided mental health and substance abuse treatment. Research staff also provided them with current information about openings at the various agencies and provided linkage assistance to help them access services at the agencies. *Conditionality of tenancy*: not applicable *Housing provision*: not applicable *Segregation*: not applicable *Case management intensity*: probably low, no detailed information
**Outcomes**	**Stably housed**: own apartment or boarding home **Homeless**: extrapolated, not stably housed **Other:** Criminal ‐ Major and minor offences **Measuremen** t: Data were obtained from service agencies, claim records, and participants self‐report. Number of days in stable housing last month. Binary outcomes extrapolated from continuous outcomes. Data available at intake, 6, 12, 18 and 24 months follow up.
Nordentoft 2010 (70)	
**Methods**	**Multi‐site RCT**. ACT (N=275) vs ST (N=272) vs HBR (N=31) Participants recruited 1998‐2000
**Participants**	**Eligibility**: Age 18–45 years; clinical diagnoses of schizophrenia, schizotypal disorder, persistent delusional disorder, acute and transient, psychotic disorder, schizoaffective disorder, induced delusional disorder, or unspecified non‐organic psychosis according to ICD–10 research criteria, based on Schedules for Clinical Assessment in Neuropsychiatry, SCAN version 2.0 and 2.1; no antipsychotic medication exceeding 12 weeks of continuous medication; absence of mental retardation and organic mental disorder; no psychotic condition solely due to acute intoxication or a withdrawal state; and written informed consent. **Sample:** Age (Mean(SD)): Tx= 26.6(6.4), Control= 26.6 (6.3) Gender (% male): Tx= 58%, Control= 60% Ethnicity: NR Substance abuse: 27% per group with dependence to any psychoactive substance Mental illness (ICD diagnosis schizophrenia) (%): Tx= 67%, Control= 65% Homeless status (%): Tx= 5%, Control= 4% Criminal: NR
**Interventions**	**Assertive Community treatment +** Specialized assertive intervention (OPUS) was modelled on elements described by Stein & Test (1980) and consisted of : (1) Assertive community treatment. Two teams were established in Copenhagen, each with one senior psychiatrist, one psychologist, one or two nurses, one occupational therapist, one social worker and a vocational/educational guide (who served in both teams). The caseload did not exceed 10 patients per case manager. One primary person was responsible for maintaining contact, coordinating treatment and treatment adherence. The patients were also visited weekly when hospitalized. During admission, however, treatment responsibility was transferred to the hospital. These teams treated patients allocated to OPUS in the two‐armed and three‐armed randomization. The average number of patients in the teams was 60. (2) Medication. As in hospital‐based rehabilitation. (3) Psycho‐educational family treatment. As in hospital‐based rehabilitation, but the multifamily groups continued for 1.5 years with approximately 40 sessions. The therapists were externally supervised. (4) Social skills training was inspired by the model described by Liberman et al. (1986). Patients with impaired skills were offered training in groups with a maximum of six participants. There were two therapists, one of whom was a psychologist. The training consisted of modules: medication self‐management; coping with symptoms; conversational skills; problem solving; conflict management. Patients who did not need training received individual psycho‐education from the primary staff member. (5) Psychological treatment. If needed, the patients were offered supportive or cognitive therapy. The reliability of the program has been described elsewhere and was measured with the Index of Fidelity of Assertive Community Treatment (McGrew et al. 1994), which was 70%. The reduced fidelity was due to the lack of 24‐h coverage and approximately, weekly face‐to‐face meetings between staff member and patient. *Conditionality of tenancy: NR* *Housing provision: NA* *Segregation: NR* *Case management intensity: Caseload 1:10. Primary team member designated for each patient. Office hours 8‐17 mon‐Fri but all case team workers available cell for messages. Visited at home or in hospital when necessary* **Standard services** Most patients were offered treatment at a community mental health centre after discharge. They were usually seen in the office, each patient being in contact with a physician, a community mental health nurse and a social worker. The caseload of the staff in the community mental health centres varied between 1:20 and 1: 30. Standard treatment consisted of the following elements: (1) Admission. Decisions on the need for hospitalization or out‐patient treatment were made as usual. Patients in standard treatment and OPUS patients were admitted to the same psychiatric departments as patients not included in the trial. The patients in standard treatment did not receive the experimental interventions. Patients in standard treatment seldom met the therapists from the local community mental health centre before they were discharged to follow‐up treatment at the centre. (2) Medication. As in hospital‐based rehabilitation. (3) Psycho‐educational family treatment. A minor proportion of the patients were offered supportive contacts with members of their families or educational groups for relatives. (4) Social rehabilitation. Supportive counselling, psycho‐ education, vocational guidance and training in daily living activities were offered sporadically. (5) Psychological treatment. This was not offered systematically. *Conditionality of tenancy: NR* *Housing provision: NA* *Segregation: NR* *Case management intensity: Caseloads 1:20 – 1:30. Contacts at treatment centre*.
**Outcomes**	**Stably housed**: NR **Homeless**: Homelessness **Other**: mental health, substance abuse, employment/education, death, Supported housing **Measurement**: 1 and 5 year
Nyamathi 2015 (83)	** **
**Methods**	RCT: Randomized N=600, Analyzed N=600, PCNCM: N=195, PC: N=196, UC: N=209 Participants recruited 2010‐2013
**Participants**	**Eligibility**: Ex‐offenders with a history of drug use prior to their most recent incarceration, 18‐60 years old, participating in residential drug treatment program, considered to be homeless before existing prison. Exclusion criteria: not speaking English, or considered by research staff to be cognitively impaired. **Sample**: Age (mean (SD)): 40 (10.4) Gender (% male): 100% Ethnicity (% African American): 46% Mental illness (%): Substance abuse: Ever used stimulants: 84%, ever used heroin: 36.4%, ever used marijuana: 85.7% Homeless status: 100% homeless, 64% in street/shelter/ someone else's house Criminal: Recruited from jail: 44.8%, recruited from prison: 55.2% **Location:** Los Angeles, California, USA
**Interventions**	**Intensive peer coach and nurse case managed (PC‐NCM) program:** Each participant received 45 min per week with a peer coach with a focus on building effective coping skills, personal assertiveness, self‐management, therapeutic non‐violent communication (NVC), and self‐esteem and avoiding health‐risk behaviors, increasing access to medical and psychiatric treatment and improving compliance with medications, skill‐building, and personal empowerment. THere were also discussions on strategies to assist in seeking support and assistance from community agencies after leaving the residential drug treatment program. These sessions continued by phone after participants left residential part of treatment. Peer coaches were trained in delivering eight sessions on non‐violent communication. One nurse was trained by an expert in nurse case management, hepatitis infection and transmission, and barriers that impede HAV/HBV vaccination among vulnerable populations. The program‐specific nurse provided 20 minutes per participant of culturally competent NCM per week over 8 weeks which focused on health promotion, completion of drug treatment, vaccination compliance, and reduction of risky drug and sexual behaviors. A peer coach spent 45 min on a weekly basis with each assigned participant plus nurse spent 20 mins per week with each participant Conditionality of tenancy: NA Housing provision: No Segregation: NA Case management intensity: approximately 65 mins per participant per week. **Intermediate peer coaching (PC) program with brief nurse counselling: W** eekly peer coaching without the nurse case management component. A nurse provided a brief, 20‐min education session on hepatitis prevention and HIV risk reduction *Conditionality of tenancy: NA* *Housing provision: No* *Segregation: NA* *Case management intensity: weekly peer coaching, and one session on hep*. **Usual services: P** articipants were encouraged to complete a three series HAV/HBV vaccine (same as other intervention), plus a brief, 20‐min session from a peer coach with training in basic health promotion. Participants received all recovery and rehabilitation services available at the site, including substance abuse services, assistance with independent living skills, job skills assistance, literacy, various counseling services, and discharge planning. The only differences were the absence of the two configurations of peer coaching and/or nurse‐led case management. *Conditionality of tenancy: NA* *Housing provision: No* *Segregation: NA* *Case management intensity: one 20 min session from a peer coach*.
**Outcomes**	**Homelessness** **Other**: Substance use, General health, Rearrest and reincarceration **Measurement**: 12 months
Rosenheck 2003 (71)	
**Methods**	**Non‐blinded RCT** Randomized N=460, Analyzed N=331‐245, HUD‐VASH (n_1_=182) vs. ICM (n_2_=90) vs. VA‐UC (n_3_=188) Participants recruited 1992‐1995
**Participants**	**Eligibility**: homeless veterans; major psychiatric disorder and/or alcohol/drug abuse disorder **Sample description:** mean age 42, women 4%, serious psychiatric diagnoses 10%, dual diagnoses 35%, other psychiatric disorders 5%, likelihood of past hospitalization for drug abuse 50%, alcohol or drug disorders 50%, participants reported being homeless one third of the 90 days prior to baseline, Criminal ‐ NR **Location**: multisite, four VA medical centers San Francisco & San Diego, California, New Orleans, Louisiana, Cleveland, Ohio, USA
**Interventions**	**Department of Housing and Urban Development (HUD) and US Department of Veterans Affairs (VA) Supported housing program (HUD‐VASH)** Through an interagency agreement, HUD allocated funds for approximately 1000 housing vouchers for a program providing housing and case management assistance for literally homeless veterans with psychiatric or substance abuse problems or both. The essential feature of the program is that participants were offered priority access to Section 8 housing vouchers, authorize payment of a standardized local fair market rent which is less than 30 % of the individual beneficiary´s income. Case managers united veterans with the local housing voucher and helped them to; 1) locate an apartment, 2) negotiate the lease and 3) furnish and move into the apartment. The majority of the case managers were social workers and nurses who encouraged counseling regarding substance abuse and employment. *Conditionality of tenancy*: each veteran had to agree to a treatment plan involving further participation in case management and other specified services if randomized either HUD‐VASH or case management only *Housing provision*: Section 8 vouchers, housing not provided by care giver *Segregation*: no information, probably not segregated *Case management intensity*: unclear, CM‐model modified from ACT‐model, encourages weekly face‐to‐face contract, community‐based service delivery and more intensive involvement in situations of crisis. No further details. **Intensive Case management only (ICM)** Case managers united veterans with the local housing voucher and helped them to; 1) locate an apartment, 2) negotiate the lease and 3) furnish and move into the apartment. The majority of the case managers were social workers and nurses who encouraged counseling regarding substance abuse and employment. Case managers were to provide the same intensity of services as in the HUD‐VASH condition and were encouraged to use whatever housing resources they could obtain for the veterans. *Conditionality of tenancy*: not applicable *Housing provision*: not applicable *Segregation*: not applicable *Case management intensity*: unclear, CM‐model modified from ACT‐model, encourages weekly face‐to‐face contract, community‐based service delivery and more intensive involvement in situations of crisis. No further details. **Standard VA homeless services (VA‐UC)** Short‐term broker case management as provided by HCHV program (Health Care for Homeless Veterans) outreach workers. *Conditionality of tenancy*: not applicable *Housing provision*: not applicable *Segregation*: not applicable *Case management intensity*: low, broker case management
**Outcomes**	**Stably housed**: an apartment, room, or house of one's own or of a family member or a friend. **Homeless**: an emergency shelter, a substandard single room occupancy hotel, outdoors on the sidewalk, or in a park, abandoned building, automobile, truck, or boat. **Other**: Alcohol use (alcohol index score), Drug use (Drug index score), Mental health, (Psychiatric index score), Physical health (medical index score), Employment (employment index score), Arrests (no.),Brief Symptoms Inventory for psychological distress, Lehman Quality of life Interview subscales (quality of life, satisfaction with current housing, family relationships, social relationships, health care, finances), Social support (avg no. ppl who would help with loan or transport in emotional crisis, no ppl in nine different categories to whom the veteran reported feeling close, index of total frequency of contact with these people) **Measurement**: baseline and follow‐up assessments interviews every 3 months were conducted by trained evaluation assistants. The number of days sleeping in specific place. Data were obtained in the duration of the current episode of homelessness, the housing status during the 90 days before each interview. Binary outcomes extrapolated from continuous outcomes. Data available at intake, 6, 12, 18, 24 and 36 months follow up.
Samuels 2015 (72)	
**Methods**	RCT. Randomized N=223, Analyzed N=210, FCTI: N=97, US: N=113 Participants recruited 2001‐2004
**Participants**	**Eligibility**: Single, female‐headed households entering family homeless shelters. Mothers met criteria for an Axis 1 diagnosis of mental illness and/or substance abuse problem sometime in the year prior to entry in the shelter system. At least one child between 18 mos and 16 years living with them. Families entering shelters for domestic violence were excluded, but mothers with histories of domestic violence were included. **Sample:** Age (mean (SD)): 32.5 (7.8) Gender (% female) 100%: Ethnicity (% African American): 56% Mental illness (GSI mean score (SD)): 57.7 (12) Substance abuse: NR Homeless status: 100% come from shelters Criminal: NR Other: Number of children (mean (SD)): 3.0 (1.6); Mean age of children (SD): 9 (5); Currently employed (%): 15% **Location:** New York, USA
**Interventions**	**Family Critical time intervention**‐ Community based care management in three phases of 3 months each (a) Transition to community; (b) Try‐out; (c) Transfer to care ‐ Designed to strengthen family members’ long‐term ties to the services they need, heal and strengthen maternal relationships with extended families and friends, and provide emotional and practical support during the critical time of transition from homelessness to stable housing in the community. FCTI focuses on the relationship between the case manager and mother that progresses through the 9 month period. ‐ 3 primary differences between FCTI and services as usual: (1) Intervention group received continuous case management services from a single worker with specific training in the CTI model (2) FCTI caseloads were12 or less families per care manager while usual services workers had 50 or more families; (3) Substantially lower threshold for housing readiness for the intervention group than for the control group. *Conditionality of tenancy: No abstinent contingent requirements or engagement in mental health services typically required of usual services clients*. *Housing provision: Families provided with scattered site housing without time limits without having to meet the housing readiness requirements typically imposed on usual services clients* *Segregation: No* *Case management intensity: FCTI caseloads <12 families per case manager. Continuous case management from a single worker*. **Housing and homeless services as usual** ‐ All families entered the country homeless shelter system that provided for the placement of homeless families, singles, and childless couples in shelter facilities, transitional residences, and emergency housing. The system has been considered service‐rich and well‐coordinated; housing and homeless services represented one program in an array of socials services provided through the country to address the needs of low‐income households, including employment services, child support services, family and children's services, medical/home care services, and temporary financial services. In 2004, housing and homeless services also began administering homelessness prevention programs, including a rental assistance program. ‐ Upon entry into the shelter system, families received a comprehensive assessment of needs over a 2 week period while staying in a 100 room former hotel. Parents and children were screened for problems in the areas of medical, mental health, substance abuse, and education. Clinical and nonclinical interviews explored families’ pathways to homelessness, housing history, income and employment, education, and challenges faced by families. Each family received an independent living plan with treatment and service recommendations as deemed necessary by shelter staff. Typically, these plans included personal goal setting, communication, housekeeping and parenting skills, and referrals for any needed treatment. In addition, county social services staff and outside agency representatives provided full‐time and part‐time, onsite and offsite services to homeless households through contractual affiliations with and referrals to county nonprofit and private service providers. ‐ Families remained at the assessment center an average of 30 to 45 days while waiting for referral to their next placement in the shelter system. Referrals were made to 1 of 4 other shelters managed by nonprofit agencies. Sites varied in size (25‐100 families) and living arrangements (converted hotel, new buildings with kitchen, apartment buildings). Sparsely furnished, relatively overcrowded and lacked privacy. Shelter sites typically provided basic on‐site services that included, but were not limited to physical and mental health assessment and treatment; case management, substance abuse screening and rehabilitations; childcare, recreation and after school programs, parenting, adult education, life skills and job readiness programs; and home‐finding program. Shelter personnel provided many of the onsite services. ‐ Stay durations ranged from a few months to more than 2 years. IF families were not able to move out with the use of personal resources, they stayed until they were evaluated by shelter staff as being housing ready (capable of finding and maintaining a permanent dwelling). Families then moved to transitional apartments designed as a step between living in a shelter and obtaining permanent housing. Transitional housing was provided with case management paid through a per diem rate that varied by provider contract and family size. To remain eligible for housing, families needed to work toward achieving housing readiness goals in specific areas, as designated in their independent living plans. Services provided often included counseling, treatment, services for specific health and mental health issues, and assistance with obtaining and maintaining permanent housing. ‐ Access to subsidized housing was difficult. *Conditionality of tenancy: NA* *Housing provision: in a shelter system* *Segregation: NA* *Case management intensity: services workers had 50 or more families*
**Outcomes**	**Stable housing** (Residential follow‐back instrument) – number of days since baseline until families moved out of a homeless shelter **Proportion of time homeless** – divided number of days spent in homeless shelter after baseline by total number of days since baseline **Other:** Maternal mental health (Brief Symptom Inventory, Global Severity Index) **Measurement:** 3, 9, 15 months
Shern 2000 (73)	
**Methods**	**Non‐blinded RCT** Randomized N=168, Analyzed N=?, CHS+ICM (n_1_=91) vs. UC (n_2_=77)
**Participants**	**Eligibility**: homeless, severe mental illness (not exclusively chemical abuse or mental retardation) **Sample description**: Age (mean): 39.97 (21‐66) Gender (% women): 24% Ethnicity (% African American): 61% Mental illness: major mental illness diagnosis 91% (our calculation), Substance abuse (%):lifetime alcohol/substance abuse disorder diagnosis 54%, dual‐diagnosis 47% Homeless status:48% reported more than 1 episode of homelessness, 61% of remaining reported being homeless more than 4 years Criminal: NR **Location**: NYC, USA
**Interventions**	**Choices with ICM (CHS+ICM)** Emphasis on individual choice, continuity in relationships, skills development, and support to foster achievement of personal goals. Features: (1) outreach and engagement, staff‐client relationship development; (2) invitation to low‐demand environment with resources (showers, food) 7am‐7pm, structured group activities possible but not required, available assistance in obtaining health, mental health, dental, and social services and in developing and implementing individual rehabilitation plans, socializing opportunities; (3) respite housing in 10‐bed shelters or rooms in blocks rented by program and overseen by staff; (4) in‐community and on‐site rehabilitation services to assist individuals in finding and maintaining community‐based housing. Choices was staffed by 6 rehabilitation specialists (who received extensive training and ongoing supervision from Boston University) and respite staff (oversaw respite housing and operated the center weekends/holidays). Many respite staff were former homeless and in recovery from alcohol or substance abuse; a psychiatrist (weekly informal consultations), a public health nurse (8 hours per week) *Conditionality of tenancy*: low level of conditionality, emphasis on consumers choice, no further information *Housing provision*: partly care provided housing, 2700 units of specialty housing for persons with mental illness were developed through a joint city/state program, choices first developed relations with the supported apartment program and then initiated an own supported apartment program. *Segregation*: partly, housing varied from structured community residences to independent apartments *Case management intensity*: high, case load 1:13, no information on availability (probably 24/7). **Usual care (UC)** UC: structurally segmented and transitionally oriented, engagement with multiple programs and caregivers to negotiate a pathway out of homelessness. UC included a range of programs for homeless and specialty programs for homeless with mental illness, including outreach services, drop‐in centers, case management programs, mental health and health services, soup kitchens, municipal and private shelters, and specialized municipal shelters for persons with psychiatric disabilities. *Conditionality of tenancy*: yes, a strong normative orientation in which set pathways in and out of services are prescribed and adherence to behavioral norms are mandated for successfully obtaining and maintaining housing (e.g., remaining sober as prerequisite for entry into a community reintegration program) *Housing provision*: partly care provided housing, 2700 units of specialty housing for persons with mental illness were developed through a joint city/state program. *Segregation*: partly, housing varied from structured community residences to independent apartments *Case management intensity*: no information
**Outcomes**	**Stably housed**: no detailed definitions of housing status, two categories: (1) community living, and (2) institutions. **Homeless**: no detailed definitions of housing status, two categories: (1) streets, and (2) shelters. **Other:** Quality if life (Lehman's Quality of Life Scale ‐ overall), Psychological status (Colorado Symptom Index) **Measurement**: housing status (change in proportion of time spent in residential setting). Face‐to‐face interview protocols were used. Interviewers attempted to contact subjects biweekly to complete a brief service use and housing status questionnaire. A structured recall method was employed to account for where the respondent slept each of the last 14 nights. Binary outcomes extrapolated from continuous outcaomes. Data available at intake and 24 months follow up.
Slesnick 2015 (74)	
**Methods**	RCT. Randomized N=270, Analyzed N=270, CRA (N=93) vs MET (N=86) vs CM (N=91) The participants were recruited between 2006 and 2009
**Participants**	**Eligibility**: Met criteria of homelessness as defined by the McKinney –Vento Homeless Assistance Act (2002) as those who lack a fixed, regular and adequate night‐time residence,; lives in a welfare hotel, or place without regular sleeping accommodations; or lives in a shared residence with other persons due to the loss of one's housing or economic hardship, 14‐20 years old, met DSM‐IV for abuse or dependence for psychoactive substance use or alcohol disorder **Sample**: Age (mean (SD)): 18.74 (1.26) Gender (% women): 47.41% Ethnicity (% African American): 65,56 Mental illness (%): NR Substance abuse (first time using drugs under 15,%): 75.56% Homeless status (mean age at first time homeless (SD), mean number of days currently without shelter (SD)): 15.89 (3.44), 69.20 (175.94) Criminal (ever incarcerated): NR **Location**: Ohio, USA
**Interventions**	**Case management** ‐ Using a strengths‐based case management (CM) model, case managers seek to link participants to resources within the community. ‐ The case manager reviews each of six general areas with the participant to gather a history and picture of the current situation: (1) housing needs; (2) health/mental health care, including alcohol/drug use intervention; (3) food; (4) legal issues, (5) employment and (6) education. ‐ Consistent with a strengths‐based CM approach, the case manager takes responsibility for securing needed services for the youth and remains a support for the youth as he/she traverses the system of care. ‐ The strengths‐based approach also includes the following features: 1) dual focus on client and environment, 2) use of paraprofessional personnel, 3) a focus on client strengths rather than deficits, 4) a high degree of responsibility given to the client in directing and influencing the intervention that he/she receives from the system and the outreach worker. ‐ Once this review is complete, an initial intervention plan is developed with specific goals and objectives. ‐ A manual and goal development sheets were developed by the first author. Service is not restricted to the office and includes transportation of clients to appointments, interviews, and related activities. Training included manual review, didactic training and extensive role play over a period of 2 days, as well as weekly supervision with audiotape review with the intervention supervisor throughout the study. Therapists included master's level counselors, marriage and family therapists or social workers. Case managers were bachelor's level social work students, and counseling was not provided. *Conditionality: NA* *Housing provision: NO* *Segregation: NA* *Case management intensity: once per week, during the initial treatment phase, counseling sessions may be scheduled more frequently than once per week. The intervals between sessions can then be extended as the client's abstinence becomes more stable* **Motivational Enhancement therapy** ‐ Assumes that the responsibility and capability for change lie within the client, and need to be evoked (rather than created or instilled). ‐ Four principles guide the practice of MI: express accurate empathy, develop discrepancy, roll with resistance and support self‐efficacy. ‐ An adaptation of MI that has been well‐tested, both with adults and with adolescents, is motivational enhancement therapy (MET) which includes feedback. ‐ Although the frequency of MET sessions was lower than the other treatments, the duration of the treatment was matched with the other, longer treatments so that sessions were spaced over the course of the treatment period. ‐ Training included manual review, didactic training and extensive role play over a period of 2 days, as well as weekly supervision with audiotape review with the intervention supervisor throughout the study. Therapists included master's level counselors, marriage and family therapists or social workers. Case managers were bachelor's level social work students, and counseling was not provided. *Conditionality: NA* *Housing provision: NO* *Segregation: NA* *Case management intensity: the frequency of MET sessions was lower than the other treatments, the duration of the treatment was matched with the other, longer treatments so that sessions were spaced over the course of the treatment period* **Community reinforcement approach** CRA is an operant‐based therapy with the goal to help individuals restructure their environment so that drug use or other maladaptive behaviors are no longer reinforced and other positive behaviors are reinforced. ‐ CRA treatment procedures are detailed in a book written by the developers (Meyers & Smith, 1995). ‐ Therapists follow a standard set of core procedures and a menu of optional treatment modules matched to clients’ needs, including (1) a functional analysis of using behaviors, (2) refusal skills training, and (3) relapse prevention (4) job skills, (5) social skills training including communication and problem‐solving skills, (6) social and recreational counseling, (7) anger management and affect regulation. ‐ Each area of focus is determined based upon the goals of counseling, and intervention components are repeated until the participant and therapist agree that the goal has been achieved. ‐ The intervention is tailored to the unique needs and environmental context of individual clients, so it is easily adapted to the multiple and various circumstances of those experiencing homelessness (e.g., limited recreational/social reinforcers). ‐ Training included manual review, didactic training and extensive role play over a period of 2 days, as well as weekly supervision with audiotape review with the intervention supervisor throughout the study. Therapists included master's level counselors, marriage and family therapists or social workers. Case managers were bachelor's level social work students, and counseling was not provided. *Conditionality: NA* *Housing provision: NO* *Segregation: NA* *Case management intensity: once per week, during the initial treatment phase, counseling sessions may be scheduled more frequently than once per week. The intervals between sessions can then be extended as the client's abstinence becomes more stable*
**Outcomes**	**Homelessness** **Other**: Alcohol use, Drug use, Depressive symptoms Internalizing and externalizing problems, Coping skills **Measurement**: 3, 6, 12 months
Smith 1998 (75)	
**Methods**	Single‐blinded RCT Randomized N=106, Analyzed N=81, CRA (n_1_=64) vs. UC (n_2_=42) Recruitment period not reported.
**Participants**	Eligibility: Homeless (chronic) and alcohol dependence Sample description: mean age 38, women 14%, most participants identified as white (64%). Details on mental illness, substance use, homeless status and criminal background were not reported Location: Albuquerque, New Mexico, USA
**Interventions**	Community Reinforcement Approach (CRA) Assumption: environmental contingencies play a powerful role in encouraging or discouraging drinking. CRA uses social, recreational, familial, and vocational reinforcers to assist clients in reducing their alcohol intake. CRA offers a multifaceted approach to alcohol treatment that addresses many of the needs of homeless individuals. CRA therapists: behaviorally oriented advanced clinically psychology graduate students trained in the CRA protocol. The CRA skills‐training groups were offered on weekdays at the shelter, focused on problem solving, communication, drink refusal, independent living goal setting. A disulfiram compliance group was conducted daily for individuals who were taking disulfiram. The project nurse and the group members served as the monitor. Additionally a social club event was held weekly off‐site in an effort to provide a reinforcing nondrinking recreational activity, a job club was run (e.g. for job seeking assistance), and couples therapy was offered to CRA group members with partners. CRA is not a CM intervention but a treatment model for homeless persons with alcohol dependence. Treatment length varied according to individual needs. In general CRA participants were expected to attend groups full time for a minimum of 3 weeks and to remain involved in the program while living in grant‐supported housing. Hosunig is transitional: normal length of stay 3 months, individuals with secured job and saved agreed‐upon amount of money could remain a 4th month. Apartments were shared by 2‐4 participants. *Conditionality of tenancy*: abstinence was required when living together in grant‐provided housing; Random Breathalyzer tests used at the apartments, offenders were suspended from housing for 1‐2 weeks; individuals allowed to return once attended CRA groups sober daily during week of suspension *Housing provision*: grant‐supported apartments. *Segregation*: no information *Case management intensity*: not applicable Usual care Day shelter's services, free meals, showers, clean cloths, telephones, and mail services. Additionally, a master's‐level 12‐step substance abuse counselor with 17 years of experience offered individual sessions, Alcoholics Anonymous (AA) meetings were held on‐site, and job program arranged temporary employment. Finally, case managers were available for the dually diagnosed. *Conditionality of tenancy*: abstinence was required when living together in grant‐provided housing; Random Breathalyzer tests used at the apartments, offenders were suspended from housing for 1‐2 weeks; individuals allowed to return once attended CRA groups sober daily during week of suspension *Housing provision*: grant‐supported apartments. *Segregation*: no information *Case management intensity*: case management only for dually diagnosed, no information on intensity
**Outcomes**	Stably housed: independent living, including paying for a more permanent dwelling, no further information Homeless: not stably housed (extrapolated) Measurement: no information, data available for intake and 12 months follow up.
Solomon 1995 (76)	
**Methods**	RCT Randomized: N=96 (ACT: 48 UC: 48), Analyzed: N=90 Participants were recruited between 1990 and 1991.
**Participants**	**Eligibility** Diagnosis of a major mental illness; Significant treatment history, such as state hospitalization for a minimum of 60 days within the past 2 years; continuous attendance at a community mental health service for 3 or more years; five or more face‐to‐face contacts with a psychiatric emergency service within the past 2 years: Disability as indicated by a Global Assessment Scale (GAS) 27 score of 40 or below if the patient is over; age 35 and 60 or below if the patient is age 35 or younger. **Sample:** Age (mean (SD)): 41 (14.4); Gender (% male): 52%; Ethnicity (% African American): 79%; Mental illness (%): Schizophrenia: 86%, Major affective disorder: 13%, Unspecified psychotic disorder: 1%, Substance use (% use past 30 days): Alcohol: 13%, Drug: 4%, Alcohol & drug: 3%; Homeless (% in past year): 12% (% in lifetime: 21%; Criminal (% arrest past year): 17%, (% arrests during lifetime): 41% **Location:** Philadelphia, Pennsylvania, USA
**Interventions**	**Consumer case management (ACT model):** The teams in each service condition were composed of four case managers. The consumer team initially comprised three consumer case managers and one nonconsumer case manager. The nonconsumer member left the position and was replaced with a consumer. The consumer team, in a self‐help organization, also had a full‐time project director who was a consumer, so it eventually became composed entirely of consumers. In the second year of the program, a full‐time clinical director and a part‐time psychiatrist were hired. The consumer team formally met three times per week. The consumer team of case managers received individual supervision weekly from the project director, a consumer, and they received additional clinical support from the psychiatrist and clinical director, a nonconsumer. The clinical director also assisted the case managers in serving their clients. *Conditionality of tenancy: NA* *Housing provision: NA* *Segregation: NA* Case *management intensity: There was no significant difference in the total number of time units (15‐minute units reported to the mental health system) of service in the first year of the program between consumer (M = 335.16, SD = 254.85) and nonconsumer (M = 258.02, SD = 218.47) teams. However, consumer case managers provided more services face to face with their clients (M = 152.80, SD = 134.52) than* *nonconsumer case managers (M = 39.80, SD = 42.16), t(df= 53.93) = 20.81, p < 001. In contrast, consumer case managers provided fewer office‐based services (M = 52.04, SD = 55.07) than nonconsumers (M = 253.18, SD = 172.59), t(df= 52.69) = 26.98*, p < .001.* **Nonconsumer case management (ACT model):** The nonconsumer team, part of a community mental health center, was supervised by a case manager supervisor who oversaw another team as well. In the second year of the project, the nonconsumer team added two part‐time specialists who worked with the case managers. They performed such functions as helping in crisis situations, engaging in social activities with clients, and generally filling in when a case manager was on vacation. This arrangement was instituted when one of the case managers reduced her time. While there were changes in the composition of the two teams based on the desires of the supervising organizations, the integrity of the service conditions remained, as one team was composed of consumers and the other of the more customary nonconsumer case managers. The nonconsumer team, during the course of the 2‐year period, lost one case manager, and the consumer team lost three case managers. The nonconsumer team met biweekly and received individual supervision from the intensive case management supervisor/clinical director on a weekly basis. They also met with another team of intensive case managers on a monthly basis. *Conditionality of tenancy: NA* *Housing provision: NA* *Segregation: NA* *Case management intensity: There was no significant difference in the total number of time units (15‐minute units reported to the mental health system) of service in the first year of the program between consumer (M = 335.16, SD = 254.85) and nonconsumer (M = 258.02, SD = 218.47) teams. However, consumer case managers provided more services face to face with their clients (M = 152.80, SD = 134.52) than nonconsumer case managers (M = 39.80, SD = 42.16), t(df= 53.93) = 20.81, p < 001. In contrast, consumer case managers provided fewer office‐based services (M = 52.04, SD = 55.07) than* *nonconsumers (M = 253.18, SD = 172.59), t(df= 52.69) = 26.98, p < .001.** As is consistent with an Assertive Community Treatment model, both teams of case managers saw clients in vivo—in the environments where the clients lived, attended programs, received treatments, and socialized. Through such strategies, case managers offered individualized social support for community living. Each team member had his or her own clients. In crisis situations, and sometimes for social activities with their clients, team members worked together. Otherwise each case manager functioned relatively independently in serving his or her own clients. Case management activities were those which met goals determined with the client. These included goals related to income, living situation, social and family relations, and psychiatric treatment. Toward this end, case managers routinely interacted with medical professionals, community and social
**Outcomes**	**Housing stability** **Homelessness** **Other**: arrests; amount and source of income; drug and alcohol use subscales of the Addiction Severity Index; family and social contacts; Pattison's Social Network; level of functioning and quality of life, both subjective and objective aspects, using Lehman's 36 Quality of Life Interview. The Brief Psychiatric Rating Scale (BPRS) was also completed at the time of the interviews **Measurement:** 12, 24 months
Sorensen 2003 (77)	
**Methods**	RCT. Randomized: N=190, Analyzed: 190, CM (N=92) vs BC (98) The participants were recruited between 1994 and 1996 (Sorensen 2003)
**Participants**	**Eligibility:** Adult patients who met DSM‐IV criteria for substance dependence, had HIV infection (verified in medical charts with CD4≥50 in last 6 mos), and were willing to provide informed conset, locator information and urine specimens. Patients were excluded if they were currently enrolled in substance abuse treatment or case management, diagnosed with medical conditions indicating they would likely be deceased within 6 months, non‐residents of San Francisco, or in police custody. **Sample** Age (mean (SD)): BC: 38 CM: 39 Gender (% female) 100%: Ethnicity (% African American): 56% Mental illness (GSI mean score (SD)): 57.7 (12) Substance abuse: NR Homeless status: 100% come from shelters Criminal: NR Other: Brief contact participants slightly younger (38 vs 39, t(188)=2.06, p=0.0414) **Location**: San Francisco, California, USA
**Interventions**	**Case management** Site of service: community and hospital Team structure: individual case loads Hybrid between brokerage and full‐service models and included elements of service brokerage (advocating for client entry to programs) and counselling (continuing contact with patients through a 1 year period). Case managers focused on linking patients with services that included medical care, psychiatric treatment, legal assistance, and social service entitlements such as low‐income housing and supplemental security income (SSI). Case managers made appointments for evaluation and follow‐up care and accompanied patients to these appointments. They educated patients about drugs, HIV, safe sex and helped them to obtain condoms and referred them to clean needle‐exchange. Paraprofessionals, former consumers of HIV or substance abuse treatment services (abstinent for at least 2 years before starting work) and certified chemical dependency counsellors with successful work history in treatment programs with 1 week orientation to programs policies and procedures and supervision from licensed clinical social worker in the beginning of working. *Conditionality of tenancy: NA* *Segregation: NA* *Housing provision: No* *Case management intensity: 1 year, multiple sessions in a staff to client ratio of 1:20 and follow‐up on referrals* **Brief Contact** Site of service: hospital only Team structure: counsellors sharing clients Department of Psychiatry at San Francisco General Hospital provided brief contact and referral through its AIDS and Substance Abuse Program (ASAP). ASAP workers received a referral and then met with patient at hospital program. They provided education about reducing risk of HIV transmission, HIV services and referrals to substance abuse treatment, social services and HIV services in the community. ASAP workers included both professionally trained individuals and paraprofessionals *Conditionality of tenancy: NA* *Segregation: NA* *Housing provision: No* *Case management intensity: until discharge, one or two sessions of contact in a staff to client ratio of about 1:100, no follow‐up*
**Outcomes**	**Homelessness**(not reported how measured) **Other**: Substance use (ASI), Physical health, Psychological status, Social support network, ASI psychiatric, ASI legal, ASI family **Measurement**: 6, 12, 18 months
Sosin 1995 (26)	
**Methods**	**Non‐blinded RCT** Randomized N=419, Analyzed N=299, CM (n_1_=70) vs. CM+supported housing (n_2_=108) vs. UC (n_3_=121) Participants were recruited from May 1991 to October 1992. Data collected at baseline, and 12 months follow up
**Participants**	**Eligibility** homeless or at risk, substance abuse (recruitment from detox treatment) **Sample description** The following is a description of the complete sample (all three trial arms): Approximately 78% of participants were available at the six month follow up, and 74% at the 12 month follow‐up. The average age of the participants was 35 (data missing for two participants), approximately one quarter were female (25.5%) and 90% were African American. Participants had experienced almost 26 months of homelessness on average prior to the study (average of 18 of the previous 60 days homeless at baseline), and reported an average of approximately 18 days of alcohol/drug use in the 60 days prior to baseline. **Location** Chicago, Illinois, USA
**Interventions**	**The progressive independence model case management** Probably ordinary case management, but also provision of immediate tangible resources ‐ transportation tokens, food vouchers, medical care, and furniture and rent deposits (for those with long‐term ability to support themselves) ‐ while supporting further treatment for abuse and other relevant personal and situational problems. Provision is conditioned on attendance in outpatient and Alcoholics Anonymous meetings in the community and clients must remain abstinent from drugs and alcohol, and must sign a contract agreeing to cooperate with the (negotiated) treatment plan. Those who do not keep these agreements are first confronted with their behavior; if the problems continue, the clients are suspended, or askedto withdraw if the issues cannot be resolved. Individuals are required to progressively take responsibility for: obtaining employment, work training, or if neither is available, welfare benefits attending the project's group and individual counseling concerning intrapersonal, relationship, and permanent housing issues cooperating with a cognitive behavioral relapse prevention model that is utilized by case managers. *Conditionality of tenancy*: not applicable *Housing provision*: not applicable *Segregation*: not applicable *Case management intensity*: no information **The progressive independence model case management and supported housing** Same case management as above but also supported housing in one of three blocks of twenty apartments, found in recently renovated buildings serving those with low incomes. Those who suffered two relapses or repeatedly violated program rules could not remain in the housing. They could continue case management as long as they agreed to a new contract that would guard against further relapses. *Conditionality of tenancy*: abstinence, treatment compliance and program rules. *Housing provision*: no information *Segregation*: no information *Case management intensity*: no information **Usual care (after care, referrals to outpatient or inpatient substance abuse agency, welfare offices, and to some kind of address)** *Conditionality of tenancy*: no information *Housing provision*: no information *Segregation*: no information *Case management intensity*: no information
**Outcomes**	Number of days in stable housing Numbwer of days of alcohol or drug use of previous 60 days
Stefancic 2007 (78)	
**Methods**	**Non‐blinded RCT** Randomized N=260, Analyzed N=138, HF+ACT (CHF+PTH n_1_=209) vs. UC (n_2_=51) Participants recruited 200‐2004
**Participants**	**Eligibility**: Chronic shelter use, severe mental illness **Sample description**: mean age (no information), women 26%, schizophrenia 42%, major depressive disorder 13%, bipolar disorder 19%, schizoaffective disorder 6%, other disorder 14%, diagnosis no information 7%, alcohol dependence/abuse 40%, alcohol dependence/abuse in remission 13%, drug abuse/dependence 41%, drug abuse/dependence in remission 13%, Homeless status: NR, Criminal: NR **Location**: Suburban New York, USA
**Interventions**	**Housing first with assertive community treatment (HF+ACT)** HF provides permanent, independent housing without prerequisites for sobriety and treatment, and offers support services through consumer‐driven ACT teams. HF promotes consumer choice, recovery, and community integration. Housing is separated from treatment. Addressing the consumer's needs first is the guiding principle for all subsequent services that are offered and is the foundation for building trusting and supportive clinical relationships. No indication of any deviation of ACT from original program. *Conditionality of tenancy*: HF programs offer immediate access to permanent independent housing, without requiring treatment compliance or abstinence from drugs or alcohol. Consumers can refuse formal clinical services, such as taking psychiatric medication, seeing a psychiatrist, or working with a substance use specialist, yet programs have requirements for a minimum of one visit per week by the team. As tenants, consumers remain housed as long as they meet the obligations of a standard lease. As in most supportive housing programs, consumers have an obligation to pay 30% of their income towards rent (typically, 30% of their Supplemental Security Income). The adverse consequences of relapse into substance abuse or a psychiatric crisis are mitigated because relapse is addressed by providing intensive treatment or facilitating admission to detox or hospital to address the clinical crisis ‐ not by eviction because the consumer is using or experiencing psychotic symptoms. After completing treatment for their clinical conditions, consumers return to their apartments. *Housing provision*: Apartments are rented from private landlords by the program, consumers have their own lease or sublease and have the same rights of tenancy as other residents in their buildings. HF offer housing in the form of scatter‐site independent apartments in buildings rented from private landlords. *Segregation*: Housing is integrated. To maintain integration, the program does not lease more than 15% of the units in any one building. *Case management intensity*: no information on case load (probably<1:15), availability 24/7. **Usual care (UC)** The county's usual array of services that included shelter‐based programs and transitional housing. *Conditionality of tenancy*: no information *Housing provision*: no information *Segregation*: no information *Case management intensity*: no information
**Outcomes**	**Stably housed**: No definition, “permanent housing”, “scattered‐site housing” **Homeless**: no definition: shelter use, but not “permanent housing”, not “scattered‐site housing” **Measurement**: data were collected from administrative records as well as the respective Housing First agencies. Each month, the two Housing First agencies submitted reports to the Department of Social Services indicating the number of consumers whom they had outreached/engaged, the number of consumers currently remaining in housing, and the number of consumers no longer housed. Residential data for Housing First consumers were available continuously for just under four years (47 months). Residential data for control participants were obtained through the county's computerized shelter tracking system, but were only available at the 20‐month time‐point. Because data were not available for all three groups throughout the study follow‐up period, two types of housing outcomes are presented. Housing status, was a single point‐in‐time count of the number of persons housed within the two Housing First groups and the control group at 20 months.
Susser 1997 (79)	
**Methods**	**Singel‐blinded RCT** Randomized N=96, Analyzed N=96, CTI+UC (n_1_=48) vs. UC only (n_2_=48) Participants recruited 1991‐1993
**Participants**	**Eligibility**: Sheltered men with severe mental illness (at risk of becoming homeless implied) **Sample description**: mean age 35, women 0%, schizophrenia (life time diagnosis) 68%, other (life time diagnosis) 32%, psychiatric hospitalizations > 5 times 64%, cocaine dependence (life time diagnosis) 47%, alcohol dependence (life time diagnosis) 54%, Homeless status: 78% homeless > 1 yr, Criminal: NR **Location**: NYC, USA
**Interventions**	**Critical time intervention (CTl)** A strategy to prevent homelessness by enhancing the continuity of care for individuals being discharged from institutional to community living. CTl creates a bridge between institutional and community care at a critical time in the deinstitutionalization process. CTI is intended for use by a broad range of institutions, including shelters, hospitals, and jails, and for prevention of first episodes of homelessness as well as recurrent homelessness. CTI is based on intensive case management (ICM). There are three phases preparing for the fourth phase when usual care begins: (1) Accommodation (1‐3 months): CTI workers make home visits, accompany patients to appointments, meet with care givers, substitute care givers when necessary, give support and advice to patient and caregiver, mediate conflicts between patient and caregiver, help negotiate ground rules for relationships. (2) Tryout (4‐7 months): CTI workers observe trial of ground rules, help negotiate ground rules as necessary. (3) Termination (8‐9 months): CTI workers reaffirm ground rules, hold parties and meetings to symbolize transfer of care. (4) Usual services begin (10‐18 months) *Conditionality of tenancy:* Depend of ground rules. *Housing provision:* No specific information, a variety of usual services and housing in NYC. *Segregation:* No specific information, a variety of usual services and housing in NYC. *Case management intensity*: probably high intensity, CTI is a short and time limited form of intensive case management. No further information. **Usual care (UC)** Two phases Transition of services (1‐3 months): shelter staff assist patients and caregivers upon request, and substitute for caregivers when necessary Usual services (4‐7 months): services provided by community formal and informal supports, and patients and caregivers can phone for advice *Conditionality of tenancy*: no information *Housing provision:* no specific information, a variety of usual services and housing in NYC. *Segregation:* no specific information, a variety of usual services and housing in NYC. *Case management intensity:* no specific information.
**Outcomes**	**Stably housed**: extrapolated (not homeless) **Homeless**: night spent in a shelter or public space. **Other**: Psychiatric symptom severity (Positive and Negative Syndrome Scale **Measurement**: After randomization, face‐to‐face assessments were conducted every 30 days for 18 months. Trained interviewer blind to the client's group status, who documented where the client had spent each night. In cases in which a man had missed an assessment, the interviewer always documented the housing experience of each night since the last completed assessment. The man's residential experience was continuously traced for each night over the 18‐month follow‐up period. Occasionally, when a man could not be directly interviewed, the assessment was conducted with a key informant such as a close relative or a caseworker. Binary outcomes extrapolated from continuous outcomes. Data available at intake, 6, 12 and 18 months follow up.
Toro 1997 (80)	
**Methods**	**Single‐blinded RCT** Randomized N=202, Analyzed N=105, DEPTH/ICM (n_1_=101) vs. no‐treatment (NT/UC) (n_1_=101) Participants recruited 1990
**Participants**	**Eligibility**: homeless adults with family members (not clearly defined), N=202 cases including 213 adults and 70 children **Sample description (N=213)**: mean age (our calculation) 35, women 42%, mental illness 20%, major affective disorder 19%, schizophrenic disorder 3%, substance abuse/dependence 58%, alcohol abuse 46%, drug abuse 38%, Homeless status: 5% homeless more than 2 years, 36% with prior homeless episodes, Criminal: 25% arrested, Other: 25% veteran **Location**: Buffalo, New York, USA
**Interventions**	**Demonstration Employment Project ‐ Training and Housing (DEPTH/ICM)** A holistic approach combining services concerned with job training/placement, locating permanent housing and support services, all targeted to the individual's specific needs and oriented toward the long‐term goal of helping the person to escape homelessness. DEPTH addressed the clients’ immediate tangible needs. For example funds were sometimes loaned to cover the security deposit for a new apartment and program staff helped clients obtain donated furniture and appliances and find quality day care for their children. Central to DEPTH's services was *intensive case management*, offering access and linkage to services (e.g. financial aid, housing support, counseling for drug and alcohol problems, mental health assessment and treatment, and job training). If appropriate services could be identified in the community, DEPTH staff would provide it. DEPTH adapted its model of intensive case management from a variety of sources, including ones from the mental health field. *Conditionality of tenancy*: no information *Housing provision*: no detailed information, a possible mix is indicated. *Segregation*: no detailed information, a possible mix is indicated. *Case management intensity*:DEPTH clients had a median of 41 staff contacts (averaging about 45 min) over a 4‐ to 8‐month active intervention period. No information on case load or availability. **No‐treatment control group (NT/UC)** Those in no‐intervention control group received none of the DEPTH's services, but were free to seek whatever other services were available to them in the community. Compensation for the lack of referral to DEPTH by seeking additional services in the community: research participants at each follow‐up interview reported on services received in seven categories (i.e. impatient mental health or substance abuse care, outpatient mental health or substance abuse counseling, child or family counseling, financial counseling, vocational counseling, crisis services, and self‐help groups). No significant group differences (p>.10) were found on any of these services and, overall, 59% of DEPTH clients received one or more of these services during the follow‐up period, compared with 51% of the controls. *Conditionality of tenancy:* not applicable *Housing provision:* not applicable *Segregation:* not applicable *Case management intensity:* not applicable
**Outcomes**	**Stably housed**: (extrapolated, not homeless) **Homeless**: not defined, number of days homeless past 180 days **Other**: Employment (Income) **Measurement**: Binary outcomes extrapolated from continuous outcomes. Data available at intake, 6, 12 and 18 months follow up.
Tsemberis 2004 (24)	
**Methods**	**Semi‐blinded RCT** Randomized N=225, Analyzed N=198‐175, HF‐ACT (n_1_=99) vs. CoC‐UC (n_2_=126) Participants recruited 1997‐2001
**Participants**	**Eligibility**: Homeless (chronic), severe mental illness **Sample description**: mean age (our calculation) 41, women 21%, psychotic 53%, mood/depressive 14%, mood/bipolar 14%, other diagnosis 5%, unknown 14%, diagnosis or history of alcohol or substance abuse disorders 90%, Homeless status: 51% literally homeless at time of entry to study, Criminal: NR **Location**: NYC, USA
**Interventions**	**Housing first with modified assertive community treatment (HF+ACT)** A consumer's choice program: psychiatric rehabilitation for chronically homeless persons. Needs are addressed from the consumer's perspective, and are encouraged to define their own needs and goals and. Housing, a basic right. An apartment is immediately provided without prerequisites for psychiatric treatment or sobriety. Housing and treatment are separate domains. Consumers may accept housing and refuse clinical services without housing status consequences. A harm‐reduction approach in clinical services regarding alcohol/drug abuse, psychiatric symptoms or crises. Consideration of consumers different stages of recovery, interventions are individually tailored to each consumer's stage. An Assertive Community Treatment (ACT) team, a community based inter‐disciplinary team including social workers, nurses, psychiatrists, and vocational, substance abuse counselors and two additional team‐workers: a nurse addressing health problems, and a housing specialist coordinating housing services (modifications of the standard ACT‐model). *Conditionality of tenancy*: no requirements regarding treatment compliance or sobriety. Tenants must pay 30% of their income toward the rent by participating in a money management program, tenants must meet with a staff member a minimum of twice a month, and follow standards rules for ordinary tenants. Requirements are applied flexibly to suit consumers’ needs. *Housing provision:* Housing is provided by the market, acquisition comes from landlord and brokers, but identification and negotiation is done by staff members of Pathways to Housing and temporary solutions are provided by the agency. *Segregation:* Housing is not segregated. *Case management intensity*: high, case load (no information), availability 24/7. **Continuum of care, usual care (CoC/UC):** Information is poor. The continuum of care model begins with outreach, includes treatment and transitional housing, and ends with permanent supportive housing. The purpose of outreach and transitional residential programs is to enhance clients’ “housing readiness” by encouraging the sobriety and compliance with psychiatric treatment considered essential for successful transition to permanent housing. It is assumed that individuals with severe psychiatric disabilities cannot maintain independent housing before their clinical status is stabilized and that the skills a client needs for independent living can be learned in transitional congregate living. A typical program would be exemplified by a group home or a single‐room occupancy residence in which clients are expected to attend day treatment, 12‐step, and other therapeutic groups and follow medication regimens enforced by on‐site staff. Sleeping, cooking, and bathing facilities are shared *Conditionality of tenancy:* Information is poor. Most programs have rules that restrict clients’ choices and that when violated are used as grounds for discharging the consumer from the program. For example, despite having attained permanent housing, clients who relapse and begin to drink mild or moderate amounts of alcohol, may be evicted if the program has strict rules about sobriety maintenance. Continuum of Care supportive housing programs subscribe to the abstinence‐sobriety model based on the belief that without strict adherence to treatment and sobriety, housing stability is not possible. The usual care programs offer abstinent contingent housing and services based on a treatment first model. House rules strictly prohibit consumption of any substances and overnight guests. *Housing provision*: no information. *Segregation*: no information. *Case management intensity:* Information is poor, probably a variety.
**Outcomes**	**Stably housed**: residing in one's own apartment; or having a room or studio apartment in a supportive housing program, a group home, a boarding home, or a long‐term transitional housing program; or living long‐term with parents, friends, or other family members. **Homeless**: living on the streets, in public places, or in shelter‐type accommodations. Measurement: number of days spent in any of the locations categorized as “homeless” was summed and divided by the total number of days of residency reported at the interview. Period was past 6 months. The mean percentage have here been multiplied with the number of persons in each group, and in this way the number of homeless persons have been estimated. **Other:** Substance use, psychiatric symptoms **Measurement**: number of days spent in any of the locations categorized as “stably housed” was summed and divided by the total number of days of residency reported at the interview. Period was past 6 months. The mean percentage have here been multiplied with the number of persons in each group, and in this way the number of housed persons have been estimated. Binary outcomes were extrapolated from continuous outcomes (graphically estimated). Data available at intake, 6, 12, 18, and 24 months follow up.
Wolitski 2010 (81)	
**Methods**	multi‐site RCT. Randomized N=630, Analyzed N=629, HOPWA (N=315) vs CM+H (N=315) Participants recruited 2004‐2007
**Participants**	**Eligibility:** (1) 18 years of age or older, (2) HIV‐seropositive, (3) homeless or at severe risk of homelessness, (4) had income less than 50% of median area income, (5) spoke English or Spanish, and (6) were willing and able to provide informed consent **Sample**: Age (mean): 40 Gender (% male): 67.7% Ethnicity (% African American): 78.5% Mental illness (%): NR Substance abuse (%): NR Homeless status (homeless past 90 days): 27.3% Criminal (ever incarcerated): 67.7% **Location:** Baltimore, Chicago, Los Angeles, USA
**Interventions**	**Immediate rental (financial) assistance with case management** · Specialists assisted treatment condition participants with initiating HOPWA financial rental assistance and locating housing of their choosing. · The amount of assistance varied depending on the Fair Market Rent and each participant's monthly income. · In both conditions, specialists assessed participants’ need for health services and provided referrals as appropriate. *Conditionality of tenancy: NR* *Segregation: NR* *Housing provision: No* *Case management intensity: NR* **Customary housing services with case management** · Those assigned to the comparison condition received assistance with developing a housing assistance plan that utilized all of the agency's customary services. · Comparison condition participants were not required to stay in their current living situation and were not restricted in any way from obtaining rental assistance or housing from other sources. · In both conditions, specialists assessed participants’ need for health services and provided referrals as appropriate. *Conditionality of tenancy: NR* *Segregation: NR* *Housing provision: No* *Case management intensity: NR*
**Outcomes**	**Stably housed**: unstably housed **Homeless**: percentage homeless > 1 night **Other**: physical and mental health, HIV medication use and adherence, substance use **Measurement:** 6, 12, 18 months

## Appendix 8: GRADE Evidence profiles

### Category 1: Case management

#### Table 8.1: Comparison 1.A.1 ‐ GRADE Evidence profile for high intensity case management versus usual services

**Author(s)**: Heather Munthe‐Kaas, Rigmor Berg

**Date**: 11.11.2016

**Question**: High intensity case management compared to usual services for adults who are homeless or at‐risk of becoming homeless

**Setting**: USA

**Bibliography**: Bell 2015, Bond 1990, Clarke 2000, Cox 1998, Garety 2006, Killaspy 2006, Lehman 1997, Morse 1992, Morse 2006, Nordentoft 2010, Rosenheck 2003

**Table:**

**Quality assessment**							**№ of patients**		**Effect**		**Quality**	**Importance**
**№ of studies**	**Study** **design**	**Risk of bias**	**Inconsistency**	**Indirectness**	**Imprecision**	**Other considerations**	**high intensity case management**	**usual services**	**Relative** **(95% CI)**	**Absolute** **(95% CI)**		
Mean number of days in stable housing (own residence, living in community, private rent accommodation) (follow up: range 12 months to 24 months; assessed with: self‐report)												
5	randomised trials	very serious ^1^	serious ^2^	not serious	not serious	none	571	569	‐	SMD **0.09 SD more** (0 to 1.79 more)	⨁◯◯◯ VERY LOW	
Number of participants living in stable community housing at follow‐up (follow up: range 12 months to 18 months; assessed with: self‐report)												
2	randomised trials	serious ^3^	not serious	not serious	serious ^4^	none	89/113 (78.8%)	71/113 (62.8%)	**RR 1.26** (1.07 to 1.49)	**163 more per 1,000** (from 44 more to 308 more)	⨁⨁◯◯ LOW	
Number of participants homeless at follow‐up (follow up: range 12 months to 18 months; assessed with: homeless)												
3	randomised trials	serious ^5^	not serious	not serious	not serious	none	39/423 (9.2%)	58/383 (15.1%)	**RR 0.59** (0.41 to 0.87)	**62 fewer per 1,000** (from 20 fewer to 89 fewer)^12^	⨁⨁⨁◯ MODERATE	
Number of days homeless (follow up: range 12 months to 24 months; assessed with: self‐report)												
6	randomised trials	very serious ^6^	not serious	not serious	not serious	none	562	636	‐	SMD **0.27 SD fewer** (0.46 fewer to 0.09 fewer)	⨁⨁◯◯ LOW	
Number of participants who experienced some homelessness (follow up: 24 months; assessed with: not reported)												
3	randomised trials	serious ^8^	not serious	not serious	serious ^9^	none	42/280 (15.0%)	28/235 (11.9%)	**RR 1.08** (0.69 to 1.72)	**10 more per 1,000** (from 37 fewer to 86 more)^7^	⨁⨁◯◯ LOW	
Number of days in longest residence during previous 6 months (follow up: 12 months; assessed with: not reported)												
1	randomised trials	very serious ^10^	not serious	not serious	serious ^11^	none	34	24	‐	MD **16.3 days fewer** (0 to 0)	⨁⨁◯◯ VERY LOW	
Number of clients who did not move during previous 6 months (follow up: 12 months; assessed with: not reported)												
1	randomised trials	very serious ^10^	not serious	not serious	serious ^11^	none	21 (62%) of HICM participants and 17 (77%) of usual services participants did not moved during this period (x2(1)=1.47, ns).				⨁⨁◯◯ VERY LOW	
Mean number of moves during previous 6 months (follow up: 12 months; assessed with: not reported)												
1	randomised trials	very serious ^10^	not serious	not serious	serious ^11^	none	Participants in the HICM Group reported M=0.56 moves compared to M=0.29 for the usual services Group (t(53)=‐1.39, ns).				⨁⨁◯◯ VERY LOW	

**CI:** Confidence interval; **SMD:** Standardised mean difference; **RR:** Risk ratio; **MD:** Mean difference

Risk of performance bias in all studies. Risk of attrition bias in three studies, risk of detection bias in two studies and risk of selection bias in one study. Inadequate reporting of randomization and/or allocation concealment methods in two studies and blinding of outcome assessors in one study.

2. Considerable heterogeneity (I2=98%, chi2=186.17).

3. Risk of performance bias.

4. Fewer than 300 participants.

5. Risk of performance bias in all studies. Risk of attrition bias in one study.

6. Risk of performance bias in four studies, risk of detection bias in two studies, risk of attrition bias in two studies and other risks of bias in two studies. Unclear reporting of selection bias in four studies and detection bias in two studies.

7. Two studies includd in the pooled analysis (N=515). One study not included in the analysis, but shows a similar result: Bell 2012 (intervention N=567, control N=563) OR=0.83, 95%CI=0.60, 1.17.

8. Inadequate reporting of randomization, allocation concealment and blinding methods in two studies.

9. Total number of events is less than 300.

10. Risk of detection bias and attrition bias. Inadequate reporting of blinding methods for participants and personnel.

11. Fewer than 400 participants.

12. Two studies included in the pooled analysis (Garety 2006 (54), Killaspy 2006). Results from Nordentoft 2010indicate a smaller proportion of participants from the intervention group being homeless at 12 month follow‐up (10%; N=227) than the control group (17%; N=192) (OR=0.53 (95%CI 0.3, 0.9), p= 0.02).

##### Table 8.1.2. Comparison 1.A.2 – GRADE Evidence Profile for high intensity case management compared to low intensity case management

**Author(s)**: Heather Munthe‐Kaas, Rigmor Berg

**Date**: 11.11.2016

**Question**: High intensity case management compared to low intensity case management for improving housing stability and reducing homelessness

**Setting**: USA

**Bibliography**: Essock 2006; Drake 1998; Morse 1997

**Table:**

**Quality assessment**							**№ of patients**		**Effect**		**Quality**	**Importance**
**№ of studies**	**Study design**	**Risk of bias**	**Inconsistency**	**Indirectness**	**Imprecision**	**Other considerations**	**high intensity case management**	**low intensity case management**	**Relative** **(95% CI)**	**Absolute** **(95% CI)**		
Mean number of days spent in stable housing (follow up: 36 months; assessed with: self‐report)												
2	randomised trials	serious^1^	Serious^3^	not serious	not serious^2^	none	204	197	‐	SMD **0.1 SD higher** (0.1 lower to 0.29 higher)	⨁⨁◯◯ LOW	

**CI:** Confidence interval; **SMD:** Standardised mean difference

1. Risk of detection bias in one study. Inadequate reporting of methods in both studies.

2. Wide confidence intervals which include benefits and harms.

3. Inconsistency between results from the pooled analysis (two studies) and the third study that could not be included in the pooled analysis (Morse 1997). The third study reported that participants in the intervention group reported more days in stable housing than the control group (F=3.54, df=2, 129, p<0.032)

##### Table 8.1.3. Comparison 1.A.3 ‐ GRADE Evidence profile for High intensity case management compared to other intervention (no case management or housing component)

**Author(s)**: Heather Munthe‐Kaas, Rigmor Berg

**Date**: 11.11.2016

**Question**: High intensity case management compared to other intervention (no case management or housing component) for improving housing stability and reducing homelessness

**Setting**: USA

**Bibliography**: Nyamathi 2015

**Table:**

**Quality assessment**							**№ of patients**		**Effect**		**Quality**	**Importance**
**№ of studies**	**Study design**	**Risk of bias**	**Inconsistency**	**Indirectness**	**Imprecision**	**Other considerations**	**high intensity case management**	**other intervention (no case management or housing component)**	**Relative** **(95% CI)**	**Absolute** **(95% CI)**		
Number of participants who experience homelessness during study period (follow up: 12 months; assessed with: self‐report)												
1	randomised trials	serious^1^	not serious	not serious	very serious^2^	none	17/166 (10.2%)	20/177 (11.3%)	**RR 0.91** (0.49 to 1.67)	**10 fewer per 1 000** (from 58 fewer to 76 more)	⨁◯◯◯ VERY LOW	

**CI:** Confidence interval; **RR:** Risk ratio

1. Inadequate reporting of methods.

2. One small study. Wide confidence interval.

##### Table 8.1.4. Comparison 1.A.4 – GRADE Evidence Profile for the comparison of high intensity case management vs high intensity case management

**Author(s)**: Heather Munthe‐Kaas, Rigmor Berg

**Date**: 11.11.2016

**Question**: High intensity case management compared to high intensity case management for adults with major mental illness

**Setting**: USA

**Bibliography**: Solomon 1995

**Table:**

**Quality assessment**							**№ of patients**		**Effect**		**Quality**	**Importance**
**№ of studies**	**Study design**	**Risk of bias**	**Inconsistency**	**Indirectness**	**Imprecision**	**Other considerations**	**high intensity case management**	**high intensity case management**	**Relative** **(95% CI)**	**Absolute** **(95% CI)**		
Housing stability ‐ not reported												
‐	‐	‐	‐	‐	‐	‐					‐	
Ever homeless ‐ not reported												
‐	‐	‐	‐	‐	‐	‐					‐	

**CI:** Confidence interval

##### Table 8.1.5. Comparison 1.B.1 ‐ GRADE Evidence profile for the comparison of low intensity case management vs usual services

**Author(s)**: Heather Munthe‐Kaas, Rigmor Berg

**Date**: 11.11.2016

**Question**: Low intensity case management compared to usual services for improving housing stability and reducing homelessness

**Setting**: USA

**Bibliography**: Marshall 1995; Sosin 1995

**Table:**

**Quality assessment**							**№ of patients**		**Effect**		**Quality**	**Importance**
**№ of studies**	**Study design**	**Risk of bias**	**Inconsistency**	**Indirectness**	**Imprecision**	**Other considerations**	**low intensity case management**	**usual services**	**Relative** **(95% CI)**	**Absolute** **(95% CI)**		
Mean number of days in better housing (follow up: 14 months; assessed with: Unclear)												
1	randomised trials	very serious^1^	not serious	not serious	serious^2^	none	40	40	‐	MD **12 days more** (0 to 0)	⨁◯◯◯ VERY LOW	
Mean number of days in worse housing (follow up: 14 months; assessed with: unclear)												
1	randomised trials	very serious^1^	not serious	not serious	serious^2^	none	40	40	‐	MD **18.3 days fewer** (0 to 0)	⨁◯◯◯ VERY LOW	
Number of days in stable housing during past 60 days (follow up: 12 months; assessed with: self‐report)												
1	randomised trials	very serious^3^	not serious	not serious	serious^2^	none	70	121	‐	MD **5.7 days more** (0 to 0)	⨁ ◯◯◯ VERY LOW	

**CI:** Confidence interval; **MD:** Mean difference

1. Risk of performance bias. Inadequate reporting of methods for dealing with missing data and blinding.

2. Fewer than 400 participants. Unkown confidence interval

3. Risk of selection bias and attrition bias. Inadequate reporting of blinding methods.

##### Table 8.1.6. Comparison 1B.2 ‐ GRADE Evidence profile for the comparison of low intensity case management with an occupational therapist compared to low intensity case management without an occupational therapist

**Author(s)**: Heather Munthe‐Kaas, Rigmor Berg

**Date**: 11.11.2016

**Question**: Low intensity case management compared to low intensity case management for improving housing stability and reducing homelessness

**Setting**: USA

**Bibliography**: Chapleau 2012

**Table:**

**Quality assessment**							**№ of patients**		**Effect**		**Quality**	**Importance**
**№ of studies**	**Study design**	**Risk of bias**	**Inconsistency**	**Indirectness**	**Imprecision**	**Other considerations**	**low intensity case management**	**low intensity case management**	**Relative** **(95% CI)**	**Absolute** **(95% CI)**		
Variation from ideal housing (follow up: 12 months; assessed with: 13‐point scale not specified)												
1	randomised trials	very serious^1^	not serious	not serious	serious^2^	none	The intervention group reported less variance from ideal housing at 12 months than at baseline. There was little or no difference in variation from ideal housing for control group from baseline to 12 month follow‐up.				⨁ ◯◯◯ VERY LOW	

**CI:** Confidence interval

1. Risk of performance bias and reporting bias. Inadequate reporting of randomization and allocation concealment methods.

2. Fewer than 400 participants.

##### Table 8.1.7. Comparison 1.B.3 ‐ GRADE Evidence profile for the comparison of low intensity case management vs other intervention (no case management or housing component)

**Author(s)**: Heather Munthe‐Kaas, Rigmor Berg

**Date**: 11.11.2016

**Question**: Low intensity case management compared to other intervention (no case management or housing component) for improving housing stability and reducing homelessness

**Setting**: USA

**Bibliography**: Sorensen 2003; Slesnick 2015

**Table:**

**Quality assessment**							**№ of patients**		**Effect**		**Quality**	**Importance**
**№ of studies**	**Study design**	**Risk of bias**	**Inconsistency**	**Indirectness**	**Imprecision**	**Other considerations**	**low intensity case management**	**other intervention (no case management or housing component)**	**Relative** **(95% CI)**	**Absolute** **(95% CI)**		
Number of participants homeless at follow‐up (follow up: 18 months; assessed with: Not reported)												
1	randomised trials	very serious^1^	not serious	not serious	serious^2^	none	11.3% of participants in the intervention group reported being homeless at 18 month follow‐up compared to 13.8% of participants in the comparison group.				⨁◯◯◯ VERY LOW	
Number of days homeless during 90 days prior to follow‐up (follow up: 18 months; assessed with: self‐report (Form 90))												
1	randomised trials	very serious^3^	not serious	not serious	serious^4^	none	At the 12 month follow‐up participants in the intervention group (N=64) reported 20.51 days (SD=35.13) days homeless compared to 20.85 days (SD=34.95) for participants in the community reinforcement approach group (N=70) and 21.89 days (SD=35.31) for participants in the motivational enhancement therapy group (N=69). All three groups reported a decrease in number of days homeless leading up to the final interview compared to baseline.				⨁◯◯◯ VERY LOW	

**CI:** Confidence interval

1. Risk of performance bias. Inadequate reporting of methods.

2. Fewer than 300 participants.

3. Inadequate reporting of blinding of participants and personnel and outcome assessors.

4. Fewer than 400 participants.

##### Table 8.1.8. Comparison 1.C.1 ‐ GRADE Evidence profile for the comparison of critical time intervention vs usual services

**Author(s)**: Heather Munthe‐Kaas, Rigmor Berg

**Date**: 11.11.2016

**Question**: Critical time intervention compared to usual services for adults who are homeless or at risk of becoming homeless

**Setting**: USA

**Bibliography**: Herman 2011; Susser 1997

**Table:**

**Quality assessment**							**№ of patients**		**Effect**		**Quality**	**Importance**
**№ of studies**	**Study design**	**Risk of bias**	**Inconsistency**	**Indirectness**	**Imprecision**	**Other considerations**	**critical time intervention**	**usual services**	**Relative** **(95% CI)**	**Absolute** **(95% CI)**		
Number of participants who experienced homelessness during study period (follow up: 18 months; assessed with: The Personal History Form ‐ dichotomized to never versus ever homeless)												
1	randomised trials	serious^1^	not serious	not serious	serious^2^	none	3/58 (5.2%)	11/59 (18.6%)	**OR 0.22** (0.06 to ‐0.88)	**138 fewer per 1 000** (from 173 fewer to 439 fewer)	⨁⨁◯◯ LOW	
Number of days homeless (follow up: 18 months; assessed with: The Personal History Form, Total for 18 weeks prior to follow‐up or mean number of days during 30 days prior to each monthly follow‐up interview)												
2	randomised trials	serious^3^	not serious	not serious	serious^4^	none	Participants in the intervention group reported fewer days homeless (M=6) compared to the control group (M=20) (Poisson regression to control for baseline homelessness, p<0.001) (Herman 2011). The intervention group reported approximately one third the number of nights homeless (M=30) as the control group (M=90) (Diff=‐61 (z=2.8, p=.003) (Susser 1997).				⨁⨁◯◯ LOW	
Mean number of nights not homeless over study period (follow up: 18 months; assessed with: Personal History Form assessed for 30 days prior to each monthly follow‐up interview)												
1	randomised trials	serious^5^	not serious	not serious	serious^4^	none	48	48	‐	MD **58 days more** (15.17 more to 100.83 more)	⨁⨁◯◯ LOW	
Total number of nights spent homeless (follow up: 18 months; assessed with: The Personal History Form)												
1	randomised trials	serious^1^	not serious	not serious	serious^4^	none	The intervention roup spent fewer days homeless during the whole study period (1812 nights) than the control group (2403 nights).				⨁⨁◯◯ LOW	
1	randomised trials	serius^6^	not serious	not serious	serious^2^	none	97	113	‐	MD **107.9 days fewer** (136.23 fewer to 79.57 fewer)	⨁⨁◯◯ LOW	

**CI:** Confidence interval; **OR:** Odds ratio; **MD:** Mean difference

1. Risk of selection bias and performance bias.

2. Fewer than 300 participants.

3. Risk of selection bias in one study. Risk of performance bias in both studies. Inadequate reporting of randomization and allocation concealment methods in one study.

4. Fewer than 400 participants.

5. Risk of performance bias. Inadequate reporting of randomization and allocation concealment methods.

6. Inadequate reporting of blinding methods. Risk of reporting bias.

### Category 2: Abstinence‐contingent housing

#### Table 8.2.1: Comparison 2.A.1 – GRADE Evidence profile for the comparison of abstinence‐contingent housing with case management vs usual services

**Author(s)**: Heather Munthe‐Kaas, Rigmor Berg

**Date**: 11.11.2016

**Question**: Abstinence‐contingent housing with case management versus usual services for improving housing stability and reducing homelessness

**Setting**: USA

**Bibliography**: Sosin 1995

**Table:**

**Quality assessment**							**№ of patients**		**Effect**		**Quality**
**№ of studies**	**Design**	**Risk of bias**	**Inconsistency**	**Indirectness**	**Imprecision**	**Other** **considerations**	**abstinence‐contingent housing with case management**	**Usual services**	**Relative** **(95% CI)**	**Absolute** **(95% CI)**	
Housing stability (follow up: 12 months; assessed with: Not reported)											
1 (Sosin 1995)	randomised trials	very serious^1^	not serious	not serious	serious^2^	none	136	187	‐	MD **6.4 days more** (6.18 more to 6.62 more)	⨁◯◯◯ VERY LOW

**CI:** Confidence interval; **MD:** Mean difference

1. Risk of selection bias and attrition bias. Inadequate reporting of blinding of participants, personnel and outcome assessors.

2. Fewer than 400 participants.

#### Table 8.2.2: Comparison 2.A.2 – GRADE Evidence profile for the comparison abstinence‐contingent housing with case management vs case management only

**Author(s)**: Heather Munthe‐Kaas, Rigmor Berg

**Date**: 11.11.2016

**Question**: Abstinence‐contingent housing with case management versus case management only for improving housing stability and reducing homelessness

**Setting**: USA

**Bibliography**: Sosin 1995

**Table:**

**Quality assessment**							**№ of patients**		**Effect**		**Quality**
**№ of studies**	**Design**	**Risk of bias**	**Inconsistency**	**Indirectness**	**Imprecision**	**Other** **considerations**	**abstinence‐contingent housing with case management**	**case management**	**Relative** **(95% CI)**	**Absolute** **(95% CI)**	
Change in number of days housed from baseline to follow‐up (follow up: 12 months; assessed with: Self‐report)											
1 (Sosin 1995)	randomised trials	very serious^1^	not serious	not serious	serious^2^	none	108	70	‐	MD **4.4 days more** (0 to 0)	⨁◯◯◯ VERY LOW

**CI:** Confidence interval; **MD:** Mean difference

1. Risk of selection bias and attrition bias. Inadequate reporting of blinding of participants, personnel and outcome assessors.

2. Fewer than 400 participants.

#### Table 8.2.3: Comparison 2B.1 – GRADE Evidence profile for the comparison abstinence‐contingent housing with day treatment vs usual services

**Author(s)**: Heather Munthe‐Kaas, Rigmor Berg

**Date**: 11.11.2016

**Question**: Abstinence‐contingent housing with day treatment versus usual services for improving housing stability and reducing homelessness

**Setting**: USA

**Bibliography**: Milby 1996

**Table:**

**Quality assessment**							**Impact**	**Quality**
**№ of studies**	**Design**	**Risk of bias**	**Inconsistency**	**Indirectness**	**Imprecision**	**Other** **considerations**		
Change in number of days homeless in past 60 days from baseline to 12 months (follow up: 12 months; assessed with: Personal History Form)								
1 (Milby 1996)	randomised trials	serious^1^	not serious	not serious	serious^2^	none	The mean change in number of days homeless in past 60 days from baseline to 12 months was 0 for the control group. The intervention group had a mean change of 52 fewer days homeless from baseline to 12 months, p=0.026.	⨁⨁◯◯ LOW

**CI:** Confidence interval

1. Risk of performance bias and attrition bias.

2. Less than 400 participants.

#### Table 8.2.4: Comparison 2.B.2 – GRADE Evidence profile for the comparison abstinence‐contingent housing with day treatment vs day treatment only

**Author(s)**: Heather Munthe‐Kaas, Rigmor Berg

**Date**: 11.11.2016

**Question**: Abstinence‐contingent housing with day treatment versus day treatment only for improving housing stability and reducing homelessness

**Setting**: USA

**Bibliography**: Kertesz 2007, Milby 1996

**Table:**

**Quality assessment**							**№ of patients**		**Effect**		**Quality**
**№ of studies**	**Design**	**Risk of bias**	**Inconsistency**	**Indirectness**	**Imprecision**	**Other** **considerations**	**abstinence‐contingent housing with day treatment**	**day treatment**	**Relative** **(95% CI)**	**Absolute** **(95% CI)**	
Changes in mean days housed in past 60 days between baseline and 12 months ‐ self‐report (follow up: 12 months; assessed with: Retrospective Interview for Housing, Employment, and Treatment History)											
2 (Kertesz 2007; Milby 1996)	randomised trials	very serious^1^	not serious	not serious	serious^2^	none	111	103	‐	MD **5.25 days more** (0.34 fewer to 10.83 more)	⨁◯◯◯ VERY LOW
Changes in mean days employed in past 60 days between baseline and 12 months (follow up: 12 months; assessed with: Retrospective Interview for Housing, Employment, and Treatment History ‐ self report)											
2 (Kertesz 2007; Milby 1996)	randomised trials	very serious^1^	not serious	not serious	serious^2^	none	111	103	‐	MD **1.62 days more** (0.99 fewer to 4.22 more)	⨁◯◯◯ VERY LOW

**CI:** Confidence interval; **MD:** Mean difference

1. Risk of performance bias and attrition bias. Inadequate reporting of allocation concealment in both studies.

2. Less than 400 participants.

#### Table 8.2.5: Comparison 2.B.3 – GRADE Evidence profile for the comparison abstinence‐contingent housing with day treatment vs abstinence‐contingent housing with community reinforcement approach

**Author(s)**: Heather Munthe‐Kaas, Rigmor Berg

**Date**: 11.11.2016

**Question**: Abstinence‐contingent housing with day treatment versus non‐abstinence‐contingent housing with day treatment for improving housing stability and reducing homelessness

**Setting**: USA

**Bibliography**: Kertesz 2007

**Table:**

**Quality assessment**							**№ of patients**		**Effect**		**Quality**	**Importance**
**№ of studies**	**Study design**	**Risk of bias**	**Inconsistency**	**Indirectness**	**Imprecision**	**Other considerations**	**abstinence‐contingent housing with day treatment**	**non‐abstinence contingent housing with day treatment**	**Relative** **(95% CI)**	**Absolute** **(95% CI)**		
Days housed ‐ self report Change in mean days housed in past 60 days between baseline and 12 months (follow up: 12 months; assessed with: Retrospective Interview for Housing, Employment, and Treatment History)												
1	randomised trials	very serious ^1^	not serious	not serious	serious ^2^	none	39	43	‐	**MD 3.5 days more** (1.22 more to 5.78 more)	⨁◯◯◯ VERY LOW	CRITICAL

**CI:** Confidence interval; **MD:** Mean difference

1. Risk of performance bias, attrition bias, and inadequate reporting of allocation concealment.

2. Less than 400 participants.

#### Table 8.2.6: Comparison 2.B.3 – GRADE Evidence profile for the comparison abstinence‐contingent housing with day treatment vs abstinence‐contingent housing with community reinforcement approach

**Author(s)**: Heather Munthe‐Kaas, Rigmor Berg

**Date**: 11.11.2016

**Question**: Abstinence‐contingent housing with day treatment versus non‐abstinence‐contingent housing with Community reinforcement approach for improving housing stability and reducing homelessness

**Setting**: USA

**Bibliography**: Smith 1998, Milby 2010

**Table:**

**Quality assessment**							**№ of patients**		**Effect**		**Quality**
**№ of studies**	**Design**	**Risk of bias**	**Inconsistency**	**Indirectness**	**Imprecision**	**Other considerations**	**abstinence‐contingent housing with day treatment**	**abstinence‐contingent housing with community reinforcement approach**	**Relative** **(95% CI)**	**Absolute** **(95% CI)**	
Mean decrease in proportion homelessness (follow up: 4 months; assessed with: Not reported)											
1 (Smith 1998)	randomised trials	very serious^1^	not serious	not serious	serious^2^	none	The rate of homelessness in the intervention group (13.7%) was lower than that in the control group (34%) (χ²(1, N=86)=5.10, p=0.024). There was little or no different at 12 month follow up.				⨁◯◯◯ VERY LOW
Proportion of participants housed more than 40 of past 60 days (follow up: 18 months; assessed with: Retrospective Housing, Employment and Substance Abuse Treatment Interview (RHESAT))
1 (Milby 2010)	randomised trials	very serious^3^	not serious	not serious	serious^2^	none	A greater proportion of participants in the intervention group (44.7%) were housed more than 40 of the previous 60 days at 18 months than in the control group (35.6%). Furthermore, there was a greater increase in pro‐portion of participants housed 40 of the previous 60 days from baseline to 18 months in the intervention group (36%) than in the control group (25.7%).				⨁◯◯◯ VERY LOW

**CI:** Confidence interval

1. Risk of selection bias and detection bias. Inadequate reporting of allocation concealment methods.

2. Less than 400 participants.

3. Risk of selection bias, performance bias and detection bias.

### Category 3: Non‐abstinence‐contingent housing

#### Table 8.3.1: Comparison 3.A.1 – GRADE Evidence Profile for the comparison of Housing First with usual services

**Author(s)**: Heather Munthe‐Kaas, Rigmor Berg

**Date**: 11.11.2016

**Question**: Housing First compared to Usual services for improving housing stability and reducing homelessness

**Setting**: USA, Canada

**Bibliography**: Aubry 2015; Basu 2009; Stefancic 2007; Tsemberis 2004

**Table:**

**Quality assessment**							**№ of patients**		**Effect**		**Quality**	**Importance**
**№ of studies**	**Study design**	**Risk of bias**	**Inconsistency**	**Indirectness**	**Imprecision**	**Other considerations**	**Housing First**	**Usual services**	**Relative** **(95% CI)**	**Absolute** **(95% CI)**		
Number of days homeless (follow up: 18 months; assessed with: Self‐report)												
1	randomised trials	serious^1^	not serious	not serious	not serious	none	201	204	‐	MD **62.5 days fewer** (86.86 fewer to 38.14 fewer)	⨁⨁⨁◯ MODERATE	CRITICAL
Proportion of time homeless (shelter, street or public place) (follow up: 24 months; assessed with: Self‐report)												
1	randomised trials	serious^2^	not serious	not serious	not serious	none	Over the course of the study participants in the Housing First group spent less time homeless (in shelter or on street) (9%) than participants in the control group (24%).				⨁⨁⨁◯ MODERATE	CRITICAL
Number of days in paid housing (follow up: 12 months; assessed with: Self‐report)												
1	randomised trials	serious^1^	not serious	not serious	not serious	none	201	204	‐	MD **110.1 days more** (93.05 more to 127.15 more)	⨁⨁⨁◯ MODERATE	CRITICAL
Proportion of time housed (stable housing includes any long‐term housing arrangement) (follow up: 24 months; assessed with: Residential follow‐back calendar)												
1	randomised trials	serious^2^	not serious	not serious	not serious	none	Over the course of the study participants in the Housing First group spent more time stably housed (73%) than participants in the control group (32%).				⨁⨁⨁◯ MODERATE	CRITICAL
Number of clients placed in permanent housing (follow up: 20 months; assessed with: Unclear)												
1	randomised trials	very serious^3^	not serious	not serious	serious^4^	none	103/209 (49.3%)	13/51 (25.5%)	**RR 1.93** (1.19 to 3.15)	**237 more per 1 000** (from 48 more to 548 more)	⨁⨁◯◯ LOW	IMPORTANT

**CI:** Confidence interval; **MD:** Mean difference; **RR:** Risk ratio

1. Risk of performance bias.

2. Risk of performance bias and detection bias.

3. Risk of selection bias and attrition bias.

4. Fewer than 300 participants.

#### Table 8.3.2: Comparison 3.A.2 – GRADE Evidence Profile for the comparison of Housing First vs abstinence‐contingent housing

**Author(s)**: Heather Munthe‐Kaas, Rigmor Berg

**Date**: 11.11.2016

**Question**: Housing first compared to abstinence‐contingent housing for improving housing stability and reducing homelessness

**Setting**: USA

**Bibliography**: Tsemberis 2004

**Table:**

**Quality assessment**							**№ of patients**		**Effect**		**Quality**	**Importance**
**№ of studies**	**Study design**	**Risk of bias**	**Inconsistency**	**Indirectness**	**Imprecision**	**Other considerations**	**Housing first**	**abstinence‐contingent housing**	**Relative** **(95% CI)**	**Absolute** **(95% CI)**		
Proportion of time spent homeless (follow up: 24 months; assessed with: self‐report)												
1	randomised trials	very serious^1^	not serious	not serious	serious^2^	none	Participants in the control group spent more time homeless over the duration of the study than Housing First group overall: F(1,195)=198, p<0.0001.				⨁◯◯◯ VERY LOW	CRITICAL
Proportion of time stably housed (follow up: 24 months; assessed with: Self‐report)												
1	randomised trials	very serious^1^	not serious	not serious	serious^2^	none	Participants in the Housing First group had faster increases in stably housed status compared to participants in the control condition: F(4, 137)=27.7, p<0.001)				⨁◯◯◯ VERY LOW	CRITICAL

**CI:** Confidence interval

1. Risk of detection bias and attrition bias. Inadequate reporting of randomization, allocation concealment and blinding of participants and personnel.

2. Fewer than 300 participants.

#### Table 8.3.3: Comparison 3.B.1 – GRADE Evidence Profile for comparison of non‐abstinence‐contingent housing with high intensity case management with usual services

**Author(s)**: Heather Munthe‐Kaas, Rigmor Berg

**Date**: 11.11.2016

**Question**: Non‐abstinence‐contingent housing with high intensity case management compared to usual services for improving housing stability and reducing homelessness

**Setting**: USA

**Bibliography**: Shern 2000

**Table:**

**Quality assessment**							**№ of patients**		**Effect**		**Quality**	**Importance**
**№ of studies**	**Study design**	**Risk of bias**	**Inconsistency**	**Indirectness**	**Imprecision**	**Other considerations**	**non‐abstinence‐contingent housing with high intensity case management**	**usual services**	**Relative** **(95% CI)**	**Absolute** **(95% CI)**		
Change in proportion of time spent homeless (streets) (follow up: 24 months; assessed with: self‐report)												
1	randomised trials	serious^1^	not serious	not serious	serious^2^	none	91	77	‐	MD **26.7 percent lower** (39.21 lower to 14.21 lower)	⨁⨁◯◯ LOW	
Change in proportion of time spent in shelter (follow up: 24 months; assessed with: self‐report)												
1	randomised trials	serious^1^	not serious	not serious	serious^2^	none	91	77	‐	MD **20.3 percent higher** (13.38 higher to 27.2 higher)	⨁⨁◯◯ LOW	
Change in proportion of time spent in community living (follow up: 24 months; assessed with: self‐report)												
1	randomised trials	serious^1^	not serious	not serious	serious^2^	none	91	77	‐	MD **11.1 percent higher** (1.5 higher to 20.6 higher)	⨁⨁◯◯ LOW	

**CI:** Confidence interval; **MD:** Mean difference

1. Inadequate reporting of allocation concealment measures and blinding.

2. Fewer than 400 participants.

#### Table 8.3.4: Comparison 3.B.2 – GRADE Evidence Profile for comparison of non‐abstinence‐contingent group living arrangements with high intensity case management compared to non‐abstinence‐contingent independent apartments with high intensity case management

**Author(s)**: Heather Munthe‐Kaas, Rigmor Berg

**Date**: 11.11.2016

**Question**: Non‐abstinence‐contingent group living arrangements with high intensity case management compared to non‐abstinence‐contingent independent apartments with high intensity case management for improving housing stability and reducing homelessness

**Setting**: USA

**Bibliography**: Goldfinger 1999

**Table:**

**Quality assessment**							**№ of patients**		**Effect**		**Quality**	**Importance**
**№ of studies**	**Study design**	**Risk of bias**	**Inconsistency**	**Indirectness**	**Imprecision**	**Other considerations**	**non‐abstinence‐contingent group living arrangements with high intensity case management**	**non‐abstinence‐contingent independent apartments with high intensity case management**	**Relative** **(95% CI)**	**Absolute** **(95% CI)**		
Housing status ‐ housed (follow up: 18 months; assessed with: point in time ‐ self‐report, records of the housing facilities, and Department of mental health, weekly logs from case managers)												
1	randomised trials	serious^1^	not serious	not serious	very serious^2^ ^,^ ^3^	none	47/61 (77.0%)	37/49 (75.5%)	**RR 1.02** (0.83 to 1.26)	**15 more per 1 000** (from 128 fewer to 196 more)	⨁◯◯◯ VERY LOW	CRITICAL
Housing status ‐ not housed (follow up: 18 months; assessed with: point in time ‐ self‐report, records of the housing facilities, and Department of mental health, weekly logs from case managers)												
1	randomised trials	serious^1^	not serious	not serious	very serious^2^ ^,^ ^3^	none	14/61 (23.0%)	12/49 (24.5%)	**RR 0.94** (0.48 to 1.84)	**15 fewer per 1 000** (from 127 fewer to 206 more)	⨁◯◯◯ VERY LOW	CRITICAL
Total days homeless after rehousing (follow up: 18 months; assessed with: self‐report, records of the housing facilities, and Department of mental health, weekly logs from case managers)												
1	randomised trials	serious^1^	not serious	not serious	serious^2^	none	“log [+1]=.99 for 61 study participants in group homes compared with 1.8 for 51 study participants in independent apartments; t=–1.85, df=97 [unequal variances], p<.05, one‐tailed”				⨁⨁◯◯ LOW	CRITICAL
Mean number of days homeless (follow up: 18 months; assessed with: self‐report, records of the housing facilities, and Department of mental health, weekly logs from case managers)												
1	randomised trials	serious^1^	not serious	not serious	serious^2^	none	Participants in the group housing intervention reported a mean of 43 days homeless over the 18 month study period compared to a mean of 78 days reported by participants in the independent housing intervention.				⨁⨁◯◯ LOW	CRITICAL
Number of participants who are homeless (shelter) (follow up: 18 months; assessed with: self‐report, records of the housing facilities, and Department of mental health, weekly logs from case managers)												
1	randomised trials	serious^1^	not serious	not serious	very serious^2^ ^,^ ^3^	none	8/61 (13.1%)	8/49 (16.3%)	**RR 0.80** (0.32 to 1.99)	**33 fewer per 1 000** (from 111 fewer to 162 more)	⨁◯◯◯ VERY LOW	
Number of participants who are homeless (streets) (follow up: 18 months; assessed with: self‐report, records of the housing facilities and Department of mental health, weekly logs from case managers)												
1	randomised trials	serious^1^	not serious	not serious	very serious^2^ ^,^ ^3^	none	2/61 (3.3%)	4/49 (8.2%)	**RR 0.40** (0.08 to 2.10)	**49 fewer per 1 000** (from 75 fewer to 90 more)	⨁◯◯◯ VERY LOW	

**CI:** Confidence interval; **RR:** Risk ratio

1. Inadequate reporting of randomization, allocation concealment and blinding.

2. Fewer than 300 participants.

3. Wide confidence interval.

#### Table 8.3.5. Comparison 3.B.3 – GRADE Evidence Profile for comparison of non‐abstinence‐contingent housing with high intensity case management vs abstinence‐contingent housing with high intensity case management

**Author(s)**: Heather Munthe‐Kaas, Rigmor Berg

**Date**: 11.11.2016

**Question**: Non‐abstinence‐contingent housing with high intensity case management compared to Abstinence‐contingent housing with high intensity case management for improving housing stability and reducing homelessness

**Setting**: USA

**Bibliography**: McHugo 2004

**Table:**

**Quality assessment**							**№ of patients**		**Effect**		**Quality**	**Importance**
**№ of studies**	**Study design**	**Risk of bias**	**Inconsistency**	**Indirectness**	**Imprecision**	**Other considerations**	**Non‐abstinence‐contingent housing with high intensity case management**	**Abstinence‐contingent housing with high intensity case management**	**Relative** **(95% CI)**	**Absolute** **(95% CI)**		
Proportion of days homeless (follow up: 18 months; assessed with: Residential Follow‐back Calendar)												
1	randomised trials	very serious^1^	not serious	not serious	serious^2^	none	There was a greater change in number of days homeless among members of the comparison group over the study period (F=6.07, p<0.05, d=‐0.52).				⨁◯◯◯ VERY LOW	
Proportion of days in stable housing (follow up: 18 months; assessed with: Residential Follow‐back Calendar)												
1	randomised trials	very serious^1^	not serious	not serious	serious^2^	none	At the end of the study 68.1% of participants in the intervention group were in stable housing compared to 85.5 % of comparison group participants (F=5.99, p<0.05, d=0.51).				⨁◯◯◯ VERY LOW	

**CI:** Confidence interval

1. Risk of attrition bias. Inadequate reporting of randomization, allocation concealment and blinding.

2. Fewer than 300 participants.

#### Table 8.3.6.: Comparison 3.B.4 – GRADE Evidence Profile for comparison of non‐abstinence‐contingent housing with day treatment with usual services

**Author(s)**: Heather Munthe‐Kaas, Rigmor Berg

**Date**: 11.11.2016

**Question**: Non‐abstinence contingent housing with day treatment compared to day treatment for improving housing stability and reducing homelessness

**Setting**: USA

**Bibliography**: Kertesz 2007

**Table:**

**Quality assessment**							**№ of patients**		**Effect**		**Quality**	**Importance**
**№ of studies**	**Study design**	**Risk of bias**	**Inconsistency**	**Indirectness**	**Imprecision**	**Other considerations**	**non‐abstinence contingent housing with day treatment**	**day treatment**	**Relative** **(95% CI)**	**Absolute** **(95% CI)**		
Changes in mean days housed in past 60 days between baseline and 12 months (follow up: 12 months; assessed with: Retrospective Interview for Housing, Employment, and Treatment History)												
1	randomised trials	very serious^1^	not serious	not serious	serious^2^ ^,^ ^3^	none	43	34	‐	MD **4.7 days more** (9.38 fewer to 18.78 more)	⨁◯◯◯ VERY LOW	
Change in mean days employed in past 60 days between baseline and 12 months (follow up: 12 months; assessed with: Retrospective Interview for Housing, Employment, and Treatment History)												
1	randomised trials	very serious^1^	not serious	not serious	serious^2^ ^,^ ^3^	none	43	34	‐	MD **5.7 days more** (8.4 fewer to 19.8 more)	⨁◯◯◯ VERY LOW	

**CI:** Confidence interval; **MD:** Mean difference

1. Risk of selection bias and attrition bias. Inadequate reporting of allocation concealment methods and blinding.

2. Fewer than 300 participants.

3. Wide confidence interval.

### Category 4: Housing vouchers with case management

#### Table 8.4.1: Comparison 4A.1 – GRADE Evidence Profile for comparison of housing vouchers with case management with usual services

**Author(s)**: Heather Munthe‐Kaas, Rigmor Berg

**Date**: 11.11.2016

**Question**: Housing vouchers with case management compared to usual services for improving housing stability and reducing homelessness

**Setting**: USA

**Bibliography**: Hurlburt 1996; Levitt 2013; Rosenheck 2003; Wolitski 2010

**Table:**

**Quality assessment**							**№ of patients**		**Effect**		**Quality**	**Importance**
**№ of studies**	**Study design**	**Risk of bias**	**Inconsistency**	**Indirectness**	**Imprecision**	**Other considerations**	**housing vouchers with case management**	**usual services**	**Relative** **(95% CI)**	**Absolute** **(95% CI)**		
Time to first exit from shelter (follow up: 12 months; assessed with: Not reported)												
1	randomised trials	serious^1^	not serious	not serious	serious^2^	none	The intervention group experienced fewer days to exit x^2^1 = 6.068, 95%CI = 0.589, 0.942		not estimable		⨁⨁◯◯ LOW	
Time to return to shelter (follow up: 12 months; assessed with: Not reported)												
1	randomised trials	serious^1^	not serious	not serious	serious^2^	none	The intervention group reported longer time to return to shelter than the control group x21 = 6.524, 95% CI = 0.379, 0.880		not estimable		⨁⨁◯◯ LOW	
Number of days housed during 90 days prior to follow‐up (follow up: range 18 months to 36 months; assessed with: Not reported)												
1	randomised trials	serious^2^	not serious	not serious	serious^2^	none	Rosenheck 2003 (Intervention N=182, Control N=188) Intervention: 59.39 days housed, Control: 47.60 days housed. t=4.88, p<0.001;				⨁⨁◯◯ LOW	
Number of days homeless during 90 days prior to follow‐up (follow up: range 18 months to 36 months; assessed with: Not reported)												
1	randomised trials	serious^2^	not serious	not serious	serious^2^	none	Rosenheck 2003 (Intervention N=182, Control N=188) Intervention: 13.05 days homeless, Control 20.45 days homeless, t=3.56, p<0.001.				⨁⨁◯◯ LOW	
Proportion of participants who were in their own home at follow‐up (follow up: range 18 months; assessed with: Not reported)												
1	randomised trials	serious^2^	not serious	not serious	serious^2^	none	More participants from the Intervention group reported being in their own home during the previous 90 days (82.48%; n=315) than in control group (50.58%; n=315)				⨁⨁◯◯ LOW	
Proportion of participants who were homeless one or more nights during the 90 days prior to follow‐up (follow up: range 18 months; assessed with: Not reported)												
1	randomised trials	serious^2^	not serious	not serious	serious^2^	none	A greater proportion of participants in the Intervention group reported being homeless one or more nights during previous 90 days (2.55%; n=315) than control group (5.22%; n=315).				⨁⨁◯◯ LOW	
Proportion of participants in transitional settings or living with others at follow‐up (assessed with: Not reported)												
1	randomised trials	serious^2^	not serious	not serious	serious^2^	none	A greater proportion of participants in the intervention group reported living in transitional settings or temporarily living with others (14.96%; n=315) compared to the control group (44.40%; n=315).				⨁⨁◯◯ LOW	

**CI:** Confidence interval

1. Risk of performance bias and detection bias. Inadequate randomization methods.

2. One small study.

#### Table 8.4.2: Comparison 4.2 – GRADE Evidence Profile for comparison of housing vouchers with case management with case management

**Author(s)**: Heather Munthe‐Kaas, Rigmor Berg

**Date**: 11.11.2016

**Question**: Housing vouchers with case management compared to case management for adults with mental illness

**Setting**: USA

**Bibliography**: Hurlburt 1996, Rosenheck 2003

**Table:**

**Quality assessment**							**№ of patients**		**Effect**		**Quality**	**Importance**
**№ of studies**	**Study design**	**Risk of bias**	**Inconsistency**	**Indirectness**	**Imprecision**	**Other considerations**	**housing vouchers with case management**	**case management**	**Relative** **(95% CI)**	**Absolute** **(95% CI)**		
Number of participants in independent housing (follow up: 24 months; assessed with: case manager records)												
1	randomised trials	serious^1^	not serious	not serious	serious^3^	none	104/181 (57.5%)	55/181 (30.4%)	**RR 1.89** (1.47 to 2.44)	**270 more per 1 000** (from 143 more to 438 more)	⨁⨁◯◯ LOW	
Number of participants living in community housing (follow up: 24 months; assessed with: case manager records)												
1	randomised trials	serious^1^	not serious	not serious	serious^3^	none	11/181 (6.1%)	44/181 (24.3%)	**RR 0.25** (0.13 to 0.47)	**182 fewer per 1 000** (from 129 fewer to 211 fewer)	⨁⨁◯◯ LOW	
Number of participants living in variable housing situations (follow up: 24 months; assessed with: case manager records)												
1	randomised trials	serious^1^	not serious	not serious	serious^3^	none	66/181 (36.5%)	82/181 (45.3%)	**RR 0.80** (0.63 to 1.03)	**91 fewer per 1 000** (from 14 more to 168 fewer)	⨁⨁◯◯ LOW	
Number of days in stable housing (follow up: 36 months; assessed with: self‐report)												
1	randomised trials	serious^2^	not serious	not serious	serious^3^	none	Participants in the intervention group eported more days in stable housing than the control group (M=59.39 vs M=50.81), t=2.90, p<0.004				⨁⨁◯◯ LOW	
Number of days spent homeless (follow up: 36 months; assessed with: self‐report)												
1	randomised trials	serious^2^	not serious	not serious	serious^3^	none	Participants in the intervention group reported fewer days homeless than the control group (M=13.04 s M=20‐33), t=2.87, p=0.004				⨁⨁◯◯ LOW	

**CI:** Confidence interval; **RR:** Risk ratio

1. Inadequate reporting of methods.

2. Risk of performance bias. Inadequate reporting of methods for blinding of outcome assessors.

3. Fewer than 400 participants.

### Category 5: Residential treatment

#### Table 8.5.1: Comparison 5.1‐ GRADE Evidence Profile of comparison residential treatment with case management vs usual services

**Author(s): Heather Munthe‐Kaas, Rigmor Berg**

**Date**: 11.11.2016

**Question**: Residential treatment with case management compared to Usual services for adults with mental illness and/or substance abuse issues

**Setting**: USA

**Bibliography**: Lipton 1988; Conrad 1998

**Table:**

**Quality assessment**							**№ of patients**		**Effect**		**Quality**	**Importance**
**№ of studies**	**Study design**	**Risk of bias**	**Inconsistency**	**Indirectness**	**Imprecision**	**Other considerations**	**Residential treatment with case management**	**Usual services**	**Relative** **(95% CI)**	**Absolute** **(95% CI)**		
Proportion of nights homeless (follow up: range 12 months to 24 months; assessed with: Personal History Form)												
2	randomised trials	very serious^1^	not serious	not serious	not serious	none	Participants in the intervention group in both studies reported less homelessness than participants in the control group.				⨁⨁◯◯ LOW	
Proportion of time in stable housing (follow up: 12 months; assessed with: Unclear)												
1	randomised trials	serious^2^	not serious	not serious	serious^3^	none	Participants in the intervention group (N=26; 79%, SD=26) reported being in permanent housing more than twice as much as the control group (N=23; 33% SD=36) during the study year (t2=4.32, df=32, p=0.0001).				⨁⨁◯◯ LOW	
Number of participants stably housed at follow‐up (follow up: 12 months; assessed with: Unclear)												
1	randomised trials	serious^2^	not serious	not serious	serious^3^	none	More than twice as many participants from the intervention group reported being in permanent housing at the 12 month follow‐up interview (69% compared to 30%).				⨁⨁◯◯ LOW	

**CI:** Confidence interval

1. Risk of attrition bias, reporting bias in one study. Inadequate reporting of methods in both studies.

2. Inadequate reporting of methods.

3. Fewer than 400 participants.

### Appendix 9: Description of studies in progress

#### Table 9.1: Studies in progress

**Table:**

**Study (ref)**	**Description of the study**
Tinland 2013(82)	This is a randomized trial aiming to assess the effect of Housing First on health outcomes and costs for adults who are homeless are at risk of becoming homless and have either schizophrenia, bipolar disorder or a severe disability. Housing stability is a secondary outcome. The interevention will be compared to usual services and the participants will be followed‐up for a period of 24 months.
Smelson 2015(31)	This is a cluster randomized trial to assess the effect of housing program for veterans with case management (and substance abuse treatment) called Maintaining Independence and Sobriety Through Systems Integration, Outreach, and Networking—Veterans Edition (MISSION‐Vet). The research team will assess this program implemented as usual versus the same program using an implementation strategy called Getting To Outcomes. The primary outcomes are housing, mental health, and substance abuse.
