# Corrigendum to “Effectiveness of interventions to reduce homelessness: A systematic review and meta‐analysis”

**DOI:** 10.1002/cl2.1333

**Published:** 2023-07-28

In Table 21 on page 62, two numbers for Basu 2012 in rows 3 and 4 were mixed up. It did not affect any other outcome or result.

Rows 3 and 4 should be:

**Table:** 

		**Intervention**, mean (SD)	**Usual care**, mean (SD)	**MD (SE)**	**95% CI on MD**
Basu 2012	No. days homeless	121 (120)	183.5 (130)	−62.3 (12.4)	[−86.6 to −38.0]
Basu 2012	No. days in paid housing	112 (122)	1.9 (18)	109.9 (8.7)	[92.8 to 127.0]

We apologize for this error.
